# Genomic and developmental characterisation of a novel bunyavirus infecting the crustacean *Carcinus maenas*

**DOI:** 10.1038/s41598-019-49260-4

**Published:** 2019-09-10

**Authors:** Jamie Bojko, Kuttichantran Subramaniam, Thomas B. Waltzek, Grant D. Stentiford, Donald C. Behringer

**Affiliations:** 1 Fisheries and Aquatic Science, University of Florida, Gainesville, Florida 32653 USA; 2 Emerging Pathogens Institute, University of Florida, Gainesville, Florida 32611 USA; 3 Department of Infectious Diseases and Immunology, College of Veterinary Medicine, University of Florida, Gainesville, FL 32610 USA; 4 International Centre of Excellence for Aquatic Animal Health, Centre for Environment, Fisheries and Aquaculture Science, Weymouth, Dorset DT4 8UB UK; 5 Centre for Sustainable Aquaculture Futures, College of Life and Environmental Sciences, University of Exeter, Stocker Road, Exeter, EX4 4QD UK

**Keywords:** Metagenomics, Virology, Microbial ecology

## Abstract

*Carcinus maenas* is in the top 100 globally invasive species and harbours a wide diversity of pathogens, including viruses. We provide a detailed description for a novel bunyavirus (Carcinus maenas Portunibunyavirus 1) infecting *C*. *maenas* from its native range in the Faroe Islands. The virus genome is tripartite, including large (L) (6766 bp), medium (M) (3244 bp) and small (S) (1608 bp) negative sense, single-stranded RNA segments. Individual genomic segments are flanked by 4 bp regions of similarity (CCUG). The segments encode an RNA-dependent RNA-polymerase, glycoprotein, non-structural protein with a Zinc-Finger domain and a nucleoprotein. Most show highest identity to the ‘Wenling Crustacean Virus 9’ from an unidentified crustacean host. Phylogenomics of crustacean-infecting bunyaviruses place them across multiple bunyavirus families. We discuss the diversity of crustacean bunyaviruses and provide an overview of how these viruses may affect the health and survival of crustacean hosts, including those inhabiting niches outside of their native range.

## Introduction

The order *Bunyavirales* was erected in 2017 to contain a group of viruses with negative sense single-strand ribonucleic acid (-ssRNA) genomes composed of 2–4 segments^1–3^. The order contains 10 families comprised of 215 recognised species^1,3^, including viruses that infect humans^4^, other vertebrates^5^, invertebrates^6^ and plants^7^ from a wide range of terrestrial, marine and freshwater ecosystems. To date, a single viral family exists to contain crustacean-infecting bunyaviruses, the *Cruliviridae*, which contains a single member, the ‘*Wenling Lincruvirus*’^3^.

Although numerous bunya-like viruses have been tentatively proposed to infect crustacean hosts, formal taxonomic descriptions have not been reported for most. These include several bunya-like virus infections in the portunid crabs *Carcinus maenas* (“Crab Haemocytic Virus”, “Y-organ virus”, “Roscoff virus”^8–11^); *Carcinus mediterraneus* (“S-virus” and “Y-organ virus”^10–12^) and *Macropipus* (=*Necora) depurator* (“S-virus”^12^); the cancrid crab *Cancer pagurus* (“Cancer pagurus Systemic Bunya-like Virus”^13^); the varunid crab *Eriocheir sinensis* (bunya-like virus GenBank accession number KM405247); and Mourilyan virus (MoV) from *Penaeus* sp. (bunya-like virus GenBank accession number AAY15205). Several viruses detected via broad virome screening programmes on tissue and environmental samples include the “Athtab virus” from the crayfish *Cherax quadricarinatus*^14^; “Wenzhou shrimp virus 1” and “Wenzhou shrimp virus 2” from *Penaeus* sp.^15^; and Wenling crustacean viruses 7 and 9 from unidentified crustacean hosts contained within pooled samples^16^. Wenling crustacean virus 9 is currently the only crustacean-infecting bunyavirus recognised by the ICTV, where it represents the sole member within the genus *Lincruvirus* in the family *Cruliviridae*^3^.

Although genomic data for the viruses infecting *C*. *maenas*, *C*. *mediterraneus*, *M*. *depurator* and *C*. *pagurus* are lacking, histological and ultrastructural data have provided insight into host-pathogen interactions in these crustacean-infecting bunyaviruses. In *C*. *maenas*, infection of the cytoplasm of haemocytes and amoebocytes of the gill, inducing haemocytopoenia and subsequent reduced clotting ability in the blood is noted^8,17^. Transmission to uninfected conspecifics following injection of filtered haemolymph has been associated with high rates of mortality in experimental infections of *M*. *depurator*^12^. Finally, the bunya-like virus reported from *C*. *pagurus* likely infects haemocytes, with high viral burdens detectable within the haemolymph of infected crabs^13^. These viruses have been associated with disease when crustaceans are retained in close proximity, while little data exists for prevalence and disease in wild populations^14,17^. For those bunya-like viruses detected in crustaceans via nucleotide sequencing methods, no pathology data is available; however, the genomic information associated with these discoveries provides important insights into the potential diversity, taxonomy and evolution within the order. As an example, the *Wenling Lincruvirus*, sequenced from an unidentified crustacean host, showed the typical bunyavirus genome structure of three genomic segments [Large (L), Medium (M) and Small (S)] encoding between three and six proteins: an RNA-dependent-RNA-polymerase (RdRp); non-structural proteins; a nucleocapsid protein; and either two glycoprotein subunits or a pro-glycoprotein. Additionally, Sakuna *et al*.^14^ showed that the RdRp and glycoprotein (G1) amino acid (AA) sequences of crustacean-infecting bunya-like viruses were phylogenetically related^14^, except for in a few specific isolates (Wenzhou Shrimp virus 1 and Wenling crustacean virus 7).

In this study we describe a bunyavirus from the portunid crab *C*. *maenas* collected from the Faroe Islands (north Atlantic). Our genetic, phylogenetic and developmental data show that the virus is a novel member in the order *Bunyavirales* and is a representative of the family *Cruliviridae* in a newly suggested viral genus (Portunibunyavirus) pending ICTV affirmation and is referred to herein as ‘Carcinus maenas Portunibunyavirus 1’ (CmPBV1). The information presented here updates the original morphological description of CmPBV1 (then described as an “irido-like virus”) from *C*. *maenas*^18^. In addition, we explore the phylogenetic and gene-similarity data for all known crustacean-infecting bunyaviruses and explore their evolutionary relationships and links to ecological effects.

## Results

### Transmission electron microscopy of the putative viral development pathway

**Figure 1:**
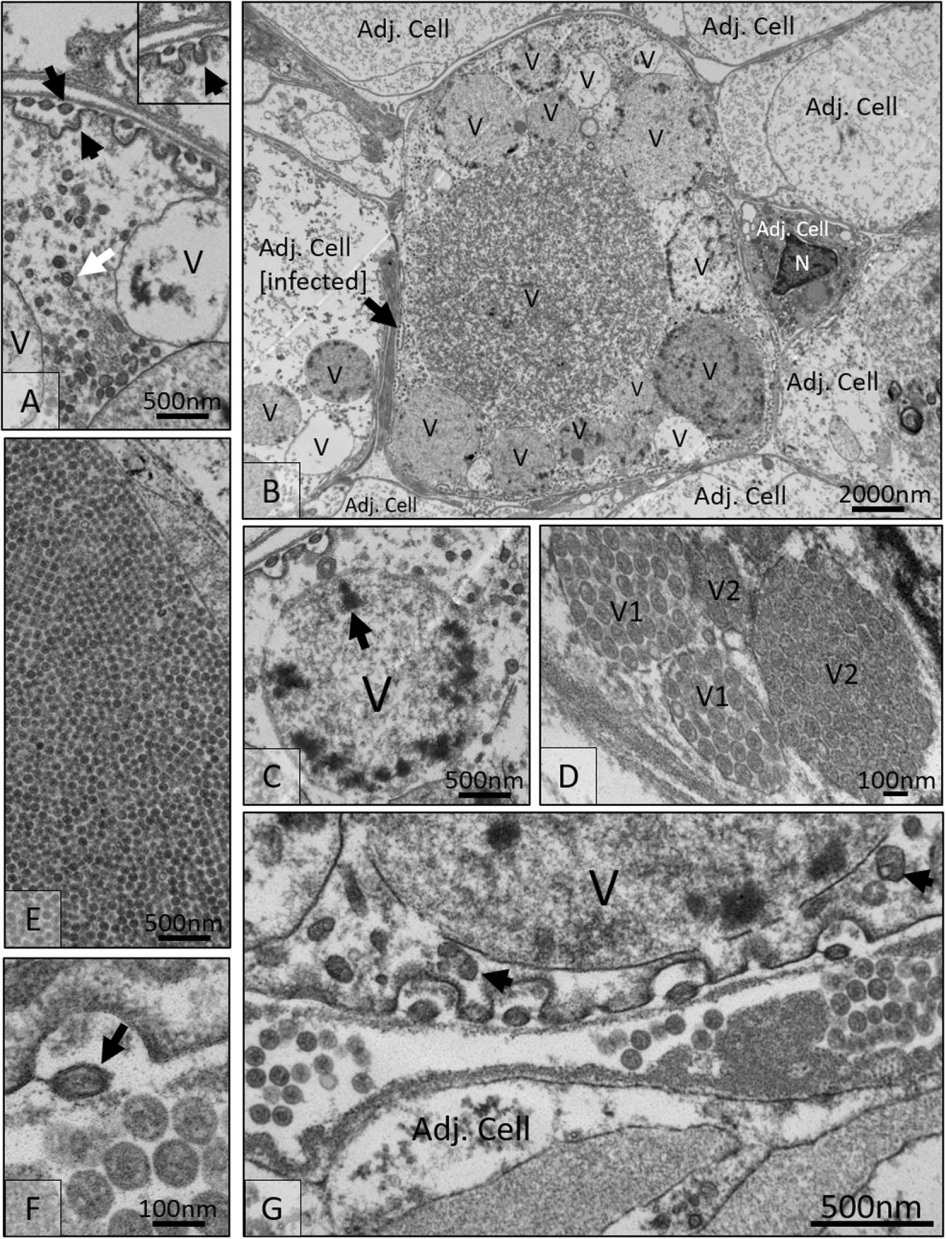
Electron micrographs of the developmental cycle for ‘Carcinus maenas Portunibunyavirus 1’. (**A**) Single membrane viruses exit/enter the cell via cytosis of the cell membrane (black arrows) and enter the cell with a double membrane (white arrow) vesicles (V) including developing viral particles are present in section. (**B**) A lower magnification image shows an infected cell, among adjacent cells, with multiple vesicles containing developing virions. The nucleus (N) of an adjacent cell can be identified. (**C**) An image of an early developing vacuole with electron dense material, predicted to be viral RNA undergoing translation/transcription. (**D**) Virions are assembled. Vacuoles with late stage virions (V1) and pre-full-assembly virions are present in section (V2). (**E**) In some infected cells a paracrystalline array was noted to form in the cytoplasm of the host. F) As virions leave the cell, they shed their second membrane (black arrow). (**G**) Finally, infective virions enter the interstitial space between cells and aggregate before moving on to adjacent cells.

**Figure 2:**
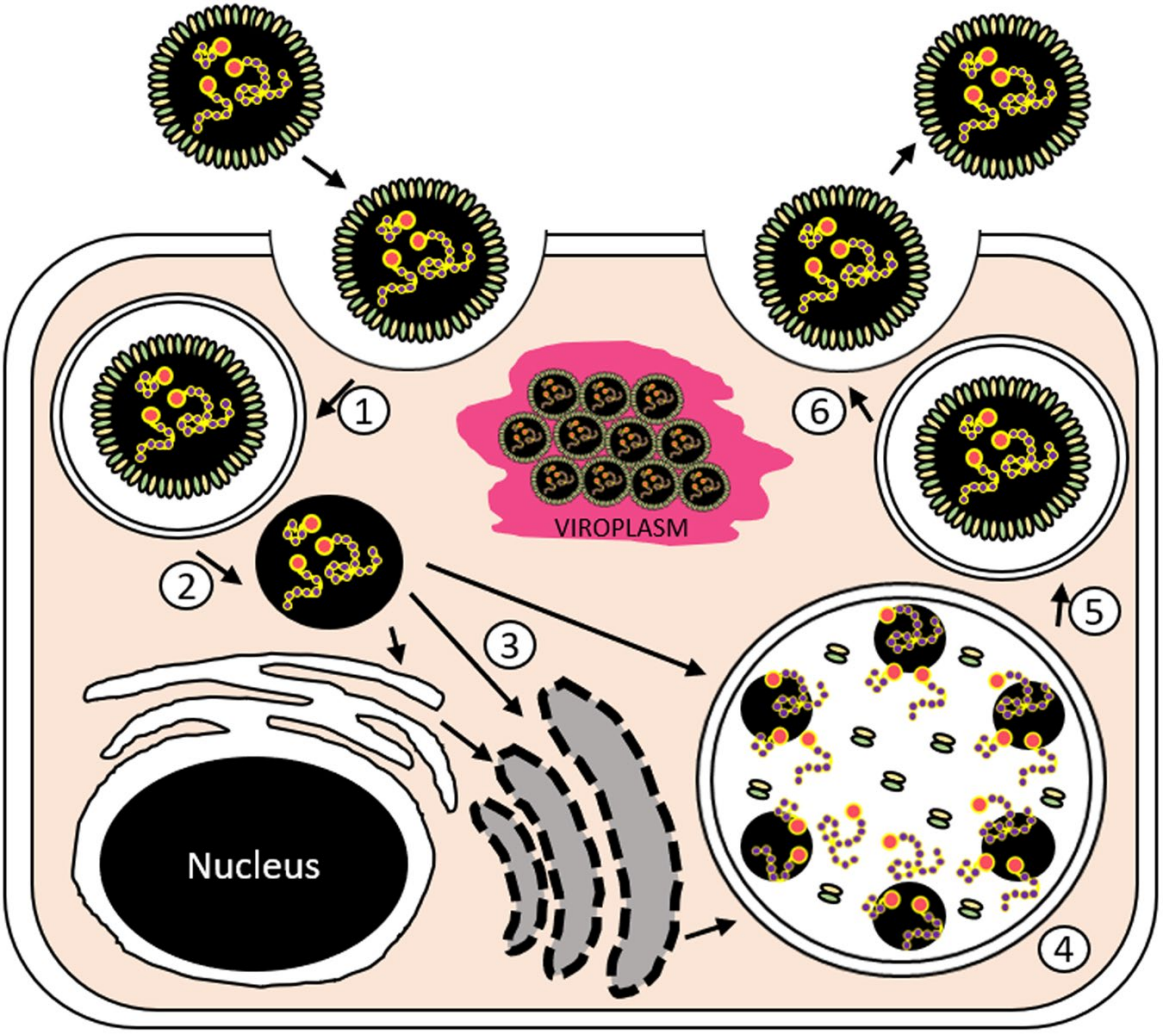
A graphical representation of the putative viral development for ‘Carcinus maenas Portunibunyavirus 1’ based on transmission electron micrographs attained from infected gill tissue. Infective stages enter the cell through endocytosis (1/Fig. 1A), providing them with a second membrane. This membrane is then predicted to shed (2), allowing release of viral -ssRNA. The genetic material then interacts with the cell, possibly through multiple pathways yet to be defined (3) but result in a series of large vacuoles where viral assembly occurs (4/Fig. 1C). After assembly, viral particles move from the assembly vesicle (5/Fig. 1D,G) and exit the cell (6/Fig. 1A). In addition to the classical bunyaviral development cycle, multiple occurrences of viroplasm development occur, suggesting a build-up of virions in the cytoplasm of the host cell which may rupture to result in virion release (“VIROPLASM”) (Fig. 1E). The diagram is not to cellular scale.

The development of the virus is predicted from transmission electron micrographs of infected gill tissues. Infection by CmPBV1 began with single membrane bound virions that apparently entered the host cell through endocytosis before acquiring a second membrane with low-level structural integrity, eliciting the appearance of variable shape virions (Fig. 1A). Infected host cells were populated by a series of large and small vesicles containing various numbers of developing virions, which appeared to become progressively more electron dense as virions matured prior to their exit from the vesicle (Fig. 1B). Within early vesicles, condensed, electron-dense material of unknown composition was observed at the periphery of the vesicle (Fig. 1C). Expansion in the size of these large vesicles preceded the release of smaller vesicles containing pre-assembled mature virions, until they became empty (Fig. 1D). In some instances, these virions were present in large vacuoles forming a distinctive paracrystalline array (Fig. 1E); possibly representing an alternative pathway of viral release through cell lysis. Those virions that exited the vacuole in a manner previously observed in the bunyaviruses acquired a second membrane, which was retained (albeit again with relatively low structural integrity) during movement through the cell cytoplasm before exiting the cell into the interstitial space (Fig. 1F,G), where they formed small aggregations of uniformly shaped viral particles, prior to proposed infection of adjacent cells (Fig. 1G). A diagrammatic representation of the putative developmental pathway for CmPBV1 is given in Fig. 2.

### Whole genome sequencing, assembly, and genome annotation

**Figure 3:**
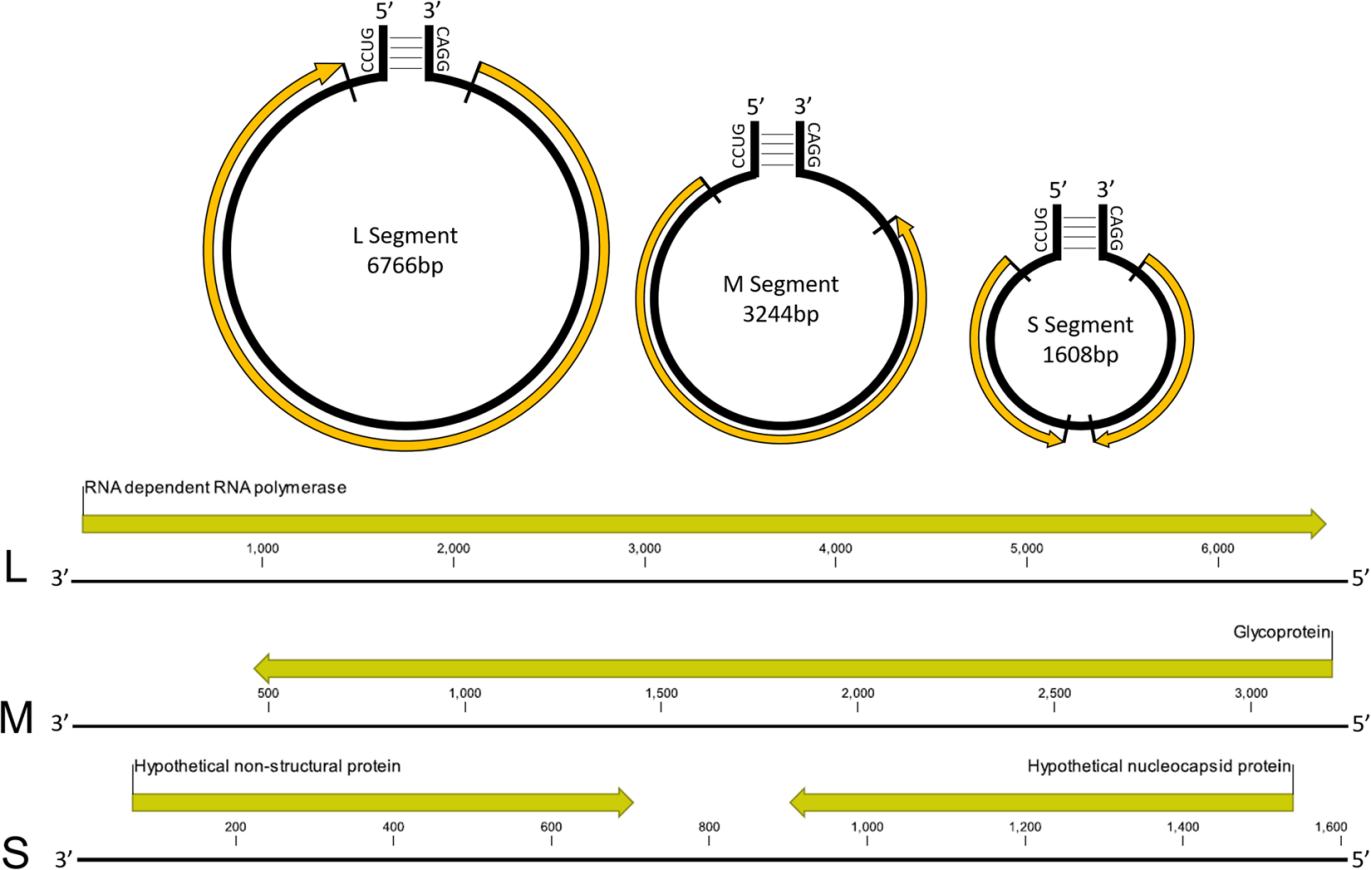
The annotated -ssRNA genome of ‘Carcinus maenas Portunibunyavirus 1’ and graphical representation of the three -ssRNA genomic segments with annotation for the bunyavirus-associated open reading frames. The predicted protein is listed above the annotation and the complementary bunyaviral ends are represented.

The genome of CmPBV1 was comprised of three segments corresponding with previously defined categories of L, M and S bunyaviral genomic segment nomenclature. The large (L) segment, 6676 bp was represented by 3470X sequence coverage. The medium (M) segment, 3244 bp was represented by 2439X sequence coverage. The small (S) segment, 1608 bp was represented by 10274X coverage (~3X greater coverage than the M and L segments). Each genomic segment began and ended in complementary RNA sequences of “3′-CAGG–CCUG-5′”, respectively (Fig. 3). The L segment of the CmPBV1 genome encoded one predicted ORF encoding an RdRp (Fig. 3; Table 1). The M segment encoded one predicted ORF with 65% identity to the pre-glycoprotein gene of the *Wenling Lincruvirus* (accession: YP009329880) (Fig. 3; Table 1). The S segment encoded two predicted ORFs, including one that showed 61% identity to the predicted nucleoprotein of the *Wenling Lincruvirus* (accession: YP009329881) (Fig. 3; Table 1). The likelihood of the protein being a nucleocapsid protein was further confirmed by VIRALpro (distance = 1.691815). The second ORF on the S segment did not show AA sequence similarity to any protein from other bunyavirus isolates and may be a divergent non-structural protein (NSs) (Table 1). This protein includes a Zinc-Finger binding site at AA 97-117. The predicted ORFs resulted in proteins with a range of molecular functions proposed in Table 1 and were associated with various stages of viral development.

**Table 1:** Hypothetical open reading frames from the cDNA of the -ssRNA genome compared to other protein sequences using the NCBI database.

Segment	Hypothetical protein	Translatory frame and cDNA sequence orientation	Predicted molecular function	Protein sequence similarity				Predicted lifecycle function
				Accession number and isolate description	Coverage (%)	Similarity (%)	E-value	
L	RdRp (2171aa)	2^nd^ Frame (5′-3′)	RNA genome replication	RdRp of ‘Wenling crustacean virus 9’ (YP009329879)	100	72	0.0	Replication
M	Glycoprotein (914aa)	2^nd^ Frame (3′-5′)	Membrane glycoprotein	glycoprotein ‘Wenling crustacean virus 9’ (YP009329880)	100	65	0.0	Viral entry into host cell
S	Hypothetical protein (211aa)	3^rd^ Frame (5′-3′)	Unknown	—	—	—	—	Zn Finger domain suggesting nucleotide binding
S	Putative nucleoprotein (212aa)	3^rd^ Frame (3′-5′)	Genome-binding properties	nucleoprotein ‘Wenling crustacean virus 9’ (YP009329881)	100	61	2e-87	Genome packaging after replication

The cDNA sequence was not adequately similar to any available isolate, so only protein comparison data are presented. Similarity searches were conducted in NCBI BLASTP.

### Phylogenetic and other genetic analyses

**Figure 4:**
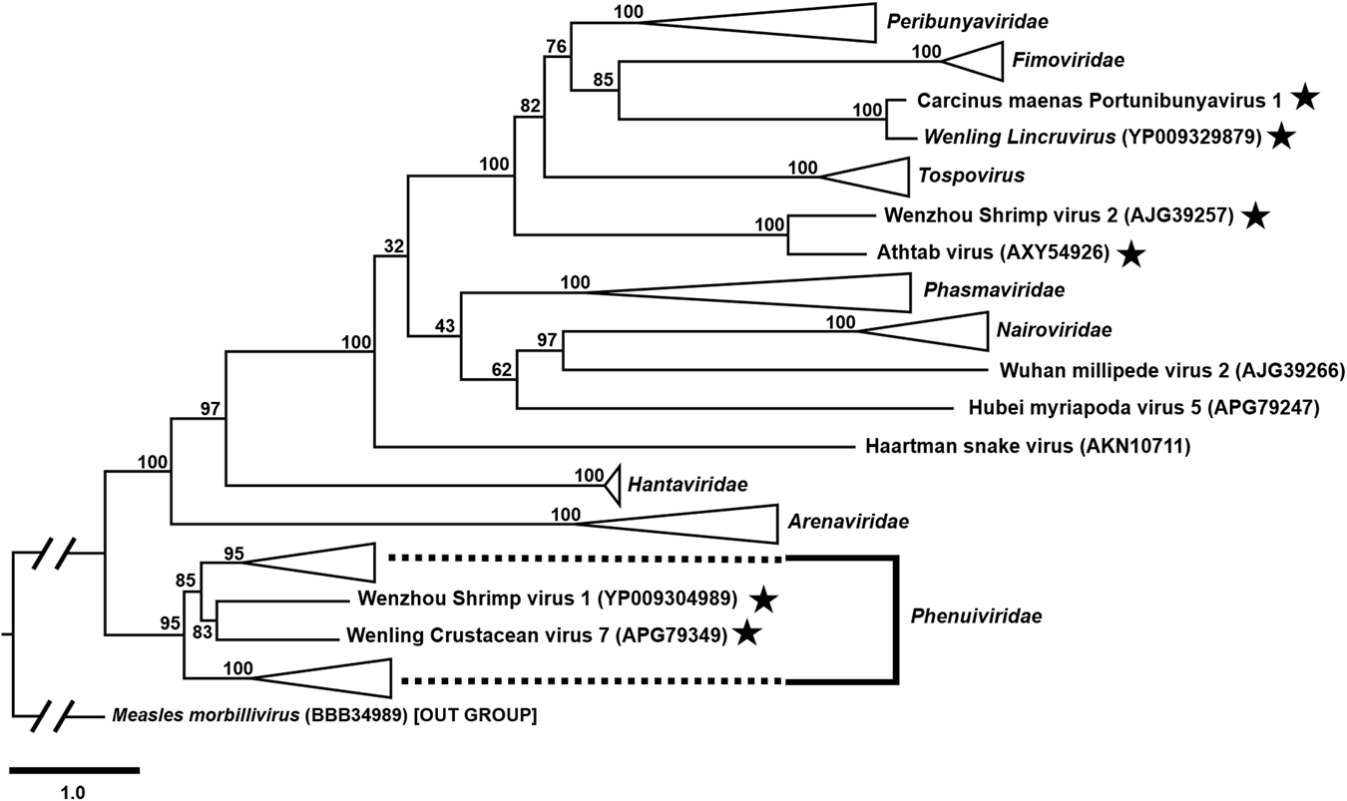
A phylogenetic tree developed from the predicted RdRp proteins of 97 bunyaviruses and an outgroup (*Mononegavirales*: *Measles morbillivirus*). The protein sequence data were aligned in Geneious using MAFFT default protocol. The tree was developed using IQ-tree. The outgroup or corresponding viral family are highlighted on the tree and the crustacean-infecting bunyaviruses are identified with a star. The FASTA file used to create the tree is available in the supplementary information (Suppl. File 1).

**Figure 5:**
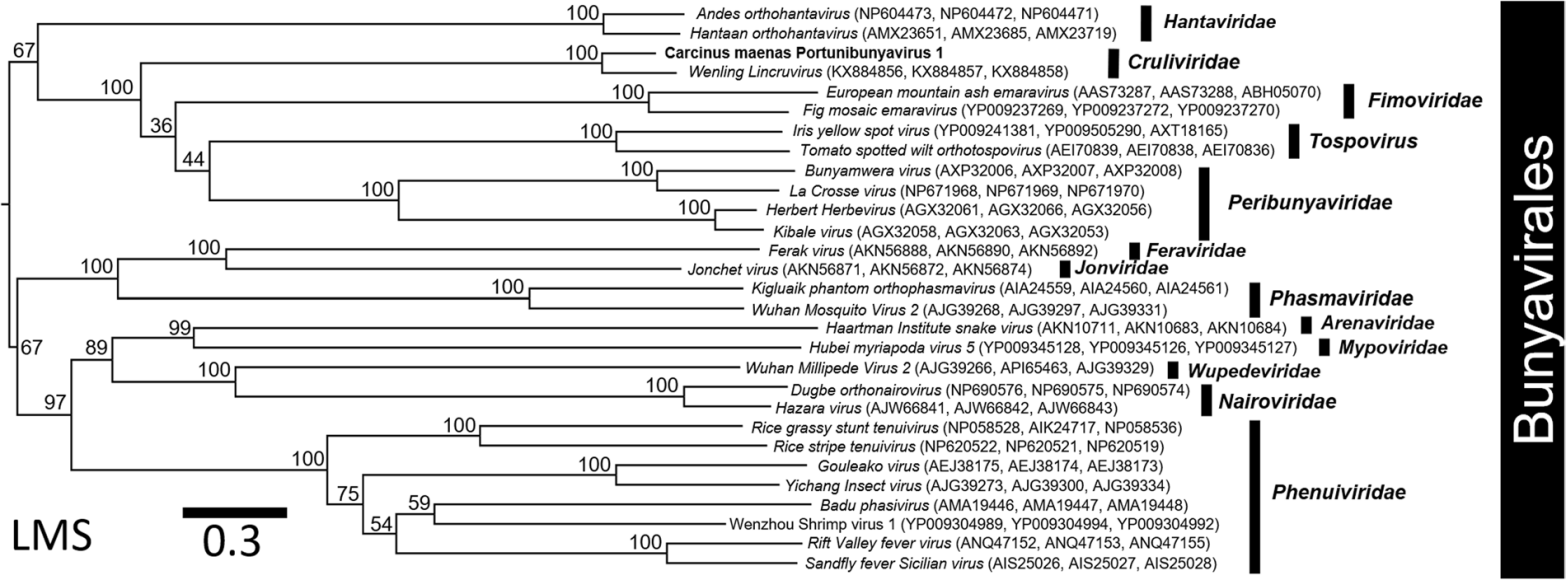
A concatenated phylogenetic tree developed from the L, M and S segment (LMS) proteins of 29 bunyaviruses. The protein sequence data were aligned separately in Geneious using MAFFT default protocol and then merged. The tree was developed from the merged data using IQ-tree. The corresponding viral family within the *Bunyavirales* is highlighted on the tree, including the *Cruliviridae* and ‘Carcinus maenas Portunibunyavirus 1’ in bold. The accession numbers for the L, M and S proteins, according to the NCBI repository, are presented after each viral isolate used in the tree.

The genomic identity of CmPBV1 to other bunyaviruses placed it within a clade containing other crustacean-infecting viruses and apparently, within the *Cruliviridae* using RdRp-based (Fig. 4), and multi-gene phylogenies (LM, LMS) (Suppl. Figure 1; Fig. 5). The best fitting model for the RdRp alignment was LG + F + I + G4 in IQ-tree. The best fitting model for the LM and LMS concatenated alignments was VT + F + I + G4 in IQ-tree.

The RdRp-based phylogeny identified three genetically distinct lineages of crustacean-infecting bunyaviruses. One lineage containing the Athtab virus and Wenzhou shrimp virus 2 branched adjacent to the *Tospovirus* group. A lineage including CmPBV1 and *Wenling Lincruvirus* branched with the *Fimoviridae* but showed highest identity to the Peribunyaviridae (Fig. 4). Finally, the Wenzhou shrimp virus 1 and Wenling crustacean virus 7 both grouped within the *Phenuiviridae* (Fig. 4). Concatenated phylogenies (LM), allowed for comparison of five crustacean-infecting bunyaviruses, and proposed that the Athtab and Wenzhou shrimp virus 2 branched with the *Tospovirus* group with low bootstrap confidence (58%) and, that CmPBV1 and *Wenling Lincruvirus* branched adjacent to the *Fimoviridae* and *Peribunyaviridae* with low bootstrap confidence (54%). The Wenzhou shrimp virus 1 groups within the *Phenuiviridae* with high support for the species representing this family (100%) (Suppl. Figure 1). The Athtab virus and Wenzhou shrimp virus 2, and CmPBV1 and *Wenling Lincruvirus*, each grouped together with 100% bootstrap confidence (Suppl. Figure 1).

The concatenated LMS phylogeny was conducted using data for CmPBV1, *Wenling Lincruvirus*, and Wenzhou shrimp virus 1, because the other crustacean-infecting viruses lack complete genomic sequence data. This phylogram altered the position of the CmPBV1-*Wenling Lincruvirus* group as basal to the *Fimoviridae*, *Tospovirus* and *Peribunyaviridae*, with 100% bootstrap confidence (Fig. 5). The Wenzhou shrimp virus 1 remained within the *Phenuiviridae*, supported by 100% bootstrap confidence (Fig. 5).

**Figure 6:**
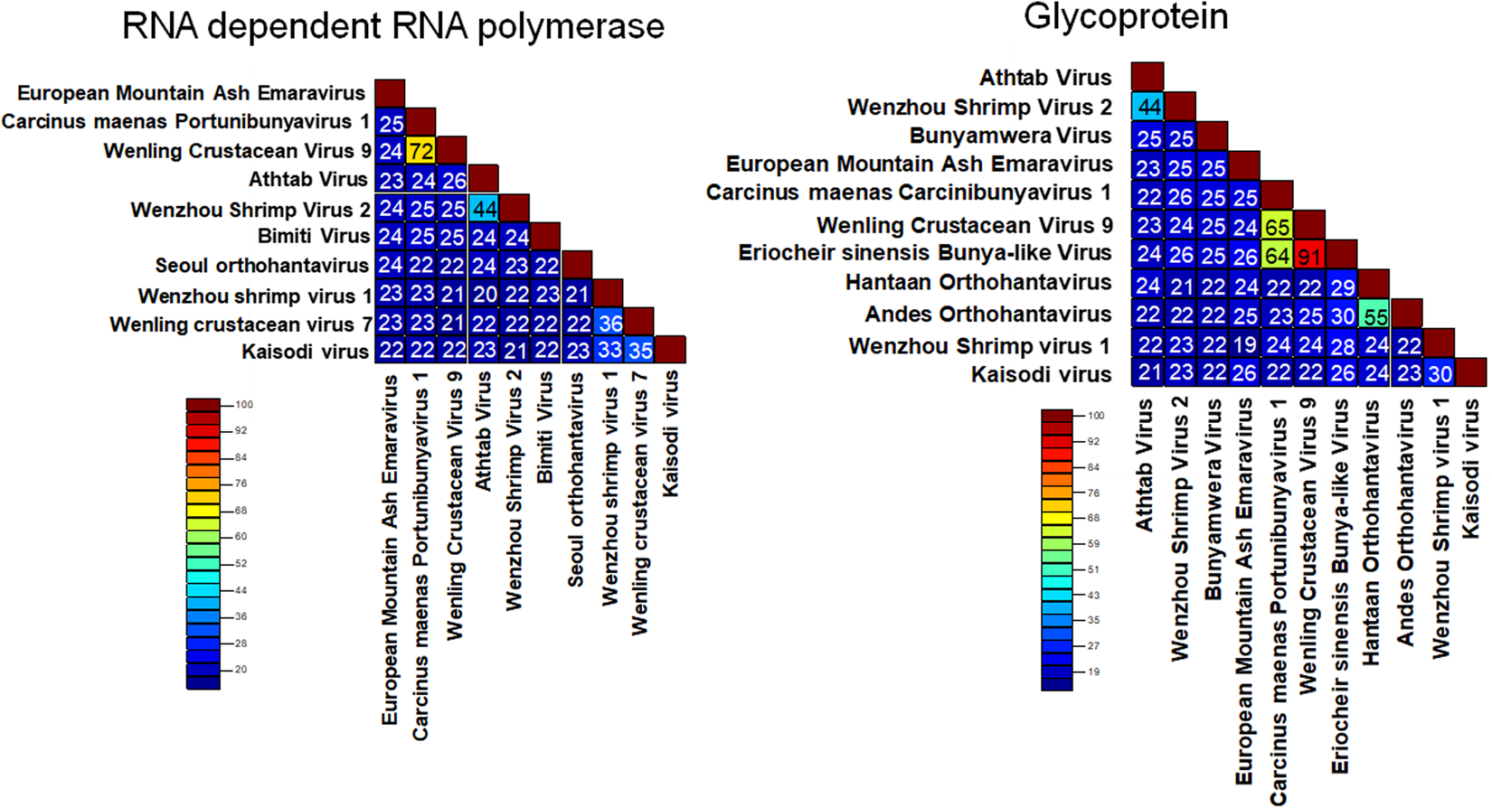
Protein distance matrix for the crustacean-infecting bunyaviruses and representatives from the closest relatable *Bunyavirales* families.

**Figure 7:**
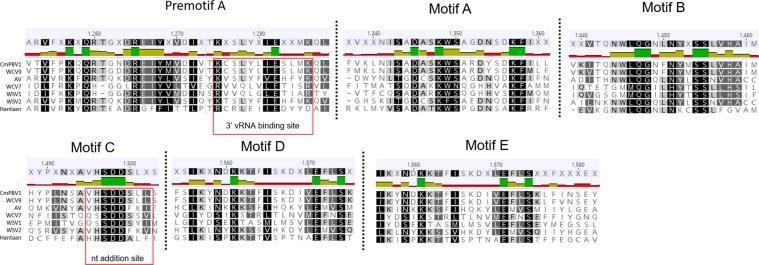
RNA-dependent-RNA-polymerase conserved domain conservation and amino acid sequence similarity using comparative methods explored by Amroun *et al*.^19^. Conservation of all amino acids (or amino acids with similar properties) across the sequences are highlighted in black. Partial amino acid conservation across most sequences is represented in grey. Carcinus maenas Portunibunyavirus 1 (CmPBV1), Wenling Crustacean Virus 9 (WCV9), Athtab virus (AV), Wenling Crustacean Virus 7 (WCV7), Wenzhou Shrimp Virus 1 (WSV1), Wenzhou Shrimp Virus 2 (WSV2), and Hantaan virus (Hantaan) are each compared. Data and images acquired from Geneious MAFFT alignment.

The RdRp and glycoprotein sequences of several of the crustacean-infecting bunyaviruses showed variable similarity among other members of bunyavirus families, *Peribunyaviridae*, *Phenuiviridae*, *Fimoviridae* and *Hantaviridae* (Fig. 6). Highest similarity was observed between CmPBV1 and the Wenling Crustacean virus 9 RdRp, with ~72% pairwise identity. Similarly, the Athtab virus and Wenzhou Shrimp virus 2 showed ~44% RdRp identity. The Wenling crustacean virus 7, Wenzhou shrimp virus 1 and Kaisodi virus (from *Haemaphysalis spinigera*) all showed 33–36% RdRp similarity (Fig. 6). The RdRp protein of *Bunyavirales* families include conserved domains or motifs^19^. The RdRp of both CmPBV1 and the *Wenling Lincruvirus* showed high levels of RdRp domain conservation across various motifs, supporting their relatedness within the same bunyaviral family, identifying 95–100% conservation in all motif regions defined by Amroun *et al*.^19^ (Fig. 7). Such similarity between domains is not observed for the remaining crustacean-infecting viruses (Fig. 7), suggesting their taxonomy lies in a separate bunyaviral family yet to be determined.

Glycoprotein sequence comparisons included that derived from a bunya-like virus detected within the crab *E*. *sinensis*, which showed high similarity to the *Wenling Lincruvirus* (~92% pairwise similarity) and, to CmPBV1 (~65% pairwise similarity) (Fig. 6), suggesting it may group within the *Cruliviridae* if full genome data could be acquired. The Athtab virus and Wenzhou Shrimp virus 2 showed ~44% pairwise identity. Finally, inclusion of the Wenzhou shrimp virus 1 glycoprotein precursor (a crustacean virus showing highest identity to the *Peribunyaviridae* and is outside the *Cruliviridae*) showed highest identity to the Kaisodi virus; however, the Wenling crustacean virus 7 (also closely associated with *Peribunyaviridae*) does not yet have a sequenced glycoprotein for comparison.

## Discussion

This study explored the genomic arrangement, phylogeny and putative development of a novel member of the *Bunyavirales* (family *Cruliviridae*), originally reported during a histological screen of the shore crab *Carcinus maenas*^18^. We suggest a novel genus (Portunibunyavirus) and species (Carcinus maenas Portunibunyavirus 1, CmPBV1) for this virus, which may represent the bunya-like virus originally identified by Bang^8^.

Our analyses support three distinct clades of crustacean-infecting bunyaviruses. These include the ‘CmPBV1-*Wenling Lincruvirus*’ group (*Cruliviridae*), the ‘Athtab-Wenzhou shrimp virus 2’ group (higher taxonomy N/A), and the ‘Wenling crustacean virus 7-Wenzhou shrimp virus 1’ group within the *Phenuiviridae* (Figs 3, 4 and 5). The *Phenuiviridae* includes viruses from invertebrate vectors, such as mosquitos and ticks associated with humans and mammals^20–22^. The presence of crustacean viruses in this group may reflect similar vectoring of a marine diseases, such as some incidental observations of shared viruses by parasitic isopods and their hosts^23^. Further genomic characterisation of crustacean bunyaviruses within the *Phenuiviridae* will doubtless provide greater insight into the evolution of this viral lineage, and the potential for marine invertebrates to act as vectors for their transmission.

Using the genome of CmPBV1, the *Cruliviridae* can now be better defined systematically within the *Bunyavirales*. The ‘Athtab-Wenzhou shrimp virus 2’ grouping also forms a clear branch within the order and requires formal taxonomic classification and nomenclature. Viruses reported upon in historic literature should be considered candidates for further work, including the study of other bunyavirus-like infections from *Carcinus maenas* (“Crab Haemocytic Virus”, “Y-organ virus”, “Roscoff virus”^8–11^); *Carcinus mediterraneus* (“S-virus” and “Y-organ virus”^10–12^); *Macropipus* (=*Necora) depurator* (“S-virus”^12^); *Cancer pagurus* (“Cancer pagurus Systemic Bunya-like Virus”^13^); the bunya-like virus from the Chinese mitten crab, *Eriocheir sinensis* (NCBI accession: KM405247); and MoV in penaeids (NCBI accession: AAY15205). The amino acid sequence identity is 91% similar between the glycoprotein precursor of the *E*. *sinensis* bunya-like virus and *Wenling Lincruvirus*, supporting its inclusion in the *Cruliviridae*. The partial sequence data for MoV suggests it is a strain of the Wenzhou shrimp virus 1 (sim. = 96.1%, cov. = 100%, e-value = 3e-^121^) and another potential crustacean-infecting member of the *Phenuiviridae*.

CmPBV1 was present at an apparent prevalence of 1.1% (2/181) in the intertidal area of coastal Nesvík, Faroe Islands, which was the only location that clinical signs of infection were detected in a multi-country (UK, Faroe Islands and Canada) study^18^. Infection resulted in eosinophilic inclusions within gill epithelia, connective tissues, amebocytes and haemocytes, caused by the development of viral paracrystalline arrays (Fig. 1E)^18^. Virions (96.6 ± 12.2 nm) were detected in the space between infected cells; and apparently exited the cell to enter the intercellular space via a putative developmental cycle similar to other bunyaviruses (Figs 1, 2).

Bunyaviruses isolated from crustaceans are present across the *Bunyavirales*, as relatives to the *Peribunyaviridae*, *Fimoviridae* and *Tospovirus* or, in the case of Wenling crustacean virus 7 and Wenzhou crustacean virus 1, are present within the family *Phenuiviridae* (Figs 4, 5). Our phylogenetic analyses suggest that the evolution of the *Bunyavirales* includes the early branching *Phenuiviridae* family, which contains two crustacean-infecting viruses. Outside this family, the *Cruliviridae* and ‘Athtab-Wenzhou shrimp virus 2’ group are under-supported with low bootstrap values supporting their split from the *Nairoviridae* and other families (Fig. 4). Concatenated data using L and M proteins support the distinction of the two crustacean-infecting groups (100% bootstrap support), one branching alongside the *Tospovirus* (Athtab-Wenzhou group) and the *Cruliviridae* as an early branching group before the *Fimoviridae* and *Peribunyaviridae* (Suppl. Figure 1). These three distinct viral lineages that infect crustacean hosts from across the *Bunyavirales* may indicate that crustaceans (among other invertebrates) are some of the earliest hosts for bunyaviruses, further indicating that marine settings may be a potential source of yet-undiscovered bunyavirus diversity, which will greatly benefit further taxonomic understanding of this order and its origin(s).

From the perspective of the host, *Carcinus maenas* (a globally invasive species) has negatively impacted biodiversity and aquaculture/fisheries on shoreline habitats and has been identified as a carrier of pathogenic species, from viruses to parasitic Metazoa^18,24^. This host has been found to introduce parasites and pathogens to multiple locations and the biocontrol of *C*. *maenas* via parasites has been considered^18,25,26^. CmPBV represents a pathogen that could co-invade with *C*. *maenas* and interact positively or negatively with local fauna in novel settings. Bunyaviruses can cause mortality in humans, animals and plants but little is known about those infecting Crustacea, meaning that an attempt to predict a co-invasion effect is difficult. In Australia, the native red clawed crayfish *Cherax quadricarinatus* (a fecund and wide-spread freshwater invader elsewhere) has a bunyavirus that causes host mortality when viral copies exceed 10^6^, resulting in 20–40% population mortality over 3-weeks^14^. Just as CmPBV1 was identified from *C*. *maenas* native to the Faroe Islands, the presence of two crustacean bunyaviruses restricted to their hosts native range suggest they may be commonly left behind during an invasion, likely by dynamics surrounding the enemy release hypothesis^27^. Experimental data on the host range and pathological effects of CmPBV1 are now required to further understand how it may affect other natural populations and communities, as well as fisheries and aquaculture industries.

In conclusion, CmPBV1 is likely a re-discovered virus based on the initial observations by Bang^8^ (despite a different geographic location) and now has corresponding genomic, ultrastructural and pathological information, which has provided insight into its systematics. Confirmation of this re-discovery requires the application of diagnostics to the original material, or recollection of specimens from the same population used by Bang^8^. The identification of a bunyavirus genome from *C*. *maenas* has provided additional taxonomic clarification to the crustacean-infecting bunyaviruses and has revealed that crustacean-infecting bunyaviruses are present across the *Bunyavirales*. Whether this virus poses certain threats to native fauna across the globe, or could be used to control *C*. *maenas*, requires exploration. Invaded areas with high densities of *C*. *maenas* provide interesting study populations to determine whether the virus is present outside the host’s native range and whether it may be applicable to manage the invasive species in non-native habitats.

## Methods

*Carcinus maenas* (n = 181) were collected by hand from the shoreline at Nesvík, Faroe Islands (62.216° N, 7.016° W) in August 2014. Dissection of samples for histology, electron microscopy and molecular diagnostics has previously been detailed by Bojko *et al*.^18^. During the study, Bojko *et al*.^18^ recorded histopathology consistent with virus infection in the gills of two crabs. Viral aetiology was confirmed using transmission electron microscopy (TEM); however, no genomic data associated with this virus was generated. Bojko *et al*.^18^ reported the finding as ‘iridovirus-like’ due to the morphological characteristics of virions and their presence within the cytoplasm of infected cells within paracrystalline array. Samples collected at the time, fixed in 99% EtOH and stored at −20 °C, were utilised in the current study as well as TEM grids from the Cefas Registry of Aquatic Pathology.

RNA (86 ng/µl) was extracted from EtOH-fixed gill tissue using a Zymo RNA extraction kit (Quick RNA miniprep kit). A cDNA library was generated using a NEBNext Ultra II RNA Library Prep Kit for Illumina and sequencing was performed using a V3 600 cycle kit on a MiSeq sequencer (Illumina). Raw data were processed to remove host reads by first running KRAKEN v.2^28^ using the *C*. *maenas* transcriptome (accession: GBXE00000000.1)^29^. *De novo* assembly of the remaining paired-end reads was performed in SPAdes v3.5.0 (default parameters) and provided 73519 contigs (min. 500 bp, max. 6813 bp) (quast.bioinf.spbau.ru: N50: 981, N75: 562, L50: 8, L75: 23)^30^. BLASTX analysis of the resulting contigs was performed in Blast2GO against the National Centre for Biotechnology Information (NCBI) GenBank non-redundant (nr) protein database. Protein annotation was completed using ExPASy in addition to protein domain assessment^31,32^. The integrity of the genome sequence was verified by mapping raw reads using Bowtie 2^33^ and inspecting the alignment in Tablet v1.17.08.17^34^. The viral genome was annotated using CLC genomics workbench and the functions were predicted based on BLASTP searches against the NCBI GenBank nr protein sequence database, ExPASy and VIRALpro^35^. The annotated genome is available under Bioproject SUB5372281, Biosample SUB5372296 and accession numbers L (MK861116), M (MK861117), S (MK861118) through NCBI.

Maximum Likelihood phylogenetic analysis was performed on the full-length AA sequence of the L protein (RdRp) for 100 representative bunyaviruses including 6 available crustacean bunyaviruses and a single outgroup (Suppl. File 1). The AA sequence alignment was performed in MAFFT v5.8 using default parameters^36^ and the phylogenetic tree was constructed using IQ-Tree^37^ with Bayesian information criterion to determine the best model fit and 1000 non-parametric bootstraps to test the robustness of the clades.

Additionally, two concatenated trees were developed for bunyaviruses with available complete genome sequences or those with available L and M protein sequence data. One tree utilised the RdRp and (pro)glycoprotein sequence from the L and M segments respectively to produce a tree consisting of 31 viral taxa across 13 bunyavirus families. The second tree utilised RdRp, (pro)glycoprotein and nucleocapsid protein from the L, M and S segments respectively to produce a tree consisting of 29 viral taxa. For the concatenated trees, the AA sequence alignments were performed for each gene via MAFFT v5.8 using default parameters^36^ and concatenated using Geneious^38^. The phylogenetic trees were constructed using IQ-Tree with above-mentioned parameters^37^. Each phylogram was annotated using FigTree^39^.

For genetic analyses, two protein distance matrices were constructed using the RdRp [10 bunyaviruses (6 crustacean-infecting viruses)] and the glycoprotein [11 representatives from closely related families (6 crustacean-infecting viruses)] using the Sequence Demarcation tool v1.2^40^.

## Supplementary information

Supplementary Dataset 1

Supplementary Figure 1

## Data Availability

Sequence data from this study are available through NCBI as stated herein. Biological materials from the host are available from the Cefas Aquatic Registry and Repository.
