# Supplementary material for: Genomic and developmental characterisation of a novel bunyavirus infecting the crustacean Carcinus maenas
Source: Sci Rep. 2019 Sep 10;9:12957. doi: 10.1038/s41598-019-49260-4 (PMC6736955; doi:10.1038/s41598-019-49260-4)
Supplement: Supplementary file 1 — Supplementary Dataset 1 [file 41598_2019_49260_MOESM1_ESM.docx]

**Genomic and developmental characterisation of a novel bunyavirus infecting the crustacean *Carcinus maenas***

Jamie Bojko^1,2*^, Kuttichantran Subramaniam^3^, Thomas B. Waltzek^3^, Grant D. Stentiford^4,5^, Donald C. Behringer^1,2*^

Supplementary dataset 1: The RNA-Dependent-RNA-Polymerase sequences for every viral species compared in Figure 4 within the main manuscript.

>Carcinus_maenas_Portunibunyavirus_1

MDWVRPHRLKVRQLIALYTNSTSAAEAGGIVKDLRIARHDGFISYLNGQIPDIDFGVDSTGDIPAEYIHKFVTDSLNVDVDEDLVIPSKTPDGFMLLDNCLYIIDVKTVTNMELQDETVGKYREVFQIITNRYGINLSVNTINFHPYQHTISVYPNPDILPVIHADDDLINYSDEITTNIQRIQARWQGNQTFQAAQHDAAVNFKGSFTDVGEWEAKCITHPEWMSVITGMGRGTAKLMKHCITNRLTQPERDELTNYYERSTKSKGYLMKAKLMDGADLDVVMTQGTPEEVTTAIREMHENTAPRKTFVHQSKPSCFYYSTKSHYGLPDGNYARIHKFASMAKECSSRHPVIEMLKNIKIDNPDIYDGEGLPENQDGFPFRAKQILKLQNTEIKNRLYQNAKVGMNYVKRCNRDDRTYNDDKPECLDYTDAEVVNQHSNLINEQFSKLTQESYRINDVSHIYLDDIKMEQKNAEAVKFIKWMRETQAFSWLSDQEDKLPNMLAISGLLKNAEVGLILGKSNTSMHIVYPGKSLTTQEANLCYNSIHIVTDSYSPVDVGLIDAYIKIPRSMYALVITKPVRLDTTRLGVLTCSHTRFQMVALHYFLTSLETHKDFESVPAGLLRNIIGVSFFLVQQCTLPFTNLVDKMKYIVQDILADMSNVSGYISEKLDITPKNSFTLFLYYHFMSQIKYLRQHFKKGKNQPIVIEADDVVEAGIEGLTIPSIVIPGLKLPNVQAIVQDYVLAWYVTAKGLHNPVHNLIKLYKVPLEFQEELNMNNGMRPDVDKRLMLASFLKYWDTRVSTSIEARNRCFTRMKIDKSWTQIPTMTSTKSVVHKLNKNELRRNKITEQDLLDMDKDEFIKRHPGLKIVNGRVLNRHTGQIPLMLRDQSHWSQVRSGRVFDEMERLNVGQNADLNIRNAKSIVTITDHQKVLKPCVTVFPKQQRTGNDREIYMVDIVTKCSLYLIESLMKQLCVENQRECISLAGDRKTQKIKSLLIQSMRNQQEFDDKSNQELQFVKLNISADASKWSARDYSDKFILLIASLPCLLPEEKWKMIFLLMNYRSKHVVLKDSILESIMDKNLTNDSVFWRMTEGFKYNSVKITQNWLQGNFNYMSSLVHAIMHETIDDSLIHFNNAMAAHNGGVPTTHYPLNSAVHSDDSLLSFCFEVPQDVTMTRLANCIFDLMVSTGGCFSIKYNDKKTFISKDIVEFLSKLFINSEYHTNYMRSLLPIFIELPFGNITQDMAAGLGPLTQATAEGAPPSLVYLAQCLLPYRVANLNAMLPGMVNDSRQYFQNEFNALPTCLTGSIGDDITDSCILGGAAYDYARVFHILSKNSPPHIEIPKMIEGFNSDRLSPPDLEFMKILSVSLEGHRTADITHVGIYGVKDRFFNTALVPMNISRVKETSKYLSSARYNFLGKDGELRPVIDQLKARHPMWSISKPVRPEDYPDYNLYKMTELRFRQSLFLPHSSHGFLLKILNRRTKVVPKKALELHDPFLPVIEENDEVEMITLNEALAYLKSKIDAIELTSDQVKAIMSTMLTSNQQVIDYLTTRNASKLTSEVMKKSRFLVQEPSGFRQKVVQNPPSLVIKRYFFPDEFEMDGDVLYLDEAVQSDLDTISSVLRAYNLIDLSRPTHPDFTRNITKILSLVFNHITNKMGSNRIYILPSRLQSAPRFIWTLMGSRMNDYKHFIYTSVVPNIHLYLKGNKQRHDVQMQRVRLQELFGVLWILDHITSGAGDQSGPLSILINNLTIEGVPARDVLDRGLTDKYTRSTCAAIMYWSGHLNFSSFKRQSIPSIVQKEWLVAQRTADVGEFYVIYELKGVALIVKGDTRIVTTIRFEIPDGQELAGIEGPLLTAFYKDFSRIIANMNLMTSNTRSNSRSYSLRHKSLRPDPQGEWLIGIRRGFGTRITTIQEEEFLDAVEACTWVSFPIPYRSQDMGLKTVKPYRPFLDHMNISSSTPLFINRTDLVKLINIDSFRDAVVSRGETYNYGPWLNCLTYRGVGFQIKVQPDDDFFIPAPSIPVGVRAMPKVSIVETYNKTWHWYHKMYKIYKGLYDVFMTGPAGNHEPRGERIMTYLLQIQEYMWYNTDKVGFTLFLSEWVGALSVLNPDHVSAEVFDGDQQTATNKANYIIGRLEALKSKGMHEFSIDVSIVKLYRDQHIGDVGVEW

>Melon_chlorotic_spot_virus_(AYL40766)

MNSILISTLGLTCKLTNLPNIKRNGRLVQVPGDGWCLLHSLAKSKDSSPEHLFIEMMDYLRETIRDNPVN

GEDLKKMLHAYLDKNVWFDDTLIILFSCCFRTTVQVFSEQGLMKYEPETYKTVVDLRYDAPNLKNGHYSI

YEDCTGILKSNLWSDIVLEVEWAGMITDALPFKGETLDTVLRTIPSPYYNDIKTTIMEQSFQNDNFKRIV

AHGINIATLLRTNFYINLSVRSSMSLFLLKNNESEYLMPVSTLVWVEEDINQEEKLTLEETHLYINPKTK

SLDCSNDYLSEPYNTFKECYAGLISWIFEDQLSEDILDLLFVLIPNSYESIGPLNEILGIKVIFHFGHHD

DNTAYWYNKSELVEYHLVLLKDHCKFLKKWNYVTKADKRQKDLLLLKRNDEYIPTTFPTFMEKVTGFRGS

LYRKAIDLRIQKYSISILKLLTLTITRNVFELVFLLRLLSLNSTKTSVIRVDAALDSTRSSDVSLRVAIK

KVGFSHTHNLLVIDSNSKCYMCSRREQANVHYLPTEKQKHAKKIVKNIIGMEVFPSIPNYAPNLVQYTPE

PKSFQFPEPEVYPTAVMKDIPIFLSLHIEIKNRGKIHVSFENSGSTISTYNNNSYILDRSKARTFIHDFT

FDLIAPETDMAFGSAGLSLRDPDDYKTPDLIVRTEDDKFYIYEFATRRSSALITLEKAQVSKDLKYRGMI

EQRSRILQKDIFYGIITATPQALQTNVIGLPKELCEELMFRMRTATEIFNKFVEMEIEGCADAESSMRQV

RIESIFKTISMENMTFVAPFSREMYEGFAKPIDKDDMENHTKLVLDCFKLSKEKIISEVNTGSTQNVETF

NDISSIRFQESLQKFYDNYRTEYPYGATRTDMKPPVQLPFIYTYNNLPQPTINNIMDLVNIFNCDSSNPH

TRLISSCVLKIKNDDINETNEFDLILMDKEERAKAEKLAKKNRKEYRRVEYQLSETDEMDLATIGLFGRK

YKDMVKDQRSYKKLPFDLHETVVSDIHELLNVQAVELFSRDEEMFFKEDIDKLLKDSLGLHNTNSIQKYL

QTYDYLKQTRVAHWARFISDLGSELSISVKQFCKSGNAIFKKLKNYSTYLLIIPTGGPIFFSLLFPKVDV

CYSNHSTVFKNLNETEGYYYTDFVSVNSSKLRNLVLLESVLYSTFMLFAELSKLPTFEEMDTNTNNFKIA

SGLTMLSLLICLDDKAKTEEACTLNRYVQMEGFVHFPKVMDQQKLLGRVNLNIRSRLELFVYESIIECMK

RYSSNPCRLLVQENKRQYEGMLIPYLRDESGSQRKCDSPEMMLNIIYIGYIKNKDENAEVNALGQLISKI

IGWEDSVPSTAEFLGLKDPKPESIQKHEFNTTAIRVLTYKAKERLKKTLGATNPTEYIGKLILDYLKKQS

LEQFSTLKASSNFGEKYYYYVPKSKSERTSHKAKMESLGIEESDPGYKRSKVIVKLKEWLDSKEKPENQT

TLVVDLLLDALEQVEENMCLHVCIFKKNQHAGLREIYVLNIAERIIQKTVEDISRAILSCFPSETMTHPN

NKYRIPEEQAKDARNNLGSKYVTYNTSDDAKKWNQGHYVTKFICMLVQFTPTYYHGFICRTLQLWLDKRI

MLPVELISLFSNLDEMHTKDEFVSRLFKAYKGYTTEKWISKGQSYIRTSSGFMQGILHYTSSLFHTIILD

FISETAQMHLMNQIKKIAPEMPSRVVVRNMQSSDDSSMMISVPVFDDDKKNNSIIAFCCYIWFTFKELYS

LQLGIYKSEKSTTMTQFIMEFNSEFQFLGEIQKPTIRWVYACCLISEQETLVARQEELSNTLKDVVEGGG

SFILALYCQIAQLILHYRLLGSGVSMFWDIYTNLIRKVRDPALGYFLMDNNICAGILGFNYNLWNSYDST

VLAVRYKQQSICEARAPIRETIKQITPDTLSLSLFTRLTTIRYGNKKIWERIMNNMNFPESIWDDMEKDP

AVLYYGANDVDEVIKKMAIKMKAPGVVASLGSANSLVRIISTGVYIMSRSVVSTQSAMFDNDGNRVLNKA

PLLQLLIDENEKTMSYVRSVDVLRPDPEVPETVVFAKDTLVQITDTPEEYLMDCRPDLSENDIEVSRPLY

QPFIKKNLLPDDKGSWIHFLFTVFNIPHQMYRKKFESENTFGLSGISDLSTTRWDDRSVKRIFYKDDDLY

YQVVTSNYQWGHNDTQLFTLGNELRKLLFPFEVDYQAIRHNMDMIDGTPMNYDPGHRRRTRSNLVIAGSE

SSDLFSLEEIAKYVWFRKTKPPLSDSVIMNLWANFKVKYPFLRDTVEETLSSEDCPFPNHISLRNFIARQ

DKGSRVIHLTGSHGKHGTQHANLLTVIKNNTSDNYSYDQPIIDENKRTQHLNYETLMHTLSMLSEMPLDD

EVRNFLMEDLLVRSPSVSTNIYALGGRRNKMSIIQSIIQEDPEMILSSKQSVDDVYAYYRQSLENRWLSY

TEDDVDSWLDMIYVKTNGETIPVPIYIEGVPEDILKAYNLEPTFNISKLKGNYVQANWDQSLIDFLIYKL

DRNIIRLMARGTVLSLRDATRVIGEFEIKRQRIVQNISLLKLGMIGGYTTRQTRSLEGIYQGIGIWEGHF

NNVGIKIEINSDGENSFFTSICVRKAPNDVKLFLSMLKTWSQDHKVGYSKDKLRPSNVGTILMYVYEGKI

VKSYDASSIPIMLNEHMEVIYNLSVSDFRVELTNHNLRLMAKVQNSDVREITTMQVKFTGRDINPNIRDV

APVDFYQIIQNWSPIISTWVKFDSLDYEWIDYFSRSEMRPMRLFPTEARLNWCTNLVRNSIYRLHLDRQA

VVLTNETKEEEKDGLNIAITGMLSLSNTIETVLRDMPEFNITDVDFEEIIELLETEDIQDRNLASSSKLF

IHPFLDNILLFFVKRYTSSTLRTLIRNCSDPGYKLLSTYSMVRPILEAYSGRIIDLEEPIVKKSDLDVMP

SLR

>Rice_stripe_tenuivirus_(ASA47670)

MSTSPLVIPLHVHGRSYELLAGYHEVDWQEIEELEETDVRGDGFCLYHSILYSLGLSKENSRTTEFMIKL

RSNPAICQLDQEMQLSLMKQLDPNDSSAWGEDIAIGFIAIILRIKIIAYQTVDGKLFKTIYGAEFESTIR

IRNYGNYHFKSLETDFDHKVKLRSKIEEFLRMPVEDCESISLWHASVYKPIISDSLSGHKSFSNVDELIG

SIISSMYKIMDNGDQCFLWSAMRMIARPSEKLYALAVFLGFNLKFYHVRKRAEKLTAKLESDHTNLGVKL

IEVYEVSEPTRSTWVLKPGGSRITETRNFVIEEIIDNRRSLESLFVSSSDYPAELCSQKLSAIKDGIALM

FGFINRTPENSGRELYINTYYLKRILQVDRNVIRDSLRSQPAVGMIQIIRLPTAFGTYNPEVGTLLLAQT

GLVYRLGTTTRVQMEIRRSPSVISRSHKITSFPETQKHNNNLYDYAPRTQETFYHPNAEIYEAVDVKTPS

VITEIVDNHIVIKLNTDDKGWSVSDSIKQDFVYRKRLMDAKNIVHDFVFDILSTETDKSFKGADLSIGGI

SDNWTPDVIISRESDPQYEDIVVYEFTTRSTESIESLLRSVEVKSLRYKEAIQERAITLKKRISYYTICV

SLDAVATNLLSLPADVCRELIIRLRVANQVKIQLADNDINLDSATLLAPDIYRIKEMFRESFPNNKFIHP

ITKEMYEHFVNPMISGEKDYVTNLKSIIDKETRDEQRKNLESLKVVDGKKYTERKADTALSEMSQAEEHY

RSYFENDNFRSTLKAPVRLPLIIRDVSSQDNQFSNKELSDRIRKKPIDHPIYNIWDQAVNKRNCSIALGH

LDELEISMLEGQVAKKVEESYKKDRSQYNRTTLLTNMKEDIYLAERGINAKKRLEEPDVKFYRDQSKRPF

HPFVSETRDIEQFTQKECLELNEESGHCSLINVEDLVLSALELHEVGDLEHLWNNIKAHSKTKFALYAKF

ISDLATELAISLSQNCKEDTYVVKKLRDFSCYVLIKPVNLKSNVFFSLYIPSNIYKSHNTTFKTLIGSPE

SGYMTDFVSANVSKLVNWVRCEAMMLAQRGFWREFYAVAPSIEEQDGMTEPDSVCQMMSWTLLILLNDKH

QLEEMITVSRFVHMEGFVTFPAWPRPYKMFDKLSVTPRSRLECLVIKRLIMLMKHYSENPIKFMIEDEKK

KWFGFKNMFLLDCNGKLSDLSDQDQMLNLFYLGYLKNKDEEVEDNGMGQLLTKILGFESAMPKTRDFLGM

KDPEYGTIKKHEFSISYVKDLCDKFLDRLKKTHGIKDPITYLGDKIAKFLSTQFIETMASLKASSNFSED

YYLYTPSRRLKNQEQSRSKHVFDAGGNISASVKGKLYHRSKVIEKLTTLIKDETPGKELKIVVDLLPKAM

EALNKNECMHICIFKKNQHGGLREIYVLNIFERIMQKTVEDFSRAILECCPSETMTSPKNKFRIPELHNM

EARKTLKNEYMTISTSDDASKWNQGHYVSKFMCMLLRLTPTYYHGFLVQALQLWHHKKIFLGDQLLQLFN

QNSMLNTMDTTLMKVFQAYKGEIQVPWMKAGRSYIETETGMMQGILHYTSSLFHAIFLDQLAEECRRDIN

RAIKTIVNKENEKVSCIVNNMESSDDSSFIISIPNFKDNEAAQLYLLCVVNSWFRKKEKLGTYLGIYKSP

KSTTQTLFVMEFNSEFFFSGDVHRPTFRWVNAAVLIGEQETLSGIQEELSNTLKDVIEGGGTYALTFMVQ

VAQAMIHYRMLGSSASSVWPAYETLLKNSYDPALGFFLMDNPKCAGLLGFNYNVWIACTTTPLGEKYHEM

IQEEMKAESQSLKSVTEDTINTGLVSRTTMVGFGNKKRWMKLMTTLNLSADVYEKIEEEPRVYFFHAATA

EQIIQKIAIKMKSPGVIQSLSKGNMLARKIASSVFFISRHIVFTMSAYYDADPVTRKTSLLKELINSSKV

PQGHDYLQEPHTLKPTKVEVDEDSWEFKSAKEECIRVLKQRIRIHTGREERSISLLFENISKSMIGRCTD

HNDVRENVSIIACALKMNYSIFKKDAAPNRYLLDEKNIVYPLIGKEVSAYVKSDKVHIEISEKKERLSTK

LFNIDKMKDIEETLSLLFPSYGDYLSLKETIDQVTFQSAIHKVNERRRVRADVHLTGTEGFSKLPMYTAA

VWAWFDVKTIPAHDSIYRTIWKVYKEQYSWLSDTLKETVEKGPFKTVQGVVNFISRAGVRSRVVHLVGSF

GKNVRGSINLVTAIKDNFSNGLVFKGNIFDIKAKKTRESLDNYLSICTTLSQAPITKHDKNQILRSLFVS

GPRIQYVSSQFGSRRNRMSILQEVVADDPTLHWPDQDTSQKQLEDKFRELAHKELPFLTEKVFHDHLEKI

EQLMKENTHLGGRDVDASKTPYVLARASDIEIHCYELWREYDEDEDEAYQAYCSEVEAAMDQEKLNALIE

RYHADPKANWIQMLMNGEIETVEELNKLDKGFESHRLALVERIRVGRLGILGSYTKCQQRIEELDGEGNK

THRYTGEGIWRGSFDDSDVCIVVQDLKKTRESYLKCVVFSKVSDYKVLMGHLKTWCREHHISNDEFPTCT

QKELLSYGVTKSSVLLYKMNGMRMLRNMEKGIPLYWNPSLSTRSQTYINWLAVDITDHSLRLRNRTIENG

RVVNQTIMVVPLYKTDVQIFKTSPVDLEQDVQNDRLKLLSVTKAGELRWLQDWIMWRSSAVDDLNILNQV

RRNKAARDHFNTKPEFKKWIKELWDYALDTTLINKKVFITTQGSESQSTVSSGDSDSAVAPLTDEAVDEI

HDLLDKELEKGTLKQIIHDATIDAQLDIPAIESFLAEEMEVFKSSLAKSHPLLLNYVRYMIQEIGVTNFR

SLIDSFNQKDPLKSVSLSILDLKEVFKFVYQDINDAYFVKQEEDHKFDF

>Wenzhou_Shrimp_Virus_1_(YP009304989)

MENWLRELETSNDAVYVHNPSTIFEPTETPELLSYEIKNWDPVAMTQVEIEFDDRPDEGTGSTLATQVIS

VQRVRTFLHDFTYSHISHNTDMRLDSVFRRMGTRGDNLTPDVILQDGNKILVVEFATTRGGDAALERSFR

TKTAAYDFELRERAENRSMDHFDTQVFYGIIVTNELKVCSNLPLTEEQVNELVFRYRMAIDIQAELGVLG

VSFHEEDDEMSQTSAEVKTILRNIPLSFHFDDKYVTREVYENSFGAPDSEYLKTMISTLMSDGRKKDLAT

GAGMNFCDEDKRWEGPSITKNLNECLSSIKKYERDLMEGERQHSSTKAIVQLPAWVAKRESHPGSTSVNY

IDVGDHPSAAHAVWKNAMFHVLSRQGFKDDDEQEILMTEEENQDHHKPHRAEYHRVDLTLEDHVRSALAV

NGVYGKEFKRNERVLAQRSEKRKPLSYSVDCSCVDTFISDNSLHKTLDTPIHEDLVRIISTSTEMHSNDS

HQRECLKKFMSSILGAFSCIMTDVATELCISLKQHVKRNQMIIKKLRYYPIYMLIYPTDSSKHIFVSLLA

EKDTVSIWDSSDCFKKTMSNDKVIFTDFVSFNSSKLANLCKLEATMYNTSMFWAEEHGEASFLGGQPMPW

YSRDSGVRAEVWKMISLCLLISMSDKRHVEEQLSLYRYMNMEAMAIFPRVPQPYKMLKKMTLVPRSRLEL

WIIKRHLRYMKHVCSGYHAIKVKDDSGDSNWHNLINPYTLAPLQSISSNVNLNYIGYLCNKEETAEGNTA

ANIYEKILVLEDELPSSNEFLGLRDPVDPGYHEFSPSLLKAAVDNIKDRFRRVFGSSWEANIESKILRAL

GSITLEEMATLKASSDFSPKYYTLKPGDAYRRRKAIQGVRDLMDGESTQMYEVLSKCLTQVEQDGCMHID

IFKKPQHGGDREIYVMDINSRVVQLGLETIARTYCSFIDSEAMTHPKSKFKLPEEHERNAEKKLGTHVTF

CQSADARKWSQGHHVSKFMQMLCRLTPNYMHGFIVRACALWTKRRILISPALIKIFCETPSFTSENDIVQ

RMYNGYAGLGTEKWISQGQSFIQVGSGMMQGILHYTSSLLHSIFQEFLRSLLRSHYRSRELVIGEPMITV

GQSSDDSYIMVTFKTRDSETWAEAALEASMMHHLKSTLSKFLGIYDSEKTASMLMSVLEFLSEYMEGSSL

HRATVKSVFACLSISEHESLSSRQEEMSTLLTKVVEDGGSINLASHCQIGQALLHYRLIGSSVSPAFNLY

CSGIFTSKDPALGFFLMDHPFTAGLLGFKYNLWNACVNSSLGKKYKRMLIQPLAHRPLEATSSGTLLHSY

HITWGNRKKWERLVRSLELPEDWREMIEQDPVVLYRKARTEKDLELRLAEKVHSPGVCSSLSKGNAVTRI

ISGSVYILTRNVMSVVGDKSKISLIKAILDERSEIEPLSWAEESLLFPSALEYHSISVALRKVDSRAGVF

RKHKDRRKRSNIQITDSDGDYLFQPDVILAWRWFNIDRMHAAMRVKEKMFQKLQQALPWVKDTPEETVEQ

SPFENQIQLHTFLTKTTMKRREVHLLGTEVTQRGGVSSLLTAVYQNHFPGYHLQFLKDEVAASQSSEYSK

LAHFIHMTHLSPYPDDHKHQLIVSKLASSPQIEFKEKFGRSRRNCLAIIQKSLALGISDLLDQITFSYKM

GVVGGFVKRQKSQTIRGKVVYTGTGTWVGSMDGVNMRVTVEGTRTSNHVMSIVVSNPRAMYTQTFGKLLQ

TWMKEMGVTEGDPQVKPNVVAYYRNGHIYQGGRGGGGGVPIFHSLHGLEVFEPRLIQSVDIQVKNNTVRL

VAGYGTRHISNMTILSYTTRDKDSTLTATRADFPEIKPAMQYWYANSSWPSDMALGIINKALTGVLMPNA

DQPAVQQWLQTLFRNSAERKGLLEETLDLPIGSITSTVVPEVFDDFWDLVDFGEPENLEEFGEAVPSWED

FDFEMSFGGANISNLTPGVLRTHPLLDSLVGKLVRDLSMPAMSKLVRDKVGTPAMHPLVALFAYLMGVDQ

SEIAIPDWDLVPSEAEAWSLV

>Wenling_crustacean_virus_7_(APG79349)

MAFKDILVDSIESDILANQFICDPYSIYDSSVNPYLIDFKIDYEEVGNIVTKFRISFDCDLDDFSTGTTL

KASANKIYNVTEMSSFIHDFTIEPIEMDTDVKLSYMFPLIDDGANHLTPDSIWRTTKGHYVLEYTTSGTT

NVSSMEKTFAKKVASYSQALQNRLEKDKYIKFDVIVISPDSVITNCQYLTDSMIDELCYRYKMAVAINRQ

LVRDGITRSDVDDSETQNQKKIHDILASIRPSPSVKFMDESFLHQALKPCDRDSALKAVNDCMGKCVKKV

NDRIKNIYQWDPDNHKAVGKTIQNKVSEMEEMIEKYEESVNENPLRSLFDKKSVIQLPFWVLNEGDGCIS

ITSDNLNSESVGWSNDETFKVWLAAIQKLEMGLVSNNEEDEDLEMKIAMGEEQADPMQKGKFHRVSLEID

GSMKKELQKVGIFVDRKFQDEDIKELRRDKNNGFSLDVEVDDIDDFLENTKIWEDTETVLMKDSEILDTL

MESSKLHGSEEASDISREVVESFLCTKMGRVSSFIADLATELCASIKQHCKKDQFVLKKLRHFDVYVLIK

QTNSMKHLFFSLLAYNQDIEFSLSSPWKKFNSNGRVSWTEFVSVSQSKLVNLVMAESNIFASWCFHHHFF

SVPIWQERNDYRVCKQTNLDTLIMLHDKSQVEEIITNMRYVMMEGFVSPPNIPNPHKMIEKLPIKIKSRL

TSYIIRRCFDSIRRISNGYYFKPIDPEKPSDDMVWLNLFHPITGDRLDSPYQLINTFYIGYWKNKDETPQ

ANTIGKMFIKILELEKDRPTKENVGETSKFEHELKYHEFNADFLKLICDHAGNYLRLSIGKDWKDVLDKE

IRSKLAEADIESHSTLKATSNFGPDWYNPDVGKKYFRSKVIENLWKLADEQKTLMLELLPKSLSFLEENG

ESLFIDLFRKPQHGGLREIYVLTVEGRVVQLVLETISRVLCSKFSSETMTHPKNKVKIPENHAKVARKQF

GSNFITMATSDDAKKWNQGHHVAKFAMMLCRLTKPLYHGIIIRGLRLWLNKKIMIPLELLKIFDCNPFLQ

SYDPYLARMNDCYRGRAEEIWMKRNSRYIQTETGMMQGILHYTSSLLHSILLQYYVDICRESGKNILRAL

DVKPNFIISTQQSSDDSSVLLTVNCQKDERVYAMALGSQLFDLKVYLSKLVGIYDSIKSTRLTLNVMEFN

SEFFIYGNQFRPTSRWVSAVNRIPETEAFSARQEQMANLLTEVLEGGGSVYLCSLLQWSQGLLHYRLLGS

SVNGLFFNYINALSLIKDPATGYFLMDHPMAAGLMGFKYNLWNVLMTNRTLSSKYKFMLQNMQEAIPKLK

EKGIQYKTLETTTSGSFVNSILVTWGNRQKWNRMIERLNLPENWLTIINNNPWVLYKKPETRSELVLRIA

AKIHSPGISESLSKGNAISRIMAASVYLISRNVVKDTSQWNLNEERRTQSLLKIALDDNKFLDGSVHEGL

SKDELRMLFPFSADYHEIRENLMHFINFAGKKQLELPKKRGTNIRITESDGDYLLNPIQLVAWKWFGIDK

LAAASRLKESSWNKLKENFNWLCDTPEETLKKSPFESQIQLQNFLSRLERKNRVVHLSGVAIKTNLGKSN

LLTAIYQNFYPGFTLDPVFDEQKAETSRSSRQVLHCLFMICAMPLTMSRKQQLMKNIIRSTPILPWDENS

ARSRRNMLHIVQKHEQSCGKIEVLQKIFHNKLGIIGGYIKPQRSKYIDGNVVYYGKGIWLGCMDDVTVRI

DVDSKDNMSYVTKVVLSSDTHVWATHKLIKSWLMDNNIISGIDIPSRDAAGFMNNQRIVMGPHGIGTPVY

LNSNLRYVYDMDSIVSIDTIYRNFTINLRAKIKHGHEFKEHNILSVSMNQSDINMEIGRMIKFEEGFPVV

AERWITGRSLKTSAARTLIDKTIDNESLRFTDLGENKIWVRDIIRESLRRKSLLPAAINLEQQSQTQVVT

TEEQDRLNRMIQNDIEVGFVQGIDFSTIMMQMSTQPDVCNPLADDVYEGVDILNVMEIDPRSGPKSHPIF

ENYIRYLIEDISTRQLQKLLIEKKISKANRDYLKFAKFILNCGEELFTDSLSSSESDIEESDDLF

>Zaliv_Terpenia_virus_(AHG54012)

MLLAICSRTNPQRGLNCPPAVTYTSSHMRPPIPSFLLWTEGSDVLMDFDLDTIPPGSVTGSSIGPKFKVK

TQAASSFVHDFTFAHWCDASDMPLKDHFPLVNDTFDHWTPDFISQRLDGSKVVVEFTTNRSDQEQSLISA

FNTKVGKYEVALHNRSSSVNILFGVVVVSETTVVTNLNLNQQEVDELCFRFLVSRAVHLEMTTKMIIPEY

DDVDEDKRSREVKAAFHSVQPDWETTEAKFSPFSRRMFSNFSQMEPDKEYLAHIVVDSIKAAQDDLDKNH

YLSESLTEQERLDRNRDESLKMVREFETEFNKGAERSAWSHKSTVPFPGVIPKVSGDTTSLSRLTELPII

TGGSDATIRAWRTAYGSVSNGTVERCDEDVERERRAALCSLSVEEIEESKALRMKYHRCKIDNGMMDKLD

LALQGVEAKEFRNHPTIIKKREESKKTFPLTTDTRDIDMFLHQDDSMFKQELSQTPPAPMIEAVKAGAEA

QSLHGLEKEDNPWYQSALTFLSLPIGLWLFMCTCIGVELSISLKQHCGRQKFIIKKLRFFDIFLLIKPTN

SGSHVFYSIAFPESAITGKLHRSQCFKGLQFEDGWFWTEFNSFKMSKLTNVVKCLSTGFNLFWFWRDFYE

VPFWAGSEKDYLAGKQRANKMFKFCLLMLLEDKARTEEIATLSRYIMMEGFVSPPCIPKPQKMIEKLPEL

ARTKFQVWLISRMLQTIIRISDYPFKITAGHKSASWSGMFNWVTGEPVDSTQKLISLFYLGYLKNKEESP

ERNASVGMYKKILEYEDKHPGRYTYLGLGDPPAEDTRFHEYSISLLKHLCIHAEHNLRQNWGESFKAMIS

RDIVDAIASLDLERLATLKASSNFNEEWYQKRTDGKVYHRSKVLEKVSKYVKKSSSHVHHIMEECLKKVE

SQGCMHICLFKKPQHGGLREIYVLGFEERVVQLVIETIARQICKRFKSETLTNPKQKLAIPETHGLRAVK

TCGIHHETVATSDDAAKWNQCHHVTKFALMLCHFTDPLFHGFIIRGCSMFMKKRIMIDQSLINIIDAHTA

LETSNRYLQLIHRGYHGSLEDQPKWMQRGGAFVQTETGMMQGILHYTSSLLHTVLQEWLRTFSQRFIRSK

VSVDQRPDVLVDVLQSSDDSGMMISFPSTDKNSTGKYRYLSALVFKFKKVIGKYLGIYSSIKSTNNTLHL

LEFNSEFFFHINHNRPLLRWITACDTISEQESLASRQEEMYNNLTSVLEGGGSFSLVSFCQYGQLLLHYT

LLGMTVSPLFLEYIKLVSEIKDPSLGFFLMDHPFGSGLSGFKYNVWVAVQNSILGSRYRCLLEAIQNSDS

AAPKKTLDTTTSGTFVQSTIIRFGDRKKWQRLVDRLNLPEDWLEIIDQNPEIVYRRPRDGTEVSLRIAEK

IHSPGVSNSLSKGNCIIRVISSSVYILSRNILSDGLAWLYDEEDELKRPLLFKVMNQPDLDAHSRLTPAQ

LSTLFPMMAEFEKLQTHLRSYMRIDGEFISKKKVITQTRINILETERFLRARPEDLIADMWFGFTRTRMT

PRTFKEEWDHLTAVFPWLKNNPAETLEQSPFQHHVQLRNFFSRLDLKGRDIRIIGAPIKKSSGISNVSTA

IRDNFFPRFVLTHIPDEAALERIEAAGILKHALFLTVTGPYTDDTRLEMCKDFIISSEPILLKPNHGKTR

SNVLSLFQDFFSMRGPDIIFNRIEMANCGVVGGFTSPQAPKEIDGRIVYTGDGVWRGIVDGFQVQLVITY

MPKQKINQLKSITVNSDRSIATLSSFCHSWCKEMGVFNTEDFSKSQRFTKASFYMFNFKISGSRQTLGAP

IFIVGERIFRPICWDPSKLEFRVRGNTLNLTYRESIPKAGQRMFNILSYTVKDTDVSDENAFKLMSLSPR

HKFHGREPSTSWICMRALPINTIDRLLDRILNRERISASIDNDRLAECFKSVLESALRRKGVFLSEFSRA

TQKMLDSLSRDMLDFFAEAGLNDDLDLEEEPWLGGLDTFSLDDEDYLEEYSLGPFGMFSVEQEMNTKYYH

HLLLDSLVEDIIQKLSLDGLRKLFQESEAPVEYKKEVSRLFSILQRDASRIVWKSRDLLSENMGLDVEDD

MFG

>Wuhan_Millipede_Virus_2_(AJG39266)

MSGLKRLFIGGQADDNIRLLNFPTVKNRIDICLYEIHLLTSLRLTPAHIFYDAIVGVYRSGLKKLSKFME

SDLGSHMSEDDVYCLPNQVTGKWVLDRMAQFSRTAAKLDATGRVSGSKTIRADLLKNNIADYQESIGLLD

VAMKLHQISVGGMTDTDILEIVHELRDKCCDLATKMLKMVTEDGLKCLAINVGLMCAMWRSISTYFPEQN

ERASLSKYNVNDFMFYTMSVISKFGENLSIAPEFDNFIDMKSHAMERSGVDRSLVRIHNANRDLKSMMDT

LKFNMNLIDRLQDNPLLHWLISRSYVKRPLSQPDIVSLQSLIGQLYRDHPEGLPEIIYANLQTIQDLAAK

IYNMYPLSDEVLSKWSSTIAIAMGEVRSSMLSAHYVQDDRLDSIFEQINATSDIQAIYNGVIHMRGFLYE

NHLSCAYGLKMCKDQVTLDDILSEFIIADADRTRQDSIDRLKRLLMTGDTLKKCPDLYMITGNRITLVEA

GWVNSAAVEMKHTQNLSRWVRVLEASNRENVELVVVSVCTDSTIGLWPSPVRPSNVGTTFRFNTSKKAVQ

EDLEFSDYKFFNASDSVRLSIRSLFKYCSTMMIEMGRPIGGDIFGSKYKPANQDGPSVPYVDINKATQCV

SLLCDEILHRPSGLDLSEFKGELMTQILGESDPNISSEEELFLLSMSEQYKTANIDCEQYDNMSSAERAV

KFQCLRGNLGDLIYDSDIKDTSAPIKDNTSVDASIKPGDTIHTWSKRLRGLTISNNSTLRVYSGRQDNDK

GCSVCRDKKGIVQQFDSITSWMHVNGSSTLSSATESQSIPDHNPMSVIGDFLITPFPTSTKEHRLMDKVN

TNIVSNFFSAMLSDCYVYRRSKLSYCKHTTPQIVLKMSDHSKTISHQHIVAMPVNIKVTSNETESFYPDI

ASKTGSELIKNLLQSLPSNFTIDSVTIDMTHMSKICPLEARRLIDIEALTNTISEIIKSKFHNTTYKEFT

LSFKKGMSGHMGGKLLTKVGIFDTMSNNKPSNSKGSTPTVNYGTFQEAHIKALGVRKHNKDIVPIEGEHL

QMPITAVYATLSSMRMFCTLYRNGVAMGHINVSNSKRLLSFIDELLKSIIASYEKAVVEIDNDLLCTVLD

HTMSVDVLPITIGLIWNVFNKLDDLEEGDIQPHNLAELIDMAINNMISTMFVNTDCDLIDFARLTLILPF

LKNQFKVWVSHSLSAFGNQSQLDDEDPVYQSESATNHPISRRLILSALIGGVGLRKITKSREEVNLEKRA

LDRLEAATTAYHPIEGDLKEIYSDSLSSHSSHKDIFSKGILLRESTKEYKCKCKNPYIHDDELFAYCRKT

FQRNKLTGIKSIAKDILSPESIQTAFESQSNISNELLDTAKQFMIDEMSEYSCMQSIYIKKLIADAFNDC

AGEFEKGCIKSSFIKEMNCTLVTNFTNDRSKKCQIWIINNSTGGIELKSTISQRTAKFWASLPYMILSLM

CLIIESLSEPGEPILNYQATLLQHKDLLIGGFNILLPAITKCSQVINKSMQQIRFGTMHRLGYFGGPDKI

LKKMCIPERSMCAVMLNELYRLTFVIHTQSISRLTSEWMPRDRSGSSGVPLMVLGGGLSHSDQAVQTDMY

VVHMYNKELENFDRSQVENFNKFSESHIDWELDIKRSYESGDYNRVRCLLGLPTTLSISGEVELVNRCNP

GHSMSSAKYMTLCGKMFLTTTSRDEIASTLKGKIAHPDIGDITETTGIIKEPLLNLNIRSLVTQGLSGYV

SKYKRLKQVTSIEDAVMKSLAVSDVNDSLGFLSTEDLRLVKDSIKDVKNILVLQPNKMFDEMVSGSEVGV

VSIYQQMLHVSKEFKQSRGGSKKLRAKKEVSKTEKDIGVESIAGNELADTKINVNIKDLRTSMLELVSSK

RLNEVSDLVGKELKKIEEVIQGKILRPDVLSKLYTYLSQGTVNSDSASTIRLEHVRSAIKGVYSTDRKNV

ADMVLKEFIQQITAAWEDSNNPNPLNFSYEISNLIEFEKLLYSEIADSFVEFGEIICSLAYIYRHRKRWG

LDTTVRSTIDQLGETVYDQFESWTYACLPSFSMSGDNKPCLLMCGLLMLIQGHISTSLIGNNNDFGQYIK

GMTFSAMATNGLKLTPLIYDYSLYSDVSVSSIESSLKRSANREFPRSVRSKVIFCMIETLSRYNDSDVIR

IATKILLSMIHQQYAGLASKEQLAGARDLFVLSVNTKLCTKVMESFSRAILDICPDDTLLHAEYKEQHLN

AGLSQKKLFFKGEVQSYTYACAAIGDNTSWGESMQPQHFLNYLKPILTCAPDWDGYVRLFLTHHSNKFVE

IPNNITESVINLIVTAKTLSIDRKSSPAGQLIKAIGITRQIDIEEAFKTSKNPRPNMNIIKNWYLSKGLN

YMRSYNHMGQGILHATSSLMGVCVNRLISVCITSICKSVDTSVDVMDFTHVGSSDDFNNNMAFRSSEMKS

PSEWRNITSRISRIIIILMRASLMKVSAKFSLSALTSEYYSTFCLGDIIQPAIIKFVITQVMPVSSGTMS

EMMQETWNLCQQQITEGTSVQMLYAFQYCRWLSHMWACKRAIATSEKLTDNALGSSLDIHNKKKQMMFLS

LTGNILFKPEFLIGGSLLQNILFYCECNTHRSACRVHGLGNRNFAESIYWISNVNVSLDESMKLFDPVTP

PMPLTIKRALSKVRSEKRSMIDDFTDRGLVVQDPVLQNVLMYNKSTTVTGNSNRLKQALSSNKVAQGIAG

GSRGCTGHRLRSSILGYYVATDKLKLPYTDIVLSLKDAANEHPLTSAITKMMPSRINTSKTVSRIRYIDL

MKMIYLSHSQAATHDVVEGISDCLDSLSPITVSMMSQLNTGEIEELHANPKMVFNRIYNQKGSTTYNDPA

SVVGYSVNPTLMSDLGISLISSENLERDMSILKPSIKMINDITRLNQIISTRGTTGDLESVDSPYNKEDQ

EEDTVIVSTIKKLQSFCELDSTGYGSTQLDEIKLDVSKIDIPGLIEQSIQKCFSKMYEDRVHAIPMHSRV

LGPSSWDSQVAQYYSLNSHPGFNLKITHAPSLISQEPKTLNTFIIYWSLLTTTSLTNFEVFSHLQDILAS

PATDMPHMVMKQVMISEISSLPIKMQAQFYNILDFISNPEMVGRSMMREVNVKHKHVKIGFSKGLITEYL

GVHSTVKICEMQSSTILVCSRINKTDIWRCIDLHIRKTFNKRRDDVVTIDEITDLMRKLEIPNNSDIMGS

DKLFDEICIALDKSGRSVSVSDGKPNLLIGPTGIPRRFRANRDELNANYSMSQGCKGGKLVVKGHFHGID

MDTVPVSTMLSDMIFSSDADSRQKSAMHSPRGKYSQELLSIRIPKFGSKKIMMAMIDKRPDYEFKLLIKP

VQSFKTLYQAMTGMTDSKVDEHNRLYGRLGKAGVSHETLKDIKERSKMSEKVYVEESPWPFQRNSDVIRE

VAQTFSRGMDILSKSALRFLIDHRLLLTSWQGIFEDAEETSIEFIINMHLVRTGQMTEVIKGVCRADIKQ

QYVDMVNRSNSSYLVLQVRTVSDFYSFIGVVGKFNRHLKKVSQAQMTEGSVVIQPKLYSDSPNLKLVIAN

IPNKFRTSYLEAVLSEISFRLGYTTSLNFSLDAIKALGESRIDRSIIEQLYNSPDRDMAGSAAANLMSSE

DDISDEDYNFDDPDDILHREEENDELIHEFSQFSNISVEEIIAELESNEITRDEILKLLNKI

>Hubei_myriapoda_virus_5_(APG79247)

MGIKWMATWISDYDNLSTLVLQKLQDVLWLWLRDHYSDFSIQKALTNYKEGWGLFESIDRNDFQTRFSPE

KSGKRYNSDHDVKELCRQAMNSLFFNMGSIVEINNLGIYEKHLVNDSFLRVHMSSLLRQSLKSHYIPFNP

EILQYMKSVKLQCSTISTKTIRELCKSSMIWQSASQGSYQGKLFEMIWEDILNIQIPEIQDVVQIIDKYG

GSSQMKINHFITNVHSESGIYHNDVSIFFNDLLNLMMGEDDRHELTQHIQMMLKTQWPMEIRSEVINIVN

MMVGTPELSVIHNLTSQIKGIVGEYIGCRFNQKEYIPEHKKKNIMEIIEGNTLCKLKYIPVLEKLTPDAF

KFTPDYSDKRVYLEVYEMGWVANTHMKYERDKAKWVYNMSKCHGIPCEVAGDTDFRFTLKLEDPTILSNE

EMEPIATRYGMQDIVAAVQLFHKHTRSYAGLDLDSGMNYIINDQQLQGVRTQRDRLFIPPEDKIELLVHQ

INSEVRSSIPDFDVNHYFKHSGKSNDNVERREEFLKFITKNIHLISSEGSKWQATTKDKKREIFTHIAQD

FLSARTIEELMSRGRFFHHKNTPFQIKEEHHPDTNPIQKRLEEHSHFLSHMSSKEQKKKKYMFSRTFQKL

IGYSWYVLPNGQYGHRKNRHDKMFEKPIVDCFSRDSAGKIFLTLKEKEVKNWHQVLSESKDTCNECKSLY

GRTVKDKKSLWHITLAKLQNSNDQLIYDDSSREDIVQSSFLNKSEDESDGEESGSLIDPVDDSDFYQEYS

DSDDDLNENQDVNNKKSDETFPCDDLDGYLEMLHSQVISSKDQNIKGPVVDVCQCPYPQDDQWKILQPSK

GFSESYLMDMICMCGARHNIRIDIPLISMIVSQMMDKVRPNWHAGHVEIIDAWSNYLKNKQKFIIKSIVR

QEYLNRNIQVKVQVNKPRKKTRQEDDVIILDDDASPEDVHVVQNILQPLYIIVLERFIENLMECSEMSLL

DSQNNEVIKHYCKSKWDYWLSCTPDDISREVLNEIVDKFLNIEQNCTALMSAECSLDIIKTKSDAFKMSD

CKGCVTYTPYIGFVHTDNANLSQESVRCIQLNGEFQYVNHRILRQWDAIPRFLISNHIIQEQVNMSAHHD

KFGKEYEYCGLMHQFMMSNSRRMNVIVQNLRYLSMVRWSKFYCKSFKKKMEMKVIKWDEFGLGVEVLRFS

RDLLYSDVIQNLFEQSNQKSHFKNYNAIIHQFYKVHFFEKSIPGVNQEMKKVYKKFLKPKLQDESLMQKI

LNDEKVWNPKCTTVQTVNKVIETFYSDKSESEKAELHAKALQLCHSAIDGHVSDDILFYQIQTVRSMDDD

NSIPLWSRKLIDDLFELYDQLPNDKYSDDVDKHKPFVELAKTSSTVDNFKVSLPFHRVLSSYDTCQKIQQ

AFKENDKSILQESYWEPSKMQGDYKLSSTFYQMCQKMMNRKSIILKGDLPDTWNEQMEEFLEQIRHCTTL

ATDILNPNHISLLSDLLNFMADCAKWLDTDVFSQMVKLNIMRSRRDNQFKIEEEDDDVQFRNTLRGFNIL

LPDMEQFDLMFSVFCALSYGNNDLECMDIINACKSGNITDLPHLTVLAAHVITQQLANFKDVIELCNKVR

VGNVNKLRNTIFQKKSVYRNTRMITTELIYTLSQRMKGSQYLTSVDAVEKLFLNDHPIFFGMSRKEQYGG

ERELTISDTEGKIVLKMLEDMFRHISKSCTNSCLNNPQNEEHHKSFTRRAQIGDKMMMVEKGLDFTPSQE

VCHEDCNNLLNKDENDDLDDKNSSISKPKMGDQMSYDEDEFQSLWFDDIDMNSLTEKDDENEVNKKDGKD

RKSEKCRSNIPDGKTEDKNIEDIQKKIGNDDADDVTRTIVQNKNGKGNGEIEISEEKKKEEKAETEKKQH

CAPSEYHFTEWYFSSEDHSAWGPCHHSLSFQKLMSNITLPKHIHKTINYGILRHQKKLNEIPASIIEGVL

SKVRHAYPILDHLSLKLIDDAEMFRCLNEEELYVWNLLKKDLLCSHHRFDMGQGMLHSASDVYGSFCSNV

SMKVAELVLKKLYPGFKMCWIDMNTSDDSNCMFRFLFPYGPGDNVSALRDKMLRDITNIKDFVQKCLNEH

MSPKSVVSDQISEFKSSFMFGGCESRVLVKFCSSQLMLGKDYMPHHFWESAYSLSQQCLANGGSMISVYL

LCASKFRHLDRAYHFSHCSPFLKEVRTLLPPAAAGFPIFTSTQLYFGCIHSIIMKACEKLTHKIMEEIKQ

KKIQVKVGQSIDTGQDNLRTIQISLIPEDSLILKMPLHLFHQNLPYDTPISKLLLTYFTFQHLKPQIEMD

NPGIFKPLPISSPKSIITKSSFKIMEHSEMNQIMKRIKTNPDEYMKYLIHNEFLENSPETAEYRSIQALK

SSILHSFKNAKHGQVYQKAYHISKGNFCHRTDRMVSMKTTLINSGAIITRLIDPRRLMSIMHPNSISICD

QFDHENLIFEDRSQVVPRHTDKLHTHEVRPVETDDLKWSNNPLAIYASLYHPNIFSIIPSDKLTLSSMVS

DVIEFKKHPELHDLDNLLDTMRCSKPNIIEGCYRFPATSNMFHDAEHIARSTQLHYADINVSYRVRVPEE

IDSHTDQDHLTKLITMCTIVANNPENELVKKKIIREYLTSPDYETDLGYLTLNKCRPGRIGDFCNCLLKF

CNLSEPVSGFKRKMQGGTNVIHEDIPGGYCTTISIEAVTWQMVKLNDKDPEILCDTNNPETWKSQIDLIC

KTLNQSFNVEQIRVDKSIRKKAVPVTFTESDNCSIQLTPNGFWVDSSRGMKSADSVWKLVGKLPQIYLNR

LTQIGQMHKEDYKYEPPDTPDESYYQFMKSYDVPEKTYVPFPIKIDIQSLWRLVKSGQLTLLTHGQHARN

EFKFDMELLNIVYDLTHDDSVNRIHRRSFIKGMSTSKKISRMMRLLFQEILEGDSEVNNKMVCFPIVQKY

FEVQCMNKDDNLSSVKFLSCMIEAIKYAENMNDLKSLKFWMDKLVDIVKVTGVMPALCIGSSRIMKISVG

KISDLNLHVTTKNYPQPDQKMKKKRMKRTSNRKTYRKKHGSYPECEDGSSSDEDPDKPEEPLDQKEEYPE

EVIDSKGRIVHFPEESGGYIKDTLQSVIDISEGRVRVTQKSKFQWLYFGCMPGTNLKKMQLECISMCGST

GAKILKGQIRVSDMNYVSKLVDIVSGNEAQDQKDVDAFNDSCEEFKIIYVAAIQNTVLDRATMIYDK

>Allpahuayo_mammarenavirus_(YP001649212)

MDVHLIELRDLVRKWVPDDLELSEQKNIMLAQTQIRATVVESLKLLSTIVEVDSCKKHSCVHNTSKTVNA

ILREHKIIGPTLPDVTPDGYCVIGDVLILLEVFVRTNQESFEKKFNQDFEKLMQMSADLKKCGVTLVPTI

DGRSTYYVDFIPDWVVERLRWLISRLMSSLREDGQEIEELEYERLISSLSSLENQSLGLESLLAMREKGL

SYKETLDKLFLEGMENKLTVDESRTRIMKMFQIFRTLLESGYLERKYQTTDREDMLKRLRDHEFIVCSKS

VEYTFDCPNCSVHLYKVLNLLLNQGSRGAPHQCLGEYMKTLSICNKIKSMKILNTRRNTLLILDTIMLNK

FLDLEKVFGHVVVERVMIMQSLMTVNDRLLSIDVLMEMLEKKMTRNPLWFLKVNEKLRKLCPPEVYQSIE

EYVHEVDRDHWFELKLTLHQTWPAKPLIDYKGKMRCTCVEKDSNNKNQLSDLTEEKFQLLLKKLSSFCLG

ITNSLKTSAVAKLRVNQPDDYYGKVTCSEVFFQSLDKEHSAVLLYQKTGEKSRAYGLAFNNVVTGQYTTE

ASFYCDPKRFFLPIMSDVVLFRMCNEMLSWLDYLSDDVMLEVRTCLYRLVLSILCTPSKRVQVYIQGLRY

FIMAFVNEFHCTGLLDKLKVTALTESERYCMKLCDDLVVKVLNSVEDENMAKAFKFVLNTSYLCHLITKE

TPDRLTDQIKCFEKFLEPKLDFGSVIVNPDSSCELTAGQEEQFYQGLEKLFTDKKLESSYANKPGVCKEV

LNVCMSLFNSGALEVKPLLNHDPITPSFTSTALDLSSNKSVVVPKLDELGEVLTEYDYSKLVSSVVVDLV

EHFKTKGKYVVSPRSLQYKIYKRLSNLVQQRAGKGNKESELTEEEFLEQVTAEQLEVINKVETKVSRTLS

GIKLSSDTENAKHDDDYHLKKLWSKDIMVRIKAETSLHEVKDFNVDTLPFDLYRELVDAIYNDPAANSHY

FSERIFNPCPLELLIKNLTLKAYKEEDFFECFKYILISSNFDNKVGKYDHKNRSRLGLSSAALLVKDEAR

ISMRESNSESIAKRLDKSFFTNSSLRNLCFYSDESPTERTSVSSNVGKLKFGLSYKEQVGGNRELYVGDL

NTKLTTRLVEDYAESLTSDMKYTCLNNENEFERALLDMKSVVRQSGLAVSMDHSKWGPHMSPALFSLMLR

GLDFRLKDGTLIDKEAVVNILSWHIHKMVEVPFNVVEAYLKGFIKRGLGLMDRGGATRVEEFMFGYFDQG

IVPSHISSVIDMGQGILHNLSDLYGLITEQFIVYALDLCYSSSFMAYTSSDDEILLSISNSFKRNDGSMD

MDLAIEALEFHYFLSDRLNKFVSPKTVAGTFASEFKSRFFIWSQEVPLLTKFVAASLHNVKAKAPSQLAE

TIDTILDQSVANGVSIEIIGAIAPRTNALITYSGHPFNLFLCLEETDVRDWVDGSRGYRLQRSIENAFPD

DVLPEIIRSACRKIFYRIQSGTLEEDYIVTTLQQSPDDCLKQMLTSCDVEKEAIDDICNYRWLNLRAHGD

LRLVLRTKIMTSTRTLQKEEVPSLIKSVQSKLSKNFVRGAKKILADAINKSAFQSCISSGFVGVCKSMGS

KCVRDGKGGFKYIKDILKEIKHHEKPDCHFCKELKGIYCSELLENISEFSRPLFWDYFSLVLSNACELGN

WVFCKIEIPKSVYHLNNPNHFWPIKPSSHAELEEKVGMNHVLYSIRRNFPVLFDEHISPYLSDLNMLKLN

WVQKIRFLDICVAIDMTSECLGIISHIIRRKREELYIVKQSELSMSHTRVSLPLERGFNIEPDEVCHNFL

LQILFESMIHPVLLTTSQFKRYFWYSEVELLPKEALHDLGQFTQFIIDCKVLNSSRAMCLDDLDVGYVSS

KVKRTDTYLNLSTFMTNLDWENRHEYSSFEDLILSSPSEVFLFEITFTFSHIRRSHKFRYDRSTNYILKT

KLVIEKSELVNGEDGVYCVTPHSIEYYVSQSSGNHISLDGVSLLVLDPLISGRELVNMDELLQNQDVTFS

APSQILSKIKLDFKPFTKEIKNKFSYKLIGPDVDMSPLHLDKGAIKEGDRIVSQIEIQVSFKSVITAIEL

LDEDQRKIFVGNLFVYLTSLKSVNRALSMSESDLRLLVENYPSVIEYMLSGCDGWLNCGSFSLIKSKTLQ

CIMLADERGPYRIKGQNCRRLFPTEEAIEIE

>Bear_Canyon_mammarenavirus_(YP001649225)

MSEYLDELKELIRKWIPDEEMYIEQKTSFLSQVNLRSVVIEGLKLLSIIIEIDSCKKHGCVHNKNKTVNQ

ILRDHRIVGPTLPDVVPDGYRVIGSTIILLEAFVRVSHESFEIKYKSDFEKLMQLSKDLSRCGLTLIPVV

DGRSNYYTEHFPDWTIERMRWLILKITNFLRDNGEEIEELEYSRLVYSLSNMENKNLGLESLKILKEEGL

DYKAKLMSVMRDGVNSNMSASECRVEMAKIYDQFSFLRKNGLYKDVYCKTSRTEIINWLKDHKLILLSGE

TRTAMLDERQCGYCRNHMFRILASLIKNKRHYQSLTNPKKCGSIQSHKKLLSDCNKIKGLKVLNTRRFTL

LCLDVIILNSLLELIDAGEIDNEFLVNNHFKSVNDRLVSIDLIIDRLNKKLMSKPNWIGSVKYKMKRTLE

IHGLYYVSKWLKQVDIDSWYEFKMMREHSDKCVKPTLKYKKDAARKCGQPEFGSSTILDDEVFLEYLEAL

STLSLGLVNSMKTSSAAKFLINDKSNYFGTVQCNECYFQDLDKSYNSLLIYQKTGERSRCYGLMFKSEQF

ENVYEVGESFYADPKRFFLPIMSSEVILKMCVEMLSWLDWLSEQELKAFKSKLYTLIINILTVPSKRVQV

YLQGFRYLIMAFVNELHFKELQNKLKVQPLTISECYVFTLMDDLVHLLLTEAQEENMSKVFRFVLNLSYL

CHLVTKETPDRLTDQIKCFEKFLEPKVDFNSVFVNLDSSPHLSGEVEEKFIKDLNRLFSKDLGVEDLKDP

GISKELISLCASCFNCGLLPMSKVLKHDPQSPSFTSTALDISSNKSVVVPKLDEVGETVTQYDYQSLLSS

TVVDMAQSFKDKLKYKLDRKSIQFAIFKRLTNMVLKRKTDHDVKDDLDDELSEIVDDDTLRVINDVEANV

SECLSKMGKISRAATVGGQNNLGRFEKIDTLKRLWDRESMNFILMETSLHEVKDFDPSIFPIEKYKSMCE

LVYDSKMKSEFFTDEVLKFCPLDLLVKNLATKCYLEEDFFECFKYILISAGFDNRVGRYDHRSRSRLGFK

DEAILIKENSRISSRESNSEAISRRLDKSFFTNSSLRNLCFYSEESPTYRSTVSSSVGKLKFGLSYKEQV

GSNRELYVGDLNTKLTSRLIEDYFESLTSECKFSCLNNDAEFERALLDMKCVVRLSGLAVSMDHSKWGPY

MSPAIFNILFSNLNLELNDGVFIDKAPIENLLNWHLHKIVEVPYNVIDAYLKGYTKRRLGLMDRSSTSIT

EDFIFNWFAKGVVPSHISSVLDMGQGILHNTSDYYGLLTEQFILQCLDFIFDIKSTAYTSSDDEILLSNS

PSLKKVDEDSLDINKCQEVLEFHNYLSSKFNKFVSPKTVAGSFASEFKSRFFIWSQEVPLLTKFVAAALH

NVKAKSPHQLAETVDTILDQCIANGVSIEVVKAISRRTNKLITYSGHPKNPFLCVENTDLKDWVDGSRGY

RLQRSVESLFNDDDLPLTIRNSCRSLFHRIRSGDIQEEFLINALQTSPDECLAKMLRLSDVDESTIDKVL

EFRWLNLRAHGDLRLVLRTKVMSGTRILDREEVPSLVKSVQSKLSKNFVRGAKKIITDAINKSAFQSSIC

SGFIGFCKSMGSKCVRDGNGSFQYIKHFLKSIILHSHCEVCKPEMSVFCRAALEELKPFSRPIFWDYFSL

TFSNACELGNWVFSNVTIPKRTPTTVNPNFFWPVKPGSHTELEDKVNMNHVLYSIKRNFPDLFDEHIAPF

LSDLNSLKISWIQRIKFLDLCVAMDMSSECLGIISHIMRRKREELYIVKQNELSVAHMRDSSPMEAGYQL

NSSEICHNFLCQLVFESMLHPVLLTTGQFKKYFWFGEVELLPNEADHDLGQLTQFVMDCKTLNISRCMSL

DDLDVGYVHSSILMGDIYVNFSSFLHLLDWENRRNYKTFDEIILCSREDTIPMEIDFTISHSRKSFKFKY

ERKTNYHIKSKVLVQKVDIEEAQNQGFDILELEVHEIECFVSGSQGNHISLDGVGLIPLHPLFSGKEFLD

VNKLLIKQDENFESTHSVFSKVKLNFSNHTKDLKNKYSYKLQGPEYNMNPLHLYRGQIMENNFVISRLDV

QITSRSVFLALEALESEDRIPFLISLHIYTRSNNKKENSCFIRMTQSDLCLLIDSYEKEFTEVLKSLSDW

MDFGDFALCFSNNLNCIMIADPDGQFKLKGRQCRKVSSASAPLEID

>Golden_Gate_virus_(YP006590089)

MSSICPEDSELERLKGLVLEVLSYERDLYRPGWISNTAGEMAMNAIKLRSTIHELNCCRDTGLKHNNDLK

EMNELFDEAIGSHETVKTIVPDGYLIDKNNNVLTVLEVSTRTEPSDQTKKVQLDRLKYDGFENLLRPLGW

TLNVITISEKKPRIGRIPEILMFKLLSTSLSILSYTTDICNWISEEDYLELKKSLTSYDFRTLTEEFKGQ

RLLFDIESAEDPYKDLFDWMLKNSEKLPFSLNWEGPKITEMIQDFKREDKQLRLLEIMRSAVTGLNFRTN

HLSLLNKLKSLNLLNTRRKQNKVYDYIALMLFVRDSEIHDFPKGWFPSVNDRLVRVDSVDSPLSVYKKLV

ERMIKSLQSLQNEVGSKQKLGELKILIKFLEESSNNFIEPPVLKTMYFGLPARVLVPQPSLCLKIINDVP

YEPQTTIVTSDETDTIEKVALNLILSQKTRSTPRASMSEEFFFQSVNGCLLLYKCTGEGQKAFSILCKSY

KQGELRYSFYSFNINPSRLFPLIFSAKPIANLVDQLIMLINPICEDAERLQETIKRQESVNEFLISSEYS

DKQIDDLKVKVTYLIYGILTTPTKRLQIELQSQRYYIMDTLSIIHHKDLKAKVIGNCITYDEYYLRDLAH

GLINEILSCKNIGLFLKVTWALNISYLCHLITKETPDRLSDLRDCYEKFFKPKFQFMSNMIYDCKSVDEL

VERMEQEVNDFIQCEEPSFETKPFLYEPILADFLKWISTEKFNGIDGEQLLNPKDYLTSNIDVLDLTSNK

STFKRSKNHHMNDVLDQQYDTEKLLKRVISERLHKRITKSTKAGDDPSTAKSFKSGGESVKERNWKAGGE

KRKKTRLAYEEFLGIVQGLIVETDSEEDLNDFLLQINKCLHEIDDELKKMNPKEINDLQKTKYCLVLDVI

RELLGPEVCDLVRSTCYSTKLSDLPTDLLRKEAYERIVGETMSRAPLEFPRNVDTIDVTNISAGMVVTKY

ELKEFFSSFKFLLLWAGFNNFQGTYDHRSGPQSSFLSISNKYRGESMLSTRVTNSEALQDRLIAIKYGAP

TLRNILFSTLNLEMKNSEIGPNNKPLQFGLAIKEQVGGPRELYVGDSDTKLITRVLEETSRNIGNRLNNS

CLNSDKKFSNFMKRIARSFFENEIVLSMDHSKWGPFNSPLQYHLMFEAMESINDTDGRRLDFSFAKTILK

WHLFKAVETPQILAEDVILSSLDVSLGRRDRQIGKERTFETFMVDAVLSNKPVPSQIHSWFDMGQGILHH

TSDLYGSLASEYITKKIKEIFGVRSSTMNTSDDMVLLFHIKYKNEDSNQKDELLLITNFLCLVSNCLNKH

ISPKFCCSPLVGEFKSHFEVEATMVPLFTKFFAASINNFRCKTPMELFNTCDAIVEQGICNGMSLKVADS

LKGRMVEMLGWLGYVADPLMKPVESRQQDWLEGCLSYRKLRSLESWLMDLGIGKLKLDVLKLELLKVIKE

LRESSIAPSVAYNKMVDISKSELGEITLWFPTLNGEFKLIVRSKLNLGTTINETCENLLIKKLINHYSRY

SKQGLGLLIAEGLERSAFQSSVVTGFIGLSISLSGACIRKGDGTFLTLKESKNVVKPVAEVFPSLVMQSV

CAVGDWVCGVESEMDKQMNKSIYMRTSLVNTAEFRFGLDELTAAIEMTMPDLFDKYLKDIVPPDKRLLMR

HSWKVRPEMELLIECANKGLSIFDGRIDRQEEAVVLVDYYEPGRLLRRTMKLEKRGPERTARKGLQAIVN

RMILDSILEPKIIDKTMVMDATKLAPYEGSMEDLKSHGESGVRRYIQQVLLGRESHYRQKEDEPNEITSS

NVVVYGRGTPEFPLEDGTWLRGAKLGTSNPIKLQCCLQQISNDLKIQATLITHTGRAHYQDFMFLKRVII

SDSDFDEPEILDTLEFRDVVVVENSKPNGKPCFLFMGCVIPLESDLVGSIMTHMSEAEWEELWKLVRTYF

ITRMKGEISCLPKARFDALWGMVSRIFGYSTVEKVVTMGFHQLDSFAEFLTFTDRVFTTKGPTLVFHRLK

GLLVSYPAGPLLYKGHRLMLYKDWASGTPEPETLED

>CAS_virus_(YP006590093)

MDPDTNLLKNVILEILSIEPDLYKQSSIVDDPYKLAMSAIRLRATIHELNCCRDLGIIHNTKEISLNMVI

DRAIPIHPTFQHIVPDGYTIDRANMTIIVLEASTRSMPSDQKRKITSDKLKYSGVEDHLKHEGWLFNIIV

ISETKPRNGNVPERLLFELLKLSLSILSYSDKSSQWISEEEYDELKRSLTTYDFKTLTSEFSGTKLYTDL

DEPSETLDADILSWLASLETQNKLPFNTAWDGPQITKLIHKWRDIELQPKDYKPLRLMRLMSSATTGLNL

PTKHLSLLNKLKSLNLLNSRRHQNILYDYMAFMSLPEMNDYPSGWFPSVNDRLVSVKSIDSNFRQLEKLK

KRLMISMFKLQASTPKGYIHDLLKDAIEMVKSCKIVKPNFKMRNRGPVPEPFINYKIAREREGKTKDYNN

IDKQRIEDCALNLLLSQKTKSCPRSSNETEYFFQSRKGQLLIYKASGEGQKAFSLLVETQVAKNHHVNIF

LSFNINPSRLFPLIFSSESINAVLRQYKDLLTFIDTPIADITDNVIKSQQAINPFLTSNIMTEELAKQTL

HKLKVLIYGVLTNPTKRLQVELQSQRYYIMDALSLIHHKDLVSKVVGNCITTDEYFLRSLAHDLINTVLS

CGNMSLFLKVTWCLNISYLCHLITKESPDRLNDMRDCFEKFMKPKIEFMSNPIINSKNVDELIDKFEDES

ERFFLNAPTIEVKPSVYKPVMSDFLRWVKMNKFNGQNGEPLLSDENVLSSSVDVLDLTSNKSTFKRTKNG

EIESALDVLYNPKLIIKRIICEKLHKNTVKSSSIKERNKGITTEKSTIIVKLEELSSTIERIIGDEGLLD

ELEYGEFLLQMLDVADEIKEEITNKAGVPDEKELTNYEIVLETISSIVGTESKNLIKSTCMSTKLSDLTS

DILSEDCMKEIIESVITRLPSSFPRDAELVSLDNVSAGLVVARLQEGDIFSSFKFLLLWAGFNNFQGTYT

HHSGPQSSILKLSDKYRKKMMISSRVTNSESIQERLLNVSFGAPTLRNILFSTLDMDIKNSEIGPNNKLL

QFGLSFKEQVGGPRELYVGDSDTKLITRVLEESSRNLGSLLENSCLSSEKKFDSFMKEISRAFYNGYIIL

SMDHSKWGPYNSPIQYHMLYEALEEIRGANGKKLNLAFAKTILKWHLFKAVEVPVTIIEDIYQTMTDISL

NNRERRPDKERSYETYFINQLKTKKIKTHDNKVPSQIHSYFDMGQGILHYTSDLYGSLASEYICNKVNEL

FNIKLISMNTSDDMVVIVKRMFRAKHINSIREEMMYLMNYIYALSNFLNKHVSPKYCCSPLVGEFKSHFE

VEGKVVPLFIKFFSACIGNFRCKNPTELFNTCDTIVEQGVCNGLSLTVADCLKKRMVNMLDWLGYSSDPQ

MHPHTSRQQDWLMGCLSYRKLRSLETWLLENGVEELTLDVVRGKLYEVIQSLRMRMIAPIVALEKMRDVV

RTHLGKIDIWFPTLWGGFRLIVRSKLNLGQTLVESCENPLVKKLMNHYSRFTKQGLGLLISEGLERSAFQ

SSVVTGFIGLSISLSGPCVKSENGNFIPIHQSKQITKEVCEVYTTLLMDSVCSIGDWVCGEDRQLNKFQP

HGVYVRTSLINTPTFNYGLDELTATLELTSPELFDKFLATLVPIDKRLNLRTNWSVRPEMELLVECSKLG

MSVFDGKFDRKEESVMLTEYFKLEHLVTRSLPLQKTHKSGTAVQELNRICDGMILSSILRPQMVEPSLIG

DVLKTEPFTSSLEVLKDIKVSGLMRYIKQVLLGRKSYYKFGEEHADGDQNLFDYQFTGTPEEPIKGYWTT

TISYRDSKPKISLTIRQEFVEGGVESQAVLATVVGRPHLQDFLLLKRKHLEYSDYPESIDLIEFGDVKVI

EKTVGHGQPVVLYMASLFPIGLTKIAEQGIGCHNEKNDATLKSLLKTAILNMTPDDVSLLPRSKLDAIDH

VVRNVYLRFNNSSAVSYGLHKLQSFEKILQLNGRIYETKESTLVFHHKKGVLVEDLKGLMSYKTLRVSEW

REKQEPEEARVEVLEE

>Haartman_Institute_snake_virus_(AKN10711)

MAKEIFERIVSEIRLLNGQGFPFVPDVSQVEMFGALESESLKAEGILRLRGNIAERLLCSKYGLKYEDES

NKRNITELLKLINVTNCSSNLTPDAYAITEDGKLMIYEFGISKNRREEKEHSDRMKWTKVCNNYPVLVHV

ETMTSFDEVENPHVRSTLQNLEKKMIKFFKKMEKNEELREINLKYFWEIRDLFQQYPYLNKFKDPKRPNK

EREEALEVFPDLDRKNFPIIDNKILSNQIRNVAKERIKNNKSFYVKTNDESMEELWLKFKTNSLKAYRSI

KTTKQGFKKTDEAVLNYFQQNQNIINLLNKFNKEQKLKRLDYSYIFSIMGLEGGLVPGPHDSLRWLDAVV

RDCEVRLKHGGKVAQKERKRLEDCFEQNKLIFESYKPMHDTLTNKLKLLEQKLLKSKKTRIQDDVNKSHN

ETYILNCAKKRMENWSNQNLQTVEETSMCILRSANTSYTYKNSFLCYQVFGSQVVFYKTRGQRAKQFGLI

SNNGISSFTAHPSRYICPSNIQLCIKNIELEYKKYTNEPMNKEVRNCILEILLNQNKTTQKNIQNIRYML

MGLNSMFHARDLGKKISIQLKNDKNAVDYWLFKNLYDNYRKNGSIFPFEKTDDQQLETRKMLFMSYLCNL

ITKDSQEKDIERIKANQKYFELKQNWANERNWRLSNMLSIEEISYGENKGPTISPAILNSFYEWFKLECL

NYLDKFSYTKLKEPLAFDISNSNSCMTQVKNEKNYNKISITNQLQKYHDRKMMSINKNNDLKDNKNPDTT

NKVRQLNEELQEYTDFYVNSGNSWVLDQLKTTEPYQLCLNLPYIENLKELALRTLESNEEDDLTTIIKQR

LISTEDPDSLHMCLLLRIKNIKNDLRITLKQEKGLKQTKFQNKLSSRNSTAIELLNEIRKEKIKNKEIIS

ELTCIDLLSEIPNMNFALSYKEQVGGTRELYIGDIKTKIATKIAEEFAKQIKDINPISCLYDHSSETLIR

KHVRNCQLAKNAIVDLKFEDISNLNDEQLSSVMTQSEEYLFGSLDHSKWGPLSMPSLFADMMDIFNDAIG

IVGTLKNDLSLISDILWKHVLKKVEISSEYAEYLIKNDNTELKTSIEPVEINLLENTIDVYANKLLKEGK

LGMQTYPYDMGQGILHGWSDIWAGKTEEFIWDFIKNHITDFTDVYNCVTSDDQASVLIGPENAALTLELH

YILSKCLNKKISEKSVWGTKVFEFKSVFVSGGQELPPTLKYLIIPSFGFEVFDPLNYLNTTDTIMQEAYD

NCATIEQCSNLLDMSKKLLSCAGFGSQYLNDLSQRHFYTKKLHATMFDNMTYTERKLTSLKESSEFKTIK

NQKLVAEIDTELSNWMTRPYQSVENARKIAKQCKEDSWDPLMHGPIRVCSGRMKNNKENNIILSLDPSKN

IEDPISAKLYNFIRKHFNSTTYSSLEQSIIESIKDSLRQMSSGESYSGLVSTIRSNSFKLKEGYGNILTS

QKENRQTILEDYVQLKVQLLIEKFSLDDRHFFPTIGIEEKPMISTEIRGNSVNDDFLVPALASLEIRSPI

FFKEVVEPSIPLKKMKLMNSQQLIRMKLIEAADASFNMQIREPYEVFRLVSLNNNSTYYQGNDNICWEET

DHVGLSKRNYNLKQTETVMLLKSFVKPVPLNYETILNAWESVTRCEFPNNVPIEGSVIQILMANAINEKR

QTQVCELLKSFPQHLSEKRTYQDQFTIIVSREILWQQQSVTICFSRNKIDKTNIFTIYWDKLPRYPEKKV

THELLKKLLSDDEIEEWQLSGMRIRNKNKLNPNLFDKLSSPVFIKEGSLGYYKYLEGIPTFFEVHSPRLD

HHSLTSQLNKLNICDYLASILESYAEMGHTVYEGIVDTKPDYLTTHMGNIITFKASVHSAIEYLTDKQKL

PASKLFNQCFDEAIILEVAQGFVLSRPDVEGWIYSKNSCFKKRDSAIVWRIEDIEAPEEEEDESWLN

>Wenling_crustacean_virus_9_(from_a_crustacean)_(YP009329879)

MDWARPHRLKIRQVSAAFTDEVPAVDAGGMVKDLRIERHNGFISYLNSQNTDIDFGVDSTGDIPAEYIHK

CVTDALNVEPDPNLNIPSKTPDGFMIYDGSLYIIDVKTVTNLELQDDTVRKYREVFEVITNRYGINLSVN

TVNFHPYHHTITTYPNADVFPAIPVSEDLIAFSDEVNQIIQRIQARWQGNQTFLAAQHDAAVNFKGSFTE

RDDWMYKCLLHPEWQGVKEAMGVPIAKLMKRCITGNLTQADRNELSDYYRRSTMAKGYSMKHKLLTEGSI

PHIMTQGTADEVTNAIRDMHTNTAPSKSFVTQAKPSCFYYSTRGHHGLPNGNYARIAKFAQFAQNCRSQH

PLIDVLKNVHISDAELYETGDIPVNQEGFSFRARQVLKLSDGEIRNKLFRNAKVGVDYVKRSARDSRYYT

QEKPECLDYTDAGILEEHSNLIHDQLPLLNEKVYLANDVSHMYMDDREMEKKNPEAIKFLKWMRETYAFS

WLSDQEDRLPNMLAVAGLLKNNEIGFILGKSNTSMHIVYPGKSLTSQESSMCYNSIHILSDDKVPVDCGL

IDAYIKIPRSIYALVITKPVRLDPTRVGVLTCAHTRFQLVALHYILTALDTYDKFENIPLFLKNNIIGVS

FFLVQQCTLPFTNLVDKMKYIVQDVLADVSNVSGYISEKLDITPKNSFTMFLYKHFMTQIKFLRQNFREG

RNQPIVVEAEDVIEAGIEGLVIPSVVIPELQLPNVQAVIQDYVLAWYVTSKGLHNQVHNLLKLYKVPLEF

QEELNIQGGMRPDVDKRLMLASFIKYWDTRGSSSIEARNRCFTKMKIDRTWTQIPTLTSTKSVVHKLTEA

EKRVKTIDEETLLEMDPDIFKRTYPRYRRINGRVLDRKTGQVPLELRNKELWHQVRSSRVFDEMERLSSG

LNPDMEIVNPRSVIEITDHGLELTPCVTVFPKQQRTGDDREIYMVDIVTKCSLYLIESLMKQLCVENQRE

CISLPGDRKTQKIKSLLIQSMRNQQEFSDRETNGMQFMKLNISADASKWSARDYSDKFMLFVATLPCLLP

QEKWKLIFHLLNYRMKVVVLKDSVLETIIDKELGDDNVFWRMTDGFTKNSVKVTQNWLQGNFNYMSSLVH

AIMHETIDDCLVHFNDAMAEVNGNIPTTHYPLNSAVHSDDSLLSFCFELPEDISLEKVANCTFDLMVTTG

KAFSIKYNDKKTFISKDIVEFLSKLFVNSEYHTNYMRSLLPIFIELPFGNITQDMASGLGPLTQATAEGA

PPSLVYLAQCLLPYRIAHLNAMLPGMVNDVGQYFQNGFHQLPTCVTGSIGSDITDSCILGGAAYDYSRVF

EILKSCAVPFIEIPRMINSIIADRISPIDMEFLKILSISLEGYREADITQVGTYGVKDRFFNTALIPMNI

SQVKETNKYLASAKYSYLSRDGGLAGIIQTLRAQHPMWSISKPVYPYEYADYNLYKMTELKYRQALFTPH

SSHGFLLKILNRRTKVVPKKALEQHDPFMPVLDATDESEKLTLNEALGYLKAKADSVTLTTDKIRAIMST

MLLSNQQVIDYLTTKNSAKVTSEIPKKSRFIVQEPSGFRQKVVQNPPSLVIKKYFFPAEFNNDSDVLYLD

EAVQSDLDTISDVLRAYNITDLARTDHPDYVQNITKILSLVFHHVTNKMGSNRLYILPSRMQSGPKFVWT

LMGSRQNDARHFVYTSIVPNIHMYLKGNRQRGDIQMQRTRLQELFGLLWVLDHLTAGVGDQRGPLSILIG

NTNVEGTPMREVLQNGLVDKYTRSICAAILYWGGCMPYDAFKRQSIPSIVQKEWLVAQQTADVGDFHVIY

ELRGVALVVKGSSRVITAIKFEIPDGQELLGVEGPILTAFYKDFARIIQVISLQTANVRSNSRSYSLRYK

SFRHDPQGDWLIKTQRGFGTRITTLTEARFLGSVDACTWVKFPIPYRSQNMELKTLKPYRPFLDHMNVTT

NTPLFVNRTDLVKLVNIDSFRDAVVNRGDTYNYMPWLECLSYRGIGFQMTVQNDEDFFEPTPSIPVGERA

IPAVSFFETYNKNWHWYHKMFKLYLRLKDAFSVKADGTPDPNGLRALTHLLQIEELMWYRTDRVGFAMFL

AEWVGQLSALNPDNISVEVFAGSEPVAIRKANYVIAKLESLRAHRPNVFNVDIAIVKNYRDTYIQDVEIE

WGE

>Khurdun_Virus_(from_Fulica_atra)_(AHL27169)

MFQIQERITQLQRRQQHLHNGTIAAELLSDVYMLRHDVVGMLICDAINIPYKDDCPFDEIVNDVEAYVGY

KLDIKQKNYSPDNYLLDNRRLVIIDYKCTTSNQYIADTYNKYNEAFRELADRAHLELVVAVINVNHRTRQ

VESTTHLFDNELLAIDTYNQELNTLCTIGDEITRDWETDDDFLAGRAFGGQKSTPCWTDRDVDLFENDEF

VKFLQSLPSKYQDILLMNMENNPSKITEWDAKLNRTMEMLKADYNREIKEMADKLFLEKKSKEPTTKEIE

EGWLGLEAAMREKYDLTLEYEKQKPSAHFVWAPQREETKIINTNTRKITYLIKALKMIQPQNEYQKLFRD

LANRIDFTDHVEEYETFIREKKNGSCKLQKKQIGKADVYWEGQFSISQMEGRKLLLKEAGVGAHIPFKRK

NPEDADTSRPKMLNPFNELLCIKAESQFETAKMILSRKSPEIYTHNVIEHYKEKIKSCNHETEAMVDDIT

KSDFFRCLMDISTLLKGALSTSLASKKDSIRVICCANPNVFLITYPSTDIKTSGSTLCYSVIALHKEQIP

DFGSTAEKVCIGDSYITISRPMRLDKERVKRLIESPGLFILLTILMSEYCNSTLQDVMAFTFFCSTSITK

SFMTLTEPARYILMNSSAIISDVKSYLSEKFDPMTKTFFSIYCFKKLQTASLNVHNSFKSINPATVKMSD

TEIIHSGVESALDIPCVWFDGKSTLKGYINHAYLPFYTNSKGLHNPHHNIIELTKVPLEIERNLPKDSFW

GKGSKQKVDLNVFIYALAKHFIRLRKVNPNLRETVERTNMFKENIVKISTMTSSKSALVIGDFSDLKKSN

KEKKKINSLSIRKEETNEGEQQKFIEENLCYQHCDYDDVKNYVPNYIDHTTIPVFDILYRTAKEQDIDRP

FIDLAFDSIRNHNQYYFTLFPKDQRTAKDREIYEGEMQAKLALYVLERIYKTYSKGDINEMISEPGDRKM

KIMEENKNTMLRWIASQSQNGKQCSLLEINADMSKWSAEDITSKYIFVIALDPSLYPNEKKHLILFLCKY

IKKKLIIPDSAIKNIMDQKKEYDNCLIREATADLTTNVVEITQNWLQGNLNYTSSFVHSIAMAVYNEVIT

EAASRNESICKVISMVHSDDNQTSICATQGAIKDTEWAKFCSDTISEVMSRFGFQVNKKKTYASHIIKEF

VSMHNLAGESFSVYGRFLLPCISNCAFMGPYEDISARLSSIQTALRHGCPPSIAMVGIMMSTWATYDTYN

MLPGQTNDPEKILNRKRFDLPLEIGGFPDMDLAVFELLGVEAHDINLLCGIIKKYSNDPFGNYLINEVEL

TKPLENYDEWLMKYYLIFSVGEITDKDAIVGETFEMKQRSLITPRRFTTYRKIKKLESYNDYVVELANDQ

CETLFEFIENHQHLLIVKAEDSEEYKKSIIYRFNSRKFRESISVQSSVQLFLEQVLFSHRNCIDIDFLEH

HSKLIEEEQITETGLTGKITIVDAFNKLNEKVQSTTITQAGMQTVLKYKTQTDPMIATLINAQRMFHAGQ

SLEPLSLKCSTIPNFRTIKATNNSPAEILMYYINNRVNDKDIILLEQDKEYLLDFLKESGLLISHEMRLA

NASTRKEKLSERTRFLQSCYVYLTSSASKNKITLIQRKCYNLMEFLTTVFGATRIDGVFITTIFHKPIIL

QSVKGRAAKTRFTASNQALTLFKSLAYFADKFIAASSRRSFCQAIIDDFFFNGMKVKDLLDIALEERDVR

KEILPFLYRMKLTTSEMLRDAIVLTRKPKANWHVPQISRTPARGPFSVTYETDDTTLTVTGQDNLLEFAE

AHSIELKPSRLKDIFDRMSHDLDMDEFRTYPGKLDSGYFIVGRRFKRKWNLTIKRDLENLSNINVKVFCK

ADFHIRELVKVDMFRIHELNKFTDTMTKIEIETDRTLIMRRGNPRLLTALAGPDLTVSNISLNKLLESPA

LLSNRMRDDESAIADIINIFNCKGETITEFDFFGDEEMLDFVDIPFELNIPSGAQNLEGRKIKYKDDLHS

KFIYAGKKLTDELKLRFDFTEEDAIAYSTISGIIDFLKINSWCTHFQSAVHIAASVNGFDKIYHSGIHDS

FLTLKGLDYEKVIKLCNRISMIKNQKTYWNSVISKVMNTIVQKCHKELAQQAMSSESSLLDMFK

>Akhtuba_Virus_(from_Anas_acuta)_(AIL53813)

MFQIQERITQLQRRQQHLHNGTIAAELLSDVYMLRHDVVGMLICDAINIPYKDDCPFDEIVNDVEAYVGY

KLDIKQKNYSPDNYLLDNRRLVIIDYKCTTSNQYIADTYNKYNEAFRELADRAHLELVVAVINVNHRTRQ

VESTTHLFDNELLAIDTYNQELNTLCTIGDEITRDWETDDDFLAGRAFGGQKSTPCWTDRDVDLFENDEF

VKFLQSLPSKYQDILLMNMENNPSKITEWDAKLNRTMEMLKADYNREIKEMADKLFLEKKSKEPTTKEIE

EGWLGLEAAMREKYDLTLEYEKQKPSAHFVWAPQREETKIINTNTRKITYLIKALKMIQPQNEYQKLFRD

LANRIDFTDHVEEYETFIREKKNGSCKLQKKQIGKADVYWEGQFSISQMEGRKLLLKEAGVGAHIPFKRK

NPEDADTSRPKMLNPFNELLCIKAESQFETAKVILSRKSPEIYTHNVIEHYKEKIKSCNHETEAMVDDIT

KSDFFRCLMDISTLLKGALSTSLASKKDSIRVICCANPNVFLITYPSTDIKTSGSTLCYSVIALHKEQIP

DFGSTAEKVCIGDSYITISRPMRLDKERVKRLIESPGLFILLTILMSEYCNSTLQDVMAFTFFCSTSITK

SFMTLTEPARYILMNSSAIISDVKSYLSEKFDPMTKTFFSIYCFKKLQTASLNVHNSFKSINPATVKMSD

TEIIHSGVESALDIPCVWFDGKSTLKGYINHAYLPFYTNSKGLHNPHHNIIELTKVPLEIERNLPKDSFW

GKGSKQKVDLNVFIYALAKHFIRLRKVNPNLRETVERTNMFKENIVKISTMTSSKSALVIGDFSDLKKSN

KEKKKINSLSIRKEETNEGEQQKFIEENLCYQHCDYDDVKNYVPNYIDHTTIPVFDILYRTAKEQDIDRP

FIDLAFDSIRNHNQYYFTLFPKDQRTAKDREIYEGEMQAKLALYVLERIYKTYSKGDINEMISEPGDRKM

KIMEENKNTMLRWIASQSQNGKQCSLLEINADMSKWSAEDITSKYIFVIALDPSLYPNEKKHLILFLCKY

IKKKLIIPDSAIKNIMDQKKEYDNCLIREATADLTTNVVEITQNWLQGNLNYTSSFVHSIAMAVYNEVIT

EAASRNESICKVISMVHSDDNQTSICATQGAIKDTEWAKFCSDTISEVMSRFGFQVNKKKTYASHIIKEF

VSMHNLAGESFSVYGRFLLPCISNCAFMGPYEDISARLSSIQTALRHGCPPSIAMVGIMMSTWATYDTYN

MLPGQANDPEKILNRKRFDLPLEIGGFPDMDLAVFELLGVEAHDINLLCGIIKKYSNDPFGNYLINEVEL

TKPLEIYDEWLMKYYLIFSVGEITDKDAIVGETFEMKQRSLITPRRFTTYRKIKKLESYNDYVVELANDQ

CETLFEFIENHQHLLIVKAEDSEEYKKSIIYRFNSRKFRESISVQSSVQLFLEQVLFSHRNCIDIDFLEH

HSKLIEEEQITETGLTGKITIVDAFNKLNEKVQSTTITQVGMQTVLKYKTQTDPMIATLINAQRMFHAGQ

SLEPLSLKCSTIPNFRTIKATNNSPAEILMYYINNRVNDKDIILLEQDKEYLLDFLKESGLLISHEMRLA

NASTRKEKLSERTRFLQSCYVYLTSSASKNKITLIQRKCYNLMEFLTTVFGATRIDGVFITTIFHKPIIL

QSVKGRAAKTRFTASNQALTLFKSLAYFADKFIAASSRRSFCQAIIDDFFFNGMKVKDLLDIALEERDVR

KEILPFLYRMKLTTSEMLRDAIVLTRKPKANWHVPQISRTPARGPFSVTYETDDTTLTVTGQDNLLEFAE

AHSIELKPSRLKDIFDRMSHDLDMDEFRTYPGKLDSGYFIVGRRFKRKWNLTIKRDLENLSNINVKVFCK

ADFHIRELVKVDMFRIHELNKFTDTMTKIEIETDRTLIMRRGNPRLLTALAGPDLTVSNISLNKLLESPA

LLSNRMRDDESAIADIINIFNCKGETITEFDFFGDEEMLDFVDIPFELNIPSGAQNLEGRKIKYKDDLHS

KFIYAGKKLTDELKLRFDFTEEDAIAYSTISGIIDFLKINSWCTHFQSAVHIAASVNGFDKIYHSGIHDS

FLTLKGLDYEKVIKLCNRISMIKNQKTYWNSVISKVMNTIVQKCHKEL

>Brazoran_virus_(Culex_sp.)_(YP008400138)

MEPFNLPPRDLVVEYNKLIRQCQDPHTGRDILTAMTMDRHNYFAREYCRAINIEYRDDVPAVDIVMEMVP

DLDLGKIEIPDVTPDNYYRDGAKIYIIDFKVSVSDETGQLTYKKYNNIFGDIFNPLGVQYEVVIIRMDPS

TMRMTISSDNFMQLFPQVAMNIDFNWYFRLKDSLFDKFRDDEDFLALTSHGEFTPTMPWVTDDTPELADH

PIFIEFMESMPIDQQRDFMTAMNYNAFKSEKWSDLLHIYMKRYGQKYEKFIRDQAKEVFLVDGNYNKPTQ

SEILEGWKEMYTRIQDAREITQDISKQKPSIHFIWTHHDNNKPTGNEAKIIRLAKLLQKIDVNDNASKAF

KAMGFLMDFSSDVEKYRNFCSNLKGEARSSIKPKSSRIEPVQINQCTILWEQQFKMDAGIMNKNVRIRFL

KDFCGIGGHKNFKDRMIDDIDLGKPRILDFEDKNVIQAAEIMFKQTKGYLSKESGLEKIGNILGEYKDEI

TGASEKTWLHIESISKSRFWQCINDISVLIKNMLSVSQYNKHHTFRVVTSANNNFFGIVYPSASIKSKQS

TIVFSSVCLHQDPYDVLTCGSLYRTYKLGPNEYISISKAIRLDKERCQRLVTSPGLFLMTCLLFKGDNDI

LLEEIMAFSFFTSLSITKSMLSLTEPSRYMIMNSLALSSHVREYIAEKFAPYTKTLFSVYMTQLIKKGCM

SANDQKDKISLKDVFLNEFEITQKGVSMERNLQSIWFPGKVNLQEYIMQVYLPFYFNPKGLHNKHHVMID

LAKTVLEIELEQRIELPNPWSTTFIKQSANLDILIYSIAKMLKMDTAKHNHLRARVESRNNFKRSLTSIS

TFTSSKSCIKIGDFEEIKSKTVDRLAKLKNKEARRTRVANSDFVAEDEIDLEVAHSNYKDLKKCVPNYTD

YMSTKVFDRLYESYRTGLIEDKPAIEVIMKTMKTHTDFKFCFFNKGQKTAKDREIFVGELEAKFCLYAVE

RIAKERCKLNPEEMISEPGDSKLKKLELNSESEIRYLIDALRDKNKDPDAKLDGIKIEINADMSKWSAQD

VFFKYFWLIVLDPILYPKEKKRIIYFFCNYMNKELILPDEMMCSLLDQKAEREDDIIRQMTNGFRTNTVN

IKRNWLQGNLNYTSSYVHSCSMMVFKDIIKEMALLLDGQCHVSSMVHSDDNQTSIIMVQDKVNNDMLIHS

FCNLFERVCRTFGNQANMKKTYVTNHIKEFVSLFNIYGEPFSIYGRFLLPAVGDCAYIGPYEDMASRLSA

TQTAIKHGCPPSLAWTSIALNQWVTFSTYNMLPGQTNDPARIFECRRDEIPIELCGLLKSELSTIALVGL

ESGNISFLTSLIKRMSPVKVLKESIQTQCQFINEWDLEKLTKMDILRLKILRYVVLDSELNEEDKMGETS

EMRSRSLITPRKFTTTSSLERLVSYKEFQDIISDQTRSNDLFEYLLAKPELLVTKGEDANEFMTTILYRY

NSKKFKESLSIQSPTQLFVEQILFSNKPTIDYSGIYEKIMSAADIPQLQNYTGIIGRKTIPETFKAIQED

LEGLQLTTSDIHMVYSFCILNDPLNTTACNAILLSQVQSLMDRTSMSAITMPEFRSMKLITHSPALVLRA

YIHRDFTVGGAVEDMMQRDVFHLEEFIQTTRIREKVERKIQEKNALTPDRDLVFELKEWTKFYQTCYDYI

KSTEHKVKVFILPTRAYTAFDFCAAIHGNLMKDKGWYAIHYLKQIVSGSKKAFVSTTPTGEQLVIDECFR

LVSHFADTFIDSSSRRYFVQTVVEQFTYKNLPVSELYRKMKNSSQRKHFLPLLFNMKDIKQNDLDKYNAD

KTANKATWNDWQINRHLNTGPIDLTIKGDTRTMRIVGEDNQLILAELYINEGDYTPPENHARKLLNTKHN

LRFEAMKEIPVMEPDCYYICWQKKSKYYTHYQMLLSNVINARNEAQATGANKYNRLIPVCIVHVARVQPE

SKVLVADLQYMNDIASFTRLKMSETDYAMVRRCHFSKMVFFSGPEIGVGRVNITRLLRNPSLLTINYSSL

SQTPLPALIDIFECNGVEIEDDEFNFLSDEILEETETQTINAVPMFNISYSVKTRRGHTYKNAIQEAIAR

GVNDFEEQFDFSGEGFFSSKGLSICALIVSLIDRCNMTEWSAVIKKCIHLTCYTNGNDRIFHNFKIPRIY

LLDPINYTIDWEKVRLFILGIKIRDENNHWGTVFKNFFKDLLDAMDEQRKVEGLSWADLMEQMDEYKGAC

GIDYQE

>El_Huayo_Virus_(from_unknown)_(ALS54742)

MAHRLNDLVRQFAARIRACNNPELGRDLLSDITVARHNYFAQEFCYSIGLEYRNDVPALDIVMEMVPDFN

PTLIRVPNITPDNYFRDGNKIYIIDFKVSVSDESGNHTFKKYNTLFGDVFDQLGVEYEVVIIRMDPSSMH

LHISSDNFLNIFPNVILNLDFSWYFRLKDELFERFRDNEEFMELVAHGEFTPTIPWVNEETGELFDHPVF

IEFMNSLGPEVRNDFFQALNYNAFQADKWNDLLHMFIRKYSLKYKDFLRKMSRKIFESDSNYNKPSRLEI

QKGWQEMIDRVRETRDVTTDIHKQKPSIHFIWSPHDPTKPHENNPKIIELSKALQRIKENDPTSSAFKYI

GKLMDFSSDIEAYEQFCLRLKRDARSSSRQKSTPIDPLTVGECTALWEQQFKMDTHIIPKEIRLRFLKEF

CGIGNHKQFKNRMLDDLDLDKPQILDFSDTNVQRTANLMFQDAKSMMSKASGLAKVGNVLEEFRDKIENA

NEKTWANIEDIAKTRFWQAINDFSVIIKNMLSVSQYNKHNTFRVVTSANNRFFGIVYPSASIQSKKSTIV

FSTISIHKDQKDVLKCGALYKTYKIDNQYISISKAIRLDKERCQRLVTSPGIFMLTTLLLKGETEIDLNE

IMAFSFFTSLSITKSMLSLTEPSRYMIMNSLAVSSHVREYIAEKFSPYTKTLFSVYMTELIHKGCMSANN

QRSKISVKDVFLNEYEITQKGVSEDRDLESIWFPGHVNLKEYINQVYLPFYFNAKGLHNKHHVMIDLAKT

VLEIELEQREQLPNPWGTEFKKQSVNLDILIYAIAKMLNNDTSKHNHLRSRIENRNNFKRSLASISTFTS

SKSCIKVGDFSEHKTKTAKRVKKIQEKDAKKTRIANTEFVAEEDRDLEIAHSTYMDLITSIPQYTDYIST

KVFDRLYEKFKTEEYGDVPAIEIIMDVMKNHKDFKFCFFNKGQKTAKDREIFVGELEAKLCLYCVERIAK

ERCKLNPEEMISEPGDGKLKKLEINSESELRYLIEMTRKEMSKEEEYVSTLTNEPKGIKIEINADMSKWS

AQDVFFKYFWLIVLDPILYPYEKQRILYFFCNYMDKELILPDEMMCSLLDQKAERENDIIRQMTNNFHTN

TVNIRRNWLQGNLNYTSSYIHSCSMMVFRDIVKESVELLEGNCNVNSMVHSDDNQTSVIIIQDKLDNDCI

LHYICDLFEACCLTFGNQANMKKTYITNNIKEFVSLFNIYGEPFSVFGRFLLPAVGDCAYIGPYEDMASR

LSATQTAIKHGCPPSLAWVSIALNHWITFNTYNMLPGQINDPTRIFFCDRDELPIELCGILRADLSTIAL

IGLEAGNISFLTNLLKKMSPPQLIKESVQSQCNNIEFWDLSKLTRMEIIRLKILRYVVLDTDITEDNTMG

ETSDMRSRSIITPRKFTTISSLTRLVSYADYQAITQNKEEFDQLLEDMLERQELLVTKGECPDDFIKTIL

FRYNSKRFKESLSIQSPTQLFVEQILFSNKPIIDYSGIRDKYVGLLDIPQVQESDTIMGKKTIPETFELL

NKDLGLFDLTNDDIKLIYSFCILNDPLNTTACNAMLLSQVQSLMDRTSMSAVTMPEFRNMKLIRYSPALV

LRAYLHGNYTIGGATEEGMKRDIYHLEEFINQTGILVKVEDRIREHEERLAERDMLFEIRERTKFLQTCY

DYIKSTEHRVKVFILPSKVYTAFDFCATIHGNLIKDKGWYSVHYLKQIVSGSAKAIVNQNPASEQINMDE

CFKLIAHFADTFIESSSRLQFAKRIVNEYTYKNIPVKKLFEMLVKDPNKRQHFMPLLYHMGELFQSDLDK

FDANKTNERVSWNDWQVNRDMSTGIIDLTIKGYMRSIRIKGEDDRLEIAELQLVIGDNTSVESHGRKLLN

TRHNLKLEKMQNHKFLEQGMYYICWQRKNKYTYTYQILLAEIIENRNRQPASLISGRVNLLNPVCPVIVS

RTPSMEKIRLTALRYLNPTCELSKLKITQNEYATTKRCHFSKMPFFYGTDFTVGNIDINKLMSTPSLLNV

NYASLCQTPLITLSQIFKCDGQSEEIDEFEFLSDETLEEIDSAPVNAIPFFNVNFPTRSKKGFTYKQALQ

QALSAGLNEMEQEFDFTGTGYFSPKNIGVISFMIGLVNRLNTNEWSTVLVKCIHMSFFNNKKDRTFHLFR

IPRAFVKDPIGEKIDWNKAREFMVLHLF

>Guajara_orthobunyavirus_(from_Mus_musculus)_(AKO90159)

MAHLIRELVRQYQARIRACTQPELGRDILTEITVARHNYFAQEFCESTNLTYRNDVPALEIVQAIRPGFD

PMSRQVPDLTPDNFLIHGNKMYIIDFKVSVSDESSIHTYRRYTERFTDIFNFIGVDFEVVIIRMDPSNMQ

LHISSDNFLQLYPNIILNLDFSWYFRLKNELFERFRDSEEFMELVAHGEFTPTIPWVTTETPELYDHPVF

IDFLNSMPPKDRNNFLRAMSDNAFHSDKWNDLLHTMMREYGDKYKAFIKDMAKRVFLADSNYDKPTRLEI

QKGWSEMILRVKESRNMTTDVHKQKPSIHFIWSPHDPKKTNENSMKLLMLSAGLQDIKEKDPFSIAFKHI

GKLMDFRSDIRGYERFCARLKQDARSTPKSKSSKIEPLQIDSCTVLWEQQFKMDTGIIPKDIRLKFLKEF

CGIGNHKQFKNRMLDDLDLDKPVILDFSDPDIQKHANLMFQDTKSMLSKESGLYKIGNVIEEFKDKIINA

NAKTWETIESIAKTRFWQAMNDFSILVKNILSVSQYNKHNTFRVVCTANNQFFGIVYPSASIQSRKSTIV

FSTVTFHDEAKDVIKCGSLFRTYKITKGYMSISKAIRLDKERCQRLVTAPGIFLLTTLLLKGDTEIDLNE

VMAFAFFTSLSITKSMLSLTEPSRYMIMNSLAVSSHVREYISEKFSPYTKTLFSVYMTELIRRGCMSANN

QRTKISVKDVFLNEFEITQKGVSENRDLESIWFPGFVNLKEYINQIYLPFYFNAKGLHNKHHVIIDLAKT

VLEIELEQREELPNPWGEDFKKQSVNLDILIYSIAKMLKNDTSKHNHLRSRVENRNNFKRSLASISTFTS

SKSCIKVGDFYAHKSDTVKRMKKIQNKEAKRTRIANTEFVDEEDRDLEIAHSTYLDLIKSVPNYTDYIST

KVFDRLYEKFKTEEFDDRPAIEIIMDVMKDHKNFKFCFFNKGQKTAKDREIFVGELEAKLCLYCVERISK

ERCKLNPEEMISEPGDGKLKKLEMNSESELRYLIEMTRRETTKEGSFLQEFTSEPKGIKIEINADMSKWS

AQDVFFKYFWLIALDPILYPYEKQRILYFLCNYMDKELILPDELMCSLLDQKAERENDIIRQMTNNFHTN

TVNIRRNWLQGNLNYTSSYIHSCSMMVFKDIVKECSDLLEGNCNVNSMVHSDDNQTSVIMIQDKIHNDCI

LNFICKLFEACCLTFGNQANMKKTYITNNIKEFVSLFNIYGEPFSVYGRFLLPAVGDCAYIGPYEDMASR

LSATQTAIKHGCPPSLAWVSIALNHWITFNTYNMLPGQANDPSKIFNCERSELPIELCGILKSELSTIAL

VGLESGNISFLTGLLRKMSPPQLVKEGVQTQCNNIENWDITKLTTMEIIKLKILRYIVLDTDITDDNTMG

ETSEMKSRSIITPRKFTTLSSLTKLTSYNDYQDVVSDPDQLEALLEGMLENQELLVTKGESPEDFRKTIL

FRYNSKKFKESLSIQSPTQLFVEQILFSNKPVIDYTGIREKYVGAVDMPNNQEDEGIMGKKTIPEALEIL

RDDLSKMLLTLDDIKLVYSFCILNDPLNTTACNAILLSQIQSLMDRTSMSAVTMPEFRNMRLIKYSPALV

LRAYLHGELTIGGAIEDDMKRDLYHLEEFIDQTGILRKVEAKIQAHEIQNGARDLLYEIRERTKFLQTCY

DYIKSTEHRVKVFILPCKAYTAFDFCATIHGNLIKDKGWYSVHYLKQIISGTAKAIVNQTPASEQINMDE

CFRLISHFADTFIEPSSRHYFADKIIKEYSYKNIPVTDLYDQLKRDMNKRQHFMPLLYHMQELKQSDLDR

YDANKTYEKVTWNNWQVNRDMSTGTIDLTIKGADRSMRILGEDDKLQIAELRLVQGDTTAVESHARRLLN

AKHNLQFEKMQEYTYLEPGMFYICWQKRSKFAYNYQMLLCEIIENRNNQPATLLSGKINLLHPVCPAVIS

RIPSPEKIRITALKYMNQNCELSRLQLLKNEFATTRRCHFSKMTHFNGKEFVVGNIDINRLMATPTLLSV

NYPSLSQTPLITLSQIFRCNGVEEEVDEFEFLSDEVLEDTDTAPVNAIPFFHVSYSTKSKHGYTYKQALQ

HALRIGLEEMENEFDFTGQNLGFFSSRNIGIISFLVGLVNRLNTNEWSTVMMKCIHMAFFNNQKDRTYHL

FKIPKIFIKDPIGEKVDWAKAKDFLQGIRPRDETNHWGQMFIHFKNKCIDAIDLEIKMEGASWGEMLEML

DEFKDEGMFNFG

>Mirim_virus_(from_unknown)_(APM83098)

MAILIEDIVRQYQARIRACTDPEIGRDILTEITVARHNYFAQEFCASIDLEYRNDVPALDIVMEMVPDFDPTAIYVPNVT

PDNFMRDGNKLYIIDFKVSVSDESSNHTYKKYTTIFGDIFNQLGVEFEVVIIRMNPSNMHLTISSDQFAHLYPNIILNLD

FSWYFRLKEALFTKFQDNEEFMELVAHGEFTPTFPWVTEPTFELMQDPIFIEFIESMDAEHQDDFFYALNNNAFQSDKWN

DMLHMFMQKYSQKYKAFVSKMAKEIFIIDENFQKPTSREITLGWNEMIKRVGEMRNVTKDVHKQKPSIHFIWSPHDYKSP

VDNNKKIIRLAQALSSIKDKDNHSLAFKYIGELMDFSSDISKYENFCISLKAAARSSIKKKSEQIKPITIGKCTILWEQQ

FKLDSDIIPKEIRLKFLKEFCGIGGHKQFKNRMLDDLDLEKPTILDFSDIEIIRHAQIMVTDSKSFLSKKSGLTKIGNTL

EDFKDKILNANENTWKIIEEISKTRFWQCINDISTLVKNILSVSQYNKHNTFRVVTSANNNFFGLVYPASSIQSKKATIV

FSTVTLHKNEKDVLNCGSLFKTYRIGAGFISISKAIRLDKERCQRLVISPGIFLLSALLFKGDSDVELTDILIFVFFTSL

SITKSMLSLTEPSRYMIMNSLALSSHVREYISEKFSPYTKTLFSVYMTELIRKGCMSANNQRHKISVRDIFLNEYEITQK

GVSDNKDLESIWFPGNVGLKDYINQVYLPFYFNAKGLHNKHHVMIDLAKTILEIELEQREELPNPWGDKFEKQSVNLDIL

IYSISKMLNNDTSKHNHLRSRIENRNNFKRSLTSISTFTSSKSCIKVGNFYDLKDAMSKKIRKQLEKEAKKTRIANTEFV

NESDRDLEVAHCTYSDLVKSIPHYTDYMSTKVFDRLYEKFKTKEYEDKPSIEIIMDVMKNHKNFRFCFFNKGQKTAKDRE

IFVGELESKLCLYCVERIAKERCKLNPDEMISEPGDGKLKKLEINAESEIRYLIEMTRKKTKEEEFLSKLTDNPKGIKLE

INADMSKWSAQDVFFKYFWLIALDPILYPFEKQRIIYFMCNYMKKELILPDEMMCSLLDQRQSREQDIIKQMTDNFSKNT

VNIKRNWLQGNLNYTSSYIHSCSMMVFKDIIKETSSLLEGSCNVSSLVHSDDNQTSVIIIQDKLHNDFIINFVCNLFEAC

CLTFGNQANMKKTYVTNNIKEFVSLFNIYGEPFSVYGRFLLPAVGDCAYIGPYEDMASRLSATQTAIKHGCPPSLAWVSI

ALNHWITFSTYNMLPGQQNDPTKVFNCSREELPIELCGILKSDLSTIALVGLEAGNISFLTSLLKKMSPPQLTKESVQTQ

CNNIKNWDLNKLTRMEIFRLKILRYIVLDTELSEDSTMGETSEMRSRSLITPRKFTTASSLNRLISYKDYQDVISNPTEL

DSLLQDMLDHQELLVTKGETSEEFRKTILFRFNSKKFKESLSIQSPTQLFIEQILFSNKPTIDYSGIQDRYLGLLDIPAI

QENEGILGKKTIPETFGSLLEDLSNFTITHEDIKLVYSFCILNDPLNTTACNALLLSQIQSLMAKTSLSAVTMPEFRNMK

LIKYSPALVLRAYLHGDLEVGGASEDAMRRDIYHLEEFIKQTGILQRLETRIEEHNRLTGQQDLLFEIRERTKFLQTCYD

YIKSTEHKVKVFILPSRPYTAFDFCATIHGNLIKDKGWFSVHYLKQIVSGSGKALVNQTPASEQINMDECFRLIAHFCDT

FIEKTSRVSFLDNILKNYTYKNLPVKQLYNQLINSPTKRQHFLPLLFYTKELTQSDLDRFDAYKSHERVTWNDWQVNRDM

STGAIDLTIKGYMRSIRILGEDDGLKIAEMELIVGDNTPIESHGRKLLNTRHNLKLERMKKIQVLEPNMYYITWQKKSRN

TFTYQILLSDIIANRNKQSFSITGSKYNELVPVCPVLISRIKSVEKIKISAIRHLNQDYNLTKLKITQNDFAVIKRCHFS

KMMFFYGPSFVVGNIDIAKLMTTPSLLTVNYPSLCQIPLITLAQIFNCNGDNQDLDEFEFLSDELLEDIESEVVNSIPFF

TVSYASKSKKGHTYKRALQEALRSGLTELESELDFCKLGFFSSKNVAILSTLVSVANRLNMNEWSTLLIKCIHMSFFNNN

KDELFHKFRIPKAFLKEIIGEVVDWQKVKLFIDGIEAINPDSHWGQMLGHFKTKAKLAIDLELKLEGSTWGEMLDMIDEF

RDEDMFQFN

>Pacui_virus_(from_unknown)_(AIN55741)

MDQGQINQFTQRIDAANSAEVALFIDTDLYEARHNYFGRELCQSIGIPYRNDVPANTILEAAIPELVGTDFFVKCSPDNF

AIRDGVTFIIDYKVTVTDETIQTAYNKYYEMFEPYFNEYGRQFEIVIVSCHPNSGVIKHTGVLFDNYFHIGDMDLDFTWF

FNLKQALYDKFREDEVFLAHAGPGEFAMTTPWCMDGADLDIDEEFMRFMATLEKPEQILFKEAMAYDPFTADKWSDFLIQ

TKSKYKEDYYSYVTESARDIFSFGKKLEKPSHDEIELGWFEMYCRLQEERNMIRDPMKQKPSIHCIWSMPDSKKKNDQIS

KLTTFSRKMRKIKSTDQYAEIFRRIGENFDISTDPESYTQFCTRIKNESRLLEKERKSKKVEPIIVGTSQVLWEQQFKMD

LNPITPLQQSSFKKNFLGIGKGKRFAIRTENDVDVEKPKILDFEDSVVIRAAQEMMCNTKGLLGRRTNLEKIGCFIDEYK

SKISGASKETWETIERIASTNYWAMVNDYSILMRNILSSSQYNKHNTFRVCFCANNSMMAIVFPSSDIKTKQATTCFATI

AIHKNRDDVLNPGCLYKTFETEEGFISISKPVRLDKERCQRVVTSPGLFLQSCILMYNNNPTIKLDDVMNFCLYTSLSIT

KPLLTLTEPSRYMIMDSLAITSSVKGYIGEKFNPMTKTLFSVYMARLIKDACSDAFSQKHLIQPRRVALDDYDITQKGVE

ETRLLKSIWFRGFVSLKEYINQIYLPFYFNAKGLHEKHHVMIDLAKTVLEIESEQRRENYFPWSDTPKPQSVNLKVLIYG

LARQLHTDTQRKSYLRMKIENRNNFRRKMYTISTLTSSKSCIEAGDFTDQKSQNLKKSHNIANPAFSEEFEANVKVEKSN

YIDLKSKIPDYIDIQSTKVLICYTKRQEMKRLGDGGAIKYMLQSMQRKEEYVFSFFNKGQKTAKDREIFVGEYEAKMSLY

VIERIMKELCKSNPEEMISEPGDSKLRILENTANSEIRELISLSKQNRQEVENDPNVVKKKPLKIDINADMSKWSAQDVT

YKYFWLIALNPILYPEEKSSIIKFLCRYMNKKLILPDAMVNSIFDQFKLYEHDIIKDMTNDFKQNWVSIRNNWFQGNLNY

TSSYIHTVSMATYKDILKKAMEWLEGTAHVSSLVHSDDNHTSIILNQGRIGDDDLIRFCYDAFVLVCLTHGNQVNKKKTY

VTNGLKEFVSLFNIFGEPFSVYGRFLLTSVGDCAYLGPYEDLSSRLSSVQTAIKHGCPPSFAWVSIAMAQWLSYSTYNML

QGQYNDPCDALLIQDRFKIPIELGGLIDCPLHVLVLLGLDAVNVFNLYKIIKKLSPIILRGNRIEDIIQTSPKWRVGDLS

DEDVFQLKIMRYVTLGVEIDSASKMGETSDMRNRSILTPRKFTTQRALSRLESYKDYKAMVASDIEYNNNLQYMLEHPEL

LVTKGECSEDYTNTILFRYNSKRFKESLSIQNPAQLFIEQVLFSKKPTIDYTRIHDKFTRTQERDDSQIIGKKTIREALE

SIRRDLKFYTVEIEDIMTIMNCIVVNDPLAVTSINAEILHVLTDSKKRTGLTCSTMPEFRNIKIINHSPAVVIRAYVNPG

FCPAGADYRILERDVWFLQEFINETKIRDRALQNIQINELSKGEKDLAFEIQEWTRFYQSCYSYIKATEHKVKMFIIPTK

VSTATQFCQAVIGNLKHDSVYYGCYFQKNAIGYNQKGMVSQIHDMAILTADECFRLICHFSDQIISKQHRVYFLKKLIES

YKFRGHSVVELLNRLLESNKRTSFIPLLFHLGELTEEDLDRFQNELGQKNVTWNKWQTNRNLNTGPIDLMITTDTASLIL

KGFDNTLEYSRMCISNPTYANIRSAGRMLLNSRHGLNLETLDSCEVSPKLWYICSQRRNAKRIFYDVKKGSDVLGENAAN

AAEGRRSYQVIAHCEMEVVYSQKEPRLDYQSIEILNSEDVYLSKLIISESMYATVRKIDLSKMQDFVGPDLVSSKLNITK

LMKSRTLMSCNYDNVISASLMELAGVMDCKGTNEDFSFDFLSDEVMDADEFDVIEATPNLKIQYGKKGQSYMTLQSAFHE

IINLKSELFKKTLTFAGPEFYSPENIVVLTNFIAMTRMLELEGEALEISNVLHLIFASNDKDPMYHMAEISQEFMSGDQP

NYRHLNMLIDSMKCKHENIWSFLFTKASIQIVQALRRRESESQIDIVSLIRQISRRVPNQSDFNFSRIRVLRLCVIYNKN

VCTLCKQTKQFNI

>Caraparu_virus_(from_unknown)_(ABQ14448)

MAILLEDVIRQYSARIRNCNNPEIGRDILAEITMTRHNYFAQKFCEAIGIEYRNDVPAADIVLEMMPGLDLTRIRIPNIT

PDNYYRDGTKIYIIDFKVSVSDESAQHTYKKYDTLFGDVFNQLNIEYEVVIIRMNPSDMHLHISSDNFAALFPNITLNVT

FDWYFRLRDDLFHQFRDNEEFMELIAHGEFTPTIPWVNENTPELFDHEVFQDFIASMPLENREDFYYALNHNAFQSDKWN

DLLHVIMRKYGDRYHDFVKANARRIFLTDDKYNRPTKEAILQGWKEMIERVQEQREIIDDLSKQKPSIHFIWGPNSKNES

NENNTKIIRLSKNLQSIKEKDSFSVAFKNIGLLMDFSEDIDKYEAFCAKLKADARSSLKPKSKKIDPIKIGPSTILWEQQ

FKMDTDIIPKEIRLKFLKEFCGIGNHKQFKDRMMEDLDLDKPKILNFENQDIKNQAYAMMQNTSYFMSKPSNLVKVGNVL

EEFKDKIVNANEETWATIEEIAKTRYWQSINDFSVLIKNILAVSQYNKHNTFRIVCTANNNFFGLVYPSTSIQSKRSTIV

FSTIVLHDNESDVLKCGALYKTYRIKNGYLSISKAIRLDKERCQRLVISPGLFMLTSLLFKGDCDVSLNDVMNFAFFTSL

SITKSMLSLTEPSRYMIMNSLALSSHVREYIAEKFSPYTKTLFAVYMTDLIKRGCMAANDQRQQISIKDVFLNEFEITQK

GVTNDRNLQSIWFPGKVNLKEYINQIYMPFYFNAKGLHNKHHVMIDLAKTVLEIELDQRVNLPTPWGVDLKKQSVNLDVL

IFSIAKMLNLDTSRHNHLRSRVENRNNFKRSLASISTFTSSKSCIKVGDFKEFKERNALHMKKLQGKEAKKTRIANTEFV

SDNDRDLEIVHSTYLDLRNSVPNYTDYMSTKVFDRLYERFKNHDFEDKPAIEIIMDVMRCHTDFKFCFFNKGQKTAKDRE

IFVGELEAKLCLYGVERIAKERCKLNPDEMISEPGDGKLKKLEINGESEIRYLIDATRNQTREQSKVDDLLDTPKGIKLE

INADMSKWSAQDVFYKYFWLIALDPILYPFEKQRIIFFFCNYMNKELILPDEMMCSLLDQKAQRENDLIRQMTNGFTTNT

VNIRRNWLQGNLNYTSSYIHSCSMMVFKDILKEVSALLEGRCNVNSMVHSDDNQTSVIMVQDKLHNDIITNFVCNTFERC

CLTFGNQANMKKTYITNHIKEFVSLFNIYGEPYSVYGRFLLPAVGDCAYIGPYEDMASRLSATQTAIKHGCPPSLAWVSI

ALNHWITFNTYNMLPGQINDPTKVFHFERRELPIELCGLLQADLSTIALVGLESGNISFLTSLLKRMSPPQLVKESVQAQ

CQNIESWDLNSLSESEIMKLKILRYVVLDSEVREDSTMGETSDMRSRSLITPRKFTTPASLERLVSYKDFQEILADPTRT

EDLLETMINNPELLVTKGENSEELMTTILYRYNSKKFKESLSIQSPTQLFIEQILFANKPVIDYTGIQDRYLSILDVPRV

QENEGIIGRKTIPETFVAIKRDLSLMNLDHKDIKLVYSFCILNDPLNTTACNAILLSQVQSLMDRSSLSAVTMPEFRNMK

LIRYSPALVLRAYIHNNLTIAGANENAMRRDLFHLQEFINQTKIKERLDKRIQDNEEIKGERDRMFEIKETTKFYQACYD

YIKSTEHRVKVFILPMKAYTAFDFCATIHGNLMKDKGWFAVHYLKQIVSGTAKASVSQAPASEMIIVGECFRLIAHFCDT

FIDVGSRLQFLYNIIDNFTYKNIPVKNLLEIMMRTNKRQHFLPILHWLEELTQHDIDKYDAYKANERVSWNNW

>Mermet_virus_(from_unknown)_(ATI21267)

MDPEKVEEYRNKIENCRDPEKAQEIWRALLVERHNYFSREFCDAAGWEFRNDIPSEDICSEVLPNHIAKGVKYCTPDNYY

IDGNGKIYIIDFKVAVDEKSARDTREKYNEIFGAIFNANGIDYEIVIIKHNPATGMTMIDSDNLRREIGQINLTVDLSWF

FELKEFLFGKFKDDERFLEIINQGDFTMTMPWLEEDTPELFEHPKFREFLESMPEEERDTFFQSLNFRSDDAGKWNDCLQ

DIMEQYKGRYQSFVKRSAQEIFNTTGDYPKPTKAAIDEGWADMVERVKEERNVTTDMAKQKPSFHFIWSPNSDESNANIP

KILKLAKKLQSITGVGTFVKAFKSMGKLMDFSNDVAGYERFCNKLKADARSTPKRLDKKIEEFTAGTCTALWEQQFKLDT

SVMAKEDRIHLMKDFFGIGNHKSFAKRLNEDLDLEKPTILDFNNPDVVRKCQAQYSNVEQILGKVSNLDRIGNYLEHYSP

KIQGASAEMWDTVFKISRTQFWQCINDYSTLMKNMLAVSQYNKHNTFRVVTCANNNVFGIVMPSSDIKTKKATLVYCVVT

IHSNEEDVAHLGSLYGTFRSSNCFVSISKAFRLDKERCQRIVSSPGLFMMTACLFIGDNYTLHLDNLLNFSFHTSVSITK

AMLSLTEPSRYMMMNSLAMSSHVKEYIAEKFAPYTKTAFSVVMTDLIKKGCYNAFNQREKVQLRDIHMTDYDVTQKGVQK

KRDLRSIWFPGKVDLKEYINQIYMPFYFNSKGLHEKHHVLIDLAKTILEIEKDQRENLPKPWSEHPKKQTVNLDILIYAI

AKNLNLDTSRHNFVRSRVENANNLKRSITTIATFTSSKSCIKRGDFTEFKTRIQKKVEKNIKKEVKKVTIANPALIDEIT

NDMEIHHATYLDIKRAVPEYIDFMSTKVFDRLYELLKINKWGTERTIDVIFKAMKEHTEFVFAFFNKGQKTAKDREIFLG

EFEAKLCLYLLERIAKERCKLNPDEMISEPGDSKLRILEKQAEEEIRFLARTIKNVNKELLDKLKVGAWGGEFSLDDLGE

DKAHGMKLEINADMSKWSAQDVIYKYFWLVAMDPILYPDEKKRILFFLCNYMQKKLLLPDELMQTILDQRVPRYNDIIGQ

MTEGYRRNWVVIERNWLQGNMNYTSSYLHSCSMSVFKDIMRECASLLEGEVLVNSLVHSDDNQTSICIVQNKLPDDNITE

FVTKLFEFICLTFGNQANMKKTYITNFIKEFVSLFNIHGEPFSVYGRFLLTAVGDCAYLGPYEDLASRLSATQTAIKHGC

PASLAWISIALNHWITHTTYNMLPNQINDPLPFFPTNNREEIPIEMCGLLNSELQTIALVGLEAGNLSYLTSLLRRMSPV

TYQREPVQSQCSQIKTWDLSLLTAMDIIKLKALRYIALDSEVTTDDGMGETSEMRSRSLLTPRKFTTSGSLNRLISYNDF

QKIISDENERDRMFEFFIRRPELLVTKGENAREYMNSVVFRYNSKKFKESLSIQNPSQLFIEQILFSNKPIIDYTSIHDK

IFGLQDHPDLEEMDTIIGKKTFVQSYVQIIDDLDKFKLSHEDIETVYSYCLLNDPLLITASNNIVMSVKGADQERLGQSA

CRMPEMRSLKLIYHSPALVLRAYVNQNPEIQGADPDEMLRDLTHLEEFIEKTKLRTHMRDRIELNQKKMMRRDIQFEVRE

LTRFYQVLYDYIKSTEHKIKVFILPYKVYTPIDFCAALTGNLIKDDKWCIVHFLKNIISTTHKAQIAICPDLEIQLAMEC

FRLIAHFGDMFLAEKSRVPFLRTIIQNFSYKNVPVVRLYEKLRESKHRTKFIPILFHMDDLTQRDLNKYDADKSDERITW

NNWQVSREMNTGPIDLIITGYDRQIQIRGEDDRLLGAELRLARVSRETISGHAKSILNRKHGLRFEKMSKIEEMSDKLDY

IVRGRNRYFYNILPKQIILDHNSRVDKTKSIGESKWIPVCPVVVKKLYQQKRPDRNKIFNLNMQTYTLSKLQVNPNEYAT

VRKAHFQKMTFFNGPPIKSGGMDIAALMSSPSLLSLNYDTMSQASLIDMCRVFKCEGFKEDQMAFEFLSDEIMQVDIGEQ

LECNPIFSIVYNTKGEKGMTYKSAFRTALIRECEKFERAFDFLDMGFCSNENLSILEEIHWIVIELKTNQWSTELDQCIH

MCMYRNNLDPEFHQFDIPKAFLDDPIKRTIRWKKVKKFIEVLRDFEVREEPWKSIVQHFCDKASRLTIEKIRETKKSTGF

EDFVDPDKRGGRSKFDFV

>Enseada_virus_(from_unknown)_(AMR98952)

MATPINDLVRQFTARIRTCRTAEIGRDILNEITMARHNYFAYEFCNAINIEYRNDVPALDIVLEMIPDFDPNTVKIPNLT

PDNYFRDGNKIYVIDFKVSVSDESSEYTYKKYNTLLGEVFNHLGIPYEIVIIRIDPSNMRLHISSDDFMRLFPNIILDVH

FDWYFNLKEQLFERFKDDEEFMALTAYGEFTPTIPWVDEPTDELWDHPIFKEFMLSMPEHQQEEFLNAMDFNSFTADKWN

DLLHTTMRKYKGIYDEFIRNQSKKLFQMDENFKKPTREEILLGWNQMVERIRSAREVTPDLSKQKPSIHFIWSPNDPKQS

NENIQKILRLSKLLKRIDDRDVNSQAFKYIGELMDFSEDIEQYTNFCSRLKAQARSSLKPKDTKITPIQIGNSTVMWEQQ

FKFDTNVIPKEIRLKFLKEFCGIGNHKLFEDRMLEDLDLEKPRILDFTNKDVLNAAHHMVVGTKQFLKEENNLDKIGNML

EEYKDKIRNANEITWDNVEKICKSRFWQAINDFSVIIKNMLSVSQYNKHNTFRVVCSANNNFYGLLYPAASIKSKRSTIV

FSTITIHNDEKDVLNCGALYKTYKIGNVYVSISKAIRLDKERCQRLVTSPGLFMLTSLMFKGDNDIDLNDIMSFSFFTSL

SITKSMLSLTEPSRYMIMNSLALSSHVREYIAEKFSPYTKTLFSVYMTELIRRGCMSANNQKDLISLKNVFLNEFEITQK

GVSNDRDLQSIWFPGKVNLKEYINQVYLPFYFNAKGLHNKHHVMIDLAKTVLEIELEQRQEVPSPWSKTMDKQSANLDIL

IFSIAKMLNMDTSKNNHLRSRIENRNNFKRSLTSISTFTSSKSCIKIGDFFEYKSKTRKIVESIKMKESKKTRIANTEFV

DEDDRDNVIAHSTYVDLIKSVPRYTDYISTKVFDRLYEMYKTDEIEDKPAIEIIMDTMRNHKDFKFCFFNKGQKTAKDRE

IFVGEFEAKLCLYGLERISKERCKLNPEEMISEPGDGKLKKLELNAESEIRYLIEATRGNDRIKEQLDDLLDQVKGIKLE

INADMSKWSAQDVFFKYFWLVVLDPILYPYEKQRIIYFLCNYMNKELILPDEMMCSLLDQKAQRANDIIREMTNNFQTNC

VNIKRNWLQGNLNYTSSYIHSCSMMVFKDIVKDSANLLEGRCNVNSLVHSDDNQTSVIFIQSKLHNDFIINFICQTFENC

CRTFGNQANMKKTYITNHIKEFVSLFNIYGEPFSIYGRFLLPAVGDCAYIGPYEDMASRLSATQTAIKHGCPPSLAWVSI

ALNFWITFSTYNMLPGQQNDPTPIFNCKRNELPIELCGTLESDLSTVALIGLESGNISFLTKILKKMSKPQLVKESVQTQ

CNHIMEWDIDNLTDMELMRFKILRYIVLDTEISEDNKMGETSEMRSRSLITPRKFTTTSSLEKLISYKDFQATIVNKESI

DDLFQYLIDRPELLVTKGETSEDYMKTILFRYNSKKFKESLSIQSPTQLFIEQILFSNKPTIDYSGIQDRFTNILDMPKI

QQGDNIIGRKTIPEAFKSIKEDLSKITLTLEDINLVYTFCILNDPLNITACNAILLSQIQSLMEKTSLSSVTMPEFRNMK

LIRYSPALVLRAYIHNDFTIGHANENMLRRDVFHLHEFIKETRMLERMQVKIIENEELKGERDLLFEIKELTKFYQTCYD

YVKSTEHKVKVFILPMKVYTAFDFCATIHGNLIKDKGWYSVHYLKQIVSGTSKAIVSRTPASEQVVVDECFKLLSHFCDT

FIAEDSRIPFLKKIFKDFTYKGIKVADLYNTLKKSHKKQHFLPLLFRTGDLEQGDLDRYDAAKSYERVSWNDWQINRNLN

TGAIDLTIKGYQRALRIIGEDDELKLAELEITKGDNTPIETHGRKLLNTKHNLRFEKMMPVPVMKPNTYYICYQKKTSNS

YIYLILSSALIESRNSMHVSLLENKPTKLTPVCPVVVAQVDSQERINLYQLKFLNPECEVSRMKVTMNEFITIRRSHFSK

MIFFDGPELVVGNINITKLIQTPALLSKNYNSLSQIPLITLSKIFSCTGEQQQQDEFEFLSDEIMEDEESEQINTVPIFT

ITYTKKSKQGHTYKKCLQEALRRGLQEMESEFNFNKDGFFSPKNIGIISLIVSLIDQLHTNEWSSIVRECIHMAYFNNGK

DVLFHNFRIPKAFLKEAIGEKPNWEKIKVFLDGLHATNRDSYWDQLLEQFKEKCRRMIDEQIKIESQNWGDILDEIDDYQ

NIAAYTFD

>Keystone_virus_(from_unknown)_(AWK60687)

MDQNEYQQFLARINSAKDACVAKDIDVDLLMARHDYFGRELCKSLNIEYRNDVPFVDIVLDIKPEIDPLSLEIPHITPDN

YLYVNNILYIIDYKVSVSNESSIITNTKYFELTRDISDKLNIPIEVVVIRIDPISRELYISSDRFKDMFPTLVVDINFNQ

FFDLKQMLYEKFGEDEEFLLKVAHGDFTLTAPWCKTGCPMVWQHPIYKEFKMSMPIPERRLFEESMRFNAYESERWNTNL

IKVKEYTKKDYAEFITKSAKNVFLATGDFKQPNKSEINEGWAIMVERIKVQREMSNSIHDQKPSIHFIWSPNNAMNSNNA

TFKLVLLSKSLQSIKGTSTYTEAFKSLGRMMDIGDKFTDYESHCESLKSKARSSWKQVMNKKLEPKKINNALVLWEQQFM

VNSEIINKTDKIKLFRDFCGIGKHKQFKNKMLDDIDTSKPKILDFNDDAIYMASLTMMEQTKLLLSEKSNLKPNNYILDE

FGQRIKDCNRDTYEIMYYIFETKFWQCISDFSTLMKNILSVSQYNRHNTFRIAMCANNNVFALVYPSADIKTKKATVVYS

IIVLHDNEASIFNPGCLHGTFKCNSGFISISRAIRLDKERCQRIVSSPGLFLTTCLLFKHENPTIRLDDIMTFSLFTSLS

ITKSVLSLTEPARYMIMNSLAISSNVKDYIAEKFSPYTKTLFSVYMTRLIKNACFDAYNQRQKVQLRDIYLSDYDITQKG

IKDNRELTSIWFPGSVTLKEYLTQIYLPFYFNAKGLHEKHHVMVDLAKTILEIEKDQRENISLIWSKNCVKQTVNLQILI

HSICKNLLADTSRHNHLRNRIENRNNFRRSITTISTFTSSKSCIKIGDFKKEKEAQYTKQKRTLDIESRKRRLANPLFVN

DEEVSLEVGHCNYSMLREAMPNYKDYMSTKVFDRLYELLDTGKLDDRPTIESIMDMMVDHTDFYFTFFNKGQKTSKDREI

FVGEYEAKMCMYAVERIAKERCKLNPDEMISEPGDGKLKVLEQKSEQEIRFLVETTRQKNREIDDVIETLAAEDFQGNLN

KIEKIAKGKARGLKMEINADMSKWSAQDVFFKYFWLIALDPILYPQEKERILFFLCNYMQKKLILPDDLIYNLMDQKISY

KEDIISEMTDQLNANHIQIKRNWLQGNFNYTSSYVHSCAMSVYKDILKEAMSFLEGSIMVNSLVHSDDNQTSVTIVQDKV

PDEVLIEFSIKEFEKVCLTFGCQANMKKTYVTNCIKEFVSLFNLYGEPFSIYGRFLLTSVGDCAYIGPYEDLASRISSAQ

TAIKHGCPPSLAWVSIAISHWMTSLTYNMLPGQSNDPLDYFPAENRKEIPIELNGVIDAPLSMISTVGLEAGNLYFLIKL

LNKYTPVMQKRESVVNQIAEIRNWDIQKLDENEIFRLKILRYLVLDAEMDPSDIMGETSDMRGRSIFTPRKFTTAGSLRK

LYSFSKYQDRLSSPGGMNDLFAYLLQKPELLVTKGEDKKDYMESVIFRYNSKRFKESLSIQNPAQLFIEQILFSHKPIID

FSGIRDKYVNLHDSRALENEPDILGKITFTDAYRLLMKDLSSLPLTNDDLQVVFSYVILNDPLMITIANTHILSIYGSPQ

KRTGMSCSTMPEFRNLKLIHHSPALVLRAYSKGTPDVQGADPTEMARDLVHLKEFIDNTNLEEKMKRRIIQNEIDKGAKD

IVFELKEMTRFYQVCYEYIKSTEHKIKVFILPAKAYTTTDFCSLMQGNLIKDREWYTVHYLKQILSGGHKAIMQHSATSE

QNIAFECFRLITHFADSFIDSGSRTAFLQLILDGFSYKDVKVSKLYEIIKNGHNRTDFIPLLFRTGDLTQYDLDKYDAMK

SQERVTWNDWQTSRHLDTGSINLTISGYNRSITIIGEDNKLTYSELNITRKTPENISISGRKLLSSRHGLRFENMAKIIT

YPGSYYITYRKKDRHQYVYQIHTHESIIRRNEEHLAIKTRIFNEIVPVCLVNIAEVDGDQRILIRSLDYLNNDVFSLSRI

KVGLEEFAVIKKAHFSKMVSFEGPPIKTGLINLTELMKSQDLLNLNYDNIRNSNLISFSKIICCEGSENIDDGLEFLSDD

PMAFTEGEVIHSTPLFNIYYSKKGERHMTYRNAIKLLIDRETTNFEEAFTFHSDGFLSPENLGCLEAIISLIKILKTNEW

SSVIDKCIHICLIKNNMDHLYHRFDTPICFYDNPISRNINWMMYRDFIATLPQTYVAPWNIMLEHFRTKCMTLILDKMEI

QRNFSDFAKMMRRSEGRTNLDFD

>Guama_virus_(from_unknown)_(AKO90160)

MAHQLGEIVRQFAARVRTCNNPELGRDLLSDITVARHNYFAQEFCYSIGIEYRNDVPALDIVMEMVPDFNPAQIRVPNIT

PDNYYRDGNKIYIIDFKVSVSDESGNHTFKKYNTLFGDIFDQLGVEYEVVIIRMDPSSMHLHISSDNFANIFPNVILNLD

FSWYFRLKDELYERFRDNEEFMELVAHGEFTPTIPWVNEETAELFDHPVFVDFMNSMGPEARNDFFQALNYNAFQADKWN

DLLHIFIRKYSMKYKDFLKEMSRKVFMADENYNKPSRLEIQKGWAEMIERVRDTRNMVTDIHKQKPSIHFIWSPHDSTKP

QENNAKILELSGALQRIKDTDPMSTAFKYIGKLMDFSSDINAYEQFCSKLKRDARSTPKQKSTPINPIKIGPCTVLWEQQ

FKMDTDVIPKEIRLRFLKEFCGIGNHKQFKNRMLDDLDLEKPQILDFSDLNIQRNANLMFQDTKSMMSKTSGLQKIGNVL

EEFRDKIEGANEKTWSHIEYIAQSRFWQAINDFSVIVKNILSVSQYNKHNTFRVVASANNRFFGLVYPSASIQSKKSTVV

FSTICLHKDSKDVLKCGALYKTYKIGDQYISISKAIRLDKERCQRLVTAPGIFMLTTLLLKGDTEIDLDEIMAFSFFTSL

SITKSMLSLTEPSRYMIMNSLAVSSHVREYIAEKFSPYTKTLFSVYMTELIHKGCMSANNQRSKISVKNVFLNEYEITQK

GVSEDRDLESIWFPGHVNLKEYINQVYLPFYFNAKGLHNKHHVMIDLAKTVLEIELEQREQLPNPWGKDFQKQSVNLEIL

IYSIAKMLNNDTSKHNHLRSRIENRNNFKRSLASISTFTSSKSCIKVGDFSEHKTKTVKRLKKIQEKDAKKTRIANTEFV

AEEDRDLEIAHSTYLDLVKSVPEYTDYISTKVFDRLYEKFKTGEFEDKPAIEIIMDVMKGHKDFKFCFFNKGQKTAKDRE

IFVGELEAKLCLYCVERIAKERCKLNPEEMISEPGDGKLKKLEINSESELRYLIEMTRREMSKEEEFVETFSKEPKGVKI

EINADMSKWSAQDVFFKYFWLIVLDPILYPYEKQRILYFFCNYMDKELILPDEMMCSLLDQKAERENDIIRQMTNNFHTN

TVNIRRNWLQGNLNYTSSYIHSCSMMVFRDIVKDATELLEGNCNVNSMVHSDDNQTSVIIIQDKLSNDCIIHYICDLFEA

CCLTFGNQANMKKTYITNNIKEFVSLFNIYGEPFSVFGRFLLPAVGDCAYIGPYEDMASRLSATQTAIKHGCPPSLAWVS

IALNHWITFNTYNMLPGQVNDPTRIFYCDRDELPIELCGILKADLSTIALIGLEAGNISFLTNLLRRMSPPQLIKESVQS

QCNNIEFWDLTKLTRMEIVKLKILRYIVLDTDITGDNTMGETSDMRSRSIITPRKFTTLSSLTRLVSYNDYQEITQSKEE

FDNLLEDMLEHQELLVTKGECPEDFIKTILFRYNSKKFKESLSIQSPTQLFVEQILFSNKPIIDYSGIRDKYIGLLDMPQ

VQESDTIIGKKTIPEAFETLSKDLDKFELDVDDVKLVYSFCILNDPLNTTACNAILLSQVQSLMDRTSMSAVTMPEFRNM

KLIRYSPALVLRAYLHGTYNLGGATEEGMKRDLYHLEEFINQTGILQKVEDRIREHETRIAERDLLFEIRERTKFLQTCY

DYIKSTEHRVKVFILPCKAYTAFDFCATIHGNLIKDKGWYSVHYLKQIVSGTAKAIVSQNPASEQINMDECFKLLAHFSD

TFIEPSSRLKFARRIIEEYTYKNIPVKKLYDMLVKDPNKRQHFMPLLYHLNELKQSDLDKFDANKTNERVSWNDWQVNRD

MSTGIIDLTIKGYMRSIRIKGEDDRLEIAELQLVIGDNTSVESHGRKLLNTRHNLKLERMQIHKFLEQGMYYICWQRRNK

YTYTYQILLAEIIENRNRQPVSLISGRANLLNPVCPVIVSRSPSMEKIRLTALRYLNPEAELSKLKITQNEFATTRRCHF

SKMPFFYGGDFTVGNININKLMSTPSLLNVNYASLCQTPLITLAQIFQCDGQTEEVDEFEFLSDETLEEIDTAPVNAIPF

FNVSFPTRSKKGYTYKQALQQALSAGLEEMEREFDFTGQGYFSPKNIGVISFMIGLVNRLNTNEWSTVLVKCIHMSFFNN

KKDRVFHLFKIPKVFIKDPIGEIIDWNKAREFLMGIRPKDETNHWGQMFSHFKQKCLNAIDLEIKMEGTSWGDMLDMIDE

FKDEEMFHFE

>Tapirape_virus_(from_unknown)_(AIN55747)

MDQNSIDQFIRRIQTAPNAEVALIIDIDLFEARHDYFGKELCNSLGIVYRNDVPGEEIITESIPEIDPDSIRAKCTPDNY

VIRDGITFIIDYKVIVNDEVLINTFDKYTSIFEPYFKDYNRQFEVVIIACNPYTGMIKHTGVLFDNYFHIDGSDINFDWF

FMLRDMLCERFKDDEVFIANAGPGGEFSMTSPWFDQMPNLMQDHEYRAFLGTLPPRERELFIKAMDYDPFKADKWSDFLV

QAKETYKKEYDEYVKETARDIFSISKILEKPSHEEIRAGWKEMYDRVQTERTLLKDPAKQKPSIHCIWSPPDEKRSNDQV

SKLLLFSQKMRKIKSGEQFAEVFRKIGENFDISSDIEQYVTFCTNVKNESRLMEKERKSKKIQPVFIGTSKVLWEQQFKM

DLDPIDKKRQAMFKKDYLGIGKGKRFALRTAEDVEKDKPKILDFEDDTIIMAADEMMENTKGLLSRRNGLSKSGCFLDEY

KPQISSASKETWQTISSITSTHYWSMVNDYSILMKNILASSQYNKHNTFRVCFCANNSLMAIIFPSSDIKTKQATTCFVT

IALHNERDSVMNPGCMYKTYETAGGFVSISRPIRLDKERCQRIVTAPGLFLESCLLMYNNNPTVKLKDVLNFCLFTSLSI

TKPLLTLTEPSRYMIMDSLALTSSVKGYIGEKFNPLTKTLFSVYMARLIKTGCMDAFNQRQVIQPRRVALDDYDITQKGV

EETRSLKSIWFPGSVSLKEYINQIYLPFYFNAKGLHEKHHVMIDLAKTVLEIESEQRREAYFPWSESPKPQSVNLPILIY

GLSRQLHLDTQRKAHLRSKIENRNNFKRKIYTISTLTSSKSCIETGDFSKEKSIIKERNHNVANPIFEDEYEENVKVEKS

TYLDLKQKIPNYIDIRSTKVFDILYKKTKEQELGDREAISYMMESMMTKEDYVFSFFNKGQKTAKDREIFVGEYEAKMSL

YVIERIMKELCKSSPEEMISEPGDGKLKVLESMANQEIRDLIATSKRNQIESKTERDDLGKPSGSTPRDEFKKRPLKIDI

NADMSKWSAQDVTYKYFWLIALNPILYPEEKACIVKFFCRYMNKKLILPDAMLNSVFDQFKLYENDIIRNMTNDFSQNWV

QIRNNWFQGNLNYTSSYIHTVSMSTYKDIVKHAMALLEGNATITSLVHSDDNHTSIIANQGKLDDDSIIRFCYDAFITVC

LSHGNQVNKKKTYVTNGLKEFVSLFNIFGEPFSVYGRFLLTSVGDCAYLGPYEDLSSRISSVQTAIKHGCPPSYAWVAIA

MSQWLSYSTYNMLPGQYNDPCEALLISDRFQIPVELGGLISCPLHVLVLLGLDSINVYNLYKILKSIVDVPIRGHRIEDL

MLSSQNWDLSKLKDSQVFQLKVLRYITLGVEIDSAQKMGETSDMRNRSVLTPRKFTTRRSLIRLESYNDYRLMLQHQVDY

DLNLQYMLDHPELLVTKGECSEDYMNTILFRYNSKRFKESLSIQSPSQLFIEQILFSKKPTIDYTRISDKFTPTAERDDE

QIMGKKTIKESMESIRRDLELYTIQVQDVETVFNCVVVNDPLAVTAINTEILHILTEKKPRNGLTCNTMPEFRNIKLINY

SPAVVIRTYVRPEFCPSNADPRLLERDVWFLTEFIKETGIKERAEESIRLNEAAKGVRDMVFEIQEWTRFYQSCYSYIKA

TEHKVKMFIIPNRATTATQFCQAIIGNTRKDDSYFGCYFQKNAIGYNQKGLISKTFDMTTYTADECFRLLCHFTDQVVQQ

EHRLFFINKIINEYKFRGNPVRHLLDRLLSSQRRTLFLPLLLRLDELKEYDLLQFNAEMGQKNVAWNNWQINRALNTGPV

DLTLSTSSARLRILGQDSKLEYARLTMQRVTNTNIRSTGRSLLGARHGLPFELFESCEIEDRSWYICSQKRHAKRIFYDV

KRGFEIRNDNASMLLDGRRSGQIVAHCELEICEDATNTSLDFESVVELNKYELSFSRMIIAENTYATVRRVDLSKMQDFE

GPMIISNRLNIYKLMRSNTLLSCNYDNVISSSLIELADVIECSGTDTEFSFDFLNDEPMEADEFESIDASPELSIKYGKK

GQRYMTLQNAFYEIIATKSILFKSTLTFAGGEFFSRENMAIWTNLIGLNNVLNIGGEFDQLIRVAHLIFAQSGHDAVFHL

SEISPEFYSGRQINYKHLNILINTLKCSGDNVWSFLFNKASIALGHELRKRDLDESIDVESLIRRIGQRMPNMSEFNFQD

>Itaqui_virus_(from_unknown)_(AVX48954)

MAILLEDVIRQYSARIRNCNNPEIGRDILAEITMTRHNYFAQKFCEAIGIEYRNDVPAADIVLEMMPGLDLTRIRIPNIT

PDNYYRDGTKIYIIDFKVSVSDESAQHTYKKYDTLFGDVFNQLNIEYEVVIIRMNPSDMHLHISSDNFAALFPNIALNVT

FDWYFRLRDDLFHQFRDNEEFMELIAHGEFTPTIPWVNENTPELFDHEVFQDFIASMPLENREDFYYALNHNAFQSDKWN

DLLHVIMRKYGDKYHDFIKANARRIFLTDDKYNRPTKVAILQGWKEMVERVQEGRETVDDLSKQKPSIHFIWGPNSKNES

NENNTKIIRLSKTLQSIKEKDSFSVAFKSIGSLMDFSEDIDKYEAFCAKLKADARSSLKPKSKKIDPIKIGPNLIKVGQQ

FKMDTDIIPKEIRLKFLKEFCGIGNHKQFKDRMMEDLDLDKPKILNFENQDIKNQAYAMVQNTSYFMSKPSNLIKVGNVL

EEFKDKIVNANEETWATIEEIAKTRYWQSINDFSVLIKNILAVSQYNKHNTFRIVCTANNNFFGLVYPSTSIQSKRSTIV

FSTIVLHDNESDVLKCGALYKTYRVKNGYLSISKAIRLDKERCQRLVISPGLFMLTSLLFKGDCDVSLNDVMNFAFFTSL

SITKSMLSLTEPSRYMIMNSLALSSHVREYIAEKFSPYTKTLFAVYMTDLIKRGCMAANDQRQQISIKDVFLNEFEITQK

GVTNDRNLQSIWFPGKVNLKEYINQIYMPFYFNAKGLHNKHHVMIDLAKTVLEIELDQRVNLPTPWGVDLKKQSVNLDVL

IFSIAKMLNLDTSRHNHLRSRVENRNNFKRSLASISTFTSSKSCIKVGDFKEFKERNALHMKKLQGKEAKKTRIANTEFV

SDNDRDLEIVHSTYLDLRKSVPNYTDYMSTKVFDRLYERFKNHDFEDKPAIEIIMDVMRCHTDFKFCFFNKGQKTAKDRE

IFVGELEAKLCLYGVERIAKERCKLNPDEMISEPGDGKLKKLEINGESEIRYLIDATRNQTREQSKVDDLLDTPKGIKLE

INADMSKWSAQDVFYKYFWLIALDPILYPFEKQRIIFFFCNYMNKELILPDEMMCSLLDQKAQRENDLIRQMTNGFTTNT

VNIRRNWLQGNLNYTSSYIHSCSMMVFKDILKEVSALLEGRCNVNSMVHSDDNQTSVIMVQDKLHNDIITNFVCNTFERC

CLTFGNQANMKKTYITNHIKEFVSLFNIYGEPYSVYGRFLLPAVGDCAYIGPYEDMASRLSATQTAIKHGCPPSLAWVSI

ALNHWITFNTYNMLPGQINDPTKVFHFERRELPIELCGLLQADLSTIALVGLESGNISFLTSLLKKMSPPQLVKESGQAQ

CQNIESWDLDLLSESEIMKLKILRYVVLDSEVREDSTMGETSDMRSRSLITPRKFTTPASLERLISYKDFQEILADPTRT

EDLLETMINNPELLVTKGENSEEFMTTILYRYNSKKFKESLSIQSPTQLFIEQILFANKPVIDYTGIQDRYLSILDVPRV

QENEGIIGRKTIPETFVAIKRDLSLMNLDHKDIKLVYSFCILNDPLNTTACNAILLSQVQSLMDRSSLSAVTMPEFRNMK

LIRYSPALVLRAYIHNNLTIAGANENAMRRDLFHLQEFINQTKIKERLDKRIQDNEEIRGERDRMFEIKETTKFYQACYD

YIKSTEHRVKVFILPMKAYTAFDFCATIHGNLMKDKGWFAVHYLKQIVSGTAKASVSQAPASEMIIVGECFRLIAHFCDT

FIDVGSRLQFLYNIIDNFTYKNMPVKNLLVLIIISSKRQHFLPILHWLEELTQHDIDKYDAYKANERVVWNDWQVNRDMN

TGNIDLTIKGYQRTLRVIGEDDTLKIGELEILKGDTTPIETHGRKLLNSKHGLKFEKMQKYKIIEPNTYYICWQNRTRFS

YTYQLLLSNIIEARNSQTVSVTGGKFNELVPVCPVIVSRIDSDDKMNLRQIKYLNMDCSLTRLQLNQNEFAVVKRCHFSK

MVFFNGPEMIVGNINITNLIQTPSLLTTNYPSLSQMPMMTLTRIFNCNGEEKEVDEFEFLSDEILEETETAVINAQPMFN

IQYETKSKKGYTYKRALQEALSRGIQEIEHNFDFCKDGFYSPKNIAIIALLVNVIDRLHTNEWSSIIRKSFHMCFFNNGK

DKLFHMMNIPKTFIKNPIGEVPNWEKIRTFIIQIDTAYPGNNWHQMFEHFKEKCILLIDREIKMEGMSWGEMLDELDDYK

DTEMFHFN

>Ananindeua_virus_(from_unknown)_(APM83095)

MAHQLGALVRQFAARIRACHSPELGRDLLSDITVARHNYFAQEFCYSIGLEYRNDVPALDIVMEMVPDFDPRAIRVPNIT

PDNYYRDGDKIYIIDFKVSVSDESGNHTYKKYNTLFGDIFDQLGIEYEVVIIRMDPSSMHLHISSDNFLNVFPNVILNLD

FSWYFRLKDELFERFRDNEEFMELVAHGEFTPTIPWVNEETPELFCHPVFTEFLDSLGYEVRNDFFQALNYNAFQADKWN

DLLHIIIRKYGVQYRDFLKTMSHKIFIADSDFNKPSRLEIQKGWSEMIDRVRQTRDVTTDIHKQKPSIHFIWSPHDSTKP

CENNPKILELARSLQEIKENDPISSAFRYIGKLMDFSGDIGKYEEFCIKLKKDARSTPKQKTNPIEPIVIGDCTILWEQQ

FKMDTQVIPKDIRLKFLKEFCGIGNHKQFKNRMLEDLDLEKPRILDFSDINVQRTANLMFQDTKSMMAKPSGLHRVGNIL

EEFRDKIEGANEKTWENIETIGKTRFWQAINDFSIIIKNMLSVSQYNRHNTFRVVTSANNRFFGLVYPSASIKSKKSTIV

FSTISIHKDQRDVLKCGALYKTYKIDGQYISISKAIRLDKERCQRLVTSPGIFMLTSLLLKGETEIDLIEIMAFSFFTSL

SITKSMLSLTEPSRYMIMNSLAVSSHVREYISEKFSPYTKTLFSVYMTELIHKGCMSANNQRSKISVKDVFLNEYEITQK

GVSEERDLESIWFPGHVNLKEYINQVYLPFYFNAKGLHNKHHVMIDLAKTVLEIELEQREQLPNPWGTDFEKQSVNLHVL

IYAIAKMLNNDTSKHNHLRSRIENRNNFKRSLASISTFTSSKSCIKIGDFSEHKTKTAMRIRKIQEKDAKKTRIANTEFV

AEEDRDLEVAHSTYIDLVSSIPQYTDYISTKVFDRLYEKFKTEEYGDVPAVEIMMDVMRSHKNFKFCFFNKGQKTAKDRE

IFVGELEAKLCLYCVERIAKERCKLNPEEMISEPGDGKLKKLEVNSEHELRYLIEMTRKEMSKEEEYVSIITKEPKGIKI

EINADMSKWSAQDVFFKYFWLIALDPILYPYEKQRILYFFCNYMDKELILPDEMMCSLLDQKAARENDIIRQMTNNFHTN

TVNIRRNWLQGNLNYTSSYIHSCSMMVFRDIVKDAVELLEGDCNVNSMVHSDDNQTSVIVIQEKLSNDCILHYICDLFEA

CCLTFGNQANMKKTYITNNIKEFVSLFNIYGEPFSVFGRFLLPAVGDCAYIGPYEDMASRLSSTQTAIKHGCPPSLAWVS

IALNHWITFNTYNMLPGQVNDPTRIFYCDREEIPIELCGILKADLSTIALVGLEAGNVSFLTTLLKKMSPPQLMKESVQS

QCHNIGSWDLSKLTKMEVIRLKILRYIVLDTDITEDNTMGETSDMRSRSIITPRKFTTISSLSRLVSYNDYQSIVQNKEE

FDQLLEEMLGHQELLVTKGECPEDFIKTILFRYNSKRFKESLSIQNPTQLFVEQILFSSKPTIDYSGIRDKYAGLLDMPQ

VQESETIMGKKTIPEVFELINKDLDMFDLTTDDIKLVYSFCILNDPLNTTACNAILLSQIQSLMDRTSMSAVTMPEFRNM

KLIRYSPALVLRAYLHGDHTIGGATEEGMKRDLYHLEEFINQTGILEKVEERIRDHEQRLAKRDLLFEIKERTKFLQTCY

DYIKSTEHRVKVFILPSKVYTAFDFCATIHGNLIKDKGWYSVHYLKQIVSGSAKAIVNQSPASEQINMDECFKLIAHFSD

TFIEPSSRLNFAKRVINDYTYKNIPVKKLYEMLIKDLNKRQQFIPLLYHMGELNQSDLDKLDANKTNERVSWNDWQVNRD

MNTGIIDLTIKGYMRSIRIKGEDNKLEIAELQLIVGDNTPVESHGRKLLNTRHNLKLEKMQTHKYLEQGMYYICWQRKNK

YTYTYQILLAEIIENRNKQPASLVSGKINILSPVCPVIVSRIPSMERIRITSLRYLNQVCELSKLRIAQNEYAIVRRCHF

SKMPFFYGSEFTVGNIDINKLMSTPSLLNVNYASLCQTPLITLAQIFRCNGLTEEVDEFEFLSDETLEEVDSAPVNAIPF

FNVNFPTKSKKGFTYKQALQQALSSGLNEMEQEFDFTGTGYFSPKNIGVISFMVSLVNRLNTNEWSTVLIKCIHMSFFNN

KKDRTYHLFKIPRVFINDPIGEKIDWNKAREFLIGIKPRDEGNHWGQMFNHFKTKCLHAIDLETRMEGASWGDMLEMIDE

FKDEEMFHFE

>Bimiti_virus_(from_unknown)_(AKO90157)

MAHQLGEIVRQFAARVRTCNNPELGRDLLSDITVARHNYFAQEFCYSIGIEYRNDVPALDIVMEMVPDFNPAQIRVPNIT

PDNYYRDGNKIYIIDFKVSVSDESGNHTFKKYNTLFGDIFDQLGVEYEVVIIRMDPSSMHLHISSDNFANIFPNVILNLD

FSWYFRLKDELYERFRDNEEFMELVAHGEFTPTIPWVNEETAELFDHPVFVDFMNSMGPEARNDFFQALNYNAFQADKWN

DLLHMFIRKYSMKYKDFLKEMSRKVFMADEDYNKPSRLEIQKGWAEMIERVRDTRTMVTDIHKQKPSIHFIWSPHDSTKP

QDNNSKILELSKALQKIKDTDPMSTAFKYIGKLMDFSSDINAYEQFCSKLKRDARSTPRQKSAPIDPIKIGPCTALWEQQ

FKMDTDVIPKEIRLRFLKEFCGIGNHKQFKNRMLDDLDLEKPQILDFSDLNIQRNANLMFQDTKSMMSKTSGLQKIGNVL

EEFRDKIEGANEKTWSHIEYIAQSRFWQAVNDFSVIVKNILSVSQYNKHNTFRVVASANNRFFGLVYPSASIQSKKSTVV

FSTVCLHKDQRDVLKCGALYKTYKIGDQYISISKAIRLDKERCQRLVTAPGIFMLTTLLLKGDTEIDLDEIMAFSFFTSL

SITKSMLSLTEPSRYMIMNSLAVSSHVREYIAEKFSPYTKTLFSVYMTELIHKGCMSANNQRTKISVKDVFLNEYEITQK

GVSEDRDLESIWFPGHVNLKEYINQVYLPFYFNAKGLHNKHHVMIDLAKTVLEIELEQREQLPNPWGKDFQKQSVNLEIL

IYSIAKMLNNDTSKHNHLRSRVENRNNFKRSLASISTFTSSKSCIKVGDFSEHKTKTVKRLRKIQEKDAKKTRIANTEFV

AEEDRDLEIAHGTYLDLVKSVPEYTDYISTKVFDRLYEKFKTGEFEDKPTIEIIMDVMKEHKNFKFCFFNKGQKTAKDRE

IFVGELEAKLCLYCVERIAKERCKLNPEEMISEPGDGKLKKLEINSESELRYLIEMTRREMSKEEEFIETFTKEPKGVKI

EINADMSKWSAQDVFFKYFWLIVLDPILYPYEKQRILYFFCNYMDKELILPDEMMCSLLDQKAERENDIIRQMTNNFHTN

TVNIRRNWLQGNLNYTSSYIHSCSMMVFRDIVKDATELLEGNCNVNSMVHSDDNQTSVIIIQDKLSNDCILHYICDLFEA

CCLTFGNQANMKKTYITNNIKEFVSLFNIYGEPFSVFGRFLLPAVGDCAYIGPYEDMASRLSATQTAIKHGCPPSLAWVS

IALNHWITFNTYNMLPGQVNDPTRIFYCDRDELPIELCGILKADLSTIALIGLEAGNVSFLTSLLRKMSPPQLIKESVQS

QCNNIEFWDLTKLTRMEIIKLKILRYIVLDTDITGDNTMGETSDMRSRSIITPRKFTTLSSLSRLVSYNDYQEIAQSKEE

FDNLLEDMLKHQELLVTKGECPEDFIKTILFRYNSKKFKESLSIQSPTQLFVEQILFSNKPIIDYSGIRDKYIGLLDMPQ

VQESDTIIGKKTIPEAFETLSKDLDNFELDVDDVKLVYSFCILNDPLNTTACNAILLSQVQSLMDRTSMSAVTMPEFRNM

KLIRYSPALVLRAYLHGTYNLGGATEEGMKRDLYHLEEFINQTGILQKVEDRIREHETRIAERDLLFEIRERTKFLQTCY

DYIKSTEHRVKVFILPCKAYTAFDFCATIHGNLIKDKGWYSVHYLKQIVSGTAKAIVSQNPASEQINMDECFKLLAHFSD

TFIESSSRLKFARRIIDEYTYKNIPVKKLYDMLIKDPNKRQHFMPLLYHLNELKQSDLDKFDANKTNERVSWNDWQVNRD

MSTGIIDLTIKGYMRSIRIKGEDDRLEIAELQLVIGDNTSVESHGRKLLNTRHNLKLERMQIHKFLEQGMYYICWQRRNK

YTYTYQILLAEIIENRNRQPVSLISGRANLLNPVCPVIVSRSPSMEKIRLTALRYLNPEVELSKLKITQNEFATTRRCHF

SKMPFFYGSEFTVGNININKLMSTPSLLNVNYASLCQTPLITLAQIFQCDGQTEEEDEFEFLSDETLEEIDTAPVNAIPF

FNVSFPTRSKKGYTYKQALQQALSAGLEEMEKEFDFTGQGYFSPKNIGVISFMIGLVNRLNTNEWSTVLVKCIHMSFFNN

KKDRVFHLFKIPKVFIKDPIGEIIDWNKAREFLIGIKPKDETNHWGQMFNHFKQKCLNAIDLEIKMEGTSWGDMLDMIDE

FKDEEMFHFE

>Serra_do_Navio_virus_(from_unknown)_(APA28985)

MEQTEYQQFLARINSAKDACVAKDIDVDLLMSRHDYFGRELCKSLNIEYRNDVPFIDIILDIRPDIDPLSLDIPHITPDN

YLYINNILYIIDYKVSVSNESSIITNTKYFELTRDISDKLNIPIEIVIIRIDPISRELYISSDRFKDLFPTLVVDINFNQ

FFDLKQLLYEKFGEDEEFLLKVAHGDFTLTAPWCKTGCPLVWQHPVYKEFKMSMPIPERRLFEESMKFNAYEAERWNTNL

IKIREYTKQKYSTFVTKSAKHVFLATGDFKQPNKNEINEGWNLMVERIQDQREVSRSIHDQKPSIHFIWGSNNPNNSNNA

TFKLILLSKSLQSIKGSSTYTDAFRSLGKMMDIGDKLSEYESHCEQLKMQTRLSWKQVMNKKLEPKKINSALVLWEQQFM

INSEMINKSDKLKLFRDFCGIGKHKQFKNKMLDDIDTSKPKILDFNDEAIYMASLTMMEQTKILLSENSNLKPNNFILDE

FGQRIKDCNKDTFEIMLAIFETKFWQCISDFSTLMKNILSVSQYNRHNTFRIAMCANNNMFALVYPSADIKTKKATVVYS

IIVLHTQESNVFNPGCLHGTFKCNNGFISISKAIRLDKERCQRIVASPGLFLTTCLLFKHENPTIRLDDIMTFSIYTSLS

ITKSVLSLTEPARYMIMNSLAISSNVKDYISEKFSPYTKTLFSVYMTRLIKNACFDAYNQRQKVQLRDIYLSDYDITQKG

IKDNRELTSIWFPGSVTLKEYLTQIYLPFYFNAKGLHEKHHVMIDLAKTILEIEKDQRENITKIWSEDCTKQTVNLQILI

HSLCKNLLSDTSRHNHLRNRIENRNNFRRSITTISTFTSSKSCIKIGDFQKEKETQYSKQKKTIEMESRKRRLANPLFVT

DEEINLEVGHCNYSMLRDAMPEYKDYMSTKVFDRLYELLDTNVLDDKPTIELIMDMMAEHDKFYFTFFNKGQKTSKDREI

FVGEYEAKMCMYAVERIAKERCKLNPDEMISEPGDGKLKVLEQKSEQEIRFLVETTRQKNREIDEAIEALAAENFQENIG

KIEKISKGKARGLKMEINADMSKWSAQDVFYKYFWLIALDPILYPQEKERILFFLCNYMQKELILPDDLIYNLLDQKISY

KEDIISAMTNQLHTNSIQIKRNWLQGNFNYTSSYVHSCAMSVYKDILKEAMSYLEGSILVNSLVHSDDNQTSVTIVQDKL

PDEVLIEFCIKEFEMICLTFGCQANMKKTYVTNCIKEFVSLFNLYGEPFSIYGRFLLTSVGDCAYIGPYEDLASRISSAQ

TAIKHGCPPSLAWVSIAISHWMTALTYNMLPGQNNDPLDYFPADTRKDIPIELNGVIDAPLSMISTVGLEAGNLFFLIKL

LNKYTPVMQKRESVVNQITEIHSWDLNKLDDNEIFRLKILRYLVLDAEMDPSDIMGETSDMRGRSILTPRKFTTAGSLRK

LYSFSKYQDKLSAPGGMNDLFAYLLQKPELLVTKGEDKKDYMESVIFRYNSKRFKESLSIQNPAQLFIEQILFSHKPVID

FSGIRDKYINLHDSRVLEREPDIIGKVTFTDAYRILMKDLNTLPLTNEDLQVIYSYIILNDPLMITIANTHILSIYGSPQ

KRTGMSCSTMPEFRNLKLIHHSPALVLRAFSKGTPDVQGADPTEMARDLVHLKEFINNTNLEEKMKARIQQNELEKGSRD

VIFELKEMTRFYQVCYEYIKSTEHKIKVFILPAKAYTTTDFCSLMQGNLIKDREWYTVHYLKQILSGGHKAIMQHSATSE

QNIAFECFRLITHFGDSFIEASSRAAFLQLIIDGFSYKDVRVNKLYEIIKNGHNRTDFIPLLFRTGDLTQYDLDKYDAMK

SQERVTWNDWQTSRHLDMGAINLTISGYNRSITIIGEDNKLTYSELNITRKTPENISISGRKLLSSRHGLKFENMTKVTT

YPGNYYITYRKKDRHQYVYQIHTHESIIRRNEEHLAIKTRIFNEIVPVCLVNIAEVDGDQRILIRSLDYLNNDVFSLARI

KVGLDEYAVIKKAHFSKMVSFDGPPIKTGLINLTELMKSQDLLNLNYDNIRNSNLISFSKIICCEGSDNIDDGLEFLSDD

PMAFTEGETIHSTPIFNIYYSKKGEKHMTYRNAIKLLIERETANFEEAFTFYNDGFLSPENLGCLEAIVSLIRVLKTNEW

SSVIDKCIHICLIKNNMDYVYHRFDIPTCFYDNPISRNINWMMYRDFMATLPKTYIAPWNIMLEHFRTKCSTLIMDKMEV

HRNFSEFAKLMRRSEGRTNLDFD

>Itaya_virus_(from_unknown)_(AJZ73167)

MAILLEDVIRQYSARIRNCNNPEIGRDILAEITMTRHNYFAQRFCEAIGIEYRNDVPAADIVLEMVPGLDLTRIRIPNIT

PDNYYRDGTKIYIIDFKVSVSDESAQHTYKKYDTLFGDVFNQLNIEYEVVIVRMNPSDMHLHISSDNFSALFPNITLNVT

FDWYFRLRDDLFHQFRDNEEFMELIAHGEFTPTIPWVNENTPELFDHEVFQEFIASMPLENREDFYYALNHNAFQSDKWN

DLLHVIMRKYGDRYHDFIKANARRIFLTDDKYNRPTKDAILQGWKEMIERVQEQREIIDDLSKQKPSIHFIWGPNSKTES

NENNTKIIRLSKSLQSIREKDSFSVAFKNMGMLMDFSDDVDKYEAFCAKLKADARSSLRPKSKKIDPIKIGPSTILWEQQ

FKMDTDIIPKEIRLKFLKEFCGIGNHKQFKDRMMEDLDLGKPKILNFENQDIKNQAYAMMQNTSYFMSKPSNLTKVGNTL

EEFKDKIINANEDTWALIEDIAKTRYWQSINDFSVLIKNILAVSQYNKHNTFRIVCTANNNFFGIVYPSTSIQSKRSTIV

FSTIVLHENETDILKCGALYKTYRIKNGYLSISKAIRLDKERCQRLVISPGLFMLTSLLFKGDCDVSLNDVMNFAFFTSL

SITKSMLSLTEPSRYMIMNSLALSSHVREYIAEKFSPYTKTLFAVYMTDLIKRGCMAANDQRQQISIKDVFLNEFEITQK

GVTNDRNLQSIWFPGKVNLKEYINQIYMPFYFNAKGLHNKHHVMIDLAKTVLEIELDQRVNLPTPWGTDLKKQSVNLDVL

VFSIAKMLNLDTSRHNHLRSRVENRNNFKRSLASISTFTSSKSCIKVGDFKEFKEKSALHMKKLQGKEAKKTRIANTEFV

SDNDRDLEIVHSTYLDLRKSVPNYTDYMSTKVFDRLYERFKNHDFEDKPAIEIIMDVMRCHTDFKFCFFNKGQKTAKDRE

IFVGELEAKLCLYGVERIAKERCKLNPDEMISEPGDGKLKKLEINGESEIRYLIDATKNLTREQSKIDDILDTPKGIKLE

INADMSKWSAQDVFYKYFWLIALDPILYPFEKQRIIFFFCNYMNKELILPDEMMCSLLDQKAQRENDLIRQMTNGFTTNT

VNIRRNWLQGNLNYTSSYIHSCSMMVFKDILKEVSALLEGRCNVNSMVHSDDNQTSVIMVQDKLHNDVITNFVCSTFERC

CLTFGNQANMKKTYITNHIKEFVSLFNIYGEPYSVYGRFLLPAVGDCAYIGPYEDMASRLSATQTAIKHGCPPSLAWVSI

ALNHWITFNTYNMLPGQINDPTKVFHFERRELPIELCGLLQADLSTIALVGLESGNISFLTSLLKKMSPPQLVKESVQAQ

CQNIESWDLDLLSESEIMKLKILRYVVLDSEVREDSTMGETSDMRSRSLITPRKFTTPASLERLVSYKDFQEILADPVRT

EDLLDTMINNPELLVTKGENSNEFMTTILYRYNSKKFKESLSIQSPTQLFIEQILFANKPVIDYTGIQDRYLSILDVPRV

QENEGIIGRKTIPETFIAIKRDLSLMNLDHKDIKLVYSFCILNDPLNTTACNAILLSQVQSLMDRSSLSAVTMPEFRNMK

LIRYSPALVLRAYIHNNLTIAGANENAMKRDLFHLQEFINQTKIRERLDRRIQDNEEIKGERDRMFEIKEITKFYQACYD

YIKSTEHRVKVFILPMKAYTAFDFCATIHGNLMKDKGWFAVHYLKQIVSGTAKANVSQAPASEMIIVGECFRLIAHFCDT

FIDVGSRLQFLYNIIDNFTYKNIPVKKLLEIMMRTNKRQHFLPILHWLEELTQHDIDKYDAYKANERVVWNDWQVNRDMN

TGNIDLTIKGYQRTLRIIGEDDVLKIGELEILKGDTTPIETHGRKLLNSKHGLKFEKMQRYKIIEPNTYYICWQNRTRFS

YTYQLLLSNIIEARNSQTVSVTGGKFNELVPVCPVIVSRVDSDDKMNLRQIKYLNMDCSLTRLQLNQNEFAIVKRCHFSK

MVFFNGPEMIVGNINITNLIQTPSLLTTNYPSLSQMPMMTLTRIFNCNGEEKEVDEFEFLSDEILEETETAVINAQPMFN

IQYETKSKKGYTYKKALQEALARGIQEIEHNFDFCKDGFYSPKNIAIVALLVNVIDRLHTNEWSSIIRKSFHMCFFNNGK

DKLFHMMSIPKTFIKNPIGEIPNWEKIRTFIVQLETAYPGNNWHQMFEHFKEKCILLIDREIKMEGMSWGEMLDELDDYK

DTEMFHFN

>Melao_virus_(from_unknown)_(APA28993)

MDRAEYQQFLARINAAKDACVAKDIDVDLLMSRHDYFGKELCKSLNIEYRNDVPFIDIILDIRPDVDPLTIDAPHITPDN

YLYINNILYIIDYKVSVSNESSIITNTKYYELTRDISDKLNLYIEIVIIRIDPITRELYISSDRFKDMYPTLVVDINFNQ

YFDLKQMLYEKFGDDDEFLLKVAHGDFTLTAPWCKTGCPEVWQHKIYKEFKMSMPIPERRLFEESMKFNSYESERWNTNL

IKIREYTKKDYQEFVTNSAREVFLATGDFKQPNKKEINEGWDLMVQRVSSQRDVSKSVHDQKPSIHFIWSTHNPNNSNNA

TFKLILLSKSLQSIKGNSTYSEAFKSLGRMMDIGDRMSEYESFCEGLKQQARSTWKQVMNKKLEPKRINESLVLWEQQFM

INTDVISKTDKIKLFRDFCGIGKHKQFKNKMLEDIDISKPKILDFNDETIYLTSLTMMEQTKLILSEKSGLKTNNFILDE

FGSKIKDCNKDTFDIMAAIFETRFWQCISDFSTLMKNILSVSQYNRHNTFRIAMCANNNVFALVYPSADIKTKKATVVYS

IIVLHKDETCIFNPGSLHATFKCSNGFISISRAIRLDKERCQRIVSSPGLFLTTCLLFKHENPTIRLDDIMTFSIYTSLS

ITKSVLSLTEPSRYMIMNSLAISSNVKDYIAEKFSPYTKTLFSVYMTRLIKNACFDAYNQRKKVQLRDIYLSDYDITQKG

IKDNRELTSIWFPGSVTLKEYLTQIYLPFYFNAKGLHEKHHVMVDLAKTILEIEDEQRKNIIDIWSSNCKKQTVNLKILV

HSLCKNLLADTSRHNHLRNRIENRNNFRRSITTISTFTSSKSCIKIGNFQKEKEIQLSRQKKTIEIESRKRRLANPIFVS

DEEISLEIGHCNYNMLREAMPEYVDYMSTKVFDRLFELLDTNQVDDKPTIEMIMDMMVNHKDFYFTFFNKGQKTSKDREI

FVGEYEAKMCMYAVERIAKERCKLNPDEMISEPGDGKLRVLEQKSEQEIRFLVETTRQRNRDIDEEIEQLAADGFEENIV

KIEKLARGKARGLKMEINADMSKWSAQDVFFKYFWLIALDPILYPQEKERILYFLCNYMDKKLILPDELLFNLMDQKIAY

KEDIISTLTNQLRSNTVQIKRNWLQGNFNYTSSYVHSCAMSVYKDILKEAMSFLEGSILVNSLVHSDDNQTSVTIVQDKV

PDEVLIEFAIKEFEKVCLTFGCQANMKKTYVTNCIKEFVSLFNLYGEPFSIYGRFLLTSVGDCAYIGPYEDLASRISSAQ

TAIKHGCPPSLAWVSIAISHWMTALTYNMLPGQSNDPLDYFPAESRKEIPIELNGVLDAPLSMISTVGLEAGNLYFLIKL

LNKYTPIMQKRESVVNQIAEVRNWDVNVLDENELFRLKILRYLVLDAEMDPSDIMGETSDMRGRSILTPRKFTTAGSLRK

LYSFNKYQDRLASPGGMNELFTYLLAKPELLVTKGEDKTDYMESVVFRYNSKRFKESLSIQNPAQLFIEQILFSHKPVID

FSGIRDKYINLHDSRALEKEPDIIGKVTFTDAYRILMRDLNSLPLTNDDIQVIYSYIILNDPLMITIANTNILSIYGSPQ

KRTGMSCSTMPEFRNLKLIHHSPALVLRAYSKNNPDVQGADPTEMARDLVHLKDFVANTNLEEKMKQRIKKNEDEKGARD

IIFELKEMTRFYQVCYEYIKSTEHKIKVFILPAKAYTTTDFCSLMQGNLIKDREWYTVHYLKQILSGGHKAIMQHNATSE

QNIAFECFRLITHFADSFIDASSRGAFLRLILDSFSYKDVKVSKLYEIIRSGHNRTDFIPLLFRTGDLNQHDLDRYDAMK

SQERVTWNDWQTSRHLDMGAINLTISGHNRSITIIGEDNKLTYSELNITKNTPENVTISGRNLLKSRHGLKFENMSRVTT

FPGSYYITYRRKDRHQFVYQIHSHESIIRRNEEHLAIKPRLFNEIVPVCLVNVAEVEGDQRILIRSLDYLNNDVFTLSRI

KVGLEEYAVIKKAHFSKMVSFDGPPIKTGLLNLTELMKSQDLLNLNYDNIRNSNLISFSKIICCEGSDNIDDGLEFLSDD

PMTFTEGEAIHATPIFNIFYSKKGEKHMTYRNAIKLLIERETANFEEAFTFHNDGFLSPENLGCLEGIVSLIRVLKTNEW

STVIDKCIHICLIKNNMDYVYHKFDIPSCFYENPITRNINWMMYRDFILTLPKTYVAPWNIMLEHFRAKCTALILDKMET

QRDFSEFAKLMRRKEGKTNLDFN

>Bruconha_virus_(from_unknown)_(AKB96243)

MAILIEDVIRQYTARIRACNNPEIGRDILAEITMTRHNYFAQKFCEAINIEFRNDVPAADIVLEMVPDLDLTKIKIPNIT

PDNYYRDGTKIYIIDFKVSVSDESAQHTYKKYNTLFGDVFNLLNVEYEVVIIRMNPSDMHLHVSSDNFSALFPNITLNVT

FDWYFRLRDDLFHQFRDNEEFMELIAHGEFTPTIPWINEGTPELFNHAVFQDFIDSMPEDNREDFYYALNHNAFQSDKWN

DLLHVIMRKYGDKYTNFIKSNAREIFITDSNFNKPSRDAILQGWAEMVERTHEQREVIDDLSKQKPSIHFIWGPNSKSES

NENNTKIIRLAQKLQSIREKDTFSIAFKNIGLLMDFSEDIDRYEAFCAKLKAEARSSLKPKSKKIEPLKIGSSTILWEQQ

FKLDTDIMPKEVRLRFLKEFCGIGNHKQFKDRMLDDLDLNKPKILNFENQDIKNQAYAMMQNTSFLMSKESGLTKIGNTI

EEFKDKIVNANDKTWELITEISKTRYWQSINDFSTIIKNILSVSQYNKHNTFRIVCTGNNNFFGIVYPSASIQSKRSTIV

FSTIVLHNDEAEILKCGALYKTYKVKNGYISISKAMRLDKERCQRLVTAPGLFMLTSLLFKGECDVNLPDVMNFAFFTSL

SITKSMLSLTEPSRYMIMNSLALSSHVREYIAEKFSPYTKTLFSVYMTDLIKRGCMAANDQRQQISIRDVFLNEFEITQK

GVTNDRNLQSIWFPGKVNLKEYINQIYMPFYFNAKGLHNKHHVMIDLAKTVLEIELDQRINIPNPWGSDLKKQSVNLDIL

VFSIAKMLNLDTSRHNHLRSRVESRNNFRRSLTSISTFTSSKSCIKVGDFREHKERNALHMKKIQNKDAKKTRIANTEFV

SENDRDLEIVHSTYIDLRKSIPNYIDYMSTKVFDRLYEKFKNNEFEDKPAIELIMDTMKNHKDFKFCFFNKGQKTAKDRE

IFVGELEAKLCLYGVERIAKERCKLNPDEMISEPGDGKLKKLEINGESEIRYLIDATKTVTRERSKIDELLDMPKGIKLE

INADMSKWSAQDVFYKYFWLIALDPILYPYEKQRIIYFFCNYMNKELILPDEMMCSILDQKAERENDLIRQMTNGLTTNT

VNIRRNWLQGNLNYTSSYIHSCSMMVFKDILRETAALLEGKCNVNSMVHSDDNQTSVIMVQDKLNNDIITNFVCNTFERC

CLTFGNQANMKKTYITNHIKEFVSLFNIYGEPYSVYGRFLLPAVGDCAYIGPYEDMASRLSATQTAIKHGCPPSLAWVSI

ALNHWITFNTYNMLPGQINDPTKVFHFDRKELPIELCGLLQADLSTIALVGLESGNISFLTSLLRKMSPPQLVKESVQAQ

CQNIEQWDLEELSESEIMKLKILRYVVLDSEVREDSTMGETSDMRSRSLITPRKFTTPASLERLVSYKDFQDILSDNTKI

EELLDKMIENPELLVTKGENADEFMTTILYRYNSKKFKESLSIQSPTQLFIEQILFANKPVIDYTGIQDRYLSILDVPRV

QENEGIIGRKTIPETFIAIKRDLSLMTLDHKDIKLVYSFCILNDPLNTTACNAILLSQVQSLMDRTSLSAVTMPEFRNMK

LIKYSPALVLRAYIHKNLTVAGANEDAMRRDIFHLEEFIEQTRIRDRLEKRIRDNEEIRGERDRMFEIKEITKFYQACYD

YIKSTEHRIKVFILPMKAYTAFDFCATIHGNLIKDKGWYAVHYLKQIVSGTAKANVSQAPASEMIIIGECFRLIAHFCDT

FIDASSRLQFLYNIIDNFSYKNIPVKDLLNTMLRTNKRQQFLPILHWLDELTQHDVDKYDAYKSNERVVWNDWQVNRDMN

TGNIDLTIKGYQRTIRIIGEDDFLKIAELEVLKGDSTPVETHGRKLLNSRHGLKFEKMKKYPVLEQNTYYICWQYRTKFS

YTYQLLLSNIIEARNSQTVSVTGGKFNELVPVCPVIVCRVDSNEKMSLRQIKYLNMECSLTKLQLNQNEFAVIKRCHFSK

MVFFHGPEMIVGNINITNLIQTPSLLTTNYPALSQMPMMTLTRIFNCTGETQEIDEFEFLSDELLEENETAVINAQPMFN

IQYETKTKRGHTYKKALQEALARGIEEIEYNFDFCREGFYSPKNIAIIALLVNIIDRLHTNEWSSIIKRSFHMCFFNNGK

DKLFHMMNIPKTFIKNPIGEIPNWEKIRTFITQLETTMPGNNWHQMFEHFKEKCIILIDREIKTEGMSWGEMLDELDDYK

DTEMFHFE

>Oropouche_virus_(from_unknown)_(ASZ70170)

MSQLLLNQYRNRILHCREPEIAKDIWRDLLNDRHNYFSREFCRAANLEYRNDVPAEDICAEVLDGYKARKVRFCTPDNYL

LHDGKMYIIDFKVSVDDRSSRITREKYNEIFGEVFNPEGVDFEIVIIRLDPSNMAIHVDSRDFMNTIGPITLNISMQWFF

DMKDFLFGKFRDDDKFHAIISQGEFTMTLPWIEEDTPELLTHPIYNEFMNSMPEAEQALFKEALEFKSFGAEKWNIFLKG

VMSKYGEYYKEFTKGHAHSIFLTTGDYPKPDKNQISAGWREMVNRVSSERDMSNDINQEKPSMHFIWAQNDSSSNNNIQK

LIKLSKSLQAMSGTGSYVNAFKSLGRLMDISSDIKKYESFCGKLKSLARSSIKKLDRKIEPIQIGTATVLWEQQFKLDTD

VIKREDRIHLMKDYFGIGKHKSFSKKLNNDINTDKPKILNFDNDDIVRKCKDKYNQVIHNLSQINELDKIGNYLEHFSTK

ISACSVEMWDFIYNTTKTKYWQCINDYSTLMKNMLAVSQYNRHNTFRIVSCANNNVFGLVMPSSDIKTKKATLVYAIIAL

HNEEAEIAELGSLYSTFKTATGYISISKAFRLDKERCQRIVSSPGLFLMTSCLLFNGNKSLEFDKLLGFSFFTSISITKT

MLSLTEPSRYMIMNSLAVSSHVREYISEKFSPYTKTSFSVVMTDLIKKGCYSAYEQRKKVQIRDIKLTDYDITQKGVDSK

RDLKSIWFPGKVNLKEYLNQIYLPFYFNSKGLHEKHHVLIDLAKTVLEIEKEQRESLPEPWSEIPAKQTVNLNVLIYSIA

RNLNLDTSRHNFVRSRVENANNFNRSITTISTFTSSKSCIKIGDFEEEKKRKTKNDTKKLAKDISKLTIANPAFLDEITN

EHEIRHSTYEDLKQSIPDYTDYMSVKVFDRLYEKITTNEISDKETVKLILETMKKHKIFHFGFFNKGQKTAKDREIFLGE

FEAKMCLYLVERLAKERCKLNPEEMISEPGDSKLRVLEKQSEDEIRYISNTIKTLGNAIESLQSGSLNWADICENKARGL

KIEINADMSKWSAQDVLFKYFWLIVLDPILYPAERKRIIYFLCNYMQKRLIMPDELLTTILDQRVPYSNDIIGLMTNNYR

SNTVEIKRNWLQGNLNYTSSYLHSCSMSVYKDIIREAATLLEGEALVNSMVHSDDNQTSICMVQNKLPDDNIIEFCIKIF

EKICLTFGNQANMKKTYLTNFIKEFVSLFNIHGEPFSIYGRFLLTAVGDCAYLGPYEDLASRLSATQTAIKHGCPPSLAW

VSIALNHWITHTTYNMLPGQNNDPLPFFPTNNRSEIPVEMCGILESDLSTIALTGLEAGNVTFLTNIARKLSXPILQRES

IQDQYNSIEKWDLSKLSQIDILRLKMLRYISLDSSVTSDDGMGETSEMRSRSLLTPRKFTTSGSLNRLKSYKDFQDIIAD

EDKTNELFENFIRHPELLVTKGETFEEFVNTILFRYNSKKFKESLSIQNPAQLFIEQILFSNKPVIDYTSIHDKIFGLQD

MPGIEELDTIIGRKTFVESYVQIVDDLSNLTLDINDVKTIFAFCLMNDPLLITSANNIIMSVKGHSQKRIGQSACKMPEV

RSLKLIHYSPAVVLRAYVRGPTNVPNVDIDELARDLSHLEDFIQSTKLRENMRERIEINEKRHLGRDFKFEIKELTRFYQ

VCYDYIKSTEHKVKVFILPYKVFTSIEFCGALTGNLINDKLWYITHYLKNIVSTTHKAQISSSPELELQIADEALRLVAH

FADTFLASESRIQFLKKIIEEFTYKGIPVKHLYSKIKNSKLRVKFLGILLWLDDLTQNDLDKFDADKSDEKIIWNNWQVS

RDMNTGPIDLMISGYSRQLRITGEDDKLIAAELQVTRLSEDLIYRHGQAMLNKPHGLKLEKMQPVTEMSKRLHYIVFQQR

SRKRYFYSILPTQVIEDHNSRVESSRLSRDSKWVPVCPVAISKLYQQGRPILSKVRNLNMQTHSLSRIQVNVDEYAITRR

AHFQKMPFFEGPSIPSGGMDLSELMKSTSLLSLNYDNIKNASLLDMSRVFKCNGSEDDQMAFEFLSDEILEQDVVEEIEC

NPIFSISYTKRGESNMTYKNAFHKALISECDKFEEAFDFLDMGFCSNENLSILEEIHWIISYLKTNQWSTELDNCIHMCM

YRNGYDAEYHKFDIPSKFLKDPINRTINWTEVIEFILLIEDFQTKIEPWSSMKSHFCSKAHSVALECMKNEKRSLAEFVD

RSKKTGKSKFDF

>Ingwavuma_virus_(from_unknown)_(AHY22331)

MNPEKVDEYRNKIENCRDPEKAQELWRALLVERHNYFSREFCDSAGLEYRNDVLAEDICSEVLPNHIAKKVRHCTPDNFY

IDGNGKIYIIDFKVAIDEKSAREAREKYNNIFGEIFNANGIDYEIVIIKHNPATGMTMIDSDNFRRELGQFNLTVDLSWF

FELKDFLFGKFKDDERFLEIINQGDFTMTLPWLEEDTPELYEHVKFVEFIKSMPDEEQETFFKALDFRTDDAAKWNECLQ

EIMEQYKGRYNSFVKRCAQEVFNTTGDYPKPTKEAIDEGWAEMVDRIKTERNVTTDMAKQKPSFHFIWSPNSDESNANIP

KIIKLAKKLQSITGVGTYVKAFKALGRLMDFSSDVSGYERFCNKLKQEARSTSKRLDKKIEEFTAGTCTALWEQQFKLDT

SVMAKEDRIHLMKDFFGIGNHKSFAKRLNDDLDLEKPTILDFNDPDVIRKCKAQYGNVEQILGKVSNLERIGNYLEHYSP

KIQGASAEMWDTVFKISRTQFWQCINDYSTLMKNMLAVSQYNKHNTFRVVTCANNNVFGIVMPSSDIKTKKATLVYCVVT

IHSSEEDVAHLGSLYGTFRSNNCFVSISKAFRLDKERCQRIVSSPGLFMMTACLFIGDNYTLHLDNLLNFSFHTSVSVTK

AMLSLTEPSRYMMMNSLAMSSHVKEYIAEKFGPYTKTAFSVVMTDLIKKGCYNAFNQREKVQLRDIHMTDYDVTQKGVQK

KRDLRSIWFPGKVDLKEYINQIYMPFYFNSKGLHEKHHVLIDLAKTVLEIEKDQRENLPKPWSERPAKQTVNLDILIYSV

AKNLNLDTSRHNFVRSRVENSNNLKRSITTIATFTSSKSCIKRGDFTEFKTRIQKKVDKNIKKEIKKVTIANPAFIDEVT

NDMEIHHATYVDVKKAVPEYIDFMSTKVFDRLYELLKENKYGTERTIDIIFKAMKDHTEFVFAFFNKGQKTAKDREIFLG

EFEAKLCLYLLERIAKERCKLNPDEMISEPGDSKLRILEKQAEEEIRFLARTIKNVNKELLDKLKIGAWGGEFSLDDLGQ

DKAHGMKIEINADMSKWSAQDVIYKYFWLVAMDPILYPDEKKRILFFLCNYMQKKLLLPDELMQTILDQRVPRYNDIIGQ

MTEGYRRNWVVIERNWLQGNMNYTSSYLHSCSMSVFKDVMRECASLLDGEVLVNSLVHSDDNQTSICMVQNKMSDENIIE

FSIKLFEFICLTFGNQANMKKTYITNFIKEFVSLFNIHGEPFSVYGRFLLTAVGDCAYLGPYEDLASRLSATQTAIKHGC

PASLAWISIALNHWITHTTYNMLPNQMNDPLPFFPATNREEIPIEMCGLLNSELQTIALVGLEAGNLSYLTSLLRRMSPV

TYQREPVQSQCSQIKTWDLSLLTVMDVIRLKSLRYIALDSEVTTDDGMGETSEMRSRSLLTPRKFTTSGSLNRLVSYNDF

QRIISDEQEKELMFEYFVRHPELLVTKGENAREYMNSVVFRYNSKKFKESLSIQNPSQLFIEQILFSNKPIIDYTSIHDK

IFGLQDHPEMEEMDTIIGKKTFVQSYVQILDDLSKFNLTHEDIETVYSYCLLNDPLLVTASNNIVMSVKGAEQERLGQSA

CRMPEMRSLKLIYHSPALVLRAYVSRNPEIPGADPDEMLRDLTHLEEFIDKTKLRVHMRERIELNQVKMMKRDLQFEVKE

LTRFYQVLYDYIKSTEHKIKVFILPYKVYTPIDFCAALTGNLIKDDKWCIVHFLKNIISTTHKAQVALCPDLEVQLAMEC

FRLIAHFGDMFLSENSRVPFLKYVIEHFSYKNVPVTRLYDKLRESKHRTKFIPILFHMDDLTQRDLNKYDADKSDERITW

NNWQVSREMNTGPIDLIITGYDRQIQIRGEDDQLLGAELRLARVSRETISGHAKAMLNRKHGLRFEKMKKVEEMSDRLDY

IVYQQRGRNRFFYNILPKQIILDQNSRVDKTKSIGESKWIPVCPVVVRKLFQQKRPDKDKIMKLNMQVYTLSKLQVNPNE

YATVRKAHFQKMTFFVGPPIKSGGMDIAALMSSPSLLSLNYDTMAQASLIDMCRVFKCEGFREDQMAFEFLSDEVMQVDI

EEQLECNPIFSVVYNTKGEQGMTYKGAFRTALIRECEKFERAFDFLDLGFCSNENLSILEEIHWIVIELKTNQWSTELDQ

CIHMCMYRNNLDPEFHQFDIPAAFLDNPIARTVKWKKVKKFIEVLRDFEVREEPWKAIVQHFCDKASRLVFEKIKETQKS

TGFEAFIDPSKKGGRSKFDFN

>Mahogany_hammock_virus_(from_unknown)_(ALP92135)

MAQPIRDLVRQFAARIRTCTSPELGRDLLSDITVARHNYFAQEFCYSIGIEYRNDVPALDIVMEMVPDFNPRNIRVPNIT

PDNYLRDGNKIYIIDFKVSVSDESSNHTFKKYNTLFGDVFNQLGIEYEVVIIRMDPSSMHLHISSDNFANLYPNVILNLD

FSWYFRLKDELFARFRDNEEFMELVAHGEFTPTIPWVNEETEELFEHPVFIDFMDSIGPEARNDFFQALNYNAFQADKWN

DLLHMFMRKYGLKYRDFLKDMSKKIFLADENFNKPTRFEIQKGWAEMIDRVKDTRNVTTDIHKQKPSIHFIWSPHDSNKP

HENNLKILELSAALQKIKESDPISSAFRYIGKLMDFSSDTTGYELFCTKLKKDARSTPGKKSTPIDPFKTGTCTALWEQQ

YKLDTEIIPKDIRLKFLKEFCGIGNHKQFKNRMLDDLDLEKPTILDFSNLDVQRNANLMFQDAKSMMSKHSGLQKIGNTL

EEFKDKIINANEKTWENIEIIGRTRFWQAINDFSIIIKNILSVSQYNKHNTFRVVTSANNRFFGLVYPSASIQSKKSTIV

FSTITIHKNVKDVLKCGALYKTYKIGDEFISISKAIRLDKERCQRLVTAPGIFMLTTLLLKGETEIDLDEIMAFSFFTSL

SITKSMLSLTEPSRYMIMNSLAVSSHVREYIAEKFSPYTKTLFSVYMTELIHKGCLTANNQRFKISVKDVFLNEYEITQK

GVSDEREIESIWFPGHVNLKEYINQIYLPFYFNAKGLHNKHHVMIDLAKTVLEIELEQREQLPDPWGNDFKKQSVNLDIL

VYAIAKMLNNDTSRHNHLRSRVENRNNFKRSLASISTFTSSKSCIKVGDFKEYKSKMVKRLKKIQEKDAKKTRIANTEFV

AEEDRDLEIAHSTYLDLIKSIPDYTDYISTKVFDRLYEKFKTGEFEDKPAIEIIMDVMKTHKNFKFCFFNKGQKTAKDRE

IFVGELEAKLCLYCVERIAKERCKLNPEEMISEPGDGKLKKLEINSESEIRYLIEMTRKEMSKEEEVLEKLIGQPKGIKI

EINADMSKWSAQDVFFKYFWLIVLDPILYPFEKQRILYFFCNYMDKELILPDEMMCSLLDQKADRENDIIRQMTNNFHSN

TVNIRRNWLQGNLNYTSSYIHSCSMMVFRDIVKDACELLEGNCNVNSMVHSDDNQTSVIIIQDKLNNDCVLHYICDLFEA

CCLTFGNQANMKKTYITNNIKEFVSLFNIYGEPFSVYGRFLLPAVGDCAYIGPYEDMASRLSATQTAIKHGCPPSLAWVS

IALNHWITFNTYNMLPGQINDPTKIFNCQRDELPIELCGTLKADLSTIALIGLEAGNISFLTNLLKKMSPPQLIKESVQS

QCNNIEFWDISKLTKMEIIKLKILRYIVLDTDITGDNTMGETSEMRSRSIITPRKFTTTSSLSRLVSYQDYQEVAQSKEK

FDELLEDMLDHQELLVTKGECPEDFIKTILFRYNSKKFKESLSIQSPTQLFVEQILFSNKPIIDYSGIRDKYVGLLDIPR

VQENDTIIGKKTIPETFETLYQDLMKFSLDVDDIKLVYSFCILNDPLNTTACNAILLSQVQSLMDRTSLSAVTMPEFRNM

KLIRYSPALVLRAYLHGTYTLGGATEEGMKRDLYHLEEFITQTGILEKVQNRIIDHEQRIGERDLLFEIRERTKFLQTCY

DYIKSTEHRVKVFILPCKVYTAFDFCATIHGNLIKDKGWYSVHYLKQIVSGTSKAIVNQNPASEQINMDECFRLISHFSD

TFIDPSSRLTFAKKLIDEYTYKNIPIKKLYEQLIKDPNKRQQFIPLLYHMNELKQSDLDKFDANKSNERVSWNDWQVNRD

MSTGTIDLTIKGYMRSIRIKGEDDKLEIAELQLIVGDNTSIESHGRKLLNSRHNLKLERMQNYKYLEPNMYYICWQKRNK

YAYTYQLLLSEIIESRNKQQISLISGKVNLLNPVCPVIISRTPSIEKIRITSLRYLNQECELSKLRITQNEYATTKRCHF

SKMPFFYGNEFVVGNIDINKLMSTPSLLSVNYASLCQTPLITLAQIFRCDGTGEEIDEFDFLSDETLEETNTEPVNAIPF

FNVSYPTKSKKGFTYKQALQQALRAGLEEMEKEFDFTGQGYFSPKNIGVISFIIGLVNRLNTNEWSTVLVKCIHMSFFNN

KKDRIFHMFKIPKVFIKEPVGEIIDWNKAREFLLGIKPKDETNHWGQMFNHFKHKCLTAIDLEIKMEGTSWGDMLDMIDE

FKDEEMFHFE

>Capim_virus_(from_unknown)_(YP009362054)

MAHLIRELVRQYRARIRACTQPELGRDILTEITVARHNYFAQEFCESTNIPYRNDVPALEIVQAIRPDFDPMTRRIPDLT

PDNYYIDGEKMYIIDFKVSVSDESSIHTYRRYTELLGDIFEFIGVEMEVVIIRMDPSNMQLHISSDNFLQLFPNIILNLD

FNWYFQLKEELFERFRDNEDFMELVAHGEFTPTIPWTNEETPELFEHPVFREFLNSMPERERANFQRALNNNAFQSDKWN

DLLHTMMREYGDNYKTFIKDMAKRVFIADENYNKPSRSEIQKGWNEMISRVRESREMTCDVHKQKPSIHFIWSPHNPEIT

NENTAKILRLSKGLQQIKERDNMSLAFKSLGKLMDFSSDVRGYERFCNKLKQDARSTPKKKTQKIEPLVIDDCTILWEQQ

FKMDTNVIQKDIRLKFLKEFCGIGNHKQFKDRMLDDLDLNKPTILDFSNIEIQRHANLMFQDTKSLLSKQSGLSKVGNIM

DEFEEKITNANKDTWETIESVAKTRFWQAINDFSVIIKNILSVSQYNRHNTFRVVCSANNQFFGIVYPSASIQSRKSTIV

FSTIAFHDEAKDVLKCGSLFRTYKIDKGFVSISKAIRLDKERCQRLVTSPGIFLLTTLLFKGQTDIDLNEIMAFAFFTSL

SITKSMLSLTEPSRYMIMNSLAVSSHVREYIAEKFSPYTKTLFSVYMTELIRKGCMSANNQRTKISVKDVFLNEYEITQK

GVSDARDLESIWFPGHVNLKEYINQIYLPFYFNAKGLHNKHHVIIDLAKTVLEIELEQREQLPNPWGSEFKKQSVNLDIL

IYAISKMLKNDTSKHNHLRSRVENRNNFKRSLASISTFTSSKSCIKIGDFSEHKSDIVKRMKKIQNKEAKRTRIANTEFV

ADDERDLEISHSTYLDLLKSIPNYTDYISTKVFDRLYEMFKIGEYDDRPAVQIIMEVMKNHKDFKFCFFNKGQKTAKDRE

IFVGELEAKLCLYCVERISKERCKLNPEEMISEPGDGKLKKLEINSESELRYLIEMTRRELSKEDKFLGELTSEPKGIKI

EINADMSKWSAQDVFFKYFWLIALDPILYPNEKQRILYFFCNYMNKELILPDELMCSLLDQKAERENDIIRQMTNNFHTN

TVNIRRNWLQGNLNYTSSYIHSCSMMVFRDIVKECSDLLEGNCNVNSMVHSDDNQTSVIMIQDKLHNDCILNYICSLFEA

CCLTFGNQANMKKTYITNNIKEFVSLFNIYGEPFSVYGRFLLPAVGDCAYIGPYEDMASRLSATQTAIKHGCPPSLAWVS

IALNHWITFSTYNMLPGQTNDPTKVFDCARSDLPIELCGLLKADLSTIALVGLESGNISFLTNLLRKMSPPQLIKEGVQS

QCNNIQNWDLTKLTNMEVLKLKILRYIVLDTDITDDNTMGETSEMKSRSIITPRKFTTLSSLTKLVSYNDYQNVVSNANL

LDELLENMLENQELLVTKGESSEDFRNTILFRYNSKKFKESLSIQSPTQLFVEQILFSNKPIIDYSGIREKYVGLVDIPN

NQEDEGIMGKKTIPEALEILRNDLNRMILTLDDIKLVYSFCILNDPLNTTACNAILLSQIQSLMDRTSMSAVTMPEFRNM

RLIKYSPALVLRAYLHGDMSIGGAVEEDMRRDLYHLEEFINQTGILRKVEARIQAHQIENGARDMLFEIRERTKFLQTCY

DYIKSTEHRVKVFILPCKAYTAFDFCATIHGNLIKDKGWYSVHYLKQIISGTAKAIVNQSPASEQINMDECFRLVSHFAD

TFIEPSSRLYFADKIIKNYRYKNIPVIDLYNQLKRDANKRQHFIPLLYYMKELKQSDLDRFDANKTYEKVSWNNWQVNRD

MSTGTIDLTIKGANRSIRILGEDDQLKIAEMQLIEGDNTSVESHARRLLNARHNLQFEKMKEYTYLEPGMYYICWQKKSK

FTFNYQMLLCEIIENRNKQPATLLASRINLLHPVCPAIVSRIPSPEKIRITALKYLNQECELSRLQLLKNEFATTRRCHF

SKMTHFAGVEFVVGNIDVNRLMGTPSLLSVNYPSLSQTPLITISQIFRCNGVETEVDEFEFLSDEILEDTDTAPVNAIPF

FHVSYSTKSKQGYTYKQALQHALRMGLEEMENEFDFTGQNLGFFSPKNIGIISFLVGLVNRLNTNEWSTVMLKCIHMSFF

NNHKDRMFHMFKIPKIFIQDPIGEKINWVKAKEFLNGVMPKDEMNHWGQMFMHFKRKCFEAIDLEMKMEGSSWGEMLDML

DDYKDEGMFSFG

>Madrid_virus_(from_unknown)_(YP009362071)

MAILLEDVIRQYSARIRNCNNPEIGRDILAEITMTRHNYFAQRFCEAIGIEYRNDVPAADIVLEMMPGLDLTRIRIPNIT

PDNYYRDGTKIYIIDFKVSVSDESAQHTYKKYDSLFGDVFNQLNIEYEVVIIRMNPSDMHLHISSDNFSALFPNITLNVT

FDWYFRLRDDLFQQFRDNEEFMELIAHGEFTPTIPWVNETTPELFDHEVFHDFIASMPLENREDFYYALNHNAFQSDKWN

DLLHVIMRKYGDKYRDFIKANARRIFLTDSNYNRPTTEAILQGWSEMIERVHDQREVIDDLSKQKPSIHFIWGPNSRNES

NENNTKIIRLSKKLQQIKEKDSFSLAFKNIGLLMDFSEDIDKYEAFCAKLKSDARSSLKPKSKKIDPIKIGNSTILWEQQ

FKMDTDTIPKEIRLKFLKEFCGIGNHKQFKDRMMEDLDLGKPKILNFENQDIKNQAYAMMQNTAYFMSKDSGLAKVGNIL

EEFKDKIINANEDTWNLISEIAKTRYWQSINDFSILIKNILAVSQYNKHNTFRIVCTANNNFFGIVYPSTSIQSKRSTIV

FSTIVLHESENEILKCGALYKTYRMKNGFLSISKAIRLDKERCQRLVISPGLFMLTALLFKGECEVNLTDVMNFAFFTSL

SITKSMLSLTEPSRYMIMNSLALSSHVREYIAEKFSPYTKTLFSVYMTDLIKRGCMSANDQRQQISIKDVFLNEFEITQK

GVTNDRNLQSIWFPGKVNLKEYINQIYMPFYFNAKGLHNKHHVMIDLAKTVLEIELDQRVNLPTPWGTNLMKQSVNLDVL

VFSIAKMLNLDTSRHNHLRSRVENRNNFKRSLASISTFTSSKSCIKVGDFKEHKERNALHMKKLQGKEAKKTRIANTEFV

SDNDRDLEIVHSTYLDLRKSVPNYTDYMSTKVFDRLYERFKNHDFEDKPAIEVIMDVMRNHKDFKFCFFNKGQKTAKDRE

IFVGELEAKLCLYGVERIAKERCKLNPDEMISEPGDGKLKKLEINGESEIRYLIDATKNLSREQSQIDELLDTPKGIKLE

INADMSKWSAQDVFYKYFWLIALDPILYPFEKQRIIYFFCNYMNKELILPDEMMCSLLDQKAQRDNDLIRQMTNGFTTNT

VNIRRNWLQGNLNYTSSYIHSCSMMVFKDILKEVSALLEGRCNVNSMVHSDDNQTSVIMVQDKLNNDIITKFVCDTFERC

CLSFGNQANMKKTYITNHIKEFVSLFNIYGEPYSVYGRFLLPAVGDCAYIGPYEDMASRLSATQTAIKHGCPPSLAWVSI

ALNHWITFNTYNMLPGQINDPTKIFHFERKELPIELCGLLQADLSTIALVGLESGNISFLTSLLKKMSPPQLVKESVQAQ

CQNIDQWDLENLSESEMMRLKILRYVVLDSEVREDSTMGETSDMRSRSLITPRKFTTPASLERLVSYKDFQEILADPLRT

EDLLDTMINNPELLVTKGENSEEFMTTILYRYNSKKFKESLSIQSPTQLFIEQILFANKPVIDYTGIQDRYLSILDVPKI

QENEGIIGRKTIPETFVAIKRDLSLMTIDNKDIKLVYSFCILNDPLNTTACNAMLLSQVQSLMDRTSLSAVTMPEFRNMK

LIRYSPALVLRSYIHNNLTIGGANENAMKRDLFHLQEFINQTKIRERLERRIADNEEIKGERDRMFEIKEITKFYQACYD

YIKSTEHRVKVFILPMKTYTAFDFCATIHGNLMKDKGWFAVHYLKQIISGTAKASVSQAPASEMIVVGECFRLIAHFCDT

FIDVGSRLQFLYNIIDNFSYKNIPVKALLDTMMRTNKRQHFLPILHWLNELTQHDIDKYDAYKANERVIWNDWQVNRDMN

TGNIDLTIKGYQRTIRIIGEDDFLRIGELEVLKDDTTPIETHGRKLLNARHGLKFEKMQRYKVIEPNTYYICWQNRTRFS

YTYQLLLSNIIEARNAQTVSVTGGKFNELVPVCPVIISKVDSDEKMNLRQIKFLNMDCSLTRLQLNQNEFAIVKRCHFSK

MVFFNGPEMIVGNMNITNLIQTPSLLTTNYISLSQMPMMTLTRIFNCNGEDSEVDEFEFLSDEILEETETATINAQPMFN

IQYETKSKKGFTYKKALQEALSRGIQEIEENFDFCKDGFYSPKNIAIIALLVNVIDRLHTNEWSTIIRKSFHMCFFNNGK

DRLFHMMNIPKTFIKNPIGEIPNWEKIRTFIVQLETAFPGNNWHQMFEHFKEKCILLIDREIKMEGMSWGEMLDELDDYK

DTEMFHFS

>Maguari_virus_(from_unknown)_(ARI46638)

MEDQIYDQYIKRIQSAKTATVAKDISTDILEARHDYFGRELCSAIGIEYKNNVLLDEIILDVVPGVNLMNYNIPNVTPDN

YIWDGDFLIVLDYKVSVGHDSTEITYKKYTSLILPVMEELGIPSEIAIIRANPVTYQISIIGENFKARYPNIPIQLDFSK

FFELRKMLLDKFADDEEFLLMIAHGDFTLTAPWCMEDTPELYEHQIFNEFLGSMPPRFVNLFKESLAFSAYSAERWNSLL

YNVKASTEKDYQDFLSIKSHNIFNMDGNYMRPTQAEIDEGWELMSRRIAKERDIITDINKQKPSIHFIWTKNADRKLSGS

TAKLIFLSNSLQAISEPSTWTESLKAIGKSMDIDGNVGLYENLCLERKLIARSTGKKIDNKRLEAVKIGNALVLWEQQFI

LANDLFQNQERQKFFKKFLGIGGHKVFKDKTATDVDFDKPKILDFNNTIILMAARSMVNKNKAYLSQANTLANPHPIMET

FSRQIAEASEETATILKKINKTCFWQCITDISTIMRNILAVSQYNRHNTFRVAMCANDSMYALVFPSSDIKTKRATVVFS

IVCIHDDRQSIMNAGALFTTLELKTKNFISISKAIRLDKERCQRIVSSPGLFLLSALLLYNNNTEVSLTDVLNFTFYTSL

SITKSMLSLTEPSRYMIMNSLAISSHVKDYIAEKFSPYTKTLFSVYMVNLIKKGCSSANEQSSKIQLRNIYLSDYDITQK

GVNDERNLDSIWFPGKVNLKEYINQIYLPFYFNAKGLHEKHHVMIDLAKTVLEIEMNQRMDNLGIWSSHEKKQHVNLPVL

VHSLAKSLILDTSRHNHLRNRVESRNNFRRSITTISTFTSSKSCIKIGDFKELKSKAHQTSLKNQKKACEKYRLSNPLFI

LEEETSLEVNHCNYEDLTAKIPNYRDYISVKVFDRLYELFKIGVLDDKPFIDQAMTMMKEHKDFSFTFFNKGQKTAKDRE

IFVGEFEAKMCMYVIERISKERCKLNTDEMISEPGDSKLRILEKKAEEEIRYIVEKTKDSIQKGDPTKALKLEINADMSK

WSAQDVFYKYFWLIALDPILYPSEKKRMLYFMCNYMEKVLILPDDLLSNILDQKTPYNNDLILNSTNGLNSNFVKIKRNW

LQGNFNYISSYVHSCAMMVYKDIFKETMKLLEGECLVNSMVHSDDNQTSLAIMQNKLPDNIMIQFAADTFESVCLTFGCQ

ANMKKTYITHTCKEFVSLFNLHGEPLSIYGRFLLPSVGDCAYIGPYEDLASRLSATQQSIKHGCPPSLAWVAISCSHWIT

HLTYNMLNDQINSPINYLPFESRHNIPVELNGYINAPLYLISLVGLEAGNLWFLIEMLKKLVPLDKQRETIQTQCQWLSG

LNNLTESEKFRFKILRYLTLDTEVSADSNMGETSDMRSRSLLTPRKFTTPGSLNKLVSYLDFKESMSSDKYKETLDYMYN

NPELLVTKGEDKEQFMESILYRYNSKRFKESLSIQNPAQLFIEQILFSHKPIIDYSSIFDKLSSLVESDVIAELPEIIGR

VTFPQAYQMIYRDITQLPLDLEDIKIVYKYCVLNDPLMITAANTSLLCVKGTPQDRNGLSANQMPEFRNMKLIHHSPALV

LKAFSKGRTDLPGADPIELEKDLHHLNEFLDNTGIKEKIDQNIENPPKHLTGLEIIMYKIRETTKLYQLCYDYIKSTEHK

VKIFILPMKAYTAIDFCTLVQGNTLSDSKWYTMHYLKQILSGSVKGNIVTTSTTEQIIAGECFRTLVHFADSFVEEASRL

SFITEVIDNFQYKNIPVNSLYNTILGSNLRLDFIPLLFRMQALTQSDLNKFDALKTNERVSWNNWQTNRSLNSGVIDLTI

SGYLRSIRIVGEDKMLKIAELTIPNFYPNTLFHAGNQLLNSRHGLKFEYMSECVLDDKYNYYITYQKKRAHLYTYQVSTV

EHIFRRNQEGLASRGTRYNKMTPVCPVVLSVRDELFRMSLHNVFSLNMTNFSMSRLYVSPDEMATIKKAHMSKMMFFNGP

DIKAGIINLTSLMRTQELLSLNYDNICKSSIIPFCRILSCDGNEDSGELIFLSDEIMDFTISEEIESMPIFTIKYQKKGH

EKMTYKNAISKLVTRGVEEITTIFDFSEDGFYSKKNLGIINTLCSLINLLETNEWSSILLNSFHIAMLMEGMDKEFHMFS

LPSPFFINVAGGIIDWTKLLKFIKSLPKIIHEPWSMMMERFIDKTIYLIERELNKEANFNDFLNELEFQSGKSLFSFF

>Madre_de_Dios_virus_(from_unknown)_(AHY22333)

MSQLLLNQYRNRILHCREPEIAKDIWRDLLNDRHNYFSREFCKAANLEYRNDVPAEDICAEVLDGHKARKVRFCTPDNYL

LHDGKMFIIDFKVSVDDRTSRVTREKYNEIFGEIFNPEGVDFEVVIIRLDPSNMAIHVDSRDFINVIGPITLNISMQWFF

DMKDFLFGKFRDDDKFHAIISQGEFTMTLPWIEEDTPEMLTHPIYNEFINSMPEAEQLLFKEALEFKSFGAEKWNIFLKK

VMSQYSDYYKEFTKGHARSIFLTTGDYPKPDKDQISEGWKEMVNRVSSERDMSNDINQEKPSIHFIWARNDATSNNNIQK

LIKLSKSLQSMSGTGSYINAFKSLGRLMDISADVKKYESFCGKLKSLARSSVKKLDRKIEPIQIGTATVLWEQQFKLDTD

IIKREDRIHLMKDYFGIGKHKSFSKKLNNDINIEKPKILNFENDDIVRRCKDKYSQVVSNLSQVNELDKMGNYLEHFSAK

INSCSVDMWDFIYNITKTKYWQCINDYSTLMKNMLAVSQYNRHNTFRIVSCANNNVFGLVMPSSDIKTKKATLVYAIIAL

HNEETDIAELGSLYSTFKTTTGFISISKAFRLDKERCQRIVSSPGLFLMTSCLLFNGNKSLELDKLLGFSFFTSVSITKA

MLSLTEPSRYMIMNSLAVSSHVKEYISEKFSPYTKTSFSVVMTDLIKKGCYSAYEQRKKVQIRDIKLTDYDITQKGVDSK

RDLKSIWFPGKVNLKEYLNQIYLPFYFNSKGLHEKHHVLIDLAKTVLEIEKEQRESLPEPWSEIPAKQTVNLKVLIYSIA

RNLNLDTSRHNFVRSRVENANNFNRSITTISTFTSSKSCIKIGDFEDEKRKKTKNDAKKLAKDISKLTVANPAFLDEITN

EYEIKHSTYEDLKQCIPNYTDYMSVKVFDRLYEKITTNEISDRETVKLILDTMKKHKVFHFGFFNKGQKTAKDREIFLGE

FEAKMCLYLVERIAKERCKLNPEEMISEPGDSKLRVLEKQSEDEIRYISNTIKTLGNAIESLQSGSLNWADICDNKARGL

KIEINADMSKWSAQDVLFKYFWLIALDPILYPAERKRIIYFFCNYMQKRLIMPDELLTTILDQRVPYSNDIIGLMTNNYR

SNTVEIKRNWLQGNLNYTSSYLHSCSMSVYKDIIREAATLLEGEALINSMVHSDDNQTSICMVQNKLPDDNIIEFCIKIF

EKICLTFGNQANMKKTYLTNFIKEFVSLFNIHGEPFSIYGRFLLTAVGDCAYLGPYEDLASRLSATQTAIKHGCPPSLAW

VSIALNHWITHTTYNMLPGQNNDPLPYFPTSNRNEIPVEMCGMLESDLSTIALTGLEAGNVTFLTNLARKLSPPILQRES

IQDQYNSMEKWDLTKLSQIDILRLKMLRYISLDSSVTSDDGMGETSEMRSRSLLTPRKFTTSGSLNRLKSYKDFQNIISD

ENKTNELFENFIRHPELLVTKGETFEEFVNTILFRYNSKKFKESLSIQNPSQLFIEQILFSNKPVIDYTSIHDKIFGLQD

MPGIEELDTIIGRKTFVESYVQIVDDLSNLPLDIDDVKTVFAFCLMNDPLLITSANNIIMSVKGHSQERIGQSACKMPEV

RSLKLIHYSPAVVLRAYVRGPGNVSNVDIDEIARDLAHLEDFIQSTKLRENMRERIEINEKRHLGRDFKFEIKELTRFYQ

VCYDYIKSTEHKIKVFILPYKVFTSIEFCGALTGNLINDKVWYITHYLKNIVSTTHKAQISSSPELELQIADEALRLIAH

FADTFLASESRVQFLQTIIEEFTYKGIPVKHLYSKIKNSKLRVKFLGILLWLGDLTQNDLDKFDADKSDEKIIWNNWQVS

RDMNTGPIDLMISGYSRQLRITGEDDKLIAAELQVTRLSEDLIYRHGQAMLNKPHGLKLEKMQPVAEMSRQLHYIVFQQR

SRKRYFYSILPTQVIEDHNSRVESSRLNRDSKWVPVCPVAISKLYQQGRPILSKVKNLNMQNYSLSRIQVNVDEYAITRR

AHFQKMPFFEGPPIPSGGMDLSELMKSTSLLSLNYDNIKNASLLDMSRVFKCSGSENDQMAFEFLSDEVLEQDVIEEVEC

NPIFSISYTKRGDSNMTYKNAFHKALISECDKFEEAFDFLDMGFCSNENLSILEEIHWIISYLKTNQWSTELDNCIHMCM

YRNGYDTEYHKFDIPSKFLKDPINRTINWTEVIEFILLIEDFQTNIEPWSSMKSHFCSKAHSVALECMKSEKRSLTEFVD

KSKKTGKSKFDF

>Mapputta_virus_(from_unknown)_(AKO90170)

MDPDIKKQLELKIMTCRDPAIAREIHTEILEMRHDYFGKEICKSLNLEYRNDVPLETILQEVWDDYDLEHVPFITPDNFL

FDNNCLYIIDYKVSVSMESSLATIEKYNEKMEQIGHLLPFRYEIVIIKIDPYSKILYINSDNFQKLFNNILIDVDFTDFI

DMRAMLLEKFQDDEDFVAMIAHGDFTMTSSWTDESCEEYKDHPVFKEFKYSMPIPYRRLFEEAMTFNAYKAERWNTQLIK

MRDYTRTSYNDFITEQANKIKTLDGTYPKPDKNEIMAGWRLMRRRIHQMRELTTDPSKQKPSGHFIWSPPSGGNNNNIAK

ILLLSRSLQSIKDNSPNAKMFNAIGKMMDFSADVDQYIKHIEGLKNESRRLNRKITSQKLEPKIIGSAKVLWEQQFVLNN

DYCEQNGRQTFYKKFLGIGGHKKFQDKTTTDLDMSKPEILDFDDKTIILAANAMIKETDQLLYQESIVKKHETIFDSYML

KIKNASEEMHSTINAIMKTNYWNCLNDISILIKNLLSVAQYNKYNTFRVVLCANNNMFGLLFPSGDIKTRQSTLLYCIVA

LHKDQNAILDPGALYQTFKIKDGYLSVSRAMRLDKARCQRLVASPGLFLLTSALFKGENSTLSIREILTFAFYTSVSITK

AMLSLTEPARYMMMNSLAISSHVKDYIAEKFSPYTKTLFSAYVCNLIKKGCIIANNQKDKIEIRDIYMTDFEMIQKGVKD

VRELQSIWFNGKVNLKEFINEIYLPFYFNAKGLHEKHHVLVDLAKTIVEIELEQRQSIKDIWSSIPKKQTVNLAIFTHAL

SKSLILDTARHNHLRSRVENRNNLRRSITTISTFTSSKSCIEIGSFKDLKSKQYSIQTAANKKQLEKMRVANPLLIEENE

EMLQTQHSNYNMLVEAMPNYQDFISIKVFDALYKRLKHNVLTDGPCVKEILKIMKTKVDFFFTFFNKEQKTYVDREIFVG

EFEAKMCMYLVERIAKERCKLNPEEMISEPGDGKLKVLESRAETEARYLIRETAEHNKRILDNTSDTLEKMSKMKRGFRL

EINADMSKWSAQDVMFKFFWLTALDPILYPEEKEHIIYFYCNYLNKKIILPDNLMCNLLDQKILHDNDILAYATNGFTTN

AINIKRNWLQGNFNYTSSYLHSVSMQTFKDIIKATLPEQQVVINSLVHSDDNQTSLVLIQDLYPEEIMIEHILNEFEIVC

LTFGCQANMKKTYATNLIKEFVSLFNICGEPFSVYGRFLLTSASDCAFIGPYEDFASRISAAQAGIKHGCPPSLAWLSIA

IAHWMTATTYNMLPGQANDPSKNMPIDFNETPIELGGPLQAPLYLTALIGLEANKIYDMLNILKKLVNPLMIREEVAMQV

SNIHEPSLHKLTEAERFKLKLIRYLVLKSDIQIDDKLGETCDMRSRSILTPRKFTTVGILKKLESFNDHANIMSTTAEAE

KVLDFMIENPELLVTKGETKEQYLNSILYRYNSRRFKESLSIQNPVQLFIEQILFSLKPTIDYNSILGENMIISDTNIDE

KHNIIGNVTIAKAMSNLYQDINEMDLTLDDIKICFMFTILNDPLIITAANSYLLSTQSNQSPRLGLSCSTMPEFRNMKLI

HHSPALVLRAYCKKTVNLPGIDPVEMQRDLNHLEHFIAQSKLEIAMNDVLQRHKDDGTYTKQLELRELTKFYQTAYDYIK

STEHKIKVYILPHRAYTTIDFCALLQGNLIKDSCYINIHYLKQITSSTHKGEVKTISNSEIMIAKECFRALAFFLDTFIS

LNDRVRVLETVIDTFTFKSCLVKDLYNLIKTSQYRYEFLPLLYRMNDLTQADIDRYEAMKSDDNVVWNQWQVNRMFDKGD

IDLIITGKDKFIRIIGSDDNLRLAELHIPTNKEDMIKSAGRRLLNQRHGLRFERMKEIIMDEKNLYIAYQKRQKNQYYYM

IQTTDYINNRNSEIKQKSTRDQNLIVPVCLVYVAVDVSRRRVYIETLEHQNHDNYDIARLRLTLDEYATIKRAQISKVLL

FDGPEITSNVFSITKLMKTHELLEPNFDRLLRKNIIPLAKIFKCSGHSIDDFDFSEQPMEADDIAEVDCKPIFSVIYPKK

GNKKMTYKNAILSIIDRELTIAELELTLIDESFFAGENRGLIEAIVALIDLLESNEWSTILKNIIHMLYLKHDMDAEFHL

IECPEIFKQGDDLNVTMIEEFIHSLPKITNATWSIIFENFKDKIILLINDYKEKKQPKNIVKFSKLLKKADKSTKGLFSF

F

>Catu_virus_(from_unknown)_(AKO90158)

MAHQLGEIVRQFAARVRTCNNPELGRDLLSDITVARHNYFAQEFCYSIGIEYRNDVPALDIVMEMVPDFNPTQIRVPNIT

PDNYYRDGNKIYIIDFKVSVSDESGNHTFKKYNTLFGDIFDQLGVEYEIVIIRMDPSSMHLHISSDNFANIFPNVILNLD

FSWYFRLKDELYERFRDNEEFMELVAHGEFTPTIPWVNEETAELFDHPVFIDFMNSLGPEARNDFFQALNYNAFQADKWN

DLLHMFIRKYSMKYKDFLKDMSKRVFMADEHFNKPSRLEIQKGWAEMIDRVRETRDMVTDIHKQKPSIHFIWSPHDGTKP

QENNAKILELSVSLQRIKETDHISTAFKYIGRMMDFSSDINAYEQFCSRLKRDARSTSRQKSTPIEPIKIGPCTALWEQQ

FKMDTDVIPKEIRLRFLKEFCGIGNHKQFKNRMLDDLDLEKPQILDFSDINIQRNANLMFQDTKSMMSKKSGLQKVGNVL

EEFKDKIEGANEKTWEHIEHIAHSRFWQAINDFSVIIKNILSVSQYNKHNTFRVVASANNRFFGLVYPSASIQSKKSTVV

FSTVCLHKDQKDVLKCGALYKTYKIGDQYISISKAIRLDKERCQRLVTAPGIFMLTTLLLKGDTEIDLNEIMAFSFFTSL

SITKSMLSLTEPSRYMIMNSLAVSSHVREYIAEKFSPYTKTLFSVYMTELIHRGCMSANNQRTKISVKDVFLNEYEITQK

GVSEDRDLESIWFPGHVNLKEYINQVYLPFYFNAKGLHNKHHVMIDLAKTVLEIELEQREQLPNPWGKDFQKQSVNLEIL

IYSIAKMLNNDTSKHNHLRSRIENRNNFKRSLASISTFTSSKSCIKVGDFSEHKTKTVKRLKKVQEKDAKKTRIANTEFV

AEEDRDLEIAHSTYLDLIKSVPEYTDYISTKVFDRLYEKFKTGEFEDKPAIEIIMDVMKEHKDFKFCFFNKGQKTAKDRE

IFVGELEAKLCLYCVERIAKERCKLNPEEMISEPGDGKLKKLEINSESELRYLIEMTRREMSKEEEFIETLTKEPKGIKV

EINADMSKWSAQDVFFKYFWLIVLDPILYPYEKQRILYFFCNYMDKELILPDEMMCSLLDQKAERENDIIRQMTNNFHTN

TVNIRRNWLQGNLNYTSSYIHSCSMMVFRDIVKDATELLEGNCNVNSMVHSDDNQTSVIIIQDKISNDCILHYICDLFEA

CCLTFGNQANMKKTYITNNIKEFVSLFNIYGEPFSVYGRFLLPAVGDCAYIGPYEDMASRLSATQTAIKHGCPPSLAWVS

IALNHWITFNTYNMLPGQVNDPTRIFYCERDELPIELCGILKADLSTIALIGLEAGNISFLTNLLRKMSPPQLIKESVQS

QCNNIEFWDLTKLTKMEIIKLKILRYIVLDTDITGDNTMGETSDMRSRSIITPRKFTTLSSLSRLVSYNDYQAITQSKEE

FDNLLEDMLEHQELLVTKGECPGDFIKTILFRYNSKKFKESLSIQSPTQLFVEQILFSNKPIIDYSGIRDKYVGLLDMPQ

VQESETIIGKKTIPEAFETLNKDLEKFELDVDDIKLVYSFCILNDPLNTTACNAILLSQVQSLMDRTSMSAVTMPEFRNM

KLIRYSPALVLRAYLHGTYTLGGATEEGMKRDLYHLEEFINQTGILQKVEERIRDHEARIAERDLLFEIRERTKFLQTCY

DYIKSTEHRVKVFILPSKAYTAFDFCATIHGNLIKDKGWYSVHYLKQIVSGTAKAIINQSPASEQINMDECFKLISHFSD

TFIEPSSRLSFAKRIINEYTYKNIPVKKLYEMLIKDLNKRQHFMPLLYHMNELKQSDLDKFDANKTNERVSWNDWQVNRD

MSTGTIDLTIKGYMRSIRIKGEDDRLEIAELQLVIGDNTSVESHGRKLLNTRHNLKLERMQTHKFLEQGMYYICWQRRNK

YTYTYQILLAEIIENRNRQPVSLISGKANLLNPVCPVIVSRAPSMEKIRLTALRYMNPEVELSKLKITQNEFATTRRCHF

SKMPFFYGSEFTVGNININKLMSTPSLLNVNYASLCQTPLITLAQIFQCDGQTEEVDEFEFLSDEALEEIDTAPVNAIPF

FNVSFPTRSKKGYTYKQALQQALSTGLDEMEREFDFTGQGYFSPKNIGVISFMIGLVNRLNTNEWSTVLVKCIHMSFFNN

KKDRVFHLFKIPKVFIKDPIGEIVDWNKAREFLIGIKPKDETNHWGQMFNHFKQKCLNAIDLEIKMEGTSWGDMLDMIDE

FKDEEMFHFE

>Wolkberg_virus_(from_unknown)_(AQM74166)

MDDQLYNQFLRRIRVASDPAVAKDIDTDILMSRHDYFGREICRALEIEYRNDVPIIDIFLEVQPDFDPMRYTIPNITPDN

YLYRNGTLYIIDYKVSVSTESSIQTNKRYRDILDSICPIIGLNYEVVIIRANPMNNEIYFSSDSFRNLFGPLVINLDFSR

FLDIKKLLFEKFGDNEEFLLKVSHGDFTITAPWCKERTPELYDNNIFIEFMNSMSEEYQDMFLDSIMFNPHEADKWNVHL

INLKEKTNREYEKYITDIAVKLFESTGNYKKPTRTEIEKGWEEMSNRVQKEREISKDVMDQKPSGHFIWSPPDDAYPKDN

IGKILFISKLLQDMSGSSEFLDAFRMIGKAMDFSANVPLYNRVTLTRKEEARSQKGKVMNKKLEPVQIGEALVFWEQQFS

LVNDKFDKVVRSNLLKNFFGIGSHKQFKSKMIDDIEVEKPKILDFNSDDIYLESIKMLQSTKNILGQNNNLDENKDFIYS

NFSKKIKTCSERMEACLQKIIKCNFWNCLNDISILMKNMLSLSQYNRHNSFRIATCANNSIYGLVLPSSDIKTKRATLVF

CILAVHKEKESLLTPGALHATFKSGQYYVSISRAIRLDKERCQRLVTSPGLFLTTSMLFYYYSPNSELLDIMTFAFYSSI

SITKSLLSLTEPSRYMIMNSLALSSHVREYIAEKFSPYTKTLFSVYVTRLIKIACFNANSQRERISLRSVALSEYDITQK

GVEDNEDFDSIWFPGKVTLKAYINQIYLPFYFNPKGLHEKHHVLIDLAKTVLEIEKDQRLNCPEPWSDEALKQTVNLPVL

IHSLAKNLLVDTSRHNHLRNKIENRNNFNRSITTISTFTSSKSCIKLGNYSELKEKQAKDREKGTLSVLKRMRIANPLFV

NEEDLKNEVAHSNYNNLRQSIPLYKDFISTKVFDRLYELLKTENLSEDGATIQNIMKMMKDHTDFYFTFFNKGQKTAKDR

EIFVGEFEAKMCMYAVERLAKERCKLNPDEMISEPGDGKLKVLEMKSEQEIRFIIETIKQRNQNLMDDILLGNQRQKFKA

TKIEINADMSKWSAQDVFFKYFWLIAMDPILYPNEKERMLYFMCNYMNKKLILPDELLCNILDQKMIYRDDIIMEMTDNL

NKNYVEIKRNWLQGNFNYTSSYVHSCAMSVYKDILRLVSIKTGSEVLVNSLVHSDDNHTSIVYIHDKLHQDLFIEHSIMS

FEKVCSAFGCQANMKKTYLNNTIKEFVSLFCISGEPFSIFGRFLLTSVGDCAYIGPYEDMASRLTSTQTAIKHGCPPSLA

WLSIAINHWITFTTYNMLPGQVNDPTARLPLKNRSELPVELFGILNADLSTIALTGLESGNLTFLVELLVKNSPIEMRKE

TIIDQSLLIKTWDINKLSDAELFKMKILRYLVLDAEIETDSVMGETSEMRGRSIITPRKFTTIGTLKKLISYTDYQKMLS

DEQKINEVFEYMATKPELLVTKGETKEDYMHTILFRYNSKKFKESLSIQNPAQLFIEQILFSHKPVIDYSGIRDKFSMIG

DNLELEEQPDIIGRMTFNQVYENLSKDMAALPLTLEDVHVVYDYMILNDPLLTTVANALILKLESLPQSRTGVTACNMPE

MRNLKLITHSPALVLRAYSQKNPDIPGADIEEMRRDLLHLEEFVEKTKLKEKMEDRIAKNQMANRNERDIKYELKELTKF

YQICYEYVKSTDHKIKVFILPSKAYTSIDFCALVQGNLIKDEKWIMIHYLKSINTIGYKGIIQRTSSGEMNLAMEAIRLI

AYFGDTFINPYSRKRFLKEIIDNFTYKFVPVEYLYRLILNSHIRHEFLPVLYWTGELTQHDLDKYDAMKANEHVSWNDWQ

VNRNLGTGPINLKITAYNKSLYILGEDDILKVSELQLTKISNDYIHIAGKRLLSSKHNLRFELFKRYNITSDFNYYITYQ

RKAKNIFVYQILNTQTIERRNAEHAAVKTRVYNEIVPVCPVIIAEMPRSQGIELKTIRNMNYDNNSLTRLKISDREYSIM

KRAHLYKMQNFDGPKIKTGILNVNKLMKTPELLNTNYSKVSRSSIVSLSKILECDGTTDDDVLEFLCDDPMDDEEVETIE

SEPIFTVTYLKKGKKVNSYKSAIKTLIFNETETFESIFDFSKLGFLSGKNLGILEVIVSLVKILRTNEWSTIMLNCIHIC

LIRHGYDRQYHLFELPRMFIIDPITHKLNWGELKRFVINLPEINVSPWDIIFTNFKAKCSELIDRELGKENRFGYYLDSL

EKDFGKSMFDFE

>Playas_virus_(from_unknown)_(ARI46654)

MEDQIYDQYIKRIQSAKTATVAKDISTDILEARHDYFGRELCSAIGIEYKNNVLLDEIILDVVPGVNLMNYNIPNVTPDN

YIWDGDSLIVLDYKVSVGHDSTEITYKKYTSLILPVMEELGIPSEIAIIRANPVTYQISIIGENFKARYPNIPIQLDFSK

FFELRKMLLDKFADDEEFLLMIAHGDFTLTAPWCMEDTPELYEHQIFNEFLGSMPPRFVSLFKESLAFSAYSAERWNSLL

YNVKANTEKDYQDFLSIKSHNIFNMDGNYMRPTQAEIDEGWELMSRRIAKERDIITDINKQKPSIHFIWTKNADRKLSGS

TAKLIFLSNSLQAISEPSTWTESLKAIGKSMDIDGNVGLYENLCLERKLIARSTGKKIDNKRLEAVKIGNALVLWEQQFI

LANDLFQNQERQKFFKKFLGIGGHKVFKEKTVTDVDFDKPKILDFNNTIILMAARSMVNKNKAYLSQANTLANPHPIMET

FSRQITEASEETATILRKINKTCFWQCITDISTIMRNILAVSQYNRHNTFRVAMCANDSMYALVFPSSDIKTKRATVVFS

IVCIHDDRQSIMNAGALFTTLELKTKNFISISKAIRLDKERCQRIVSSPGLFLLSALLLYNNNTEVSLTDVLNFTFYTSL

SITKSMLSLTEPSRYMIMNSLAISSHVKDYIAEKFSPYTKTLFSVYMVNLIKKGCSSANEQSSKIQLRNIYLSDYDITQK

GVNDERNLDSIWFPGKVNLKEYINQIYLPFYFNAKGLHEKHHVMIDLAKTVLEIEMNQRMDNLGIWSGHEKKQHVNLPVL

VHSLAKSLILDTSRHNHLRNRVESRNNFRRSITTISTFTSSKSCIKIGDFKDLKSKAHQVSLKNQKKACEKYRLSNPLFI

LEEETSLEVNHCNYEDLIAKIPNYRDYISVKVFDRLYELFKLGVLDDKPFIDQAMTMMKEHKDFSFTFFNKGQKTAKDRE

IFVGEFEAKMCMYVIERISKERCKLNTDEMISEPGDSKLRILEKKAEEEIRYIVEKTKDSIQKGDPTKALKLEINADMSK

WSAQDVFFKYFWLIALDPILYPSEKKRMLYFMCNYMEKVLILPDDLLSNILDQKTPYNNDLILNSTNGLNSNYVKIKRNW

LQGNFNYISSYVHSCAMMVYKDIFKETMKLLEGECLVNSMVHSDDNQTSLAIMQNKLPDNVMIQFAADTFESVCLTFGCQ

ANMKKTYITHTCKEFVSLFNLHGEPLSIYGRFLLPSVGDCAYIGPYEDLASRLSATQQSIKHGCPPSLAWVAISCSHWIT

HLTYNMLNDQINSPVNYLPFESRHNIPVELNGYINAPLYLISLVGLEAGNLWFLIEMLKKLVPLDKQRETIQTQCQWLSG

LNNLTESEKFKFKILRYLTLDTEVSADSNMGETSDMRSRSLLTPRKFTTPGSLNKLVSYLDFKESMSSDKYKETLDYMYN

NPELLVTKGEDKEQFMESILYRYNSKRFKESLSIQNPAQLFIEQILFSHKPIIDYSSIFDKLSSLVESDIIAELPEIIGR

VTFPQAYQMIYRDITQLPLDLEDIQIVYKYCVLNDPLMITAANTSLLCVKGTPQDRNGLSANQMPEFRNMKLIHHSPALV

LKAFSKGRTDLPGADPIELEKDLHHLNEFLDNTGIKEKIDQNIENPPKHLTGLEVIMYKIRETTKLYQLCYDYIKSTEHK

VKIFILPMKAYTAIDFCTLVQGNTLSDNKWYTMHYLKQILSGSVKGNIVTTSTTEQIIAGECFRTLVHFADSFVEEASRL

SFITEVIDNFQYKNIPVNSLYNTILGSNLRLDFIPLLFRMQALTQSDLNKFDALKTNERVSWNNWQTNRSLNSGVIDLTI

SGYLRSIRIVGEDKMLKIAELTIPNFYPNTVFHAGNKLLNSRHGLKFEYMSECVLDDKYNYYITYQKKRAHLYTYQVSTV

EHIFRRNQEGLASRGARYNKMTPVCPVVLSVRDELFRMNLHNVFSLNMTNFSMSRLYVSPDEMATIKKAHMSKMMFFNGP

DIKAGVINLTSLMRTQELLSLNYDNICKSSIIPFCRILSCDGNEDSGELIFLSDEIMDFTISEEIESMPIFTIKYQKKGH

EKMTYKNAISKLVTRGVEEITAIFDFSEEGFYSKKNLGIINTLCSLINLLETNEWSSILLNSFHIAMLMEGMDKEFHMFS

LPSPFFVNVAGGIIDWTKLLKFIKSLPKIIHEPWSMMMERFIDKTIYLIEKELNKEANFSDFLDELEFQSGKSLFSFF

>Cache_Valley_Virus_(from_unknown)_(ARI46682)

MEDQMYDQFLKRIQSAKTATVAKDISTDILEARHDYFGRELCASIGIEFRNNVLLDEIILDVAPGINLMNFNIPNVTPDN

YIWDDDFLIIMDYKVSVGNDSTEITYKKYTTLILPVMEQIGIPTEIAIIRANPVTYQISIIGENFKAKYPNIPIQLDFSK

FFELRKLLLDKFEDDEEFLLMIAHGDFTLTAPWCMDDTPELYDHPIYKEFIGSMPPRFVNLFDEAISFSAYSAERWNSLL

YTAKAKTEQDYQEFLSEKSHAIFNMNGEYMKPTQSEIDKGWELMSRRISEERDIITDINKQKPSIHFIWAKNANRKLSSA

TAKLIFLSNSLQSIIEPSTWTDSLKAIGKSMDIDGNVGAYENLCMERKLIARSTGKKIDNKRLEAIKIGNALVLWEQQFI

LANDLFKGQERQKFLKNFLGIGKHKNFKDKTTTDLELDKPKILDFNNTIVLMAARSMINKNKTFLAQTNTLENLHPIMGV

FSDQIKEASEDTFNTLKKINKTCFWQCITDISMLMRNILSVSQYNRHNTFRVAMCANDSMYALVFPSSDIKTKRATVVFS

VVCIHQEKQSIMDAGALFTTLELKTKNFISISKAIRLDKERCQRIVSSPGLFLLSALLLYNNNPNISLVDVLNFAFYTSL

SITKSMLSLTEPSRYMIMNSLAISSHVKDYISEKFSPYTKTLFSVYMVNLIKRGCSSANEQSSKIQLRNIYLSDYEITQK

GVNDERNLESIWFPGRVNLKEYINQIYLPFYFNAKGLHEKHHVMIDLAKTVLEIEMTQRMDDLGIWSKHGKKQHVNLPIL

IHSLAKSLILDTSRHNHLRNRVESRNNFKRSITTISTFTSSKSCIKIGDFREIKAKNLATSVKSQQKACEKYRLSNPLFL

VDEESTLETRHCSYEDLVAKIPNYRDYISVKVFDRLYELFKLKELDDRPFIDQAMEMMQKHKDFSFTFFNKGQKTAKDRE

IFVGEFEAKMCMYVIERISKERCKLNTDEMISEPGDSKLRILEKKAEEEIRYIVEKTKDSIQKGDPTKALKLEINADMSK

WSAQDVFYKYFWLIVLDPILYPNEKKRMLFFMCNYMQKVLILPDDLLSNILDQKTPYNNDLILNSTNGLNANFVNIKRNW

LQGNFNYISSYVHSCAMMVYKDIFKETMKLLDGDCLVNSMVHSDDNQTSLAIMQNKVKDDMVIQFAAHTFESVCLTFGCQ

ANMKKTYITHTCKEFVSLFNLHGEPLSIYGRFLLPSVGDCAYIGPYEDLASRLSATQQSIKHGCPPSLAWLAISCSHWIT

HLTYNMLDEQVNCPTNHLPFTNRKDIPVELNGYINAPLYLISLVGLEAGNLWFLIEMLKKLVPIDKQRETIQTQCQWLAQ

LGNLTESEKFRFKILRYLTLDTEVSTDSNMGETSDMRSRSLLTPRKFTTPGSLNKLVSYLDFKNSMSNDDYKETLSYMLQ

NPELLVTKGENKEQFMTSVLYRYNSKRFKESLSIQNPAQLFIEQVLFSHKPIIDYSSIFDKLSSLVESDIIADLPEIIGR

VTFPQAYQMIVRDINHLPLDLEDISIVYKYCILNDPLMITAANTSLLCVKGTPQDRNGLSANQMPEFRNMKLIHHSPALV

LKAFSKGTTDLPGAEPTELEKDLHHLNEFLESTGIKEKIGQNMDNPPKHLTGMEITMYKIRELTKLYQLCYDYIKSTEHK

VKVFILPMKAYTSVDFCTLIQGNTISDDKWFTMHYLKQILSGSAKGNIVTTSTTEQIIANECFRVLTHFADSFVEEASRL

SFITEVVDNFSYKNIPVNSLYNTILGSNLRLDFIPLLFRMQALTQADLNKFDALKTNERVSWNNWQTNRSLNSGVIDLTI

SGYLRSIRIVGEDKILKIAELTIPNFYPNTVFHAGNKLLNSRHGLKFEFMSEQVLDDKFSYYITYQKKRAHIYTYQVSTI

EHIYRRNQEGLAARGNRYNRMVPVCPVVLSVRDELFRMSMNNVFSLNMTNFSMSRLYISPDEMATIKKAHMSKMMFFNGP

DIKAGIINLTALMRTQELLSLNYDNICKSSIVPFCRILSCEGSEDNGELIFLSDEVMDFTISEEIESMPIFTIKYQKRGN

EKMTYKNAISKLVSHGVEEITTVFDFSKDGFYSKKNLGIINTLCSIINLLETNEWSSILLNSFHIAMLLEGMDREFHLFS

LPSAFFNNVASGNINWTKLLKFIKSLPKIHNEPWSMMMERFIEKTIYLIEKELNKEANFSDFLDELEFQTGKSMFSFF

>Ferak_Virus_(from_Culex_decens)_(Feraviridae)_(AKN56888)

MSHIEELLVEQEDLDDPHKAVEKSQWFLDERSKAAEGYIRNYLI

ILDPEGSFHDSEVNQFLIHRELNPIPTTNQQSRKTPDLMCLYKNILIVGDVAVSRSPE

ETSVRKEIKYRMLIDAIKLANPQLFIRFEAFVFKDDGSNVETVQDRLRNLITEYTPTM

GWKFGSLEIKLRQDIITWNNRLNFIARGVKDKITYIHLYQQLMMEEDDEVLLGSMTQQ

EQYIPKVTERDIAKSIFDKMRSSGPSMKTTKDIDVMKLMDDIEKKRDEDYEEAEIKHC

IPYLFNSGDDSELMDLELLDDYRYDLAIHGKNLSTLFPHGDQLSKMKQIKDEFNEQRI

NKENKDRRTFMKTLKEKYGQATPYYCPKSGLTDRASEEDNIKEWYFSKVNQINDAKKP

GSIKSSEYNTCDKQISSLINVMNMKGKSRSTNIEFDMPDRGDKIRRESQLHEVVKPRF

DDINKKVGTCLLSCVRKFVNDVAHMPLNLKNGIYYSIPIQKNMILLVFTQTNLTKRNP

SVYFMTITRFDKSCSNDLMLFGAQAKLSKYCEETTKYHYIASKLHKYELEKLSKLCNI

DAEFRVHYMSMMSMSDLDEEIEKRYKSSYVGLTCLCLLDLHQKPSEMLDLMKYLVAMP

MSSHSSLDLLLSDKLSLMMKTNLDVYLHRNIFRFLEKNIKLNKTRSLNPLRIQKNGYV

LPESFNITGQFAMFCDTEVITENLLFYISESQLIFHARPKKLYNSQFIDKCAYKVADY

NDKMKKDEEANPGWTTKGFNEMDNGLFNYPFESDFCYSRDAMYYAQLDEDSELARFIP

SMLRTNNKKVADLFPNNLSMRGACIIPGSTLDNTREIKKKIKNHDEVIKMLESVNKTK

REKPLEELFKVRRSTTAMLSGLVMMERYINEDDTEKCRVGQIMSDHKDDEMYFNMSEK

EQRGGGRPIGSADFFTKQRLYCIEMIYQRIGQAQGENLMAKKVNRSAKLSDVSRNRIN

QAIRNDKKVLSYIVMDQSQFSESDNMNKFTANIVDNRCIPENLKGDMIDSLNKMKTRT

QFFPKIPDQVIKEYPEYLTKDGGIRGVAGWVQGMLNISSTHIHIIAVKWLTKIFNKYY

KEFSNKEYDQVEVDHLVNSDDSFAVICSKYSHMVTDFYDFFLLGKRMFCLKQNKKKSY

MSSLIGEVIQKYVANGSTINIWAKDAVSIFNSMRGLDMYKDISTAIGSLQTLSRNGAP

EDACCYVRAECKTKILNMFNVNKGKINDISSLGIDIGKLPIELMGWPKYITTFELCVS

GSFAQANYCVGNFKRSANEKGDWTTKEAMVTIAAVMVNMSNKINDNSDIEIYDKEVDQ

DSNEMVVRNIRRIRDLLEAEDYTDDLNLTVEEIQADEKHREKNLIAYKSLGMSIIGGN

FTKSLVNPFCFCHPFSSKVTETVKFLRNISGEGSGLAGLLKVRTSIRQSVVDIKENVS

TLLMQLSESGFNNDVRMIASASSIVASSRSVTISGSNKRHTIRQCYKILLDISYKMSG

REFSRKSASQIPSILRDPTFRADMADEILPNMQDSGTEAKKPTVVTLMPEMESSLGIA

NPLQLVLAEIVKPGIIVKEGYILNYPDQLSLDINIVKSKFGDWLSVTSNKLQVACGIY

YHYLNARRGRYVVGPPLDKSDMMSFLISWYKEKFSQDFSIVSQFVGDTVRRSEHFSDY

APHALMSAVEMYMKLCVISKTIEKELFINNLFLNLRGAKYSLKQLAHSELVDILQDEV

DVSSKQLLGAMLLDITGDDKWIRRFVRAETTRADWVKEQDKRYNPISKRVTWAGEFIV

DIKKGDDVVRIEGEPGNMKIIKSTTANTRKISDMMLFLRKNNRFDNYEIKKDIKTLYN

DKFYSTMNEVNILTVDEVLDPSSKYLTVRSNPIKEGKDISYFTEKGLIPFKLVQPSEL

TSAKSDMEEYIRFELDVGKYNFLVGVNTSSVLDRDKASGVERYVSRETKTRICKLDQH

YNKFTVTGMESKILMLAGISMQIFIEDNELKNIIRNQVNMISSTRLEKYVKWLVPKSE

IMDSVFRILCSHHCGFAKHENELYSDLKMIDVDITATELNYEQDIDKLTEEQEELGML

EASYVTESEGNMVSKMINAYSSRNIEPQKIVELCELLASTQMSNILQTLRDKVGQGEG

SDWADEVDREQKERELLTNTAAIFQMLFITEMEEMEAFIEEEEEEADPLNIDFKEKTL

STFDDEMFSKGKTKEQCKSILANVNEALECMLLNRLFQYDTFLPLLKKTGSTDFNMKN

KNVLLGRLIASCSRRAISQMQSI

>European_mountain_ash_ringspot-associated_emaravirus_(from_Sorbus_aucuparia)_(Fimoviridae)_(YP003104764)

MENIRKLEAQRRKEYLRAETEIRNNNVFEKDILQKFLHIVGKKQ

RHYTISSKKKEVQDIFNKCSTNPGFTKDVIDLAERILYAPPNLNQIDFAMTIVSLIEM

QRHDDLIHKLNELLVRSGMKVISFEFKLCDHFPFTNSILTPDILFEDPYGSRYILEVK

VRNKHTDLEHYYMRYKKVVGVHAKVGVFNLSQSGYMQHGDYKLSEKINLESDDFDDIL

LCVELAGKIREKYIQYPQYFLYTLQSEVTNPDSFLDGFKTRLSDLSMFEEIKSGFGDY

WNDITYHMDNYSLIDNHDEVTEDLLNSTDDLTQYCNDLYDEFLDHSQSYSRQGKYGKT

ILRNSGLDNIIDKKNRSKYTITSKLKPSVYIPITKTIKLDTYGGSRLKFYKDAFINIK

CTGDSYSRSAYNLVDNVFNTSSIDLLMTKDDKIDPSLYYEVLDPGFIAHLHDDQVKYK

KIAKVTNITSDMTILANNSFSIHCHTDQRLKDNICGYDKKHYDSTQKAKECLDFSNSS

LLLPSLGVHLSAIFKSEHHAGVYWNDLVTLGTDNLNHMTSDIPEEAQTKFLEHLYNSH

IIFKAIISLNTINSHKFRLLQSPDPGTIIILLPNSDGLKGAPLRYFVVSILQKQDNDS

IEANKLLGIYHSHTESKKYKIMLSKVISLDITRLKLLSNSFVKYSLLISYYSQFKKSL

KFDTHMLSWMLSQVTTIASLSITDVYKNFIMAIYSDYSNIDDLINDKLECRPRTLGHV

FVMKHLFQGITSAVEQLGKINKNKLIADVNDEGELISTGFDPNLRLKLPISQLSTNNP

KEIIHESFILFYLGNKGLHGSPQELLNLYYTPMQFESEYTKMMTDCGLYCQELGNNGN

LSFSFQAMFLTSKVAYAKLLNNTDEIRRSLVKEMKMDEPIMSIKQFSSTKSMVSNSVP

DMSIKDCNLNKNIDVIQLERYIDTSLISDPMKYITLMNNSIESINQERLLIYKDKLNK

GIVLLPKLILTTFKGKSFIGLENHYYTKLVSGDYIKQTNTKVFDEFYRLTDEIQEEKL

RGFYKGYITEGDLLVRIFNKDQRTTDDREIYTGNAQVRLCLYPLEMTFKSICKKIPEE

AITISGDQKQRKLLEQRLALIKTKRQFNKSGYKTEIYSVSSDASKWSARDLLPKFIIS

IATNPYLTSDEKYFLVYLLVRYYDKKIVLTDSAFSNALRFSREDINGKYEEMTNNFTQ

NWFNVRSNWLQGNLNMTSSFVHHCSTIMTDTLLSISAKHNGFEAVMTSMVHSDDSTYD

FLIAKNSKTSSYINNEANMGRFIISLITYSNKKHCITLNEKKTYISTFYKEFLSTTIV

SNELFFFYMADLMPISSDTSYKSPLEDLASYTGYINNSFSHACPIQILKCAITLLNHL

TLSTYNMQYTSEKNPRCNIPNSTDLPIQIYPRYKLPLSLAGCIPYYSSDAYNILDDII

KTLEKNKVIKNSLLEDVIDDETLDEYITLVNKQKPEYAKYIQACLLTMDYTQYERDDE

DPYNIVDYDLSQKSIINVASINKGSRIKKTYTYKKYLENETDIRLTSCVNPMWCISKP

KDEVLIKNPILANYMNPNFKDSLIFSKSALDYGRRIIGSNKSMDTLSSHAFEKEKKQG

IKTIYKKLDDKISTVEISKQSLQRFLECIYSVIKKSLVALQVYYSKVQVLVKTRPEFT

KVIMPRSVYAEEYGKNSNTSMVENLLVEQYCEIEQVDSKVEKFISFCKHVLQRCGDIK

IYRDPEDIDDDFRKYIEFKYTLKDATMGLIQPHQHLAEYAFDVYNNKLIFQGLMVRYY

IDICETISNPSYNIPSYTSPNSIIMTLDSLMKRDEISSKIYISHIRTNRFDEYWLSRF

GMYVYENYFVKYKLGYRIKIAANEKLMPTMKKVRNLREPFKFICSLVANDPSLFIQMT

ESPDFQISGWKYSDIIAEMKSTTDFSYNLFLYMMNEINFQTLMRVMNLNRRVWNHWLM

KTDSEPSDPNASIALYMYQSTVVKVQTKTIGGGVTFSMLLLRHGMQHRQAFDEISKKI

ASDYAPQLRIANITPQTSFGRLQFCVNEYGRTVKPGSYRSSCICNVNIAALTDLKPDI

AYKENTINQIVTIISPTFEGEFVFKLNTYCDSEYYTCVMLENLDLNRVMILDHLCRGK

YLIENPEYFTEISDQITPGACLALFSNNVNNKLWSNTIDTSKFAKLVHIGNYLKTEHE

ASIVTKLCDSLVAICALNGIDHTLSLKPDNFIKSLRQYKLSYGFHEEFYNNYKKNERE

PYTELIMAIASTAGDPFQKVILAIITIFKAYTDLFISYKTDEVEF

>Hantaan_Virus_(from_Rattus_sp.)_(Hantaviridae)_(NP941982)

MDKYREIHNKLKEFSPGTLTAVECIDYLDRLYAVRHDIVDQMIKHDWSDNKDSEEAIGKVLLFAGVPSNI

ITALEKKIIPNHPTGKSLKAFFKMTPDNYKISGTTIEFVEVTVTADVDKGIREKKLKYEAGLTYIEQELH

KFFLKGEIPQPYKITFNVVAVRTDGSNITTQWPSRRNDGVVQYMRLVQAEISYVREHLIKTEERAALEAM

FNLKFNISTHKSQPYYIPDYKGMEPIGANIEDLVDYSKDWLSRARNFSFFEVKGTAVFECFNSNEANHCQ

RYPMSRKPRNFLLIQCSLITSYKPATTLSDQIDSRRACSYILNLIPDTPASYLIHDMAYRYINLTREDMI

NYYAPRIQFKQTQNVREPGTFKLTSSMLRAESKAMLDLLNNHKSGEKHGAQIESLNIASHIVQSESVSLI

TKILSDLELNITEPSTQEYSTTKHTYVDTVLDKFFQNETQKYLIDVLKKTTAWHIGHLIRDITESLIAHS

GLKRSKYWSLHSYNNGNVILFILPSKSLEVAGSFIRFITVFRIGPGLVDKDNLDTILIDGDSQWGVSKVM

SIDLNRLLALNIAFEKALIATATWFQYYTEDQGQFPLQYAIRSVFANHFLLAICQKMKLCAIFDNLRYLI

PAVTSLYSGFPSLIEKLFERPFKSSLEVYIYYNIKSLLVALAQNNKARFYSKVKLLGLTVDQSTVGASGV

YPSFMSRIVYKHYRSLISEVTTCFFLFEKGLHGNMNEEAKIHLETVEWALKFREKEEKYGESLVENGYMM

WELRANAELAEQQLYCQDAIELAAIELNKVLATKSSVVANSILSKNWEEPYFSQTRNISLKGMSGQVQED

GHLSSSVTIIEAIRYLSNSRHNPSLLKLYEETREQKAMARIVRKYQRTEADRGFFITTLPTRCRLEIIED

YYDAIAKNISEEYISYGGEKKILAIQGALEKALRWASGESFIELSNHKFIRMKRKLMYVSADATKWSPGD

NSAKFRRFTSMLHNGLPNNKLKNCVIDALKQVYKTDFFMSRKLRNYIDSMESLDPHIKQFLDFFPDGHHG

EVKGNWLQGNLNKCSSLFGVAMSLLFKQVWTNLFPELDCFFEFAHHSDDALFIYGYLEPVDDGTDWFLFV

SQQIQAGHLHWFSVNTEMWKSMFNLHEHILLLGSIKISPKKTTVSPTNAEFLSTFFEGCAVSIPFVKILL

GSLSDLPGLGYFDDLAAAQSRCVKALDLGASPQVAQLAVALCTSKVERLYGTAPGMVNHPAAYLQVKHTD

TPIPLGGNGAMSIMELATAGIGMSDKNLLKRALLGYSHKRQKSMLYILGLFKFLMKLSDETFQHERLGQF

SFIGKVQWKIFTPKSEFEFADMYTSKFLELWSSQHVTYDYIIPKGRDNLLIYLVRKLNDPSIVTAMTMQS

PLQLRFRMQAKQHMKVCRLDGEWVTFREVLAAANSFAENYSATSQDMDLFQTLTSCTFSKEYAWKDFLNG

IHCDVIPTKQVQRAKVARTFTVREKDQIIQNSIPAVIGYKFAVTVEEMSDVLDTAKFPDSLSVDLKTMKD

GVYRELGLDISLPDVMKRIAPMLYKSSKSRVVIVQGNVEGTAEAICRYWLKSMSLVKTIRVKPHKEVLQA

VSIFNRKEDIGQQKDLAALKLCIEVWRWCKANSAPYRDWFQALWFEDKTFSEWLDRFCRVGVPPIDPEIQ

CAALMIADIKGDYSVLQLQANRRAYSGKQYDAYCVQTYNEVTKLYEGDLRVTFNFGLDCARLEIFWDKKA

YILETSITQKHVLKIMMDEVSKELIKCGMRFNTEQVQGVRHMVLFKTESGFEWGKPNIPCIVYKNCVLRT

SLRTTQAINHKFMITIKDDGLRAIAQHDEDSPRFLLAHAFHTIRDIRYQAVDAVSNVWFIHKGVKLYLNP

IISSGLLENFMKNLPAAIPPAAYSLIMNRAKISVDLFMFNDLLKLINPRNTLDLSGLETTGDEFSTVSSM

SSRLWSEEMSLVDDDEELDDEFTIDLQDVDFENIDIEADIEHFLQDESSYTGDLLISTEETESKKMRGIV

KILEPVRLIKSWVSRGLSIEKVYSPVNIILMSRYISKTFNLSTKQVSLLDPYDLTELESIVRGWGECVID

QFESLDREAQNMVVNKGICPEDVIPDSLFSFRHTMVLLRRLFPQDSISSFY

>Joncet_Virus_(from_unknown)_(Jonviridae)_(AKN56871)

MKSYKISLAEINELIVVEDVHDVFQAYDRWAQTIKLRADASEKL

VENVIRSSPTVPTHSIGDAPVNDFLSMINANQITEHKRKSPDLLIFIQGSNTLYLGDV

AVTRSARRVRSIKSEKYEPLRQEISNKNSWLRVEELNVIFDEANTNVEAEIRQVSDLV

GTPWDQVNIRDYTDCVDKSKVAVDRLFSTVSNVSELQELIAIGGQDQEHVDFFAGTEQ

MQIPKLSTLRHDEEYFANMIFRRMAQDANPDGSDQTQTISKKDTLKAISDAADANYDP

CMEKVNHNLQFFVNCDEIKSDQTTIDILASMCIDLDVAQNSKIREMLPSSQELIHMKS

MENEKKTLKETRYLNTDELASARDRITLKYGKSTPYYCAYKEMAKKGPRPYAEVYLNA

IKEKAQDKKADKLNAIMTETPFIMPCIPAGKWEEAEKLMRNELSYYAGDADEINAASL

KACKIVTGDHVNQRDKRTDSIISEIEDVIYKTRAYWLLKAVTKFVVDVNRMDPRLSNG

IYYSNSLNSDTIILLHCNNSGFKTNQYFDFQCLTRHRKGTKGNEHALKTHSGQGNAQV

LHVESTAKYTYILTKMVKYDIDTLDQIKDNDKTIPMLVINLTLEYFKDTYNDSSQFKM

NSLVRSFITENMAFYTLLSVDLHQKPSVIMDLMKYVVSMPFSERCNFKMLLKDLLPFK

TKFDVFLFKRMVSYFDWNRINAKTFRSKTFMLVNGQLSSAPGLSNKFTTFVNGTTHNN

SYYHFMEGQMLNQIRPKKLYNEQFLDQACTDIIERNIEFEEEKLKYGSWTQGHSDGKD

YPFEAKYCFSRDVVVQASDKLNRERAGHAMGMKLSIAGKLQELAINHLSMRKGCISRD

EIVERVTKRQKIQEMLDQELSKGMFKSKHRVMLLRKELSKAKGGKSKSALLAGLEALE

PYIKANDTTSCRSINVMKANHDKISQYNTSSKQQRGKGRQIASADFYTKNGLHCIEEA

YKSQSAKDETNLLRSGVNRARAVSQTCEKAINEGIVHGYEHFYYLVEDQTKWSESDNV

GKMKVMIECDIGIPAGLKLPLIRELSKMERRMVFSNKPLGQQPIRKMKQEYAPYITEN

GAILGRIGWMQGMLNFTSTDCAKRCTLWAVQTFNKYYKGYRNNDEGDIIVKSSLNSDD

SFHAVAGPSKRKILDFRNYLYFAKKLFCMKLNIKKSYISSNFGEMIQLYVDGNSVLNI

WMKQMYSGFGSLMGVSYEKDMTHAVSHCSTLIKQGADEMNVRVWWLTAKRAVLKSYNM

NKGGINDMTRIDVDPSMLPISLFGYPTKLSQYSLNMYGVRAHNDYVNTALSTSSVSNE

KLVIMMSLLAKVEKNNTGYLARNNFTINDELVKSSQLAIQCTIEPGDDANPTKHEDDL

LLLELDDDNADTDFGAKQLSIVSTRLPIKHKQAEVLKKLETLTAGVDPSGVQGLIRNN

NNIRTCLRDRLETSQKEMIELSESNFTTNPKLKALCNSYLASRKCFFIKGFPKTSFTA

LGLINHLLVMGRSAKDTMDPSIISGAYNSVSEILKDSTLLVNMIKEIERIESNNTIVM

SAVKRSKVVNSYPTEIGDIDFENDISHILCEIVKPGALSQMDITMKSPHKMEAEKLIV

ENLYKSWFAIMKSSDGSLLKAALDIYHNELSKRISRTIISSHLDSRTKGKFFRDVYLN

CYYPGYKTVSEAVLPPGVYRNPNDKVESDVNTLLALFEIYAAMSGTGDIANQFPKICY

YSELTGSRKLATSENLAADITVLLSQSTKMESRRKLAVFYERLGYGTGEIAKTIRAMS

YSMTWHKEQRSRVVAGRKEWYGDFRVTITKGDLAVTIEGEPGNISKIITNDDDVVTVI

KAMQYLTSYASFPGFNKTTMHKYEDWGRNPFWSSYDTAGAPNGLFYQPQGKTAILPIA

PLDSLMGRIPGRPGLIREGQKLQYIPLFTTTRSVTIDEARIDARYRSPNNRLIKVDME

AGKIYEDPGQLDGKMKVVGTFRVKSWFADYSKLSVNPGDVVTLDGLDFAMAISRGWAE

DIIKGTTKKLEKSSVKMLMRTVLGDSQVVANTMINLFGKWCDANGHAVSVESRMELKG

IPEVVININEEEDDEDAPNIETLSFTMGNTGDVIKDLLVRLVTNSTNYQRGVESASQH

VFKTAEFTSYMQSLLERCEGWDGTNAIDKIIVDSTKKSRLSKTMLDALLEFGFFTRQD

AFLMDALSSVPAATDIDYEMAASTYGVRYSDNKAVSFEDTEPTKEADALTDIIEYMSQ

GDDEEGWY

>Jonchet_virus_(from_Culex_quinquefasciatus)_(AKN56880)

MKSYKISLAEINELIVVEDVHDVFQAYDRWAQTIKLRADASEKLVENVIRSSPIVPTHSIGDAPVNDFLS

MINANQITEHKRKSPDLLIFIQGSNTLYLGDVAVTRSARRVRSIKSEKYEPLRQEISNKNSWLRVEELNV

IFDEANTNVEAEIRQVSDLVGTPWDQVNIRDYTDCVDKSKVAVDRLFSTVSNVSELQELIAIGGQDQEHV

DFFAGTEQMQIPKLSTLRHDEEYFANMIFRRMAQDANPDGSDQTQTISKKDTLKAISDAADANYDPCMEK

VNHNLQFFVNCDEIKSDQTTIDILASMCIDLDVAQNSKIREMLPSSQELIHMKSMENEKKTLKETRYLNT

DELASARDRITLKYGKSTPYYCAYKEMAKKGPRPYAEVYLNAIKEKAQDKKADKLNAIMTETPFIMPCIP

AGKWEEAEKLMRNELSYYAGDADEIDAASLKACKIVTDDHVNQRDKRTDSIISEIEDVIYKTRAYWLLKA

VTKFVVDVNRMDPRLSNGIYYSNSLNSDTIILLHCNNSGFKTNQYFDFQCLTRHRKGTKGNEHALKTHSG

QGNAQVLHVESTAKYTYILTKMVKYDIDTLDQIKDNDKTIPMLVINLTLEYFKDTYNDSSQFKMNSLVRS

FITENMAFYTLLSVDLHQKPSVIMDLMKYVVSMPFSERCNFKMLLKDLLPFKTKFDVFLFKRMVSYFDWN

RINAKTFRSKTFMLVNGQLSSAPGLSNKFTTFVNGTTHNNSYYHFMEGQMLNQIRPKKLYNEQFLDQACT

DIIERNIEFEEEKLKYGSWTQGHSDGKDYPFEAKYCFSRDVVVQASDKLNRERAGHAMGMKLSIAGKLQE

LAINHLSMRKGCISRDEIVERVTKRQKIQEMLDQELSKGMFKSKHRVMLLRKELSKAKGGKSKSALLAGL

EALEPYIKANDTTSCRSINVMKANHDKISQYNTSSKQQRGKGRQIASADFYTKNGLHCIEEAYKSQSAKD

ETNLLRSGVNRARAVSQTCEKAINEGIVHGYEHFYYLVEDQTKWSESDNIGKMKVMIECDIGIPAGLKLP

LIRELSKMERRMVFSNKPLGQQPIRKMKQEYAPYITENGAILGRIGWMQGMLNFTSTDCAKRCTLWAVQT

FNKYYKGYRNNDEGDIIVKSSLNSDDSFHAVAGPSKRKILDFRNYLYFAKKLFCMKLNIKKSYISSNFGE

MIQLYVDGNSVLNIWMKQMYSGFGSLMGVSYEKDMTHAVSHCSTLIKQGADEMNVRVWWLTAKRAVLKSY

NMNKGGINDMTRIDVDPSMLPISLFGYPTKLSQYSLNMYGVRAHNDYVNTALSTNSVSNEKLVIMMSLLA

KVEKNNTGYLARNNFIINDELVKSSQLAIQCTIEPGDDANPTKHEDDLLLLELDDDNADTDFGAKQLSIV

STRLPIKHKQAEVLKKLEALTAGVDPSGVQGLIRNNNNIRTCLRDRLETSQKEMIELSESNFTTNPKLKA

LCNSYLASRKCFFIKGFPKTSFTALGLINHLLVMGRSAKDTMDPSIISGAYNSVSEILKDSTLLVNMIKE

IERIESNNTIVMSAVKRSKVVNSYPAEIGDIDFENDISHILCEIVKPGALSQMDITMKSPHKMEAEKLIV

ENLYKSWFAIMKSSDGSLLKAALDIYHNELSKRISRTIISSHLDSRTKGKFFRDVYLNCYYPGYKTVSEA

VLPPGVYRNPNDKVESDVNTLLALFEIYAAMSGTGDIANQFPKICYYSELTGSRKLATSENLAADITVLL

SQSTKMESRRKLAVFYERLGYGTGEIAKTIRAMSYSMTWHKEQRSRVVAGRKEWYGDFRVTITKGDLAVT

IEGEPGNISKIITNDDDVVTVIKAMQYLTSYASFPGFNKTTMHKYEDWGRNPFWSSYDTAGAPNGLFYQP

QGKTAILPIAPLDSLMGRIPGRPGLIREGQKLQYIPLFTTTRSVTIDEARMDARYRSPNNRLIKVDMEAG

KIYEDPGQPDGKMKVVGTFRVKSWFADYSKLSVNPGDVVTLDGLDFAMAISRGWAEDIIKGTTKKLEKSS

VKMLMRTVLGDSQVVANTMINLFGKWCDANGHAVSVESRMELKGIPEVVININEEEDDEDAPNIETLSFT

MGNTGDVIKDLLVRLVTNSTNYQRGVESASQHVFKTAEFTSYMQSLLERCEGWDGTNAIDKIIVDSTKKS

RLSKTMLDALLEFGFFTRQDAFLMDALSSVPAATDIDYEMAASTYGVRYSDNKAVSFEDTEPTKEADALT

DIIEYMSQGDDEEGWY

>Dugbe_Virus_(from_unknown)_(Nairoviridae)_(NP690576)

MDFLDSLIWERVVDEQYITNPTFCVSDYFEVIRQPGDGNCFYHS

IAELFFDVKTPSSFRKVKEHLQLAAEVYYDTEPEAVGTGISKDEYIKVAMKDNEWGGS

LEASMLSKHLQTTIILWVVNSTEQVTAAIKFGPGRVSTALNLMHVGRTHFDALRIIEQ

LENNQPQDRNRLDIADRIAAAEVYVRQSIEDNLQEDEFFDYAREDEISEDVSAPGGSR

EATELKKKAILLNKTVKRGENIPIRVGRVLDCLFSCKIAVSLDEGLLYLRPETRESEA

TSISLRQLGHKLLTRDRHIKMEYARSKLYVTRDLIDHLDIGGLLRSSFPGLGLERYIQ

LLHSELVLDLVTVVLAVLLSTFLYGSNNKNKKQFITNCLLNTKLSGKRVFKALSKLTG

QMLYRTPKRAVSIVSQELYGKLMLKVKNNLEGMGPISMLALRNLNFDNMQLQDYLEML

SEMSKIDNSDVEYTHREISDLHTLVERLSKLQKSQDVNELKLWFKEEVLTKRSQRSVG

NAFEFLINDYFKKKDIMKFVSTSGKASSTGNIGNVLSYAHNLYLSKESLRMTSEDVTQ

LLIEIRKLHKLQGDLSIEPVAIICDKLEDQFRKLFRELPEECSSECQTLFNDIRNSPS

HSVAWKHALRLKGTAYEGMFAKQYGWSYISEDIKPSLTMIVQTLFPESFEAFLDRTQL

HPEFRDLTPDYALTQKIFFPRNTIPRTENRQLAIDVSLEGSVEAVPIVEKRMFPLPEV

PIGEANSISRVMNIFKEKREESMQKKLEHDRQAEANRLKSAGLSASKAEQEVCNSAQD

RKEEKERTTEPAGKQQRTEDLVVIEGNQDEGDSDPQKKVDEKTVPGESKQHSKSSGSS

STNQMSQKVVDVPSVEDSSDQAPGDFPDYGYYFKRIVMDESGTVLTEEAQLEKRQLLF

IEVGYQTDVDGKITTDYKKWKDILRLLELLNIKCSFIACADCSSTPSNNWWISEDKVR

LLKNSISHLFSKLTQNSPADVTDIVVGSISTQKVRSYLKSGTATKTPISLKDVQETWS

KMKDYIVNRPTGISLNKELVGALYQGLVEGAIISKEGTVNLIQMLKDKQERITDEFER

TKLKHEVNEDVKTSEKLLLGWLMEDLKGCRCMGCLTKIKELSESMSVNQDRLEYLSTN

CQTKSHCTECHPRSLEYRNISNVDNRVPSMQRVSHSRNEGFEDTNETLTELDRLVRLT

LPGKTEKERRVKRNVEGLIRFMMQQSSLDCIKLPSGQIIAHRCSKKFKNSSEAEEKCN

ERFERLMKELSEQKLKPYSDHVRKTITSSLKKTDKQAGSKCAVPRLWLETLIRDLRVP

TKDEEILLNIRTSMQSKTNFIRNNDKLIIRSNKEIADYLETKRKNLLSEKASDKIFSS

DCILFKEVIAEALRRYYSTPYEGVPETIVKLINFLCTFGWFQEVVLYSKICETFLRCC

TEFSRSGIKLVKVRHCDTNLSIKLPSNKKENMLCCLYDKNMSLLKGPFFLNRRQAILG

SAYPYILITLYIQVLQQHRCLEVLNSVNDRVVGNINTCTSNLLNTVKAELTLVNSGLF

EKAYECRTEQCRLGGNFLNRSSRDHFISTVSGLNVVYGALIKDNLLANSQPQNKQLQM

LRFGMLCGLSRLSSALELGKKFSTSCRRIEDNIMRLYLQSTIYSANRDVSQNVQNWKM

KDLCPDITIPCFSVYGLFVNSDRQRIFDIYNVHIYNKEMDNFDEGCITVLEETAERHM

LWELDLLRSLEGDTRDVRAARLLLGCPNIRKATDKDGNRLMKKGITDDWREEAGSDSS

SISGRRSYASSGTRVKSMFGKYNSSQKPFELKPGLEVVNDPLHDYKQAVQDSFCYSEY

TPNTESVLKDCIHIIRTNPSHTMGSYELIQAVTENARRKYPPENIERARKDPKNWVSI

SEVTETTSIVSQPRTHFMLKDCYKVLLGTENKKIVKMLRGKLKKLGAMRTDIEIGKKD

CLDLLTTVDGLSEEQCKNIVNGIFEPSKLSFYHWKDLLKKELSEVLLTDDGNYIYCWL

KTLSSMVKHSLKKDLRFMTGKNSLDIKPEMFTDEEYSALNIMKLELLGEHTDGIQGKT

DFLLSSWKKCALKPKEGQSILNVGLNSLAALHDELYDIRLQHLELTRIKKENPTVSFT

KEEILVKRLEKGFLNKYKKEVMEAVNLIFYCCLTAPWCLHYKSLEAYLVRHPEILETE

CIKENDIPLLDLTVTSLIRSLIDDIEGESSFNDSSDIKVRFAVKYLITLFTANGEPFS

LSLNDGGLNDDLQLTTDEKLLYQTKKVFAKLGLSGNNYDFIWTLQMIANSNFNVCKRL

TGRTTGERLPRSVRSKVIYEMVKLVGETGMAILQQLAFSQALNYDHRFYAVLAPKAQL

GGSRDLLVQETGTKVIHATTEMFSRNLLKTTSDDGLTNPHLKETRLNIGLDMLSTARA

LDGKQVSDDSNLLNFFKTVCISGDNTKWGPIHCCSFFSGMMQQLLKDVQDWSSFYKLT

FIKNLCRQIEIPAPSIRKILNVLRFKLSDKGGVEKLSEEAIRSELINNLAEWEGNDTV

KFLITTYISKGIMAMNSYNHMGQGIHHATSSLLTSMMAETFEELAVDYMKKHFPGLTV

NVDHAGSSDDYAKCIIVSGLVSKDMYKRYDGVFWRHMCRLKNFLAAVRRCCQMKDSAK

TLVGDCFLEFYSEFMMGNRVTPAVIKFIFTGLINSSVTSPQSLVQACHVSSQQGMYNS

VPLVTNAAFTILRQQIFYNHVEDFIRRYGLITLGAVSPFGRLFLPRFSGLVSSSVALE

DSETISKAAAEINSNDIFFNTSSLSNLDKLEQSPDSSGLDDDSVVSTTTVESSDSKGS

SSSFTFDLNRPLSETEVKFLKLLRELTSTTACEMLQEKINTLYNDSREGPLDRHNILQ

NCRLSESCDWLLDGKKRGLLELSRRMSCLLNVLIAGYYRSFGSEGTEKQVKASWSRDD

NRVIEDPMIQLIPEKLRRELERLGLSRMEVDELMPAVGPDESLSQLVAKKLISLNVST

EEYSAEVSRLKQTLTARNVLHGLAGGIKELSLPIYTIFMKPYFFKDNVFLDLEDRWSS

RHSTNYRDSTGKMLTGKVVTKFTHWLDTFLSCVVSANRSQEIKECSLFNPNLRCVNIM

VKGNGIKELSYIRSHLSVLSVEFENLNLQFSDVNRQKLKIVESRPPECELEANKAVII

KSKLFSAVEHVRLSNNPAVVMGYLLEESSISEVKPTKVDFSNLLKHRFKLMQFFPSVF

TLLRSLQSESKELEKLGEPVDMHQVSKYANHLTLLCRMIQQSKPSLTVFYMLKGSQMN

TEPTVSELVSYGIKEGRFLKLPEIGLDASTYSVRYWKILHCISAIGELPLSSEDKTSL

LISFLNWKVTSDCVDDCCPLEKYDKAIVSEFSGQVLINTLASELSSVRKDQEREGLTD

LIDYINSPSELLKKKPYLGTTCRFQCWGEGAKSGKFTYSSRTGEAIGIFVAGKLHIHL

TSDSPGLLCEVERQVLSWLGKRRTDVLTKEQHQFFLDFLPNLSEVVQKNRDGAILGVT

IDSTNVRMLKYVPPKRNTPVIKIKKQILTVKKQTTLDVESEPRIVWGHGQLSIVYDEC

ETETTYHENLIKVKKLVDLASGTTDKLPTAIFSDTRITLARVKFKTELLLNSLCLLHC

FLKHTSQDAIQEVESKCNVLERYLRSGGVQFRPMSESLDKKVTKLPLQCQSDKDVDKE

INFCEDLTRVFSNENVPLSSWSEVQSYIEEVGFGNVLVHIEKNPTRSDLIWRFSIDSI

SGNFGPIKDIRTLVTYMSTETVPKFLLPFLLFEEQLKHLIAGCVELRDALNSSGINDR

EIAIVALFTCFYYQSDSVKRQGPVCSISSFCSLIGDDLLPLDNRLQARVLPEQDNVKL

HFKLNLTTDSALGKKDKAIQAKKIISRYLRLIFTEDDMDLKRLKSDATKVKLSSEKEC

EFLEFCLHSDLSYALNYRVLLEHLIDLEDRAKKTACVLIEEFILMLTGRLMISSTIDS

DSKRTLEDDALCLEDLLDSDNEASSSKSDNEEQIALQTGKFNFNWDSD

>Crimean-Congo_hemorrhagic_fever_Virus_(from_Homo_sapiens)_(Nairoviridae)_(YP325663)

MDFLRSLDWTQVIAGQYVSNPRFNISDYFEIVRQPGDGNCFYHS

IAELTMPNKTDHSYHYIKRLTESAARKYYQEEPEARLVGLSLEDYLKRMLSDNEWGST

LEASMLAKEMGITIIIWTVAASDEVEAGIKFGDGDVFTAVNLLHSGQTHFDALRILPQ

FETDTREALSLMDRVIAVDQLTSSSSDELQDYEDLALALTSAEESNRRSSLDEVTLSK

KQAEILRQKASQLSKLVNKSQNIPTRVGRVLDCMFNCKLCVEISADTLILRPESKEKI

GEIMSLRQLGHKLLTRDKQIKQEFSRMKLYVTKDLLDHLDVGGLLRAAFPGTGIERHM

QLLHSEMILDICTVSLGVMLSTFLYGSNNKNKKKFITNCLLSTALSGKKVYKVLGNLG

NELLYKAPRKALATVCSALFGKQINKLQNCFRTISPVSLLALRNLDFDCLSVQDYNGM

IENMSKLDNTDVEFNHREIADLNQLTSRLITLRKEKDTDLLKQWFPESDLTRRSIRNA

ANAEEFVISEFFKKKDIMKFISTSGRAMSAGKIGNVLSYAHNLYLSKSSLNMTSEDIS

QLLIEIKRLYALQEDSEVEPIAIICDGIESNMKQLFAILPPDCARECEVLFDDIRNSP

THSTAWKHALRLKGTAYEGLFANCYGWQYIPEDIKPSLTMLIQTLFPDKFEDFLDRTQ

LHPEFRDLTPDFSLTQKVHFKRNQIPSVENVQISIDATLPESVEAVPVTERKMFPLPE

TPLSEVHSIERIMENFTRLMHGGRLSTKKRDGDPAEQGNQQSITEHESSSISAFKDYG

ERGIVEENHMKFSGEDQLETRQLLLVEVGFQTDIDGKIRTDHKKWKDILKLLELLGIK

CSFIACADCSSTPPDRWWITEDRVRVLKNSVSFLFNKLSRNSPTEVTDIVVGAISTQK

VRSYLKAGTATKTPVSTKDVLETWEKMKEHILNRPTGLTLPTSLEQAMRKGLVEGVVI

SKEGSESCINMLKENLDRITDEFERTKFKHELTQNITTSEKMLLSWLSEDIKSSRCGE

CLSNIKKAVDETANLSGKIELLAYNLQLTNHCSNCHPNGVNISNTSNVCKRCPKIEVV

SHCENKGFEDSNECLTDLDRLVRLTLPGKTEKERRVKRNVEYLIKLMMSMSGIDCIKY

PTGQLITHGRVSAKHNDGNLKDRSDDDQRLAEKIDTVRKELSESKLKDYSTYARGVIS

NSLKNLSRQGKSKCSVPRSWLEKVLFDLKVPTKDEEVLINIRNSLKARSEFVRNNDKL

LIRSKEELKKCFDVQSFKLKKNKQPVPFQVDCILFKEVAAECMKRYIGTPYEGIVDTL

VSLINVLTRFTWFQEVVLYGKICETFLRCCTEFNRSGVKLVKIRHCNINLSVKLPSNK

KENMLCCLYSGNMELLQGPFYLNRRQAVLGSSYLYIVITLYIQVLQQYRCLEVINSVS

EKTLQDIENHSMTLLEDSFREITFALEGRFEESYKIRTSRCRASGNFLNRSSRDHFIS

VVSGLNLVYGFLIKDNLLANSQQQNKQLQMLRFGMLAGLSRLVCPNELGKKFSTSCRR

IEDNIARLYLQTSIYCSVRDVEDNVKHWKQRDLCPEVTIPCFTVYGTFVNSDRQLIFD

IYNVHIYNKEMDNFDEGCISVLEETAERHMLWELDLMNSLCSDEKKDTRPARLLLGCP

NVRKAATREGKKLLKLNSDTSTDTQSIASEVSDRRSYSSSKSRIRSIFGRYNSQKKPF

ELRSGLEVFNDPFNDYQQAITDICQFSEYTPNKESILKDCLQIIRKNPSHTMGSFELI

QAISEFGMSKFPPENIDKARRDPKNWVSISEVTETTSIVASPRTHMMLKDCFKIILGT

ENKKIVKMLRGKLKKLGAISTNIEIGKRDCLDLLSTVDGLTDQQKENIVNGIFEPSKL

SFYHWKELVKKNIDEVLLTEDGNLIFCWLKTISSSVKGSLKKRLKFMNIHSPELMPEN

CLFSSEEFNELIKLKKLLLNEQQDEQELKQDLLISSWIKCITACKDFASINDKIQKFI

YHLSEELYDIRLQHLELSKLKQEHPSVSFTKEEVLIKRLEKNFLKQHNLEIMETVNLV

FFAALSAPWCLHYKALESYLVRHPEILDCGSKEDCKLTLLDLSVSKLLVCLYQKDDEE

LINSSSLKLGFLVKYVVTLFTSNGEPFSLSLNDGGLDLDLHKTTDEKLLHQTKIVFAK

IGLSGNSYDFIWTTQMIANSNFNVCKRLTGRSTGERLPRSVRSKVIYEMVKLVGETGM

AILQQLAFAQALNYEHRFYAVLAPKAQLGGARDLLVQETGTKVMHATTEMFSRNLLKT

TSDDGLTNPHLKETILNVGLDCLANMRNLDGKPISEGSNLVNFYKVICISGDNTKWGP

IHCCSFFSGMMQQVLKNVPDWCSFYKLTFIKNLCRQVEIPAGSIKKILNVLRYRLCSK

GGVEQHSEEDLRRLLTDNLDSWDGNDTVKFLVTTYISKGLMALNSYNHMGQGIHHATS

SVLTSLAAVLFEELAIFYLKRSLPQTTVHVEHAGSSDDYAKCIVVTGILSKELYSQYD

ETFWKHACRLKNFTAAVQRCCQMKDSAKTLVSDCFLEFYSEFMMGYRVTPAVIKFMFT

GLINSSVTSPQSLMQACQVSSQQAMYNSVPLVTNTAFTLLRQQIFFNHVEDFIRRYGI

LTLGTLSPFGRLFVPTYSGLVSSAVALEDAEVIARAAQTLQMNSVSIQSSSLTTLDSL

GRSRTSSTAEDSSSVSDTTAASHDSGSSSSSFSFELNRPLSETELQFIKALSSLKSTQ

ACEVIQNRITGLYCNSNEGPLDRHNVIYSSRMADSCDWLKDGKRRGNLELANRIQSVL

CILIAGYYRSFGGEGTEKQVKASLNRDDNKIIEDPMIQLIPEKLRRELERLGVSRMEV

DELMPSISPDDTLAQLVAKKLISLNVSTEEYSAEVSRLKQTLTARNVLHGLAGGIKEL

SLPIYTIFMKSYFFKDNVFLSLTDRWSTKHSTNYRDSCGKQLKGRIITKYTHWLDTFL

GCSVSINRHTTVKEPSLFNPNIRCVNLITFEDGLRELSVIQSHLKVFENEFTNLNLQF

SDPNRQKLRIVESRPAESELEANRAVIVKTKLFSATEQVRLSNNPAVVMGYLLDESAI

SEVKPTKVDFSNLLKDRFKIMQFFPSVFTLIKMLTDESSDSEKSGLSPDLQQVARYSN

HLTLLSRMIQQAKPTVTVFYMLKGNLMNTEPTVAELVSYGIKEGRFFRLSDTGVDAST

YSVKYWKILHCISAIGCLPLSQADKSSLLMSFLNWRVNMDIRTSDCPLSSHEASILSE

FDGQVIANILASELSSVKRDSEREGLTDLLDYLNSPTELLKKKPYLGTTCKFNTWGDS

NRSGKFTYSSRSGESIGIFIAGKLHIHLSSESVALLCETERQVLSWMSKRRTEVITKE

QHQLFLSLLPQSHECLQKHKDGSALSVIPDSSNPRLLKFVPLKKGLAVVKIKKQILTV

KKQVVFDAESEPRLQWGHGCLSIVYDETDTQTTYHENLLKVKHLVDCSTDRKKLLPQS

VFSDSKVVLSRIKFKTELLLNSLTLLHCFLKHAPSDAIMEVESKSSLLHKYLKSGGVR

QRNTEVLFREKLNKVVIKDNLEQGVEEEIEFCNNLTKTVSENPLPLSCWSEVQNYIED

IGFNNVLVNIDRNTVKSELLWKFTLDTNVSTTSTIKDVRTLVSYVSTETIPKFLLAFL

LYEEVLMNLINQCKAVKELINSTGLSDLELESLLTLCAFYFQSECSKRDGPRCSFAAL

LSLIHEDWQRIGKNILVRANNELGDVSLKVNIVLVPLKDMSKPKSERVVMARRSLNHA

LSLMFLDEMSLPELKSLSVNCKMGNFEGQECFEFTILKDNSARLDYNKLIDHCVDMEK

KREAVRAVEDLILMLTGRAVKPSAVTQFVHGDEQCQEQISLDDLMANDTVTDFPDREA

EALKTGNLGFNWDSD

>Hazara_Virus_(from_Ixodes_redikorzevi)_(AJW66841)

MDFLEGITWDSVSDIQSVSNPSFTITDYFEVVRQPADGNCFYHSLAELYIPNKSDHAYRLVKNELREAAE

KYFPTEPEAAATGMRLDEYLDTALRDNEWGGSLEAAMLSRHLGLTVVIWLVDGSNRVVGATRFGKGSLKT

ALHLLHSGLTHFDALRLLATEEDPQQETMTLVEKMELVERFTLTEGEECLQEEELLLDSETDTAAPEEPS

TSEPRLRSQAILLHRLVRHGENIPVRVGRVLDCLFNCKLCVEVSQELLILKPDRKEATAESMSLRQLGHK

MLTRDKQLKSEFSRHKLYLTKDLLDHLDVGGLLRSAFPGKGLERNLSLLHSELVLDICTVVLGTLLSTFL

YGSNNKNKRRFITNCLLGTSLSGKRVFKSLSKLTSNLLYRSPRRAVSIICNDLYGRLIQKLSNCFALMNP

ISLLALRNLDCDNMELKDYIDMVVEMSKLDNSDVDFTHREIADINQLTDRLQILAGRKNPDVLLEWYKKE

ELHKRALRDIAGAQEHLISDFFRRKDIMKFISTSGKASSAGSLGNVLSYAHNLYLSKESLRMSSEDVTQL

LIEIKRLYKLQGDQSVEPIALICDRLEDSFRRLCRELPADCAQECVTLFEDIRNSTSHSTAWKHALRLKG

TAYEGMFSRQYNWRYTPEDIKPSLTMLIQTLFPEKFEMFLDRTQLHPEFRDLTPDYALTQKVYFKRNQIV

EVVSHQISIGETLDESVDAIPLEEKKMFPLPETPVGEVYSIQSILKNFQDKIDRCRDDSKASAEEGKASD

SAGEQLDYNSRIIIDKNNIEISDEEELIRRQLLLVEVGYQTDVDSKITTDYKKWKDILRLLEMLDIKCSF

VACADCSSTPSDSWWISEDKVRLLKNSISHLFSCLTKNSPSDVTDIVVGSISTQKVRSYLKSGSATKTPI

SSKDVQETWARQQEYIINRPTGISIPKRLADAMKQGFVDGVVMSADSSKECVANIKKNAERLTDEYERTK

FKHELNYSRVTSEKLLLGWLGEDLQGIRCDNCLHTIKETVEQMNENCDRLEYLASSCLLSNHCSGCHPSG

IALNNQTNVQKRLPEMGLLKHSENKGFEDTNEAITDLDKLVRLTLPGKTEKERRVKRNVELLIRQMMQQS

GIECIKLPSGQIVTHRLTRKNKQAPSTQECERIEERVTKLKKELSEKKLSQYSKHINSTIAHSLERLDRQ

TGSRCSVPKEWLEKLLRDLKVPTKDEDILIGIQRSMQEKVGFTVNNDKLLIRDEDDLVRFIESRSKSLLE

TNEEGIFQSDCVLFKEVVAEAMCRYTSTPYQEIPETLVKLINLLCKFVWFQECILYGKVCETFLRCCTEF

SRSGIKLVKIRHCDANLAIKLPSNKKENMLCCIYSKDMELIKGPFFLNRRQAILGAAYPYILITTYVQVL

QQHRCLEVLNNHGPRILENISRCTKTLLETATKELSFTLRGLFEKAYETRTKQCQLGGNFLSRSSRDHFV

SVISGLNLVYGLLIRDNLLANSQQQNKQLQMLRFGMLCGLSRLSCPKELGKKFSASCRRMEDNVMRLYLQ

STVYSANRDCEMNVSNWKLKDLCPEVTIPCFSVYGLFVNSDRQLIYDIYNVHIYNKEMDNFDEGCINVLE

ETAERHMMWELNLLETLNPNTKDDRTARLLLGCPNIRKCTGKDGRKIRPLLHDSESADSSSESSTISGRR

SYGSSKGKIQSMFGRYNSNKKPFELKPGLEVSNDPLHDFQQVVTGGSAYSEYSPNQDSLLKDYIQIIRSN

PGYTMGSFELIQAVTEFARTKFPAEGIEKARRDPKNWVSISEVTETTSIVATPKVHMMLKDCYKVLLGTE

NKKIVKMLRGKLKKLGAISTDVEIGKKDCLDLLNTVEGLSEEQKKNIVNGIFEPSKLSFYHWRELIKKDL

YEVLLTDDGNYIFCWLKTLSSAIKGRLKRDLRFMNQNSQTSSQTDLFSEEEYEELLKMKQVICSQSDAED

ELNVDVLLDAWVKCAHKPRDASSIINEGMSRVLPISEILFELRMQHLELTKLKKDNPSVSFTKEEVTVKR

MEKQFLAKHNLDIMHLTNLIFYCALAAPWCVHYKALEAYLVRHPEILEFSGSAPTESKVLDLSVAALIIK

MTENYRDDTSDGVEVKVRFLVRYIITLFTANGEPFSLSLSDGGLNEDLQKTTDEKLLHQTKVVFAKIGLS

GKNYDFIWTVQMIANSNFNVCKRLTGRSTGERLPRSVRSKVIYEMVKLVGETGMAILQQLAFAQALNYDH

RFYAVLAPKAQLGGSRDLLVQETGTKVIHATTEMFSRNLLKTTKDDGLTNPHLKETILNAGLEALQTMRL

VDGKPAAEGSSLVTFYKVVCISGDNTKWGPIHCCSFFSGMMQQLLKDVPDWCSFYKLTFVKNLCRQVEIP

TASTKKILNVLRFYLSDKGGVERLSEEEIRNRLCETLDLWGGNDIVKFLITTYLSKGIMAMNSYNHMGQG

IHHATSSVLTSVMAELFEELTVDYYKKHYPNLSVSVTHAGSSDDYAKCIVVTGLLSKDLFDKYSETFWMH

TCRLKNFTAAVQRCCQMKDSAKTLVGDCFLEFYSEFMMGYRVTPAVIKFIFTGLINSSVTSPQSLSQACH

VSSQQAMYNSVPLLTNATFTLLRQQVFFSHVEDFIRRYGLLTLGSLSPFGRLFVPTYSGLVSSAVALEDS

ETIARSSSTLVENSIFLETSSLSIIDQISSSSSSEGEGFSTASSTTVESTHSASSSSSFTFELNRPLSET

ELQFLKTLRDNSRQTSSEHIQDKITELYSTSKEGPLDKYYLLYSSKIVDSCSWLKKGREKGPIECAKRLQ

CILNVLIAGYYRSFGSDGTEKQVKACLNRDDNRVIEDPMIQLIPEKLRRELERLGVSRMEVDELMPATSP

DDTLCQLVAKKLISLNVSTEEYSAEVSRLKQTLTARNVLHGLAGGIKELSLPIYTIFMKSYFFKDNVFLD

LDDRWSTKHSTNYRDSTGRLLTGRVITKYSHWLDNFLNCKVSVDRVQEVRDCSLFNPDLRCVNLLIGENK

VRELSIVVSHLKVFAREFDNLNLQFSDLNRQKLKIVESRPPESELEANKVVIVKSKLFSATEHVRLSNNP

AVVMGYLLEESSISEVKPTKVDYSNLLKDRFKLMQFFPSVFSLLRTLQVESRETEKLGDPVDMNLVSRYS

NHLTLLCRMIQQARPSLTVFYMLKSTHLATEPTVSELVSFGVKEGRYLRLSDSGLDASTYSVKYWKILHC

ISAIGELPLSPKDKTTLLMSFLNWKVDLESCEQDCPLYKNELSVLSEFSGQVIINTLASELSSVRKDGER

DSLTDLIDYVNSPSELLKKKPYLGTTAKFSSWGDSNKSGKFTYSSRSGEAIGIFIGGKLHIHISEESTGL

LCEVERCVLSWLSRRRTDIITKEQHGQFLAFLPTLSEVAQKNRDGGVQGVCVDPSNPRLLRFTVAKRQSP

VIKVKKQILTVKKQITYDAESEPRLQWGHGSLAIVYDECETQTTYHENIIKIKQLIDNTVDKGKILPQSV

FSDTRIILAKIRFKSDLLLNSLCLLHAFLRHTATYAVMEVESKSQLLEKFLRSGGVQFRSCCTSIKDKLK

TKELDGLITQTLDEEVAVCDELNKVFSEAQVPLSSWSEVQTYIEEVGFNNVLVNVDKSPAKSELLWRFSL

DSHVSNLGMIRDLRSLVGYVSTEAVPKFLLPFIFFEGLLVSIIGKCKTLKELINSTGTTDRDIDILLCLI

LFCFQNDSFAREGPRCSASALKQLQCCNVVRVNNRINIELVSEGSNVSLKVVIVLIDATESTLERSKRVK

VAKRNLSSSLGLMFFDKSIDVTGLKRVASRVKVSSDKEKEFLDFVLPSQSASEVDYQTILDLTIDKAKKG

RTVHGIEDLLLTLMGKAGTPKSEDEEDLIQDSTEGHLCLEDLLEDTSVSDPRAESEDEEQIMKRGFKFNW

DSD

>Leopards_Hill_Virus_(from_Hipposideros_gigas)_(BAP90965)

MEALGDLVWEELYEGSYSNVNKFQFNNFFDVDEMPGDGTCFFSSVSKYIFNTTELWQTVKSTCANYARAH

WKEVMEMDRQYAEADAYISDLMRDQYWGGSIEAEILSKALNMTIYIWVSGDGVWVSNARRWGGEPIQTSL

NLIHVHGGHFNLLLPKYLQPVQVREQMSYEEKVNVTVSAILDNEGSAETLAQELTENKSSFQDELNEELK

AMAELKFSLFEKKHDIQLEKERAKPEKTTKSELDWRRKVQDKKNIPVRVGKVLNNLFNAPIEAVFDDGNL

LLYPRIQGKLRPKAFSISALGHRILDGDKSFLNACTRLLVVVTSELMSFLDASYLLRLVAPGSGLSQIPG

VVHPGIKVDMCLCVISILISSFLYKAKPKLKRNFINSANTGSSVDKQLLLKGLLKLNNRTLYNTPYKLVQ

EVSVPLFKESMDTIAEALRLMNAQSKVAFFCLDLNGMRRETYFNLLEELKTQDLEDKGFVSEELKELHSC

VLLVVKLSKIADKRNVPDDVLEDVRQYLGSDGKIFRKNTTKKRNSSELAQECITVFFRRRMIFKFVSLRG

KAYSGASVGNLIAYCHNLYLSRESLNFTEEDTEQLSIEIRKLNELLSTEPKKPIALICAELYKPFQELFA

ALPKDCSEECQTLFEDVRNSESHASAWSSALRIKGVAYEGFFSMSNGWRYIPEDLKPTLGMAIQTVFPEK

FEKFLERTHLHPEYRDFTPDYLMCRSKVFKSDKVTKKVDSQGDPLVKKPNHAEADDDIQYAKLSSHRKRF

PMPEIAVQEVSSVSAIMDRFKLKSSEKGRPIRQEESKPSASTEKGDIEIDELLIVEVGYQTDIEGKVVSD

MEKWRGVINLMTHLGIKVNIITCADNSQTPRSDWWIEEKYVKLLLNSISYLFKELLSNSPSEITDIAVGN

ISTQKFRSVLKSGSIVKTPVTLKEVYDAWKVAKPHIMCRPTGTNLPEHIAEAIEVSLVEGAIMNRQSAED

VLSRIEKESGNIIKEFERTKNRHELNKNEVTAQKILFGWLMSDLNNACCHECMVDIKDNLNKIESHAEKL

SYMCLMLKPNEKGCCRPSKKSLYEVSSFERRMPPLDHINHKELNIGVEEQKGTMLDAIVKLTLPGKTEKE

RKLKRSVEQLIRSMMRHSKVQAIKLPSGQMLVDSSFTTQATLDKEEGKSFKKGGTKTLLEDKEHFDKVLA

PSKLMQYSEHVKSVIKHSIQRMDCCKGSLCEINPQWVKNVHYDLGSDVSDDNILAKIQESYEKKANFEVK

NDKYRCIEWPAIEEYLHNKLNSHRLSDNKVFKLDCILFKEVYTELSVRLRETPYNSCPSLIASLLKLLLE

FQWYQHHVLYSKICESFLQSCSEFHRAGIKVLRVRHTDVNLVVALPSNKKQNMRCVVYSKDFQLLQGPFM

LNRRQAVLGAAYPYLITICFLQCLQHYRCLDQVQSSSEEVRAKIVRKVEMLGEVSLGLLKDVYHGAFMSA

SSNLLRLCKESGNFLNRSSPDHFISVFSGLTLVFDVLLGDSILNNSQPFNKQIQMMRFGMLSGLSRMSCP

TELGKKFSSSCRKVEFHVSRLYMQLVVFACNHKVKSNLENWVKGDLCPEINMPCFSVFGTMINSDRQLIF

DIYLVHIYNKELDDFDEGCIKVLEETAERHMSWETEVKEASKKIEAGQEARYYKRMLRLLLGVPNLKRMD

ITSSEGTESLSVSERGSTDSRLSNVSSKSARSFRSLRYRPTSIYGIRSSQDKPFSLNEVLEVSRDDQRDY

QQAVTDRGMYHTYQANPESVYKDVVTCIRHNPNHTFASYELVQACTEIARVRFPPESIERARKDRKNWIS

ISEVTETTSIVAEPRSVIMIKDAYQIILGAENKKIVKLLRGKFQRLGMECKAEMKGKTRCQELLTTIEIL

TDNQLDSIVKGITDPSKLTFYNWRDLVKMKIKDVLLTDDGNYIYCWLKSLAQAVKGSLRTEIKGLKYNTM

GTKSNINKTKTLEDAEFTAVSSFINFLKACAAGEINSDLDNKEISLDSLLSSWLKFFRKVRNSKEVEGEG

LSSLAKMAQDIKKMDLSYRTLCQLKKELPDLSFSREEIILRQLEKDFIKQHGDNILRLSNLIFLICLSCP

WCVQYKTFESIMMRNVAEAPGFNLPKSSTSLRELHPDSIILMMIREACLDTSDEEAILCTKYCMCLFTVN

EMPYTSAMNSHGDFVYKSPNEQLIGRVKGIMAATGLDDSRSDFKWTVCLIANSNFEVARKITGRSNGERL

PRSVRSKVIYEIVKLVDSTEMAILQQLAFSYILDPNHRFFAVLAPKAQLGGHRDLLVQETGTKVIHAATE

MFSRTLLSTTKDDGLTNNHLKETILNCGLEAINQFKIIHGKELSKGSGQYYFYRVCCISGDNTKWGPIHC

CSIFSGMMQQLLRDYDDWTSFYKLTFLKNLFRQIEIPAASIKKILNSFRYKNKDIKVDQLTETELRDEMA

NRLQTWKGNDIMTFLVENYVSKGKMALNSYNHMGQGIHHATSSVLTSIMAEINERLIINFCGQRLPDLQV

TVAHAGSSDDYAKCIVLSGVLTETLMENYEEAFWPTMCNLKNYLAGFNRACQMKDSAKTLVSDCFFEFYS

EFMMSQRITPAVIKFILTGLINSSVTSPLSLVQACQVSSQQALFNSVPLITNIAFTIFRQQMFFNHTEYF

SRVYGPITLGTLSPFGRLYVPKFSGLISSSLAIEDAEEVVKSCLEMKRLLLHLPDGTRAELDSASDDSTD

KVIEADVSVESESVDTSSLSSAPTDSSGASFHFGIMRSLTSAEEEYSKVISQQFTGGVLDKVEEEMMLTY

QDNQDYPIAPCISKLLKSGMVISNVHLKDLCDKPLRLIKIVTAVLNCLIAGHYRTFTSEGTEKSVKAHLN

RDENRMIEDPMIQLLPEKLRRELNRLGLARMEADELIAKPDIKDTLSNLIANKLITMNCATEDYKSEVLR

LKQTLTSRNVLHGLAGGIKELSLPLYTIFMKSYFFKDLVFLEHKDRWNSKHSTNYRDSTGKVLDGKVVVK

YTTWLDCFLSSELSLNRSTPIVSDSLFDPGLKGIEVIHKSNGTFELQVLPEEIEVIESETRSLAIQFTDV

NRQKIKVAESRPAKHELDAHKAVIVKSKLFSAIDQVKLVNNPAIVVGNLLDETSLFQTKPRIDMGNLGRD

SFKLTQFYSSLVNLIREINNESETLRKEKIVPSAELVNRYANNLTVLCRMVQQARSKLTSFYMMKGSHVN

NEPTVVELLNYGIVEGKFYEVQDEIADMSSYSVKYWKILQCVSAISVMPLKDNDKTALLTSFLNWKPSVN

ECDSDCPLSRREKSVLQEFNGRVLVDILSSELPSIKNEVQRKSIEDLVDFVNSPMELLRKKPCLGTTASF

NCWDNGGKRGRFTYSSSTGDSSGVFIGPYLYIVLSNNSPALLLQVEKKVLEWLNKRRTDVMTREQHEYFL

ELLPDYRIFPKKVGDGKIFSLKPSREDPKLMCFLSPKNGDKVVKVKKNILSVKRKAADEPLGEPRAVWSN

NNITILYDEQVEKTDYHQDLVAIKNLLDDVLGSDRSKVPSSVYEDTRIILSRIRFSADLFLKSLLLLHHF

LAHTPSAAVLQAQSKTNLLKFIESSAVSNCQIGSLGKKIQKSVLSMIAEEVTGISSKEELICAKLTDALR

KKNFSLEAWPEVQSYLDETGFSNIQLEFLQRGTTDTYDWKFKVIQEVSLSQRSQGVRGIISSISSEAIPR

FLAPVIIDGILTHNIVQMFINCRNVISRTGISDTELDGLVCSIVFCSQSLPIVREGHRFSFNNLLHLATK

KTFATADGSISCTFQNNGDEVQLAVKVRTVKPDELTESKKRRIELTKMRILTAYNSFLPPVTSVSEILSH

VEGAYEDPVKNTGEYFNLDLGRKTSEGCRTRDLWELICPGTSWRRNDFESLMQIISLLSATKKAEVCEEV

SVEDPRMFSAEQVTYLDLFEDSPSAEQGDSKVMEEPVGASFTFNWDD

>Tacheng_Tick_Virus_1_(from_Dermacentor_marginatus)_(AJG39253)

MAGRRRSSNLSESLISDCDNLGNFYRGPIVLDINKEFTIEDVPGDGDCFFHCLAKQLPEVSVSRLKGIIT

SYALRNWDTLTEAPRFYSDPKDYERELNRAGYWGGTTEAEIINHSFGVPVVIWTTEDKKLTSAVQVWTRK

HGNLPELHLLHTGTHFMCLAPIVKEGTPPRELVIEEEGVEIVSNTENDTIENLEDIFLPHHERERIELLS

STSSPSTSSHKTVPKRLLMLLEQEKTLPLRIGRLLSRLFPCNVKAQRFRGDVFVLVPEHQSGRDMVSLSD

LGRVMLQRRQTHHLISRYNISVSEELLAFVDSQFVLTTIVGSSLLANNAAILPTEMVNRIATICTVILMS

SFLYKASMKVKREFILVNLANSGVPTKNVKKYVHGLRPFAIYQRPLETAQTVTLLALGKEIETISLKVSR

MSSSSYLLLSCVDLTGKTTKEFNKLLDELENNEEELAISSSEVQDVITTTHSLEKLFELKCSGAKRLQFR

DVLASAGLNPDKKLLGESNIYHRCKSLVLEAFFHSRNIMKLVSKHGKAYAGTSITSLMSYISNLILTREK

LGLTTEDVAALETVERRLMSIQSRDRRIPIPILCSLVETKMEELFSALPDDCSQECRMLFTSIRNSETHS

TAWSSALRLKGVAYEGLFAKTYNIRYVPEDQKPTLSMAIQTYFPEKFERFLQRTQLHPEVREFRPDFLLA

RKPLFQHSLNLKRPHQVVQVFATGTETEGASIIKRKERAFPLPEVPIDEPSCFHSVGPKLREIAVRKLQK

YQVESGKVMSTEEGDIDLLSGAEAQKATGVDPVPNEDLPGQGISLEEMVAKEQQPTEFLILEVGYQTDTE

AKVQTDMNKWKIAVSLLKDLGISSTVIACSDSTGTKASDWYIPEQLVTVIKNSISNLFSKLSQNSPQEVT

DMMVGAISTQKIRSVLKSGSSIRTPVTIQDILTTWNENKQFILTRPTGAQLPKKMENIMKVALCEGAVVS

REFARKVVDEVIQQKEQIADWVEQTKYAVECLEPVSSAEKVLEGWLLDDLEQCRCQTCITQIKKTIKETT

SVHERLEYISHQLNPDQHGSCCHTIPIQRSTIDRLSRRTPTLSAVKHLETEISDLEGRSTALDRLCRLTL

PGKTEKERSIKRGVESLMRQCMKDSGISCIKLPTGQISVNTELFGTRKGKKPVMKDGDKERLERLRRVLA

PEKLKTYSELVQKTISEALGSTDRQQGSLCEMKPEWVRRVLADLDADDTEAELLTKLESTRESKLNFSVN

NDKLQPCTDSEIKEYISFKSTSLLNLGSTRGPYKLDCVIHKELFLECVRRYQNTPYFDCVNIIGELTKFL

LQFDWFQEQLLYSKVCETFLQSCTEFNRSGIKIRRVRHTSTNLAIALPSNKKENMKCALYDTNFLLIGPR

HFMLNRRVAVLGAAIPYIQIICILQCLQHSRCVEMVHEANFNTVQEVLTRNASNLDVIAECLVLINQGKF

ETASRTYLKHCRQSGNFLNRSSKDTFVTVMSGMSLMFGLLLGPAMLLNSQPFNKQLQNMRFGMLYGLSRV

ASPKELGKKLSSSSRHIESYLSRLYMQLVVFCSGLDPPHNIKAWKQHDLCPDTTIPSLSIPCCLVSGDRQ

LIFDIYLVHIYNKEMDNFDEGCIKVLQETLERHMTWEMQVMDSCNKYSKGKQAERLKESRTLRLLLGLPN

LKLASEYRAEESKGSESDSLSTASSTHSIGKTRSFSSGKKIRSIYGRLRSTMRPISLQEGLEMVTDEMSD

YQFIAQDTGMGVKYEPSADSVMKDIKQIIHDNPSHTYGSYDFVQCMVEIAKVKFPPESISKAQRNATNWQ

GVSAYTETTSSVSEPKTRIVLKDALKVLTGGETKKTVKLIRNRLKKLDGGASQKPEKLSELADLICTVET

FSDRQKEEIRRGISEPSRLSFFPWHEIVNKPIKDVLITNDANMIYCWLKSLASGIKKAVKPYMPALRYCK

ETKVRDHPKLARLLSPDEMLSLNQLMDTFSSLVKGQADPVGVVPPLSDLLDVWVRFLEAVGFMRAILEDG

YDSIENCLDSFKELSVAYKTLIDTKKEYPELSFIREEIHVKNLEQCFLKDYEREVMRIINLIFSVSLACP

WAIHYKSFELLLSREDSPLSEHELQNQQLEVLRALGPCTVITSRLAKFLNREEFRDGDLPYIEGLYRYCC

ALFTANYEPMKAVLELKEVIVTSHVEESALSTTRSLLAKYGLEKTDLDFKWTLNLIANSNFEVTKRLSGR

TEGEKLPRSVRSKVIYEMIKLVKSTGMAILQQHAFSYILNSGHRFFAVLAPKAQLGGHRDLLVQEIMTKI

VHAASETFSRSLLSTTDDDGLTNQHLKESILQSAYDQMQSSKASHGRPVSDSPGGLRYFSITFTISGDRT

KWGPIHCTAFFSGMMQQLLQDAPDWNSFYKLVMMKNLYRQVEIPSGATRKILNAFRLHLSTKVDLDSLTE

DQLRRLLLENVDIWKDNLMIQFLVQAYFSKGKMALDCYNHMGQGIHHATSSVMTSCMAVLTEEVIHSYFQ

VHMPELSTTVKHAGSSDDYAKVVTVSGCVPASLFERYEERFWKHVSRLQNILIGIARACQMKDSAKTLIG

DLLCEFYSEFMLFHRVTPAVIKFILTGLINSSVTSPQSMIQACQVSAQQAMFNSVPLLTNICFSLFRQQM

FGNHTELFQRKYGPLVHGLPSSFGRLYLPMFSNLTSSAIAVEDAESIALDLSSALELCDLLPQAPEPDYS

HLALPSPEVSEASSDYGSRELSSDQEGTGSVSSGSTSSFRFGEISKFTPTELEYLRTSSRLDSQEAERAV

FETVVRMYANHSDFDGWPCLDKLASSTLVDSNIDLRRVLNENPVRLLKYVRSLISSLVIGYYRCFSSEGT

EKSMKANLNRDENRIIEDPMIQLVPEKLRRELNRLGLARDDYEEYVSRSTSGIPLVDQVARKVITMNCLT

EDFEAEADRLKQTLSSRNIIHGLAGGIKELSLPLYTIFLKSYFFIDKIFLDHKDRWNSKHSKNYRDSTGG

SLEGRVVTKYMVWLDAILSSCASRVSTKKTEPCSLFNPNIKCIELLTFQDRTRFLVLKTEDMKAVRDELR

ALSVQFSDQNRLKMKILESSRPLCEREANKVVISKSGLFSAGEQVKIRNNPALVIGFLLSKEVVLSVKPS

KMDLSSLIVDTVKLEQFYTSISEVCARIQEESNALERLGESPRAEDVTAHSNTLTLLSRLAQRSNSRIVS

FHMIKPMSTHTESTVSDLISYGTKEGRNIVLAESSVETGTTSLKYWRILQCLAAISALNLSNTNKTNLLQ

GFMNWVPRSNNIPEECPMKKHEATVLEQFKDRSLVSSLYEELPSIKNESDRKQVECLVDYVKDPMVLVAK

KPFFGKTVDFNTRGGELPRTGSFTLSSSSGEAVGTFVAGNLHIHISKDSDILLTEVESYVLRWQNKVRSD

IVTKEQHDYFIELLPSYLGLPRKLAEGILKSVSIDRSNPRMLHLSTCKGNCKVVKVRPHILTVRKTSEDA

RLNEPRLMWGKNSISIIYDEHTEEATYHESILSLRRKLDITLSAEQITIPKKFYSDMRVVLGKVQLKPDA

TATSLSLLHHYLVHSAPQARIEFHSKTALLERMLGAEAMELNMLRLIRESSLSKEACLRDGTEHLVTVAA

KVQDTLNSDGTPLHCLSEIQQYLDETGNSAIKVDAEIKGMHTSFIWKCTVDSPGMRRPSSDLRTVINTIG

TESLPLQFAHIIADAELWVRVQNIGKMAQRQMVESLLPDGDIESLFCSALYCCQNKDRNRENFTFSAVSL

LNLIGERKFAVSGIAEAAVVAEEDGSLKVKATLKVPEISASDKQSIRRLNQAAVTVNNALMSRPRTFAEI

RREVSLDLVHLEGSTQLTLNYSSDFCTETYLERLFYAFTGQDSEAIRKLKDVVNLACVLLGVESPVEAFN

EEEQQMSEASGDKLATLDDLDIEDATPELGNNDLEDFRFDFS

>Rift_Valley_fever_Virus_(from_Homo_sapiens)_(Phenuiviridae)_(YP003848704)

MDSILSKQLVDKTGFVRVPIKHFDCTMLTLALPTFDVSKMVDRI

TIDFNLDDIQGASEIGSTLLPSMSIDVEDMANFVHDFTFGHLADKTDRLLMREFPMMN

DGFDHLSPDMIIKTTSGMYNIVEFTTFRGDERGAFQAAMTKLAKYEVPCENRSQGRTV

VLYVVSAYRHGVWSNLELEDSEAEEMVYRYRLALSVMDELRTLFPELSSTDEELGKTE

RELLAMVSSIQINWSVTESVFPPFSREMFDRFRSSPPDSEYITRIVSRCLINSQEKLI

NSSFFAEGNDKALRFSKNAEECSLAVERALNQYRAEDNLRDLNDHKSTIQLPPWLSYH

DVDGKDLCPLQGLDVRGDHPMCNLWREVVTSANLEEIERMHDDAAAELEFALSGVKDR

PDERNRYHRVHLNMGSDDSVYIAALGVNGKKHKADTLVQQMRDRSKQPFSPDHDVDHI

SEFLSACSSDLWATDEDLYSPLSCDKELRLAAQRIHQPSLSERGFNEIITEHYKFMGS

RIGSWCQMVSLIGAELSASVKQHVKPNYFVIKRLLGSGIFLLIKPTSSKSHIFVSFAI

KRSCWAFDLSTSRVFKPYIDAGDLLVTDFVSYKLSKLTNLCKCVSLMESSFSFWAEAF

GIPSWNFVGDLFRSSDSAAMDASYMGKLSLLTLLEDKAATEELQTIARYIIMEGFVSP

PEIPKPHKMTSKFPKVLRSELQVYLLNCLCRTIQRIAGEPFILKKKDGSISWGGMFNP

FSGRPLLDMQPLISCCYNGYFKNKEEETEPSSLSGMYKKIIELEHLRPQSDAFLGYKD

PELPRMHEFSVSYLKEACNHAKLVLRSLYGQNFMEQIDNQIIRELSGLTLERLATLKA

TSNFNENWYVYKDVADKNYTRDKLLVKMSKYASEGKSLAIQKFEDCMRQIESQGCMHI

CLFKKQQHGGLREIYVMGAEERIVQSVVETIARSIGKFFASDTLCNPPNKVKIPETHG

IRARKQCKGPVWTCATSDDARKWNQGHFVTKFALMLCEFTSPKWWPLIIRGCSMFTRK

RMMMNLNYLKILDGHRELDIRDDFVMDLFKAYHGEAEVPWAFKGKTYLETTTGMMQGI

LHYTSSLLHTIHQEYIRSLSFKIFNLKVAPEMSKSLVCDMMQGSDDSSMLISFPADDE

KVLTRCKVAAAICFRMKKELGVYLAIYPSEKSTANTDFVMEYNSEFYFHTQHVRPTIR

WIAACCSLPEVETLVARQEEASNLMTSVTEGGGSFSLAAMIQQAQCTLHYMLMGMGVS

ELFLEYKKAVLKWNDPGLGFFLLDNPYACGLGGFRFNLFKAITRTDLQKLYAFFMKKV

KGSAARDWADEDVTIPETCSVSPGGALILSSSLKWGSRKKFQKLRDRLNIPENWIELI

NENPEVLYRAPRTGPEILLRIAEKVHSPGVVSSLSSGNAVCKVMASAVYFLSATIFED

TGRPEFNFLEDSKYSLLQKMAAYSGFHGFNDMEPEDILFLFPNIEELESLDSIVYNKG

EIDIIPRVNIRDATQTRVTIFNEQKTLRTSPEKLVSDKWFGTQKSRIGKTTFLAEWEK

LKKIVKWLEDTPEATLAHTPLNNHIQVRNFFARMESKPRTVRITGAPVKKRSGVSKIA

MVIRDNFSRMGHLRGVEDLAGFTRSVSAEILKHFLFCILQGPYSESYKLQLIYRVLSS

VSNVEIKESDGKTKTNLIGILQRFLDGDHVVPIIEEMGAGTVGGFIKRQQSKVVQNKV

VYYGVGIWRGFMDGYQVHLEIENDIGQPPRLRNVTTNCQSSPWDLSIPIRQWAEDMGV

TNNQDYSSKSSRGARYWMHSFRMQGPSKPFGCPVYIIKGDMSDVIRLRKEEVEMKVRG

STLNLYTKHHSHQDLHILSYTASDNDLSPGIFKSISDEGVAQALQLFEREPSNCWVRC

ESVAPKFISAILEICEGKRQIKGINRTRLSEIVRICSESSLRSKVGSMFSFVANVEEA

HDVDYDALMDLMIEDAKNNAFSHVVDCIELDVSGPYEMESFDTSDVNLFGPAHYKDIS

SLSMIAHPLMDKFVDYAISKMGRASVRKVLETGRCSSKDYDLSKVLFRTLQRPEESIR

IDDLELYEETDVADDMLG

>Toscana_virus_(from_Sandfly)_(CAA48478)

MERILKKQPAPVRALTIHPLRRYESSIYDTPIPAYVIKHSSDGVTIDIATSELADGQSGSTIQPFESVPA

QNLTLFKHDFTFGHLADTTDKKFVEVFGVLENRADDSDFQSPDMIIETETGHVYVVEFTTTMGDANSADL

AARNKIAKYEIACLNRSAIKPISLYIIAVHFNGVISNLDLSDEEVNEIVFRFRLARDIFEELREINPALF

DSDETISRLEREVNSVMSAIQIDWDTTEKKFPSFRRELFENFRSKEVDDEYISKIIKRCTDEALRGIERD

SLYTEDITNKERFELNSKRAASDIKNKMAEMMSYEFLRDTEDHKSTVQFPPWVTRTGPAGKDLEPLKSVS

VEGSHPMCKIWNKVCTNASIEKIERMHDDPVLELEYAMSGSTERSVERNKYHRTVLTLSPEEREYAAVLG

VCGKRNANLGAVKEARVRSKKGFSIGHNTERVEEFLSDSCVEDLIPTEGLYNPLSEDKSLRLLAMGLHQP

TLIHMDDETPETLDCHLKFLSSPIGSWLQMVSIVGAELSASVKQHVKPNQFIVKRLLDSAIFLLIKPTTS

KGHIFVSLAVNKKFLHGELSKSSVFKQSIDAGDLLVTDFVSFKLSKITNLCKALCVLEAASCFWAETYGF

EPWKFVDQASAVKFLDAWFMIKLSLLTMLEDKATTEELQTMQRYVIMEGFVSLPEIPKPHKMLSKIPKVL

RSELQVFLTHRLFSTMQRISATPFQLHKVGGNIRWKGLFNPYSGNSIDELQTLISCCYNGYFKNKEEDTE

PSALSAMYKKIIELEHLRPPTDTYLGYEDPIDPKMHEFSRSYLKLLCNHAKTKLRKQYGRGVMNQIENSI

VREVQSITLERLATLKATSNFDDSWYTFKDVKDKNYTRDKLLVKMTQFAHRGKTLAIEVFEECMSRIEEK

GCMEICLFKKQQHGGLREIYVMGADERIVQSVIEAIARAIGRFFDSDTLCNPSNKIRIPETHGQRAKRRC

GRSVWTCATSDDARKWNQGHYVTKFALMLCEFTPQEWWPLIIRGCSMFTNKFMMMNLDFLRIIDSHKELQ

IEDEFVSKLFKAYHGESVEPWISQGCTYLKTSTGMMQGILHFTSSLLHSLHQEFVKTTAIQLFTLKLGSD

ASSKVVCDMMQGSDDSSMIISFPSYNEKIKMRYKLVAAMCFRIKKSLGIYIGIYPSEKSTPNTDFVMEYN

SEFFFHSQHVRPTIRWIAASCSLPEVETLVASKEEAANLLTAITEGGGSFSLAAMIQHCQSSIHYMLMGL

GVSALFSEFSKAISKWLDPGLGFFLFDNPYSAGLSGFKYNLYRAIMNSSLKSIYSFFMKRVKGGSQRTDG

IISESCSVSPGGAIVMSSTLRWGSVEKFKRLRNRLNIPETWKEMINESPEVLYRAPQTGTEIMLRIAEKV

HSPGVVSSLSTGNAVCKVMASSVYFLSACIFEDAGSQEYKVVNNDKYSLMQKIIAFDQIGCNDEISQEDL

LFLFPNLAEFEAFDSIIYDKGRFNVIPRASQREATQTRIVVFEHHSSARVAPEKLVSDKWFGTRKSKIGS

PGFRQEWDRLKAIVRWLRDTPEETLDSSPFSNHIQIRNFFARMEGRPRVIKVTGAPVKRDLGMSKIAMAI

RDNFCKTGFLQGLEDEVGHSRAMQVEKIKHYLFSVLMGPYSEEAKLEYVVKILKEEPQVILNYNDKRSRA

NIISLLQRFIKSEIGIATLIEDMKAGVFGAFVKAQQFSQSSVNNKYYGRGIWKGVMDGYQVQIDIDGKEG

MPSHLSGITISNCSKTWILTQSLKAWCEDMQVYNNTDVSKANPKANYWMYGFKMYGSSYPYGCPIYLVRH

DITNLGLLHDDDIDIKVRRNTINLFVRSKDKRPRDLHILSYTPSDSDISSVSSKHIMEDEYFVYKGAFSV

EPTRSWMLCQPLPWSFVRPVLQVATGSRRSPRQLDLERLREIIRLCTESSIRNKVGTVYGQNRPEKFIEA

EPIDMSEMFDMMLDEGMDDAFEELADYLTVEEDPDYMDEVSFDDDSLNLFGPAHYKELQSLTVLAHPLMD

DFVTRLVGKMGRPQIRRLLEKNVTTRDLRELSELLFMALDRDPSQIREELILGDSPTEVPDDLLG

>Nairobi_sheep_disease_virus_(from_Tick)_(AIZ00432)

MEFLNSIPWEEVVPGQFTANPGFQVTDYFEIVRQPADGNCFYHSIAELFVPNKNDFSFRLVKQHLELAAR

RFFEEEPEAKGLGLGLEKYLEVAMCDNEWGGSLEASMLAKHLDVTIVIWVIEGPSRVAAAVKFGSGDVAG

AINLLHTGYNHFDALRLLVDDSQVSRQPRDITERIEIVEEVLSEDREETFFEEDLLNFATVETIEEKLTR

QDKKVQDEMQRRAVLLGKIIKKGENIPVRVGRVLDCLFNCKLFVELRDGLLVLKPESKEDPGSGCSLRQL

GHRLLTRDKQIKQEYAKSKLYLTKDLLDHLDAGGLLRSAFPGMGLERNLQFLHSEVLLDVCTVVVAVLLS

SFLYGSNNRNKKTFITNCLLNTSLSGKRVFKALGKLTGTTLYKSPRNALSHVCQTLYGKMMGKLQSYISV

MSPISLLALRNLDFDNMSVKDYMEMLKEMSVIDNTDVDYTHREIADLNQLTDKLQKLFKEGRADELKNWY

KEEELTKRSLRSVQNASEFLISDYFKKKDIMKFISTTGKASSTGNIGNVLSYAHNLYLSKESLKMTTEDT

TQLLIEIKRLYRLQGEQSIEPIAIICDKLEEQFRRLFKELPEECSLECQTLFNDIRNSGSHSTAWKHALR

LKGTAYEGMFSRQYGWSYIPEDIRPSLTMLIQTLFPHKFEEFLDRTQLHPEFRDLTPDFALTQKVYFKKN

KIVEIQNTQLVIDSSLEGSVEAVPVVEKKMFPLPETPVDEVHSIQRIMKNFRDKVERDKRKKEEEGDRAP

ADEHMEDQKSGNVQDPDKPAHTTKEAIQEKLEETEVPKASPESSGADKLQPALVPIGSRDKAQSAARGPL

TYSDRILIDENSTELTEEEELEKRQILLVEVGYQTDVDGKITTDFKKWKDILRLLEMLEIKCSFIACADC

TSTPADNWWISEDKVRCLKSSISHLFSSLTKNSPADVTDIVVGSISTQKVRSYLKSGSATKTPISSKDVK

ETWQRMRQNIIERPTGAVINPGLESAMRQGLVDGVVMSKEGCVKTIETLKQNCDKITDEFERTKYKHELN

ENRTTSEKLLLGWLSEDLQGCRCGNCLNVIKQTVESITENSDRLEYLASSAILKSHCPECHPKGVSVSNS

TNIMNRLPGLEKTQHSDNKGFEDTNDALTDLDRVVRLTLPGKTEKERRIKRNVETLIRLMMQASGLECIK

LPSGQIITHRLTKKIRQEDENVLLDKQTERLERIKKELSDTKLSSYSEYAKKTLRHSIQRVDKQKESKCS

VPRAWLEKLLRDLKVPTKDEDILTNIRESMSKRTNFIKNNDKLVIRSEEELIKFIGNRSVQLMPDRSKKL

FQSDCILFKEVTAEAMKRYYSTPYEGVPEMIVLLINFLCRFPWFQEVVLYAKICETFLRCCTEFSRSGIK

LVKVRHCDLNLAIKLPSNKKENMLCTLYNKEMELLKGPFFLNRRQAVLGASYPYITITLYMQVLQQHRCL

EVLSTVGERTFENIKTCTSDLIASLSLELTFAVNGQFEKAYEVRTKQCKLGGNFLNRSSRDHFITVISGL

NTVYGLIIRDNLLANSQQQNKQLQMLRFGMLSGLSRLSCPKELGKKFSTSCRRLEDNIMRLYLQSTVYCS

NRDVEHNINEWKSKDLCPEVTIPCFSVYGVFVNSDRQLIFDIYNVHIYNKEMDNFDEGCISVLEETAERH

MLWEMDLLRSLSKETKDERSARLLLGCPNVRKAVDKDGNKLSKAGHASPDDGDSDSSSLSGRRSYCSSRG

RIQSIFGRYNSNKKPFEFRPGLEVRADPMNDFEQAVTDTSQYAEYTPNQESLMKDFIQIIRMNPSHTMGS

FELIQAVTEFSRNKYPAENIEKAKRDPKNWVSISEVTETTSIVSQPRTHIMLKDCFKILLGTENKKIVKM

LRGKLKKLGAINTDIEIGRKDCLDMLNTVEGLSEEQRRNIVNGIFEPSKLSFYHWKELVQKDVEEVLLTD

DGNYIFCWLKTLSSMLKGALKKELRFMNNGGVLELSNGFFSDDDFEELLNVKKELTGSMSLDRELNTELL

LSSWMKCIYKPKEGASIVQEGLEALKVMAGELYEIRLQHLELTRMKKDNPSVSFTKEEVLVKRLEKSFLK

KFNKEAMKFVNLVFFCSLSAPWCVHYKSLESYLVRHPEILELKAKGDLGSVILDLSVASAISRLVQQETN

IELDAEVLNEMKVRFAVKYFVTLFTANGEPFSLSLNDGGLDENLQKTTDEKLLHQTKVVFTKIGLAGNNY

DFMWTTQMIANSNFNVCKRLTGRTTGERLPRSVRSKVIYEMVKLVGETGMAILQQLAFAQALNYDHRFYA

VLAPKAQLGGSRDLLVQETGTKVIHATTEMFSRNLLKTTQDDGLTNPHLKETILNVGLDALSTMRLLDGR

PVSEDSKLLNFYKVICISGDNTKWGPIHCCSFFSGMIQQLLKDVPDWSSFYKLTFIKNLCRQVEIPAASV

KKILNTLRFRLSNKGGVESRSEEELRKELSDSTEEWGDNDTVKFLITTYLSKGIMALNSYNHMGQGIHHA

TSSLLTSLMAELFEELVIDYFKRHLPQLTVNVTHAGSSDDYAKCIVATGLLPKELYDRYSEAFWKHACKI

KNLTAAVQRCCQMKDSAKTLVGDCFLEFYSEFMMGNRVTPAVIKFIFTGLINSSVTSPQSLVQACHVSSQ

QAMYNSVPLMTNATFTLLRQQVFFSHVEDFVRRYGLITLGTVSSFGRLFVPTFSGLVSSAVALEDSEVLA

KSAAEINENSIFLDSSSLSNLDSLKDIVSSKARDDGSSISETTIESTESGSSSSSFTFELTRPLSETELQ

FLKTLKSTTSVTACEIIQDKLTPLYADSQEGALDKYNVLYNSRLTASCDWLRQGRKMGPLELARRLQCIL

NILIVGYYRSFGSDGTDKQVKASLNRDDNRVIEDPMIQLVPEKLRRELERLGVSRMEIDELMPSIKPDET

LCQLVAKKLISLNVATEEYSAEVSRLKQTLTARNVLHGLAGGIKELSLPIYTIFLKSYFFKDNVFLDLED

RWSTKHSSNYRDSSGRMLTGRVITKFTHWLDTFLNCTVSINRTQEIKDSSLFNPDLRCINILVREDNIKE

MSIVQSHLRVVTSEFNNLNLQFSDCNRQKLKVVESRPPECELEANKAVIVKSKLFSAVEQVRLANNPAVV

MGYLLEESSISEVKPTKVDFSNLLKDRFKLMQFFPSVFALLKHLQSESSEMEKLGAPVDMQQVSKYSNHL

TLLCRMIQQARPSLTVFYMLKGNQMNTEPTVSELVSYGIKEGRYLRLPEIGLDASTYSVKYWKILHCISA

IGELPLSDRDKTSLLISFLNWKVSSDSMAQDCPLYKQEHAVISEFAGQVVVNTLASELSSVRRDAERDSL

TDLIDYVNSPTELLKKKPYLGTTCKFATWGENNRNGKFTYSSRSGEAIGIFIAGKLHIHLSRESTGLLCE

VERNVLGWLGRRRTDILTKEQHQQFLEFLPTLSEVSQKNRDGTTQGICQDNTNVRMLKFVHPKKNTPVVK

IKGQILTVKKQVSFEAESEPRLMWGHGCVSVVYDECETQTTYHENLLKIKQMVDSTTDRARSLPQSVFSD

TKVILARIKFKSDLLLNSLCLLHSFLRHTTTDAVLEAESKCALLERYLQSGGVRVKSADETLEKKLCSKV

IECKLEQTLDEEIGVCDNLNRVFSETPMPVSSWSEVQCYIEDVGFSNILITLDKTSTKGELIWKFSLDNT

SNIAGSIRDIRSLVSYISTETIPKFLLPFLLFENLLSSILKQSLTVKETLHSTGISDKEIEAVATLFAYC

FQNDKVKRKGPRCSMASILNLTKGDWVEVGQRMKLQAHLDSDTVRLSVQLAISTDAEQTADKKARVSMAK

KVIASHLTLLLQEDGIDIKKLKDIAVNVQVRKEKTGEVLDFTLLDDQAGNLNYIGVLETIMDRRKKGPAI

SALEDFFLLLTGMAESNIQQDTAVKSEEACSDDICLEDLLEPSEESSSSSGALDKETVPDKVTFNWDSDS

D

>Uukuniemi_Virus_(from_unknown)_(Phenuiviridae)_(NP941973)

MLLAICSRTIRQQGLNCPPAVTFTSSHMRPPIPSFLLWTEGSDV

LMDFDLDTIPAGSVTGSSIGPKFKIKTQAASSFVHDFTFAHWCDASDMPLRDHFPLVN

DTFDHWTPDFISQRLDGSKVVVEFTTNRSDQEQSLISAFNTKVGKYEVALHNRSTTSS

ILFGVVVVSETTVVTNLNLNQQEVDELCFRFLVARAVHLEMTTKMIIPEYDDEDEDKR

SREVKAAFHSVQPDWNVTEANFAPFSRRMFSNFAQMEPDKEYLAHIILDSLKQAQADL

DGNHYLNESLTEQARLDRNREESLNMVKDFERDFNNAAQRSAWSHKSTVPFPGVIPKV

SGDTTSLSRLVELPVITGGSDATIRAWRSAYGSVSNGTVERCDEDVERERRAALCSLT

VEELEESKALRMKYHRCKIDNGMMDKLDLAMQGVEAKEFKNHPSIIKKRSKSKKTFPL

TADTRDIDLFLHHDDLMFNNEHSQTPPAAMIEAVKAGADAQSLHGLDKSANPWYASAL

WFLGLPIGLWLFMCTCIGVELSISLKQHCGRQKFIIKKLRFFDIFLLIKPTNSGSHVF

YSIAFPESAILGKLHRSQCFKGLQFEDGWFWTEFSSFKMSKLTNVVKCLSTGFNLFWF

WRDYYEVPFWAGNEKDFQTGKQRANKMFKFCLLMLLEDKARTEEIATLSRYVMMEGFV

SPPCIPKPQKMIEKLPNLARTKFQVWLISRMLQTIIRVSDYPFKITAGHKSANWTGMF

NWVTGEPIESTQKLISLFYLGYLKNKEESPERNASIGMYKKILEYEDKHPGRYTYLGL

GDPPSDDTRFHEYSISLLKHLCIHAEHDLRRNWGESFKAMISRDIVDAIASLDLERLA

TLKASSNFNEEWYQKRGDGKTYHRSKVLEKVSKYVKKSSSHVHHIMEECLRKVESQGC

MHVCLFKKPQHGGLREIYVLGFEERVVQLVIETIARQICKRFKSETLTNPKQKLAIPE

THGLRAVKTCGIHHETVATSDDAAKWNQCHHVTKFALMLCHFTDPLFHGFIIRGCSMF

MKKRIMIDQSLIDIIDSHTTLETSDAYLQKIHRGYHGSLDDQPRWISRGGAFVQTETG

MMQGILHYTSSLLHTLLQEWLRTFSQRFIRTRVSVDQRPDVLVDVLQSSDDSGMMISF

PSTDKGATGKYRYLSALIFKYKKVIGKYLGIYSSVKSTNNTLHLLEFNSEFFFHINHN

RPLLRWITACDTISEQESLASRQEEMYNNLTSVLEGGGSFSLVSFCQFGQLLLHYTLL

GMTVSPLFLEYIKLVSEIKDPSLGYFLMDHPFGSGLSGFKYNVWVAVQNSILGSRYRS

LLEAIQNSDSAAPKKTLDTTTSGTFVQSTIIRFGDRKKWQRLVDRLNLPEDWLDVIDK

NPEIVYRRPRDGFEVSLRIAEKVHSPGVSNSLSKGNCIIRVISSSVYILSRSILSDGL

AWLYDEEEEVKRPLLYKVMNQPELDLHSRLTPAQLSTLFPMMAEFEKLQTHLRSYMKI

EGEFISKKKVITQTRVNILETERFLRARPEDLIADKWFGFTRTRMTPRTFKEEWENLT

SVFPWLTGNPSETLELSPFQHHVQLRNFFSRLDLKGRDIRIIGAPIKKSSGVSNVSTA

IRDNFFPRFVLTHIPDEAAMERIEAAGILKHALFLTVTGPYTDQSKLDMCRDFITSSE

PITLKPNHGKTRTNVLSLFQDYFSKRGPDIIFNRIQMANCGVIGGFTSPQKPKEVDGK

IVYTGDGVWRGIVDGFQIQLVITYMPKQKSNELKSITVNSDRCISALSSFCQSWCKEM

GVFNTEDFSKTQRFSKASFFMHKFKISGSKQTLGAPIFIVSEKIFRPICWDPSKLEFR

VRGNTLNLTYKEVNPGAGQRMFNILSYTVKDTDVSDENAFKLMSLSPRHKFHGREPST

SWICMRALPISTIDKLLERILNRERISGSIDNERLAECFKNVMESTLRRKGVFLSEFS

RATQKMLDGLSRDMLDFFAEAGLNDDLLLEEEPWLSGLDTFMLDDEAYLEEYNLGPFG

VFSVEQEMNTKYYHHLLLDSLVEDVIQKLSLDGLRKLFQEEEAPLEYKKEVIRLLNIL

QRDASQIKWKSRDLLSENMGLDVDDDMFG

>Bunyamwera_virus_(from_unknown)_(Peribunyaviridae)_(NP047211)

MEDQAYDQYLHRIQAARTATVAKDISADILEARHDYFGRELCNS

LGIEYKNNVLLDEIILDVVPGVNLLNYNIPNVTPDNYIWDGHFLIILDYKVSVGNDSS

EITYKKYTSLILPVMSELGIDTEIAIIRANPVTYQISIIGEEFKQRFPNIPIQLDFGR

FFELRKMLLDKFADDEEFLMMIAHGDFTLTAPWCTSDTPELEEHEIFQEFINSMPPRF

VSLFKEAVNFSAYSSERWNTFLYRARAETEVDYNQFLSDKAHKIFMLEGDYMRPTQAE

IDKGWELMSQRVYTEREIITDVTKQKPSIHFIWVKNADRKLIGSTAKLIYLSNSLQSI

TEQSTWTDALKAIGKSMDIDGKVGQYETLCAERKMIARSTGKKVDNKRLEAVKIGNAL

VLWEQQFILANDLFKNQERQKFMKNFFGIGKHKSFKDKTSSDIETDKPKILDFNNTIV

LMAARTMVNKNKALLAKDNTLQDLHPIIMQYASEIKEASKDTFDALLKISKTCFWQCI

VDVSTIMRNILAVSQYNRHNTFRVAMCANDSVYALVFPSSDIKTKRATVVFSIVCMHK

EKNDLMDAGALFTTLECKNKEYISISKAIRLDKERCQRIVSSPGLFILSSMLLYNNNP

EVNLVDVLNFTFYTSLSITKSMLSLTEPSRYMIMNSLAISSHVRDYIAEKFSPYTKTL

FSVYMVNLIKRGCASANEQSSKIQLRNIYLSDYDITQKGVNDGRNLDSIWFPGKVNLK

EYINQIYLPFYFNAKGLHEKHHVMIDLAKTVLEIEMNQRSDNLGIWSKAEKKQHVNLP

ILIHSIAKSLILDTSRHNHLRNRVESRNNFRRSITTISTFTSSKSCIKIGDFREIKDK

ETEKSKKSTEKFDKKFRLSNPLFLEDEEANLEVQHCNYRALIQKIPNYKDYISVKVFD

RLYELLKNGVLTDKPFIELAMEMMKNHKEFSFTFFNKGQKTAKDREIFVGEFEAKMCM

YVVERISKERCKLNTDEMISEPGDSKLKILEKKAEEEIRYIVERTKDSIIKGDPSKAL

KLEINADMSKWSAQDVFYKYFWLIAMDPILYPAEKTRILYFMCNYMQKLLILPDDLIA

NILDQKRPYNDDLILEMTNGLNYNYVQIKRNWLQGNFNYISSYVHSCAMLVYKDILKE

CMKLLDGDCLINSMVHSDDNQTSLAIIQNKVSDQIVIQYAANTFESVCLTFGCQANMK

KTYITHTCKEFVSLFNLHGEPLSVFGRFLLPSVGDCAYIGPYEDLASRLSAAQQSLKH

GCPPSLVWLAISCSHWITFFTYNMLDDQINAPQQHLPFNNRKEIPVELNGYLNAPLYL

IALVGLEAGNLWFLINILKKLVPLDKQKETIQSQCLHLCNSIDKLTESEKFKLKILRY

LTLDTEMSVDNNMGETSDMRSRSLLTPRKFTTLGSLNKLVSYNDFRSSLDDQRFTDNL

NFMLNNPELLVTKGENKEQFMQSVLFRYNSKRFKESLSIQNPAQLFIEQILFSHKPII

DYSSIFDKLTSLAEADIIEELPEIIGRVTFPQAYQMINRDIGQLPLDIDDIKLIFRYC

ILNDPLMITAANTSLLCVKGTPQDRTGLSASQMPEFRNMKLIHHSPALVLKAFSKGTS

DIPGADPIELEKDLHHLNEFVETTAIKEKILHNIDNPPKHLIGNEILIYRIREMTKLY

QVCYDYVKSTEHKVKIFILPMKSYTAIDFCTLIQGNTISDNKWYTMHYLKQIASGSIK

GNIVTTSTSEQIIANECFRVLCHFADSFVEEASRLSFINEVLDNFTYKNISVNSLFNT

LLASTTRLDFIPLLFRLKVLTQTDLNRFDALKTNERVSWNNWQTNRSLNSGLIDLTIS

GYLRSIRVVGEDNKLKIAELTIPNFYPNTVFHAGNKLLNSRHGLKFEYMEEIVLDEKY

NYYITYQKKRAHIYTYQVSTIEHILRRNNEGLQSRGPRYNKMVPVCPVVLSVRDELFR

MSLENVFSLNMTNFSMSRLFVSPDEVATVKKAHMSKMMFFSGPTIKAGIINLTSLMRT

QELLTLNYDNLCKSSIVPFCRILECNGDEQGELIFLSDEVMDFTISEEIESMPLFTIR

YQKRGTEIMTYKNAIMKLVSAGVDEIKEVFDFSKQGFYSKKNLGIINTICSIINILET

NEWSTILYNSFHIAMLLESMDREFHMFTLPEAFFINVAGGVVNWTKLLKFIKSLPVIE

QEPWSMMMSRFVEKTVYLIEREMNKDVDFTDFLDELEFSSGKSLFTFF

>Herbert_herbevirus_(from_Culex_nebulosus)_(Peribunyaviridae)_(YP009507855)

MENYEIIKKKFNERSNNGFQNAEIYNSLIKCRHDIFGEQICSAL

DIPIRNDVDFEVIIEDLLNKYDFRLEKYFKVTPDNYKIEDNILLIIDYKVSRSTMNIE

KTLIKYNNAFNWVPKLLPIDFKTIVINLNPDTLMIWSNQMEFLDQYRFEINLEEIKSI

NDMLEVLEIQNSDDDIFMRNRIGDSIQVTEDWFDVDDSVVDQTLKNDVTFFDFESSLD

KDILGIYTKSLNSNVGMSGIEMQEIYKETIKKTQENANKTKKKMLGNLSDLEKLIPNK

DNIDSGWRILESKLLETKSLSNNLSDSKPTVHLLVSTIDIVDPDLEMNNFKKCCLFGR

LIKLLDWSKVIGYKNNLIRNDHFQEFFKILGEDMDMAGYETSYNSMVQEFKSNRTGFS

NNTKYQKKSQSKSVSPNLPGIMVNDMQFLLRNFPNRKLFTSFCGIDISKSNDFKKGLV

PEQTKPFSLSPFDESLKEQVNEIFNNIKLAQISPTQEFSKTVLKDYINKTRDIDQDNY

MLYENIIKTKGYKICTDMSILIKNALSVANFQNYKTYRLVFSANKSSFLILLPSMSIK

YAQSSICFISCCFVKKGDSIEYYAGCDRYRIPVTGTEFEMLISNPIRLNKERAKRLVE

APFLMFLISSSFVTTGCDLDSIINYSFYSSLNITKSLMTLTEPARYILMGSSAKVSDV

KSYIGEKFEPSLKTAFSIWIYNKIKDASYVANEKLKGIVCKDVFFDEDKVKGRGVKSI

KNLPSIYFKGDLDLKGYLNEVFIPFYINSKGLHEFHHNIIDLTKVPCQIEIETKKDMK

EFWKESDSQYCNLPVFLNYSAKYYQRLVKQGARYRNNVETSNMFKEPIYKIPTMTSSK

SCVQVGDFFDVKIHSTDKKVQLALKTKMPNTRSYITIDYDPEILDDMVLYIPIDKVFS

SKLFSSKMVIMDTIKTSKQFKTWIQGSNIGDTFRLSGKNGYYIFCICWDNLKDKFEWD

SPSVGFFVNAMKLYNDSSKMQGITNIFPSGFTKNVNQLEYMIETGIMKANLKVYNNSY

YLFEQGLRDYSHTVRENMEITPKDSDNSNLLKTGLYQHHDYKAIRSNIKNYTDYITKR

VFDRLYDKIGEFQNKEFIKIAGECILSHETYYSTLFCKDQRTAKDREIYEMELEGKIL

LYVIERLFKTYSREDMNEMISRPGDVKVLDIENSRNRLFSFATQFQSNERYKYNVYMN

EINADMSKWSAKDITAKYLFLIALNPSLRSKEKKVISLGLCRYMRKRLILPDSAMGIL

LDQYSKRIDDPIIEMTNNLSTNCVTISQNWFQGNLNYMSSFCHSIAMDFYKDLNFDFA

KTMGIDNVLTVSLVHSDDNQTGVCMIEKIDTLLETEKTTNIYNQSQDKKIATAIFKLL

ELSMRQFGFILNTKKTYISSIIKEFISMHNLNGEPFSVYHRFLFPVVGACSFLGPYED

LTARLSGIQTAIRHGCPPSLAICSIGCATEMTYGTYSMLPGMKNDPGHIFGYDRFLLP

LEIGGYPFFDLSNFVELGTVANDIRLLRPMIAKLVNTIKYKTIEEQLLNFNSSDIKLL

NEYEKFIIKFYKEFCITDLFDPSGSIGETYEMSKRSLLTPRKFTTLKLIKKLTSYKHF

TSMSQAEKLQNYEFMLSNKELLVSKPLDFEAFKQVILFRYNSRKFKESLSIQSPVQLF

LEQIISSGSKCIDREFLNDIDNEPNIDLAEPNRNQLLGRLTFVEAFQRIRSIIQRKEI

SFDDMMVVYKSKITNDPLTSTCYNIEYAVEELSHSAKESVYSQKLQEYRQVRSFLSTP

HSIIMSLLKYIESGIEPSEDTVLYSDMMELLKYFIESGLQAYLRDYNYKHMNDQNLYV

KEQLSIWTEVLQILYLYTTQINKRNMSILLPKRTYSLTEFLITLKGALQRDKFLVNYK

LTFNPLKFKAKGITKLEQLDTINTAESFFRSMTTFMESYIKPEFRANLILQIIKNCSL

GSTTISELYDLVRINCPHQFMALLNLLGDLEETTVKSYVSNIKSVSQAWLKEQSFRQG

MIGEFDVIYYNLRSSLQVKGDNKKFTDMIFTYQRGLDLNQLIYKDLDIMLNKLRYDMK

LDQIEFDPPISMDDNSIYFVKAWNKGIAKYTAKYYKDILQTDLMLCPINIHISNETNY

EINDIMNSNINNMSCRYFKINDALKSYQTSRRGPISVLLNLKGDIDALGTFSLSSYIR

FIASFQGTQKPSFASLLNFIDLFKCRDTNKTLVEAPKEIRTEIFCFDDANLDIEFNFD

VIFKMKVKTPYSSLTTLMDIVNNKEKLVEYMMTKRDDKKTVRDYQKSLAILIRQFQVI

QNMKMKLGVSLEADSIFVKMLHLYSLDTPAHKGFHDINSLPEKFFKKDKLFELDLEFV

EKNVMIKLDLPNNFLEKKLEIEILEVFKKLKTEYRNFVSKEFPDLSWADEVEKSGFSF

F

>Herbert_herbevirus_(from_Culex_quinquefasciatus)_(AGX32061)

MENYEIIKKKFNERSNNGFQNAEIYNSLIKCRHDIFGEQICSALDIPIRNDVDFEVIIEDLLNKYDFRLEKYFKVTPDNY

KIEDNILLIIDYKVSRSTMNIEKTLIKYNNAFNWVPKLLPIDFKTIVINLNPDTLMIWSNQMEFLDQYRFEINLEEIKSI

NDMLEVLEIQNSDDDIFMRNRIGDSIQVTEDWFDVDDSVVDQTLKNDVTFFDFESSLSKDILGIYTKALNSNVAMSGIEM

QEIYKETIKKTQENANRTKKKMLGNLSDLEKLIPNKDNIDSGWRILESKLLETKSLSNNLSDSKPTVHLLVSTIDIVDSD

LEMNNFKKCCLFGRLIKLLDWSKVIGYKNNLIRNDHFQEFFKILGEDMDMAGYETSYNSMVQEFKSNRTGFSNNTKYQKK

SQSKSVSPNLPGIMVNDMQFLLRNFPNRKLFTSFCGIDISKSNDFKKGLVPEQTKPFSLSPFDESLKEQVNEIFNNIKLA

QISPTQEFSKTVLKDYINKTRDIDQDNYTLYENIIKTKGYKICTDMSILIKNALSVANFQNYKTYRLVFSANKSSFLILL

PSMSIKYAQSSICFISCCFVKKGDSIEYYAGCDRYRIPVTGTEFEMLISNPIRLNKERAKRLVEAPFLMFLISSSFVTTG

CDLDSIINYSFYSSLNITKSLMTLTEPARYILMGSSAKVSDVKSYIGEKFEPSLKTAFSIWIYNKIKDASYVANEKLKGI

VCKDVFFDEDKVKGRGVKSIKNLPSIYFKGDLDLKGYLNEVFIPFYINSKGLHEFHHNIIDLTKVPCQIEIETKKDMREF

WKESDSQYCNLPVFLNYSAKYYQRLVKQGARYRNNVETSNMFKEPIYKIPTMTSSKSCVQVGDFFDVKIHSTDKKVQLAL

KTKMPNTRSYITVDYDPEILDDMVLYIPIDKVFSSKLFSSKMVIMDTIKMNKQFKAWIQGSNIGDTFRLSGKNGYYIFCI

CWDNLKDKFEWDSHSVGFLVNAMKLYNDSSKMQGVTNIFPSGFTKNVNQLEYMIETGIMKANLKVYNNSYYLFEQGLRDY

SHTVRENMEITPKDSDNSNLLKTGLYQHHDYKAIRSNIKNYTDYITKRVFDRLYDKIGEFQNKEFIKIAGECILSHKTYY

STLFCKDQRTAKDREIYEMELEGKILLYVIERLFKTYSREDMNEMISRPGDVKVLDIENSRNRLFSFATQFQSNERYKYN

VYMNEINADMSKWSAKDITAKYLFLIALNPSLRSREKKVISLGLCRYMRKKLILPDSAMGILLDQYSKRIDDPIIEMTNN

LSTNCVTISQNWFQGNLNYMSSFCHSIAMDFYKDLNFDFAKTIGIDNVLTVSLVHSDDNQTGVCMIEKIDSLLEPEKTTN

IYNQSQDKKIATAIFKLLELSMRQFGFILNTKKTYISSIIKEFISMHNLNGEPFSVYHRFLFPVVGACSFLGPYEDLTAR

LSGIQTAIRHGCPPSLAICSIGCATEMTYGTYNMLPGMKNDPGHVFGYDRFLLPLEIGGYPFFDLSNFVELGTVANDIRL

LRPMIAKLVNTIKYKTIEEQLLNFNSGDIKLLNEYEKFIIKFYKEFCITDLFDPSGSIGETYEMSKRSLLTPRKFTTLKL

IKKLTSYKHFTSMSQAEKLQNYEFMLSNKELLVSKPLDFEAFKQVILFRYNSRKFKESLSIQSPVQLFLEQIISSGSKCI

DREFLNDIDNEPNIDLAEPDRNQLLGRLTFVEAFQRIRSIIQRKEINFDDMIVVYKSKITNDPLTSTCYNIEYAVEELSH

SAKESVYSQKLQEYRQVRSFLSTPHSIIMSLLKYIESGIEPSEDTVLYSDMMELLKYFIESGLQAYLRDYNYKHMNDQNL

YVKEQLSIWTEVLQILYLYTTQINKRNMSILLPKRTYSLTEFLITLKGALQRDKFLVNYKLTFNPLKFKAKGITKLEQLD

TINTAESFFRSMTTFMESYIKPEFRANLILQIIKNCSLGATTISELYDLVRINCPHQFMALLNLLGDLEETTVKSYVSNI

KSVSQAWLKEQSFRQGMIGEFDVIYYNLRSSLQVKGDNKKFTDMIFTYQRGLDLNQLIYKDLDIMLNKLRYDMKLDQIEF

DPPISMDDNSIYFVKAWNKGIAKYTAKYYKDILQTDLMLCPINIHISNETNYEINDIMNSNINNMSCRYFKINDALKSYQ

TSRRGPISVLLNLKGDIDALGTFSLSSYIRFIASFQGTQKPSFASLLNFIDLFKCRDTDKTLVEAPKEIRTEIFCFDDAN

LDIEFNFDVIFKMKVKTPYSSLTTLMDIVNNKEKLVEYMMTKRDDKKTVRDYQKSLAILIRQFQVIQNMKMKLGVSLEAD

SIFVKMLHLYSLDTPAHKGFHDINSLPEKFFKKDKLFELDLEFVEKNVMIKLDLPNNFLEKKLEIEILEVFKKLKTEYRN

FVSKEFPDLSWADEVEKSGFSFF

>Herbert_herbevirus_(from_Culex_sp.)_(AGX32060)

MENYEIIKKKFNERSNNGFQNAEIYNSLIKCRHDIFGEQICSALDIPIRNDVDFEVIIEDLLNKYDFRLEKYFKVTPDNY

KIEENILLIIDYKVSRSTMNIEKTLIKYNNAFNWVPKLLPIDFKTIVINLNPDTLMIWSNQMEFLDQYRFEINLEEIKSI

NDMLEVLEIQNSDDDIFMRNRIGDSIQVTEDWFDVDDSVVDQTLKNDVTFFDFESSLDKDILGIYTKALNSNVGMSGIEM

QEIYKETIKKTQENANKTKKKMLGNLSDLEKLIPSKDNIDSGWKMLESKLLETKSLSNNLSDSKPTVHLLVSTIDIVDSD

LEMNNFKKCCLFGRLIKLLDWSKIIGYKNNLIRNDHFQEFFKILGEDMDMAGYEASYNSMVQEFKSNRTGFSNNTKYQKK

SQSKSISPNLPGIMVNDMQFLLRNFPNRKLFTSFCGIDISKSNDFKKGLVPEQTKPFSLSPFDESLKEQVNEIFNNIKLA

QISPTQEFSKTVLKDYINKTRDIDQDNYMLYENIIKTKGYKICTDMSILIKNALSVANFQNYKTYRLVFSANKSSFLILL

PSMSIKYAQSSICFISCCFVKKGDSIEYYAGCDRYRIPVTGTEFEXLISNPIRLNKERAKRLVEAPFLMFLISSSFVTTG

CDLDSIINYSFYSSLNITKSLMTLTEPARYILMGSSAKVSDVKSYIGEKFEPSLKTAFSIWIYNKIKDASYVANEKLKGI

VCKDVFFDEDKVKGRGVKSIKNLPSIYFRGDLDLKGYLNEVFIPFYINSKGLHEFHHNIIDLTKVPCQIEIETKKDMREF

WKESDSQYCNLPVFLNYSAKYYQRLVKQGARYRNNVETSNMFKEPIYKIPTMTSSKSCVQVGDFFDVKIHSTDKKVQLAL

KTKMPNTRSYITIDYDPEILDDMVLYIPIDKVFSSKLFSSKMVIMDTIKMNKQFKTWIQGSNIGDTFRLSGKNGFYIFCI

CWDNLRDKFEWDSHSVGFLINAMKLYNDSNKMQGVTNIFPSGFTKNVNQLEYMIEAGIMKANLKIYNNSYYLFEQGLRDY

SHTVRENMEITPKDSDNSNLLKTGLYQHHDYKAIRSNIKNYTDYITKRVFDRLYDKIGDFQNKEFIKIAGECILSHETYY

STLFCKDQRTAKDREIYEMELEGKILLYVIERLFKTYSREDMNEMISRPGDVKVLDIENSRNRLFSFATQFQSNERYKYN

VYMNEINADMSKWSAKDITAKYLFLIALNPSLRSREKKVISLGLCRYMRKMLILPDSAMGILLDQYSKRIDDPIIEMTNN

LSTNCVKISQNWFQGNLNYMSSFCHSIAMDFYKDLNFDFAKTMGIDNVLTVSLVHSDDNQTGVCMIEKIDSLLETEKTTN

IYNQSQDKKIATAIFKLLELSMRQFGFILNTKKTYISSIIKEFISMHNLNGEPFSVYHRFLFPVVGACSFLGPYEDLTAR

LSGIQTAIRHGCPPSLAICSIGCATEMTYGTYSMLPGMKNDPGHIFGYDRFLLPLEIGGYPFFDLSNFVELGTVANDIRL

LRPMIAKLVNTIKYKTIEEQLLNFNDSDIKLLNEYEKFIIKFYKEFCITDLFDPSGSIGETYEMSKRSLLTPRKFTTLKL

IKKLTSYKHFTSMSQAEKLQNYEFMLSNKELLVSKPLDFESFKQVILFRYNSRKFKESLSIQSPVQLFLEQIISSGSKCI

DREFLNDIDNEPNIDLAEPDRNQLLGRLTFIEAFQRIRSIIQRKEISFDDMMVVYKSKITNDPLTSTCYNIEYAVEELSH

SAKESVYSQKLQEYRQVRSFLSTPHSIIMSLLKYIESGIEPSEDTVLYSDMMELLKYFIESGLQAYLRDYNYKHMNDQNL

YVKEQLSIWTEVLQILYLYTTQINKRNMSILLPKRAYSLTEFLITLKGALQKDKFLVNYKLTFNPLKFKAKGITKLEQLD

TINTAESFFRSMTTFMESYIKPEFRANLILQIIKNCSLGSTTISELYDLVRINCPHQFMALLNLLGDLEETTVKSYVSNI

KSVSQAWLKEQSFRQGMIGEFDVIYYNLRSSLQVKGDNKKFTDMIFTYQRGLDLNQLIYKDLDIMLNKLRYDMKLDQIEF

DPPISMDDNSIYFVKAWNKGIAKYTAKYYKDILQTDLMLCPINIHISNETNYEINDIMNSNINNMSCRYFKINDALKSYQ

TSRRGPISVLLNLKGDIDALGTFSLSSYIRFIASFQGTQKPSFASLLNFIDLFKCRDTNKTLVEAPKEIRTEIFCFDDAN

LDIEFNFDVIFKMKVKTPYSSLTTLMDIINNKEKLVEYMMTKXDDKKTVRDYQKSLTILIRQFQVIQNMKMKLGVSLEAD

SIFVKMLHLYSLDTPAHKGFHDINSLPEKFFKKDKLFELDLEFVEKNVMIKLDLPNNFLEKKLEIEILEVFKKLKTEYRN

FVSKEFPDLSWADEVEKSGFSFF

>Kibale_virus_(from_Culex_simpliciforceps)_(YP009362027)

MDSYDGLKRRFNERSTNGFQNADIYNSLIKCRHDIFGEQICASFDIPIRNDVDFEVIVDDLQNTYDFQLEKYFKVTPDNY

KIQDDLLLIIDYKVSRSTMNIEKTLVKYNNAFNWVPLVLPIDFKTIIINLNPDTLMIWSNQTEFLELYKFEIVLDQIRDI

NDMLEILEIQNSDDDIFMRNRTGDNIHVTDDWFDEENHEIEELLNNDLTFFDFTKSLDSSMLSLYNKMINSDVELTGNQM

QEVYRNVIKETQQSVKETKKSMLEKLVDLDSLVPNKTNIDVGWKMLEEKLEKNKTLSNDLSDSKPTIHTLISTIDLVDPE

LDMNNFKKCCLFGSLLKLLDWNRSTSYKNNLIRVDHFQDFFKILAEDMDMAGHETAYNSMVAEFKCNRTSFSNNTKYQKK

SQSKSVSPSLPGIMVNDMQFLLRNFPYKRNFTSFCGIDVSKSNDFKKGVSPELQKPFSLCPFDPSLKEQVNEIFEGVKLA

SVNPTQEFTKTVLKDYINKTRGIDNDNYELYETIIKTKGYKIATDISVLIKNALSVANFQNYKTYRLVFSANKSSFLILL

PSMSIKYAQSSICFISCCFVKKNTQLEYYSGCDRYRVPVIGTEFELIISNPIRLNKERAKRLVEAPYLMFLVAASFITTG

CDLDSIINYSFYSSLNITKSLMTLTEPARYILMGSSAKVSDVKSYIGDKFEPSLKTAFSVWIYNKIKDASYIANEKLKGI

VCKDVFFDEDKVKGRGVKSIKNLPSIYFKGDLDLKGYLNEIFIPFYINSKGLHEFHHNIIDLTKVPCQIELETKKDLKDF

WVESDNQYCNLPVFLNYSARYYQRLVKQGARYRNNVETSNMFREPIYKIPTMTSSKSCVQVGDFFDIKVHGTDKKVQHLL

KTKMPNTRSYHTIDYDPEVLDDMILYIPIDKAYTNKLYSSNMTIMESITMNKHFKEWLINGTLGDTFRFNVKNSCYVFSL

CWDNLKTKFEWDLSSVGHLVKAFKEYNNLDKMQGITNLFPLGFTKNINQFEYMCESLLVKSSLMVYNNSYYIFENGLRDY

THIIRENLEISPKDSDNNNLLKTGLYKHHDYKAIRSNIKNYTDYITKRVFDRLYDKIDEYQDKEFIKIAGSCITKHQVYY

STLFCKDQRTAKDREIYEMELEGKILLYVIERLFKTYSREDMNEMISRPGDVKVLDIENSRNRLFSFATQFQSNDRYKYN

VYMNEINADMSKWSAKDITAKYLFLIALNPSLKSKEKKILTLGLCRYMRKILILPDSAIGILLDQYSHRLNDPIMLMTEN

LSSNCVKITQNWFQGNLNYMSSFCHSIAMDFYKEMNQEFGRSIGINDILTVSLVHSDDNQTGVCMIEKMDLLKTEINMNE

HTLKQDTKIATCVFKLLELSMRQFGFILNTKKTYISSIIKEFISMHNLNGEPFSVFHRFLFPVIGACSFLGPYEDLTARL

SGIQTAIRHGCSPSLAICAIGCATEMTYGTYSMLPGMKNDPGPTFGYDRFLLPLEIGGYPFFDLSNFIELGTVANDIRLL

RPIISKLTNTIKHKTIEDQLLSFTESDMMLLNDYDRFIIKFFKEFCITDLFDPSGSIGETYEMSKRSLLTPRKFTTLKLI

KKLTSYKEFSKLTPISKLENFQFMLEHKELLVSKPLNSEAFKNVILFRYNSRKFKESLSIQSPVQLFLEQVISSGSKCID

REFLNDIDNEPDIDISEPNRNQLLGRLTFLEAFSKIKKITLKTQIGLNELLIVYKSKITNDPLTSTCYNMEFAVEELSTT

PKESLYSQKLQEYRQVRSFLSTPHNIITSLLTLLETGVEPNEDTVLYSDVMELYKYYVESGLKEYMDNYNYKHKSDSTLY

TKEQLSVWTEVLQILYLYTTQINKRNMSILLPKRAFSLTEFLLTLKGTLQQDKQFINYKLLFNPLKFKAKGITKLEQFDT

VNTAEALFRSLTTFMENYIKPEFRANLILQIINNCSIGDVPISDYYELVKLNCPHQFMALLNLLGDLQETTVKSYISNIK

SVSQSWIQEQSFRQGMIGEFDVIYYNLRSSLEVKGNNKTFTDMIFTYQKSLDLNQLIYKDLDIMLNKLKYDMKLDHIDFN

PPLSQDDNSIYFVKAWNKGIAKYTAKYYKDILQSDLMLCPISIHASGQTNYEIDDILGSNLDNMSCRYFKINESLKSYQT

SRRGPISTLLNLKGDIDALGTFSLSSYIRFISGFQGVQKPSFSSILNFIDLFSCRDTSKTFIEAPEEIKTEIFCFDDTNL

DIEFNFDVIFKMKVKTPYSSLNALMDVANNKDKLMEYMITRTRDVKTVKEYQRSLSILIRQFQVVQNMKLKLGVKMDEDS

IFVKLLHLYSLDTGSHKGFHDSNDLPGVFYKKGKVLELDIDFIEKNVMTVLELPNSFLEKRSNSEVLDVFHKLKNEYRNL

VSREFPDLSWADEVERSGFSFF

>Tai_virus_(from_Culicidae_sp.)_(YP009362026)

MELYTVLRKRFNERGANAFQNADLYNSLIKCRHDIFGEQICASFDIPLRNDVDFEVIVDDLSNNYEFKLEKFFKVTPDNY

KIEEGMLLIIDYKVSRSTLNIEKTLVKYNNAFNWVPKLLPIDFKTIIINLNPDTLAVWSNQMEFLDNYKFELNLDEIRDI

NDMLEVLEIQNSDDDTFMRNRVGDNIAVTDDWFDIEEANIEKVLENDVTFYDFKRSLDSDTRKLFEKMLKSNVSIMGTEM

QELYKDVIKTTQQSVNKTKKSLISKLTDLEKLVPSKENIDIGWKDLETKLTSTKDLSNNILDAKPTVHMMISTIDLVDKE

LDMNNFKKCCLFGGLIKLMDWSKCIPYKNGLIRVDHYQDFFKVLGDDMDMVGYESEYNSMVQEFKSNRTGFSNNSKYQKK

SQSRSISPNLPGIMVNDMQFMLRNFTNKKEFTKFCGIDVSKNNDFKKGITPEQTKPFCLSPVDESLKEQVNELFNAAKLS

EVNPTQEFQKTVLKDYIVRTRSMDPGNYELYDSIIKTKGYKIATDISILIKNALSVANFQNFKTYRLIFSANKSSFLILL

PSMSIKYAQSSICFISCCFTKKNEDIDYYSGCNRYRVPLTGTDFDILISNPIRLNKERAKRLVEAPFLMFLVASSFITTD

CDVESIINYSFYSSLNITKSLMTLTEPARYILMGSSAKVSDVKSYIGEKFEPSLKTAFSIWIYNKIKEASYIANEKLKGI

VCKDVFFDEDKVKGRGVKSIKNLPSIYFKGNLDLKGYLNEVFIPFYINSKGLHEFHHNIIDLTKVPCQIEIETKKDMKEF

WIESENQYCNLPVFLNQSAKYYQRLVKQGARYRNNVETSNMFNEPIYKIPTMTSSKSCVQIGDFFDIKVHGTDKKVHHAL

KTKMPNTRSYYTVDFDPEILEDTVLYIPIDKNFSSKLFSSKMTIISTITMNKEFKVWQSKSHIGDCFKQVSKNSTYVFCI

CWDTLKDRYEWDAVSANYLVNCIKLNNNQDKLQGITNLFPLGFTKDINAIEYLIESKLIKSNLKIYNNSIYIFEQGLRDY

THHVRENLEITPKDSDNNNLLKTGLYKHHDYKAIRNNVKNYQDYITKRVFDRLYDKVDEFKDKEFIKIAGDCILKHQTYY

STLFCKDQRTAKDREIYEMELEGKILLYVIERLFKTYSREDMNEMISRPGDVKVLDIENSRNRLFTFATQFQSNARYKYN

VYLNEINADMSKWSAKDLTAKYLFLIALNPSLKSKEKKLLILGLCRYMRKVLLLPDSAIGILLDQYSFREDDPIMKMTNN

LSTNCVKISQNWFQGNLNYLSSFCHSIAMDFYKDLNFEFAKTLNIENTLTVSLVHSDDNQTGVCMIEKIDSVTEPDKMTQ

EYSQNQDQKIASAIFRLLEFAMRQFGFILNTKKSFISSIIKEFISMHNLNGEPFSVFHRFLFPVIGACSFLGPYEDLTAR

LSGIQTAIRHGCPPSLATCAIGCATEMTYATYNMLPGMKNDPGPVYGYDRFQLPLEIGGYPAFDLSNFVELGTVANDIRL

LRPIISKVVNTIKFKTIEEQLLNFDESYIKLLNTYEKFIVKFYKEFCITDLFDPSGSIGETYEMSKRSLLTPRKFTTMKL

IKKLTSYKDFVNLSGEAKLNNFQFMLQNKELLVSKPLDSASFKQVVLFRYNSRKFKESLSIQSPVQLFLEQIISSGSKCI

DREFLNDIDTEPLVDISEPNRNQLLGRLTFVEAFQKIKLIVQNKDLTLEDLLVVYKSKITNDPLTSTCHNIEFAVEELSS

VGKESYYSQKLQEYRQVRSFLSTPHNIITALLRLIETGVEPGEDTVLYSDVTELYKYFVESGLQSYLADYNFRHKNDPTL

YVKEQLSIWTEVLQILYLYTTQINKRNMSILLPKRAYSLTDFLLTLKGSLQKDKLLINYKLLFSPLKFKAKGITKLEQLD

TINTAESFFRSLTTFMENYVKAEYRANLILQIIKNCSIGSNTVSELYDLVRLNCPHQFMALLNLLGDLEEQTVKSYISNI

KSVSNAWIKEQSFRQGMIGEFDVIYYNLRSSLEVKGNNKTFTDMIFYYQSGLDLNQLIAKDLDIMLNKLRYDMKLDQIEF

NAPLSEDDNSLYFVKTWIRGIAKYSAKYLKDITSTDLMLCPISLHITKQSNYEFNEILDANLDNKSCRYFKINESLKSYQ

TSRRGPISVLLNMKGDIDALGTFSLSSYIRFIAGYQGMQKPNFASLLNFIDLFSCRDTTQTLIETPSEIKTEIFCFDDTD

LDIEFNFDVIFKMKVRTPYSSLNALMDIINNKDKFLQYMTTNEKPKTSQEYHKNINILTRQFQVIQHMKSRLGFQLEEDS

IFIKLLHLYSLGTPTHLGFHDVNKLPEKFMKKDKFLELDLDFIEKNVMVDISLPNLFLERKHQTEIKSVFDKLKSDYKSL

INKDFPDLSWADEVEQSEKSGFSFF

>Bahig_virus_(from_Oriolus_oriolus)_(YP009362040)

MDQEIIRRFNGRIRAVKEPTVARDILTDLYDERHNYFAREFCFANNLVYRNDVKATEIIGEMIPDFYIGDVEHIKYTPDN

YVKVGEKLFIIDFKVSTDDTSSIETYNKYMNAFADVFKDVDYEVVIVRANPMSNQIIIKSDEFRTYFGRVPPGLTFNWFF

DLRRMLMDKFKDNDEFQDMVDHGDFTLTAPWVSEDTPELYDHPIYREFIDSMGAKHEVLFNKTLNHDAYESKADKWNSNL

VYLKEKTEDYYNDFVRKMSDNVFIADGKYSKPTKDEIEQGWKEMTKRVHEEREVIQDVSKQKPSIHMIWSPNDPSASNEN

NAKILKLSKYLQNIKDTDKYAQNFQAIGKLMDFSENVFKYERFCNELKRDARAKIQKRDQKIEPIKIGDCTILWEQQFKY

EIIDHDKYSRSNFYKNYLGIGGHKEFKNRGLDDINLEKPKVLDFNDSSIIMASRVMMDKTKALLAQNSTLQKCGNLLDEY

QSKIENASPKTWDNIKTICGTRYWQAINDISTLTKNMLSVAQYNKFNTFRVVCCANNNMFGIVFPSSDIKTKKATMVFVT

ICIHDSIEDVLDSGALYRTYKSNGKYISISKGIRLDKERCQRLVSAPGPFMLTASLFKSDNDLASMPDIPNSPFLTSLSI

TKAMLSLTEPSRYMIMNSLAISSDVKNYMAEKFSPYTKTLFAVYMTNLIKKGCYEANEQKDSVELRDIFMSDIEITQKGV

RSERKLKSIWFPGLVSLKEYINQIYLPFYFNSKGLHEKHHVMIDLAKTVLEIEKDQRVNIPGIWGESFEKQTVNLDVLIH

SLTKNLMLDTSRHKHLRHRVESRNNFKRAISTISTFTSSKSCIKIGDFEKVKTEQVEKKRKAQKKADSTLRIANPLLAGE

MGAEEVHHADYIDLKKAVPEYVDMQSTKVFDRLYEKIKNGEIVDVTIEEIMKVMKNHKQFYFAYFNKGQKTAKDREIFVG

EFEAKMCLYGVERISKERCKLNPEEMISEPGDGKLKRLEQMAEDEIRFIIENVKSLKERDGEATAESEFLDKVSGENTRA

QKIEINADMSKWSAQDVMYKYFWLIAMDPILYPFEKKRIIYFLCNYMQKRLILPDELMYNILDQRYVRSDDIIMEMTNGF

KRNWVEIKRNWLQGNLNYTSSYLHSCAMSVYRDIFDITAKRLQGEVLVNSLVHSDDNQTSISMIQSRMDPDVIIQFAINT

FAKVCLAFGNQPNMKKTYITNFIKEFVSLFNIYGEPFSVFGRFILTCVGDCAYIGPYEDFASRLSATQTAIKHGCPPSIA

WVSIALNQWITYSTYNMLPGQNNDPARNLGMENRFEIPIELGGYLESDLSTIALLGLEAGNVTFLTRLIQKMSNIMYKKE

DIVGQTLRIDTWNLEQLTKSELFRLKLLRYVSLDSEMSIDNGLGETSDMRSRSLITPRKFTTVGSLAKLTSYRDYQKIAE

SPDETEELFEFFIANPELLVTKGETAEEFCKTIIYRYNSKKFKESLSIQNPVQLFIEQVLFSNKPTIDYTGLAERFVNAI

DFDENLDETTIAGRMTIAEALRRIADDITQLELDHEDVKTIYGFCITNDPMIITASNSMILQITGTGQERTGMSSNYMPE

MRNFRLMQHSPAVVLRGYIHGVDSVEFADQSELRRDIIHLEDFIKKTGLKERMEERIKTYHETKDDDSKRFDLKEITKFY

QVCYDYIKSTEHKVKVFILPIKVHTASDFCAVLHGNLLMDDLWFNIHYLRQIESSSHKGEITSFRNVEIDVAVECFKLLA

HFADWFIAGHSRKTYLKLMIQDYSYKGMTVKELYELLLKSEQRVNFIPILFRLGDLKQEDINKFDALKTNEKITWNNWQV

SQRMNTGLIDLTITGYNKRITITGEDDRLDEAMLYIKKDSYDQISNQSRKLLNTRHNLMFEKMKPVKFIDPREWYICHQK

GRRGKCDYVVLLGKQINSRNEQISQIHHRSQNYLVPVCPVGISIFEDSEATTLEDIEDMNLDNNSVTRLRVNRDEFAIVR

RAMITKMIHFSGPPMVAGIIDIEKLMHCRPLLNITYTTVRNSSLMDIVQIFSCTGDDDSKDGTFAFSEDPLEDEESQEID

SLPIFNVTFRKRGKSYMSYKNAIEEAIKVTTDEFKVAFDFSDDGFFSSKNLGIIQTISSLIKELGTNEWSTSLDKCIHLC

MYREGMDEIYHLFEMPKYFLRKVDETKADETVVNRGDWGDVVEQAEYEIINRINWEKVLGFIDALPNAKGAPWCEIFTHF

KKKAKELVLSKISALQKKMTLDDFAEELKVEGGKSMYQFF

>Matruh_virus_(from_Sylvia_curraca)_(AKO90169)

MDQEIIRRFNGRIRAVKEPTVARDILTDLYDERHNYFAREFCFANNLVYRNDVKATEIIGEMIPDFYIGDVEHIKYTPDN

YVKVGEKLFIIDFKVSTDDTSSIETYNKYMNAFADVFKDVDYEVVIVRANPMSNQIIIKSDEFRTYFGRVPPGLTFNWFF

DLRRMLMDKFKDNDEFQDMVDHGDFTLTAPWVNEDTPELYDHPIYREFIDSMGAKHEVLFNKTLNHDAYESKADKWNSNL

VYLKEKTEDYYNDFVRKMSDNVFIADGKYSKPTKDEIEQGWKEMTKRVHEEREVIQDVSKQKPSIHMIWSPNDPSASNEN

NAKILKLSKYLQNIKDTDKYAQNFQAIGKLMDFSENVFKYERFCNELKRDARAKIQKRDQKIEPIKIGDCTILWEQQFKY

DIIDHDKYSRSNFYKNYLGIGGHKEFKNRGLDDINLEKPKVLDFNDSSIIMASRVMMDKTKALLAQNSTLQKCGNLLDEY

QSKIENASPKTWDNIKTICGTRYWQAINDISTLTKNMLSVAQYNKFNTFRVVCCANNNMFGIVFPSSDIKTKKATMVFVT

ICIHDSIEDVLDSGALYRTYKSNGKYISISKGIRLDKERCQRLVSAPGLFMLTALLFKSDNDLVSMLDILNFSFFTSLSI

TKAMLSLTEPSRYMIMNSLAISSDVKNYMAEKFSPYTKTLFAVYMTNLIKKGCYEANEQKDSVELRDIFMSDIEITQKGV

RSERKLKSIWFPGLVSLKEYINQIYLPFYFNSKGLHEKHHVMIDLAKTVLEIEKDQRVNIPGIWGESFEKQTVNLDVLIH

SLAKNLMLDTSRHKHLRHRVESRNNFKRVISTISTFTSSKSCIKIGDFEKVKTEQVEKKRKAQKKADSTLRIANPLLAGE

MGAEEVHHADYIDLKKAVPEYVDMQSTKVFDRLYEKIKNGEIVDVTIEEIMKVMKNHKQFYFAYFNKGQKTAKDREIFVG

EFEAKMCLYGVERISKERCKLNPEEMISEPGDGKLKRLEQMAEDEIRFIIENVRSLKERDGEATAESEFLDKVSGENTRA

QKIEINADMSKWSAQDVMYKYFWLIAMDPILYPFEKKRIIYFLCNYMQKRLILPDELMYNILDQRYVRSDDIIMEMTNGF

KRNWVEIKRNWLQGNLNYTSSYLHSCAMSVYRDIFDITAKRLQGEVLVNSLVHSDDNQTSISMIQSRMDPDVIIQFAINT

FAKVCLAFGNQPNMKKTYITNFIKEFVSLFNIYGEPFSVFGRFILTCVGDCAYIGPYEDFASRLSATQTAIKHGCPPSIA

WVSIALNQWITYSTYNMLPGQNNDPARNLGMENRFEIPIELGGYLESDLSTIALLGLEAGNVTFLTRLIQKMSNIMYKKE

DIVSQTLRIDTWNLEQLTKSELFRLKLLRYVSLDSEMSIDNGLGETSDMRSRSLITPRKFTTVGSLAKLTSYRDYQKIAE

SPDETEELFEFFIANPELLVTKGETAEEFCKTIIYRYNSKKFKESLSIQNPVQLFIEQVLFSNKPTIDYTGLAERFVNAI

DFDENLDETTIAGRMTIAEALRRIADDITQLELDHEDIKTIYGFCITNDPMIITASNSMILQITGTGQERTGMSSNYMPE

MRNFRLMQHSPAVVLRGYIHGVDSVEFADQSELRRDIIHLEDFIKKTGLKERMEERIKTYHETKDDDSKRFDLKEITKFY

QVCYDYIKSTEHKVKVFILPIKVHTASDFCAVLHGNLLMDDLWFNIHYLRQIESSSHKGEITSFRNVEIDVAVECFKLLA

HFADWFIAGHSRKTYLKLMIQDYSYKGMTVKELYELLLKSEQRVNFIPILFRLGDLKQEDINKFDALKTNEKITWNNWQV

SQRMNTGLIDLTITGYNKRITITGEDDRLDEAMLYIKKDSYDQISNQSRKLLNTRHNLMFEKMKPVKFIDPREWYICHQK

GRRGKCDYVVLLGKQINSRNEQISQIHHRSQNYLVPVCPVGISIFEDSEATTLEDIEDMNLDNNSVTRLRVNRDEFAIVR

RAMITKMIHFSGPPMVAGIIDIEKLMHCRPLLNITYTTVRNSSLMDIVQIFSCTGDDDSKDGTFAFSEDPLEDEESQEID

SLPIFNVTFRKRGKSYMSYKNAIEEAIKVTTDEFKVAFDFSDDGFFSSKNLGIIQTISSLIKELGTNEWSTSLDKCIHLC

MYREGMDEIYHLFEMPKYFLRKVDESKPDETVVNRGDWGDIVEQAEYEIINRINWEKVLGFIDALPNAKGAPWCEIFTHF

KKKAKELVLSRISALQKKMTLEDFAEELKVEGGKSMYQFF

>Pueblo_Viejo_virus_(from_Aedeomyia_squamipennis)_(ATB53300)

MNETLEDDLKALEGRLKGVRETSDGKELFTAILVRRHDYFGYELCKAMDIPYRNDVKLVDILHECFHDIDTGDIVIPNIT

PDNFIYDGEILTILDYKVSVSLESTEHTIDKYTKELDKIRHFLPSRVELCIIRCHPRTTKLTFTNDRIKRMYANIQLELN

FEPFENLITELKDRFRDSDEFLAMIAHGDVTLTVKWCQTGCKELRQHPIFKEFYDSLDEDYQEMFDRSVEFSAYSAEKWN

QNLIRIRDQTAEDYKEFVKRNSSELFQTDGNYPEPSKEEILAGWDEMSERIQQTRQVSKDVNDQKPSIHFIWSEHNPKLP

THSTHKLMMVSKLLQNISSNSDYADDFRQIGKLMDIGDNALAYENHCEVLKLEARKNLKPIQNRKLNPRRIGNAQVLWEQ

QFLMSAEDMGKARKVHLLKEYFGIGRHKDFSHKTNTDINMDKPKILDFNDPDIIRMSQIMMEKTSGILSQQTTLKPNHAI

VDEYMDNIKEANENTFKTIKQILSTKYWSCISDISMLMKNILSVAQYNRSHTFRLAMCANNSLYALVLPSADIKSRKSTV

VFCIIALHTEEEAVFNPGCLHSTYKTFTSDKTGWLSISKAIRLDKERCQRIVTSPGLFLSTVTLLKQDNNSIDLNDVMNF

ALFTSLNVTKSMLSLTEPSRYMIMNSLAVSSHVKEYISEKFNPYTKTLFSVYMCNKIKTGCLNAFNQRQKIKLRDIFLTD

FDITQKGISDNRDLTSIWFPGFVTLKEYLNQIFLVFYLNPKGLHEKHHVMIDLLKTVIEIELDQRNNIPVPWSETPKKQT

VNLPIFLNSVAINLMHDTAYCTHLRSRIENRNNLRRSFSTISTFTSSKSCIKVGDFSNIKTEINRKIEKARKSEEKRSRI

ANPDFIHDEEYKNVVEHCNYDMMRKAIPEYTDHISTKVFDRLYELQKNKILTDEPVVGQILDMMVNHKQFYFSFFNKGQK

TAKDREIFVGEYEAKMCMYLVERIFKERSKVNPDEMISEPGDGKLKVLERKAEQEIRYLIERIKQRNSPIIEEIENLKES

VLFDKSKLDDLVNSKYHGLKLEINADMSKWSAQDVFFKYFWVIAMDPILYPEEKERIIYFFCNYMNKRLIIPDEVIYTIL

DQQIPYKNDMVMQVTNGLHSNTFNVKRNWLQGNFNYMSSYVHSCAMSVYKDILRAAAKRIEADILVNSMVHSDDNQTAIC

LINNRLDTRIFTKFAIQLFEKICLTFGCQANMKKTYVTNHIKEFVSLFNLYGEPFSVYGRFTLTAVGDCAYIGPYEDLAS

RISSTQTAIKHGCPASLAWLSISISHWITYLTYNMLPGQINDPSMSLGIEKRREIPIELGGYLEADLSMIALAGLESGNI

QFLINLLTKMVNPILFREDVISQCSDIKNWDLAKLTFSEIFRLKVLRFLALDAEMDCSDTMGETGEMRSRSLLTPRKFTT

AGSLRKLVSFGDFQSCQSEPEGINTVVEYMLEHPELLVTKGETVEDYRNIVLYRYNSKRFKESLSIQNPAQLFLEQILFS

HKPIIDYTGIQEKYLNLSDSTVTEDQPEILGRVTFPEAFRKIREDLSTLKLTINDVQTVYDFVILNDPMIIAIANAHLLS

NQGAMQDRLGSSCSTMPEFRNLRLITHSPALVLRSYSTGRYDVPGADPDEMRRDRTHLQEFIDNTKLLLKMEQRISQNEK

AKGTRDILFEIKEITKFYQVCYEYIKSTEHKVKVYILPVKCYTTTDFCAAIQGSLIRDKQWAIIQHIHPVSAGGHKGIVQ

KHTTNEIKLAEDCMRLIAFFIDTFIDENYRIYMFRRMVDEFSYSGRRVSDLLDIIATSKNRVDFLPILYRSGLLEQRDLD

SYDALKSSGRPTWNKWQTTRALDMGKIDLSIDTHRSSIRIIGEDRSLHYAELYLPSINYDSITRSGYKLLNAKHGLKFQS

MSEVSVKKDEWYITYQRKSRDNYVYKIFYGKKLLNPLPDHDLRYNRTTNPIVPVCHVIPMIRPDIDRIMIEDMDGLNIEN

FKIGVLNTGVGEQARLRRCMLHKMKNFEGPDIKSRVINLTKLMKDNTLMSLNYDNVCNVHLSQFCKLLDCNGNEGTLDDA

MYSFSDEPMEQTQVDEVECTPIFKVQYSVPGSRYLNYRNAIKELLSREIRQFRDAFTLVGNDFDSPENIGYLENIVCIID

QLQTNEWSTIMKKCIHICFLASDQDHVFHTFNLNKYFFEGNTPASGKLNWRNLKSFIISLPEIKTAPWRNIFSSFKLKCI

ALLDKEIAKVEVDQDFTRILAREDLRQSFLNFF

>Tete_orthobunyavirus_(from_Ploceus_cucullatus)_(AJT55735)

MDQEMIRRFNGRIRAVKEPTVAKDILSDLYNERHNYFAREFCFANNLTYRNDVKATEIIGEMIPDFYYGDVEHIKYTPDN

YVRVGDKMFILDFKVSTDDTSSIETYNKYMNAFSDVFKDVDFEVVIIRANPMSGQIIIKSDEFRTYFGRVPPGLSFNWFF

DLRRLLMNKFKDNDEFQDMVDHGDFTLTAPWVNEDTPELYDHPIYREFIDSMGARHEALFNKTLNHDAYESKADKWNSNL

VHLKEKTEEYYNDFVKSISDNVFMLDGNYSKPTKMEIEQGWQIMTERVHEEREVIQDVNKQKPSIHMIWTPNDPQSSNDN

IQKLIKLSKHLQDIKDTDKFAQNFQAIGRLMDFSKNVPKYEKFCNELKRDARAKIYKKDQRIEPLKIDDCTILWEQQFKY

EIIDHDKYSRAHFYKKFLGIGGHKEFKDRTLEDLDLDKPRILDFNDPSVIMAAKVMMEKTKALLAQDNMLERAGNLLDEY

RPKIENASPKTWQNIEVITKTRYWQAINDISTLLKNMLSVAQYNKFNTFRVVACANNNMFGIVFPSSDIKTKKATMVFVT

ICIHDEESNVLDPGCLYRTYKSRGKYISISKGIRLDKERCQRLVSAPGLFMLTSLLLKGDNDMVSMLDILNFAFYTSLSI

TKAMLSLTEPSRYMIMNSLAISSDVKNYMAEKFSPYTKTLFAVFMTNLIKRGCYEANEQKNNVELRDVFMSDIEITQKGV

RSERKLKSIWFPGYVNLKEYINQVYLPFYFNSKGLHEKHHVMIDLAKTVLEIEKDQRINIPGIWGDKFEKQTVNLDVLIH

SLAKNLILDTSRHKHLRHRVESRNNFKRAISTISTFTSSKSCIKIGDFENIKKQIVEKKRKALKKAEAALRIANPLIAGE

VTNNEVHHADYLDLKKAVPDYVDMQSTKVFDRLYEKIKEGEMGRTTIEEIMDTMRKHKQFYFAYFNKGQKTAKDREIFVG

EFEAKMCLYGIERISKERCKLNPEEMISEPGDGKLKRLEQMAEDEIRFIVENVKSLQPNEGDATRESEFLEKVAESGLKA

QKIEINADMSKWSAQDVMYKYFWLFAMDPILYPFEKKRILYFLCNYMQKRLILPDELMYNILDQRFVRENDIIVEMTNDF

KRNWVEIKRNWLQGNLNYTSSYLHSCAMSLYRDIFEVTAKRLKGEALVNSLVHSDDNQTAIAIIQSVVDPEVIIHFSIDT

FSKVCLTFGNQPNMKKTYLTNFIKEFVSLFNIYGEPFSVYGRFILTCVGDCAYIGPYEDFASRLSATQTAIKHGCPPSLA

WVSIALNQWITYSTYNMLPGQNNDPAKNLRIQDRFKIPIELGGYLDADLSTVALLGLESGNVSFLTRLIQKMSHIMYKKE

DIVGQTLRIDSWNLEKLDNTEKFKLKMLRYVTLDSEMSIDNGLGETSDMRSRSLITPRKFTTIGSLAKLTSYRDYQLVAG

SNEQTEELYEFFLQRPELLVTKGETLEEFCKTIVYRYNSKKFKESLSIQNPVQLFIEQVLFSNKPTIDYTGLADRFANSL

DFDENLEDNSISGRMTIADALSKISDDIESLPLTHEDIKTIYGFCITNDPLIITAANSLILQITGTGQERTCLSSNYMPE

MRNFRLMQHSPAVVLRAYIHGVESLAFADQAELSRDVIHLDTFIKKTKLKEKMEQRIKEYHENNDDDSKKFDLREVTKFY

QVCYDYIKSTEHKVKVFILPIKVHTAGDFCAVLHGNLLMDDQWFNIHFLRQIESSSHKGEITSLRNVEIDIACECFKLLA

HFSDWFIAGASRANFVRRIIREYTYKGLVVQELYNLLLKSEQRINFMPILFHLGDLTQEDINKFDALKTNEKITWNDWQV

SQRMNTGVINLTITGYNKKITIVGEDDRLDEAILYIKKDTYDQISNQSRKLLNTRHNLMFEKMRQVKFVDPREWYICYQK

GRRNKYDYVVLLGKQINARNEQIKVIQHRSQNYLIPVCPVGINVFEDSKAITLEDIKTKNIINESMTKLRINDDEIATVR

RAMITKMIHFSGPPMVAGVIDIEKLMHCRPLLSITYTTVKNSSLMDIVQIFHCEGDDESKGGTYTFSEEPMEDEESEEIA

SQPVFNVTFRKKGKKYMTYKNAIEEAIQKTTEEFQSAFDFSDDGFFSPKNLGIIKTISSLIKELGTNEWSTSLDKCIHLC

MHRAGLDDTYHLFDMPKYFLRKIDENKPDREVIVQGDWGEIVREAELEVVNRVNWEKVLSFIETLPEAKVAPWGEIFAHF

KRKARELVLRRIALCQKKMTLEEFAEELQIEEGHSMYHFNA

>Gamboa_virus_(from_Aedeomyia_squamipennis)_(ATB53299)

MNETLEEELKALEGRLKGVRETSDGKELFTAILVRRHDYFGYELCKAMDIPYRNDVKLVDILHECFHDIDTGDVIIPNIT

PDNFIFDGEVLTILDYKVSVSLESTEHTIEKYTKELDKIRNFLPSRVELCIIRCHPRTTKLTFTNDRIKRLYANIQLELN

FEPFENLITELKDRFRDSDEFLAMIAHGDVTLTVKWCQTGCPELKQHPIFNEFYESLEDKYKEMFDRSMQFSAYSAEKWN

QNLIKIRDQTSDEYKEFVRRNSSELFQTDGNYPEPSKEEILAGWDEMSERIQQTRQMSKDINDQKPSIHFIWSEHNPKLP

THSTHKLMMVSKLLQNLSSNSDFADDFRQIGKLMDIGDNVLVYENHCEALKLEARKNLKPIQNRKLNPRRIGSAQVLWEQ

QFLMSAEDMGKARKVHLLKEYFGIGRHRDFSHKTNSDINMEKPKILDFNDPDIIRMSQIMMEKTSGLLSQQTTLKPNHAI

VDEYMDNIKEANENTFKTIKQILSTKYWSCISDISMLMKNILSVAQYNRSHTFRLAMCANNSLYALVLPSADIKSRKSTV

VFCIIALHTEEESVFNPGCLHATYKTFTSDKTGWLSISKAIRLDKERCQRIVTSPGLFLSTVTLLKQDNNSIDLNDVMNF

ALYTSLNVTKSMLSLTEPSRYMIMNSLAVSSHVKEYISGKFNPYTKTLFSVYMCNKIKIGCLNAFNQRQKIKLRDIFLTD

FDITQKGISDNRDLTSIWFPGFVTLKEYLNQIFLVFYLNPKGLHEKHHVMIDLLKTVIEIELDQRNNIPVPWSDTPKKQT

VNLPIFLNSVAINLMHDTAYCTHLRSRIENRNNLRRSFSTISTFTSSKSCIKIGDFSSLKTEINRKIEKARKAEEKRSRI

ANPDFIHDEEYKNVVEHCNYDMMRKAIPEYTDHISTKVFDRLYELQKNGTLTDEPVVGLILEMMVNHKQFFFSFFNKGQK

TAKDREIFVGEYEAKMCMYLVERIFKERSKVNPDEMISEPGDGKLKVLERKAEQEIRFLIEHIKQRNSPIMEEIENLKES

VLFDKSKLEDLVNSKYHGLKLEINADMSKWSAQDVFFKYFWVVAMDPILYPEEKERIIFFFCNYMNKRLIIPDEVIYTIL

DQQIPYKNDMVMQVTNGLHSNTFNVKRNWLQGNFNYMSSYVHSCAMSVYKDILRTAAKKIEADIMVNSMVHSDDNQTAIC

LINNRLDTRVFTKFAIQLFEKICLTFGCQANMKKTYVTNHIKEFVSLFNLYGEPFSIYGRFTLTAVGDCAYIGPYEDLAS

RISSTQTAIKHGCPASLAWLSISISHWITYLTYNMLPGQINDPSVSLGIENRREIPIELGGYLEANLSMIALAGLESGNI

QFLINLLTKMVNPILFRENVVSQCSDIKNWDLTRLTFSEIFRLKVLRFLALDAEMDCSDTMGETGEMRSRSLLTPRKFTT

AGSLRKLVSFGDFQACQSEPEGINTVVEYMIEHPELLVTKGETAEDYKNIVLYRYNSRRFKESLSIQNPAQLFLEQILFS

HKPIIDYTGIQEKYLNLSDSTVTEDQPEILGRVTFPEAFRKIREDLSTLKITINDIQTVYDFVILNDPMVIAIANAHLLS

NQGAMQDRLGSSCSTMPEFRNFRLITHSPALVLRSYSTGRYDVPGADPDEMRRDRTHLQEFIDNTKLLLKMEQRIEQNEK

AKGSRDILFEIKEITKFYQVCYEYIKSTEHKVKVYILPVKCYTTTDFCAAIQGSLIKDKQWAVIQHIHPVSAGGHKGIVQ

KHTTNEIKLAEDCMRLIAFFIDTFIDENHRIYMFKRMVDEFTYSGRKVSDLLEIIATSKNRVDFLPILYRSGLLEQRDLD

SYDALKSSGRPTWNKWQTTRALDMGKIDLSIDTHRSSIRIIGEDRNLHYAELYLPSINYDSITRSGYKLLNAKHGLKFQS

MSEVSVRKDEWYITYQRKSRDNYVYKIFYGKKLLNPLPDHDLRYNRTTNPIVPVCHVIPMVRPDIDRIMLEDMDSLNIEN

FKIGVLNTGIGEQARLRRCMLHKMKNFEGPDIKSKVINLTKLMKDNTLMSLNYDNVCNVHLSQFCKLLDCNGNEGTLDDA

MYSFSDEPMEQTQIDEIECTPIFKVQYSVPGSRYLNYRNAIKELLSREIRQFREAFTLVGDDFDSPENIGYLENIICIID

QLQTNEWSTIMKKCIHICFLASNQDHVFHTFSLNKYFFEGNTPASGRLNWRNLKSFIISLPEIKSAPWRNIFSSFKLKCI

ALLDREIAKVEVNQDFSRILAREDLRQSFFNFF

>Tomato_spotted_wilt_orthotospovirus_(from_Solanum_lycopersicum)_(Tospoviridae)_(NP049362)

MNIQKIQKLIENGTTLLLSIEDCVGSNHDLALDLHKRNSDEIPE

DVIINNNAKNYETMRELIVKITADGEGLNKGMATVDVKKLSEMVSLFEQKYLETELAR

HDIFGELISRHLRIKPKQRNEVEIEHALREYLDELNKKSCINKLSDDEFERINKEYVA

TNATPDNYVIYKESKNSELCLIIYDWKISVDARTETKQWRNTYKNIWKSFKDIKVNGK

PFLEEHPVFVSIVILKPIAGMPITVTSSRVLEKFEDSPSALHGERIKHAKNAKLLNIS

YVGQIVGTTPTVVRNYYANTQRIKSEVRGILGDDFGSKDVFFSHWTSKYKERNPTEIA

YSEDIERIIDSLVTDEIPREEIIHFLFGNFCFHIETMNDQHIADKFKGYQNSCINLKI

EPKADLADLKDHLIQKQQIWESLYGKHLEKIMLRIREKKRKEKEIPDITTAFNQNAAE

YEERYPNCFNDLSELKLTFHDLVPSLKIELSSEVDYNNAIINKFRESFKSSSRVIYNS

PYSSINNQTNKARDITNLVRLCLAELSCDTTKMEKQELEDEIDINTGSIKVERTKKSK

EWNKQGSCLTRNKNEFCMKDTGRENKTTYFKGLAVMNIGMSSKKRILKKEEIKERISK

GLEYDTSERQADPNDDYSSIDMSSLTHMKKLIRHDNDDSLSGKRFKGSFFLLHNFNII

EDGKITSVFNNYAKNPECLYIQDSVLKTELETCKKINKLCNDLAIYHYSEDMMQFSKG

LMVADRYMTKESFKILTTANTSMMLLAFKGDGMNTGGSGVPYIALHIVDEDMSDHFNI

CYTKEIYSYFRSGSNYIYIMRPQRLNQVRLLRLFKTPSKVPVCFPQFSKKANEIGKSL

KNKDIEKVNLFSMTMTVKQILINIVFSSVMIGTVTKLSRMGIFDFMRYAGFLRLSDYS

NIKEYIRDKSDPDITNCGRYLFRNGIKKLLFRMEDLNLSTNAKPVVVDHENDIIGGIT

NLNIKCPITGSTLLTLEDLYNNVYLAIYMMPKSLHNHVHNLTSLLNLPAEWELKFRKE

LGFNIFEDIYPKKAMFDDKDLFSINGALNVKALSDYYLGNIENVGLMRSEIENKEDFL

SPCYKISTLKSSKKCSQSNIISTDEIIECLQDAKIQDIENWKGNNLAIIKGLIRTYNE

EKNRLVEFFEDNCVNSLYLIEKLKEIISSGSITVGKSVTSKFIRNNHPLTVETYLKTK

LYYRNNVTVLKSKKVSEELYDLVKQFHDMMEIDLDSVMNLGKGTEGKKLTFLQMLEFV

MSKAKNVTGSVDFLVSVFEKMQRTKTDREIYLMSMKVKMMLYFIEHTFKHVAQSDPSE

AISISGDNKIRALSTLSLDTITSYNDILNKNSKKSRLAFLSADQSKWSASGLTTYKYV

LAIILNPILTTGEASLMIECILMYVKLKKVCIPTDIFLNLRKAQQTFGENETAIGLLT

KGLTTNTYPVSMNWLQGNLNYLSSVYHSCAMKAYHNTLECYKNCDFQTRWIVHSDDNA

TSLIASGEVDKMLTDFSSSSLPEMLFRSIEAHFKSFCITLNPKKSYASSSEVEFISER

ISKWSDYSSLLQAFSKLLHRIFAYKLFDDLMSLSIHVTMLLRKGCPNEVIPFAYGAVQ

VQALSIYSMLPGEVNDSIRIFNKLGVSLKSNEIPTNMGGWLTSPIEPLSILGPSSNDQ

IIYYNVIRDFLNKKSLEEVKDSVSSSSYLQMRFRELKEKYERGTLEEKDKKMIFLINL

FEKASVSEDSDVLTIGMKFQTMLTQIIKLPNFINENALNKMSSYKDFSKLYPNLKKNE

DLYKSTKNLKIDEDAVLEEDELYKKIASSLEMESVHDIMIKNPETILIAPLNDRDFLL

SQLFMYTSPSKRNQLSNQSTEKLALDRVLRSKARTFVNISSTVKMTYEENMEKKILEM

LKFDLDSYCSFKTCVNLVIKDVNFSMLIPILDSAYPCESRKRDNYNFRWFQTERWIPV

VEGSPGLVVMHAVYGSNYIENLGLKNIPLTDDSINVLTSTFGTGLIMEDVKSLVNGKD

SFETEAFSNSNECQRLVKACNYMIAAQNRLLAINTCFTRKSFPFYSKFNLGRGFISNT

LALLSTIYSKEESYHFVSTASYKLDKTIRTVVSAQQDMNLEKILDTAVYISDKLQSLF

PTITREDIVLILQNVCLDSKPIWQSLEDKMKKINNSTASGFTVSNVILSHNSELNTIQ

KQIVWMWNMGLCSHRTLDFVIRYIRRRDVRYVKTEEQDESGNYVSGTMYKIGIMTRSC

YVELIASDQDVAVSLRTPFEILNEREYLFDTYRESIEKLLAEIMFDKVNIINQTTTDC

FLRTRRSCIRMTTDNKMIVKVNATSRQIRLENVKLVVKIKYENVNSDVWDIIESQKSL

VLRLPEVGEFFSDMYKTADSETETIKTIKNRLMTSLTFIEAFGNLSQQIKEIVDDDIR

ETMDEFLMNIRDTCLEGLENCKSVEEYDSYLDENGFNDTVELFENLLRTHDNFENEYS

PLFSEIVDKAKQYTRDLEGFKEILLMLKYSLINDASGFKSYRATGMHAVELMAKKHIE

IGEFNLLGMIQLIKACETCHNNDSILNLASLRNVLSRTYATFGRRIRLDHDLDLQNNL

MEKSYDFKTLVLPEIKLSELSREILKENGFVISGENLKMDRSDEEFVGLASFNVLRLD

EEEMYEGLIKEMKIKRKKKGFLFPANTLLLSELIKFLIGGIKGTSFDIETLLRNSFRP

DIFSTDRLGRLSSSVPALKVYATVYMEYKNVNCPLNEIADSLEGYLKLTKSRSKEHFL

SGRVKKALIQLRDEQSRTKKLEVYKDIANFLARHPLCLSEKTLYGRYTYSDINDYIMQ

TREIILSKISELDEVVETDEDNFLLSYLRGEEDAFDEDELDEEEDTD

>Groundnut_ringspot_virus_(from_Arachis_hypogaea)_(AST36116)

MNIQKIRKLIENGTTLLLSIEDCVGSNHDLALDLHKRNSDEIPEDVIINNNAKNYETMRELIVKISTDGEGLNTGIATVD

VKKLNELVSLFEQKYLETELSRHDLFGELVSRHLRIKPKQRNEVEIELALRDYLEELNKKQCINSLSDDEFERINREYVA

TNATPDNYVIYKESKNSELCLMIYDWKISVDAKTETKTMEKYYKNIWKSFKDIKVNGKPFLEDHPVFITIVILKPMGGMP

ITVTSSRVLGKFEDSPSALHGERSKLAKNAKLLNIYHVGMIVGTTPTIVRNYYANTQKLKSEVRGILGDDFGSKDVFFSH

WASKYKDRNPTEIAYSEDIERIIESLTTEEMTKDEIVHFLFGNFCYHIETMNDQHIADRFKGYQDACLNLKVVPKTDLSD

LKDHLIQTQNVWESLYGKHLDKILQRIKKKKSKEREIPDITTAFNQNAVEYEEKYPNCFTNDLSETKTNFSMTWSPSFEK

VELNSNIDYNNAIIKKFRDSFQTTSRITYNSPYSSVHNQTNKARDVTNLVKLCLTELSCDTTKFNKQELEDEIDINTGSI

KVERTKKSQEWCKHNSCLTRNKNEFCMKDTGKENKAVYFKGLAVMNIGMSSKKRILKKEEIKERISKGLEYDTSIRQTDP

NDDYTSVDMASLTHMKKLIRHDNEESLSWCEKIKDSLFVLHNGDIREEGKIASVYNNYAKNPECLYTQDSVLKTEMETCK

KINKLCNDLAIYHYSEDMMQFAKGLMVADRYMTKESFKILTTANTSMILLAFKGDGMNTGGSGVPYIALHIVDEDMSEQF

NICYTKEIYSYFRSGSNYIYIMKPQRLNQVRLLSLFKSPSKVPVCFAQFSKKASELEKWLKNKDIEKVNVFSMTVTVKQI

LINIVFSSVMIGTVTKLSRMGIFDFMRYAGFLPLSDYSNIKEYIRDKFDPDITNVADIYFVNGIKKLLFRMEDLNLSTNA

KPVVVDHENDVIGGITNLNIKCPITGSTLLTLEDLYNNVYLAIYMMPKSLHNHVHNLTSLLNVPAEWELKFRKELGFTIY

EDIYPKKDMFDDKDLFSINGLLNVKALSDYYIENVENVGLMRSEIENKEDFLSPCYKITTLKSSKKCSQSNIINTDDIIE

CLQTVKVKDVENWKGNTLAIIKGLIRTYNEEKNRLIEFLEDNCVNSLYLLEKLKEIISSGSVTVGKSMTSKFIRNNHPLT

VETYLKTKLYYRNNVTVLKSKKVSEELYDLVKQFHNMMEIDIDSVMNLGKGLEGKKHTFLQMLEFVISKAKNVTGSVDFL

VSVFEKMQRTKTDREIYLMSMKVKMMLYFIEHTFKHVAQSDPSEAISISGDNKIRALSTLSLDTITSYNDILNKNSRKSR

LAFLSADQSKWSASDLTYKYILAILLNPVLTTGEASLMVECLLMYVKLKKVCIPTDIFLNLKKSQSTFGQNETAIGLLTK

GLTTNTYPVSMNWLQGNLNYLSSVYHSCAMKAYHKTLECYKGCDFQTRWIVHSDDNATSLIANGEVDKMLLDFSSSSLPE

MLFRSIEAHFKSFCITLNPKKSYASSSEVEFISERIVNGAIIPLYCRHLANCCTESSHISYFDDLMSLSIHVTMLLRKGC

PNEVIPFAYGAVQTQALSIYSMLPGEVNDSIRIFQKLGVSLMPNEIPTNMGGWLTAPIEPLSILGPSSNDQIIYYNVIRD

FLNKKSLEEVKNSVSTLGYLQMRFKELEGKHKKGTLDIKDKKMIFLINLFEKASVSEDSDVLTIGMKFQTMLTQIIKLPQ

FINENALNKMSSYKDFSKLYPNLKKNEDLYKSTKNIKFNEDSLLEEDELYEKVASSAEMESVHNIMIQNPETILIAPLND

RDFLLSQLFMYTSPSKRNQLSNQSTEKLALDRVLRSKARTFVDIDSHVKMTYEENMEKKILEMQKFDPGSYCSFKTCINL

VIKDVNFSMLTPILDAAYPCESRKRDNYNFRWFQTEKWIPVVEGSPGLVVMHAIYGSNYIENLGLKNIPLTDDSINVLTS

TFGTNLLMDDVKSFVSGSSSFETEAFINANNCQRLVKACNYMITAQNRLLAINTCFSRKSFPFYSKFNLGRGFISNTLAL

LSTIYSKEESYHFVSTANYKLDKTIRTVLNAQQDMNLEKILDTAVYISDKLQSLFPTITREDISLILQNICLDSKPIWES

LEEKMRKINNSTGSGFTVSNVILSHNSELNTIQKQVVWLWNMGLCSNRTLDFVIRYIRRSDVRYVKTEEQDELGNYISGT

IYKIGIMTRSCYVQLIASDHDVAVTLRTPFEILNERDYLYDTYRESIEKLLQKFMFDKINIIKSKQPQIVFLEPGEACLR

MTTDNKMIVKVNATPKQIRLENVKLVIKIKYENVNSDVWDIIESQKALVLREPEVGECFSDMYKTVDSEAEAIKVIKHGL

MNSLTFIETFGNLSKQIDGIEDETVRETMHDFLMNIRDTCLEGLENCKSIEEYDTFLAENGFDDTVELFEDLLKTQDSFE

NEYSPLFSEIVDKAKQYTRDLEGFKEILLMLKYSLINDASGFKSYRATGAHAVELMAKKHIEIGEFNLLGMIQLIKACET

CHNNDSILNLASLRNVLSRTYATLGRRINLNHELDLQNDLMEKSYDFKTLVLPDIKLSDMSREILKDNGFIISGENLKID

KSGDEFEGLANFNMLRLDEEEMYEGLIKEMKIKRKKKGFLFPANTLLLSELIKFLIGGIKGTSFDIETLLRNSFRPDTYS

TDRLGRLSSSVPALKVYATVYMEYKNTSCPLNEIADSLEGFLKLTKSKAKEQFLDGRVKKALVQLRDEQSRSKKLEVYRN

IADFLSRHPLCLSEKTLYGRYTYHDINDYIMQTREIILSKINELDDYVEMDDDDFLLGYLKGEEDAYDEDEESD

>Chrysanthemum_stem_necrosis_virus_(from_chrysanthemum)_(AII20576)

MNTQKIRKLIENGTTLLLSIDDCVGANHDLSMDLHKRNSDEIPEDIIINNNAKNYETMRELIVKITADGEGLNNGIATVD

IKKLNELVSLFEQKYLETELSRHDIFGELISRHLRIKPKHRNEVEIELALREYLEELNKKSCINNLSDNDYERVSKEYVA

TNATPDNYVIYKESKDSELCLIIYDWKISVDAKTETKTMEKYYKNIWKSFKDIKVNGKPFLEDHPVFISIVILKPIGGMP

VTVTSSRVLGKFEDSPSALYGERVKHARNSKLLSINNVGQIAGTTPNIIRSYYASTQKIKSEVRGILGDDFGSKDVFFSH

WTNKYRDRDPTETVYSEDIERIVDSLVTTEISREEIIHFLFGNFCFHIETMNDQHIADKFKGYQNSCINLKIKPKEDLAD

LKDHLIQTKEIWESLYGKHLDKIISRIRDKKKKEKEIPDITTAFNQNAIEYEEKYPGCFTNDLSETKTNFSMTWSPSFEK

IELDSDIDYNNAIIKKFRESFKTASRISYNSPYNSVNSQNNKARDITNLVQLCLTELSCDTTKMSKQELEDEIDINTGSI

KVERTKKSQEWQKINSCLTRNKNEFCMEETERETKTIYFKGLACMNIGMSLKKRVLKKEEMKERISKGLEYDTSERQVDP

NDDYSSVDMSSLTHMKKLIRHDNEESLTWCDRIKESLFVLHNGDIRNDGKISAVYNNYAKNPECLYTQESVLKSELDTCK

KINKLCNDLSIYHYSEDMMQFAKGLMVADRYMTKESFKILTTANTSMLLIAFKGDGMNTGGSGVPYIAVHIVDEAMSEQF

NICYTKEIYSYFKSGSNYIYIMRPQRLNQVRLLSLFKSPSKVPVCFSQFSKKASELEKWLKNKDIDQVTALSMTMTVKQI

LINIVFSSIMIGTVTKLSRMGIFDFMRYAGFLPLSDYSNIKEYIRDKFDPDITNVADIYFVNGIKKLLFRMEDLNLSTNA

KPVVVDHENDIIGGITNLNIKCPITGSTLLTLEDLYNNVYLAIYMMPKSLHNHVHNLTSLLNVPAEWELKFRKELGFTIF

EEIYPKKEMFDDRDLFSINGALNVKALSDYYLENITNVGLMRSEIENKEDFLGPCYRISTLKSSKKCSQSNIISTDEIID

CLQNIKISEVEDWKGNTLAIIKGLIRTYNEEKNRLMEFLEDNCVNSLYLMEKLKEIVNSGSVTVGKSMTSKFIRNNHPLT

VETYLKTKLYYRSNVTILKSKKVSEELYDLVKQFHNMMELDMESVMNLGKGLEGKKHTFLQMLEFVMSKAKNVTGSVDFL

VSVFEKMQRTKMDREIYLMSMKVKMMLYFIEHTFKHVAQSDPSEAISISGDNKIRALSTLSLDTITSYNDILNKNSKKSR

LAFLSADQSKWSASDLTYKYVLAIILNPVLTSGEASLMIECILMYIKLKKVCIPTDIFLNLRKSQDTFGLNETAIGLLTK

GLTTNTYPVSMNWLQGNLNYLSSVYHSCAMKAYHRTLECYKDCDFQTRWIVHSDDNATSLIANGEVDKMLADFSSLSLPE

MLFRSIEAHFKSFCITLNPKKSYASSSEVEFISERIVNGAIIPLYCRHLANCCTESSHISYFDDLMSLSIHVTMLLRKGC

PNEVTPFAYGAVQVQALSLYSMLPGEVNDSIRIFSKLGVSLKSNEIPTNMGGWLTSPIESLSILGPSSNDQIIYYNVIKE

FLNKKSLDEVKNNVSTLGYLQKRFEELEEKSKKNTLEEKDKKMIFLINLFEKASVSEDSDVLTIGMKFQTMLTQIIKLPQ

FVNENALSKMSSYRDFSKIYPNLKKNEDLYKSTKNVRIDEDSILEEDELYDKISSSLEMESVHTIMIENPETILIAPLND

RDFLLSQLFMYTSPSKRNQLSNQSTEKLALDRVLRSKARTFIDIDSKTKLTYEENMEKKIKEMLDFEPGSYCSFKTCINL

VIKDVNFSMLMPILDSAYPCESRKRDNYNFRWFQTEKWIPVVEGSPGLVVMHAVYGPNYIENLGLKNIPLTDDSINVLTS

TFGTNLLMDDVKSFVAGRKSFETDIFKNSNDCQKLVKACNYMITAQNRLLAINTCFSRKSFPFYSKFNLGKGFVSNTLAL

LSTIYSKDESYHFVSTASYKLDKTIRTVINAQQDLNLEKILDTAVYISDKLQSLFPTISRDDIKIILQNVCLDSRPIWQS

LEEKMKKINHSTGSGYTVSNVILSHNSELNTIQKQIVWLWNMELCSTKTLNFVIRYIRRSDVRYVKTEEQDESGNYVSGT

LYKIGIMTRSCYVQLIASDQDVAVSLKTPFEILNERDFLFDTYRESIEKLLQKFMFDKINIIKSKQPQITFLEPGDACIR

MTTDNKMIVKVNASPRQIRLENVKFVVKIKYENVNSDVWDIIESQKSLVLRLPETGECFSDMYKTVDSEAETIKTIKSRL

ITSLTFIESFGSLSDQVLEISHEQVKDTMFDFLMNIRETCLEGLENCRSIEEYDEFLDDNGFNATVELFEDLLRTQDSFE

NEYSPLFSEIVDRAKQYTRDLQGFKEILLMLKYSLINDASGFKSYRATGVHAVDLIVKKHIEIGEFNLLGMIQLIKACET

CHNNDSILNLASLRNVLSRTYATFGRKIILDHELDLQNDLMEKSYDFKTLVLPEIKITEFSKDILKENGFIISGENLKIN

KSDEEFEGLANFNVLRLDEEEMYEGLIKEMRIKRKKKGFLFPANTLLLSELIKFLIGGIKGTSFDIETLLRNSFRQDIYS

GDRLGRLSSSVPALKVYSTVYMEYKNVNCPLNEIADSLEGYLKLTKSKPKEQLLDGRVKKALIQLREEPSRSKKLEVYKD

IASFLSRHPLCLSERTLYGRYTYHDINEYIMQTREIILSKINELDEIVETDEDSFLLSYLRGEEDAFDEEEEVE

>Zucchini_lethal_chlorosis_virus_(from_Cucurbita_pepo)_(YP009316178)

MNIQKIKRLIENGTTLLLSIEDCVGSNHDLALDLHKRNSEEIPEDIVINNNAKNYETMRELIAKITADGEGLNTGIATVD

IKKLNELVSLFEQKYLETELARHDIFGELVSRHLRIKPKYRTEVEIETALREYLEELNKKQCSNKLSDADFERVSKEYVA

TNATPDNYVVYKESRGSELCLMIYDWKISVDAKTETKTMEKYYKNVWKSFKDIQVDGKPFLENHPVFISIVILKPIGGMP

ITVTSSRVLQKFEDSPSALHGERVRHARNARLLSIDYVGQISGTTPTVVRNYYANTQKLKSEVRGILGDDFGSKDVFFSH

WTNKYRERDPTEIAYSEDLEKIIDSLATDEISKEEVIHFLFGNFCFHVETMNDQHIADKFKGYQNACINLKIQPKIDLPE

LKDHLIKSKEVWDSLYLKHLDKILSRVKTKKEKEKEIPDITTAFNLNAIEYEEKYPGCFTNDLSETKTNFSMTWSPNFEK

MEVNADIDYNNAIIKKFRDHLNNPSKVFYNCPYSSITNQTNKARDVTNLARLCLTELSCDTTKMSKQELEDEIDINTGGI

KVERTKKSQEWVKVGKCLTRNKNEFCMREASREEKSIYFKGLSVMNVGMGAKKKVLKRQEMKEKISQGIEYDTSEKRTDE

NDDYSTVDVSSLTHMKKLIRHDNEESLSWCDKIKESLYVLHNGDIRVDGKIAAVYNNYAGNPECLYTQDSVLKEEMDTCK

KINKLCNDLAIYHYSEDMMQFAKGLMVADRYMTKESFKILTTSNTSMILLAFKGDGMNTGGSGVPYIALHIVDEDMAEQF

NICYTKEIYSYFRSGSHYVYIMRPQRLNQVRLLSLFKSPSKVPVCFSQFVKKASELEKWIRNKDIDNISTFSMTMTAKNI

LENIVFSSVLIGTVTKISRMGIFDFMRYAGFLPLSDYSNIKEYIRDKFDPDITNVADTYFVHGIKKLLFRMEDLNLSTNA

KPVVVDHENDIIGGITNLNIKCPITGSTLLTLEDLYNNVYLAIYMMPKSLHNHVHNLTSLLNVPAEWELKFRKELGFNLH

EDIYPKKEMFDDRGLFSINGALNVKALSDYYLENIDNVGLMRSEIENKEEFLSPCYKISTLKSSKKCSQSNIISNADIIE

CLQNAKISEIGNWKGNTMAIMKGVIRTYNEERNRLVDFLEDNCDSPLQLLNKIKEVVNSGSVTIGKSMTSKFIRNNHPLT

VETYLKTKLYYRDNITVLKSKKVSEELYDLVKQFHNMMELDMDSIMNLGKGLEGKKHTFLQMLEFVMSKAKNVEGSVDFL

VSVFEKMQRTKMDREIYLMSMKVKMMLYFIEHTFKHVAQNDPSEAISISGDNKIRALSTLSLDTITSYNDILNKNKSKSR

LAFLSADQSKWSASDLTYKYVLAIILNPVLTSGEACLMIECMLMYVKLKKVCIPTDIFLNLKKAQETYGQNETAIGLLTK

GLTTNTYPVSMNWLQGNLNYLSSVYHSCAMKAYHKTLECYKNCDFQTRWIVHSDDNATSLIANGKVDDMLTDFSSSSLPE

MLFRSIEAHFKSFCITLNPKKSYASSSEVEFISERIVNGAIIPLYCRHLANCCTESSHISYFDDLMSLSIHITMLLRKGC

PNEVIPFAYGAIQVQALSLYSMLPGEVNDSIRIFKKLGVSLSSNEIPTSMGGWLTSPIEPLSILGPSSNDQIIYYNVIRD

FLNKKSLEEVKDCVSSFGYLQMRFNELEEKHKKGTLEEKDNKMIFLINLFEKASVSEDSDVLTIGMKFQTMLTQIIKLPQ

FINENALNKMSSYKDFAKLYPSLKKNEDLYRSTKNVRLDEEMIIDEDRLYDAISSSIEMEAVHTIMIENPETILIAPLND

RDFLLSQLFMYTSPSKRNQLSSQSTEKLALDRVLRSKARTFVDTDSNVKMTYDENMEKKIKEMMKFEPGSYCSFKTCINL

VVKDVNFSMLVPILDSAYPCESRKRDNYNFRWFQTEKWIPVVEGSPGLVVMHAVYGSNYIEKLGLKNIPLTDDSINVLTS

TFGTNLLMDDVKSFVAGNGSFETENFSNSSNCQRLVKACNYMITAQNRLLAINTCFSRKSFPFYSKFNLGRGFVSNTLAL

LSTIYSKEESYHFVSTASYKLDKTIRTVISAQQDLNLEKILDTAVYISDKLQSLFPTITWKDVKLILENVCLDSRPIWQS

LEEKMRKINNSTKSGYTVSNVILSHNSELNTIQKQIVWLWNMGLCSDRTLDFVIRYIRRSDVRYVKTEEQDELGNYISGT

MYKIGIMTRSCYVQLIASDQDVAVSLKTPFEILNERDFLFDTYRESIEKLLQKFMFDKTNIIKSKQTHITFLEPGDACIR

MTSDNKMIVKVNASPRQIRMENVKLVVKIKYENVNSDVWEIIESQKSLVLRYPETGECFSDMYKTVDSEAEAIKTIKSKL

MTSLTFIEAFGNFSKQINEIVDETVRETMHDFLMNIRDTCLEGLENCKSIEEYDEFLDDNGFNDTVELFEDLLRTQDSFE

NEYSPLFSEIVDRAKQYTRDLNGFKEILLMLKYSLINDASGFKSYRATGSHATDLMMKKHIEIGEFNLLGMIQLIKACET

CHNNDSILNLASLRNVLSKTYATFGRRLVLNHDLDLQNDLMEKSYDFKTLVLPEIQLSDFSKEILKGNGFVVSGENLKID

KSADEFEGLASFNALRLDEEEMYEGLIKEMKIKRKKKGFLFPANTLLLSELIKFLIGGIKGTSFDIETLLRNCFRPNIYS

RDRLSRLSSSVPALRVYSTVFMEYKDVNCPLSEIADSLEGFLKLTKSKSREQFLDGRVKKALIQLRDEQSREKKLEVYKD

IAEFLSRHPLCLSEKTLYGRYTYQDINDYIMQTREIILTKINELDESMEDDDDNFLLSYLKGEEEALDEDEVDYSD

>Melon_severe_mosaic_tospovirus_(from_Nicotiana_benthamiana)_(YP009346017)

MNTQKIRSLIDNGGTLLRTIEDCIGSNLDLANDLHKYNSEEIAEDIIINNNAKNYESMRELLAKICQDGEGLNSGLATVD

FKKINDDVTLLEQKYLATELARHDIFGELISRHLHLKPKHRTEVELEHAIRDYFSELNKKSCPNKISDEDLARLNIEYVA

INATPDNYVIYKDHKGGNLCLMLYDWKISVDAKTETKTTEKYYKNIWKSLKDIKINDELLLEYCPIFITIVTLKPMGNMP

ITATTSRVLGKFEDSPSALHGNRVKHARNSRLVSADYAGQISGTTPSIIRNFYANTQKLKLEFRSIIGDDFGSKDIFFSH

WTSKYKERHPMEAAYSEDLESIINYISTEDFPKEEVVHFLFGNFCFHIETMNDQHIADRFRGYCECCLSMHVRPKENIAV

LKDHLLASKPKWDSLYEGHLKKIIERVAQKKSKEKTIPNITAAFNMNAQEYEAKYPGCFTSNLSETKTNFSLTWSPSFET

IEVDKIYDYNNSVIQLFRNAFKSVAKITYNCAYGSIQDQANKARNITNLVDLCLTKLSCNTTSMIKESLEDEIDINTGGI

KAGRTKKAQEWQSFNGCLTRNKNEFSIRDCAKEDKAVYYRGLSCMNIGMGKKQKVQKREELKSKITLGLEYDTSQRQADP

NDDYSSLDLGSYTHSKILIRHDNEESLSWCEKIRDSLFVLHNGDLREEGKVATVYNNYTKNPETLYTQDSIIKGEMQCCK

KINKLCNDLAIYNYSEDMMQFSKGLMVADRYMTKESFKILTTSNTSMILLAFKGDGMNTGGSGVPYIAIHLVDGDMAESF

NICYTKEIYSYFKTGDKVLYIMRPQRLNQVRLLSLFKSPSKVPVCFAQFCKKASELDKWLKTRDIDEVNTLSMNLPVKQI

LRNIVFSSVLIGTVTKLSRMGIFDFMRYAGFLPLSDYSNIKEYIRDKFDPDITNVADMFFVSGIKKLLFKMENLNLSSSA

KPVVIDHENDVIGGITDLNIKCPITGSTLLTLEDLYNNVYLAIYMMPKSLHNHVHNLTNLLNVPAEWELKFRKEMGFNLS

EDIIPKQKMFQDSGLFSIDGVLNLKALSDYYVENITNIGLMRTEIENREDFLQPCHKIPTLKSSKKCSQSNIISSKDIIE

NLEAYRLGEESSNSSTSVAVVKGLLRTYYEDKNRLLEFFDDNLVDSFSLLNKVKEIAASKEISIGKSMTSKFIRNNHPFT

VETYLKTKLHYKNTITVLKSKKVSEELYDLVKQFHNITEVDMDSIMNLGKGMEGKKHTFLQMLEFVLTKAKNATDSIDFL

VSVFEKMQRTKTDREIYLMSMKVKMMLYFIEHTFKHVAQSDPSEAISVSGDNKIRALSMLSLDTITSYNDILKTSSSKSR

LAFLSADQSKWSASDLTYKYILAILMNPVLTCGEATLMTECLMMYVKLKKVCIPTDIFLNLQKSQSVFGQNETAIGLLTK

GLKTNVYPVSMNWLQGNLNYLSSVYHSCAMNAYHKSLKSYKECQFQTRWIVHSDDNATSIIAHGEVDKMLECFNSQSLPE

MLFRSIEEHFKSFCITLNPKKSYASSSEVEFISERIVNGAIIPLYCRHLANCCTESSHISYFDDLMSLSIHITMLLRKGC

PNEVIPFAYGAVQTQSLSLYSMLPGEVNDSLTMFSNLNVSLKPNEIPISIGGWLTAPIEALSILGPSSNDQLVYYGVIKE

FLRGGEFGELKDKITTNLYLRARFDELEKRVKNDTLEDRDKKMIFLTNLFEKSSISEDSDVLTVGMKFQTMLTQIIKLPQ

FINENALMKMSSYKDFCLIYPNLKKNENLKSVFEKVRCDEDSLLLDEAYVQSYASRQEMEQVHQIMIENPETILIAPLND

RDFLLGQLFMYTSPSKRNQLSSQSTEKLALDRILRSKARTFVSFDSMQKMTYEENMISKIKEMSVFSSESYSSFKTCIQL

VVKDVNFSMLFPVLDSLHPCESRRRDNYNFRWFQTEKWIPVIEGSPGLVIMFSVYGANYIESLGLKNIPLTDDSISILTS

TFGDNLVLDDVKTFISCKEVFETSQFQNADHCQRLVRTCNYMIAAQNKLLAINTCFSRKNFPFYSKFNLGKGFVSNTLAL

LSTIYSKDDSYHFISNASYKLDRTVRAIVSAQQDLNLEKILDTAVYISDKLQALFPSIKMETMREILKNVCLDSAPIWDS

IEKKMTRINSSTAGRFTVSNVILSHNSELNTIQKQIVWLYNMGLCSEKTLEFVIRYIRRSDVRYVRTEEQDEMGNYVSGT

VYKTGIMTQNCYVQLMASDQDISVSLKTPFSILNERNHLYDTYKESIEKLLHKYMLDKSNIVKSKLPQTTYLEPGQACLR

MTSDNKMIVKVNATPRQIKMENVKMVINIKYENVDSDLWEIIENQKTVTLRKPDPGECFSEMYKTLDSESRMISAIKEQL

AKSLTFLNTFENLSNQIDDVEEEDIRETLQDLLQQLQESCLEGLGQCKSVEEYSMFLDSNGFSQTIELFKGILDSYDSFE

SEYSSLFSSIIDKTTRFTNDLNGFKDTLMMLKYSMINDASGFKSYRAVGNFAVNLATKKHIEIDGLNVLGLIQLIKACET

CHNNDSILNLASLRNVLSKTYASVGTRMRFYHEFDLQNNVMERSYDFKTLVLPNIELSSLSRDILKDNGFVISGENLKFD

TDELGFEGLANLDMLRLDEEEMYEGIIRDMKIKRKKKGYLFPANTLLLSELIKFLITGIKGTSYDVETLLLNSFKMSYHT

IDRLGRLSSSVPALKAYSIVYYEYLSSDNPLNEIAECLEGFLRLSKCKPKGQALDGKMKRVLVQLRDEQDRSKKLEVYKA

IASFLSKNPLCLSERTLYASYTYHEINQYIMKTREIIIEKISELEDNTGDEDFQILSKYLNEEEIMTDDEELGSCSY

>Impatiens_necrotic_spot_orthotospovirus_(from_Impatiens_sp.)_(AWK77935)

MNNYKARLLIENSVTLLSSIDDCIKSNLELSRDLHKKNPDEISEDIIINNNAKNYEALRTLIARITRDGEGIETGLATVD

MKKISEDMTLLEQKYLETELARHDMFGELVSRHLHLKPKKRHDVEIEHAVREYFEELSKKECSNRLSEEEFKKVSKEYVA

TNATPDNFVIYKESKGGPLCMMIYDWKISVDAKTETKTTEKYYKNIWKSLKDVKVKGKSFLEEHPIFISIVILKPIGSMP

IVVTTSRVLEKFEDSKSALHANRLRHASQSKLVGVSNIGQIIGTTPTVVREFYADTQKLKIEFRSILGEEFGSKDIFFSH

WTNKYKDRDPTQIAHSEDLEKIIESMVTDDISREEIVHFMFGNFCLHIETMNDQHIADRFNGYRSSCVNLNVEPKKDISE

LKDHLLSTKGLWESLYDHHLIKVMDRIKTKKQKEKIIPDIMTAFKLNAEEYEKKYPNCFTSDLSETKTNFSVTWSPCTDM

VELGNQDYNNAVIDRFRKAFLSNPRIRYNSAYSKEMNVTSKAKDVTELVRSCLTTLSCDTSGMDSQQLEDEIDISTGGIK

VERTSKSQEWIKKNDCLTRNRNEFNMRETSKDNKVIYFKGLSVMNVSMSKKKRIIKHEELKGKITKGLEYDTSERQYDPN

DDYVSLDLSSFTHAKKLIRHDNEESLEWCSQIQDGLFVLHNSDIRENCKVATVYKNYTKNPENLFTQSTIIKTEMETCKK

INKLCNDLAIYNYAEDMMQISKGLMVADRYMTKESFKILTTSNTSMLVLAFKGDGLNTGGSGVPYILVHMVEETLSEQFS

VCYTKEIYSHFSFGSHVVYIMRPQRLNQVRLLSLFKSPSKVPVCFAQFSKKAHELEGWLKIKDMQEVQTLSMSSNVRRIM

RNIVFSSVMIGTVTKLSRMGIFDFMRYAGFLPLSDYSNIKEYIQDKFDPDITNVADMFFVEGIKKLLLKMENLNLSTSAK

PVVIDHENDVIGGITNLNIKCPITGATLKTLEDLYNNVYLAIYMMPKSLHNHFHNLTSLLNVPAEWELKFRKEMGFTLFE

DIYPKQKMFQDNELFSINGVLNLKSLSDYYASTVENVGLMRTEIENKEDFLSPCYKISTLKSSKKCSQSNIICSDDIINC

LQEVNVRSLEDLSPKNLAILKGLLRTLHEDKNRLYEFFEDHSENPYYLMEKMKTIKSSEKITTGKSKTSKFIRNNHPLTV

ETYLKTKLYFRNNITVLKSKKVSEELYDLIKQYHNIMDIDMESIMNLGKGLEGKKHTFLQMLEFVMSKAKNASDAIDFLV

SVFEKMQRTKTDREIYLMSMKVKMMLYFVEHTFKHVAQSDPSEAISISGDNKIRALSMLSMDTITSYNDILKNSKNKSKL

AFLSADQSKWSASDLTYKYVLAIIMNPILTSGECTLMVDCLMMYVKLKKVCIPTDIFLGLRNSQEKFGTNETAIGLLTKG

LSTNSYPVSMNWLQGNLNYLSSVYHSCAMKAYHRMLESYKKCEFQTRWIVHSDDNATSLIADGDIDQMLTDFSSKSLSEM

VFRSIESHFKSFCITLNPKKSYASSSEVEFISERIVNGAIIPLYCRHLANCCTESSHISYFDDLMSLSINVTMLLRKGCP

NEVIPFSYGAVQTQALSIYSMLPGEINDTMRICKKAGVNLEHNEIPTNLGGWLTANVESLSLLGPSSNDQTIYYNIIRDF

LRKDDFEQVKQSTSSERFLDLRFEELKQKKERDKLELNDKKMIFLMNLFEQSSVSEDSDVLNIGMKFQTMMTQIIRLPQF

INENALSKMSSYNDFCKLYPHLRKNQQLHNSTKEIKYDEDMLIEDLDDYEKMAPANEMEEVHEIMIKNPETILIAPLNDR

DFLLSQLFLYTSPAKRNQLSSQSTEKLALDRILRSKARTFINPDSDTKMTYDENLEKKISTMKTLNEDSVSVFKTCINLV

LKDVNFAMAIPIIDNIYPCEARRRDNYNFRWFQTEKWIPVVEGSPGLVVMYSIYGSDYIEKLGLKNIPLTDNSMVVLTST

FGSSLMLEDVKYYVKGLEVLETGEFQNSNSCQRAVKACNYMITAQNRLLAINTCFSRKNFPFYSKFNLGRGFVSNTLALM

SSIYSKEESFHFMSNVHFKIDKSIRAIISAQQDLNLERILDTAVYISDKIQAIFPDITRFDIMTILKNVCIDSVSVWDTL

ESKMDKINHAMERKMTTSNILLSHNSELNTIQKQIIWLYNMGLCSKKTLNFVIRYIRRSDVRYVRTEEQDSFGNYVSGTV

YKIGIMNQNNYVQLMASETDIAISLRTPYDIRDERDVLYSAHKDSIEKLLSKFLFDKGNVIRSKQSQTVFLNPGQACLRT

TTDGKLIAKVNSTPKLLKVDNVKLIMDINYENVNSDVWSIIESQKQIDLRLPETGECYSEMYKTIDSEQGLIYEMKSNLI

KSLTFINTFADLNESVYSIDDEVTRETIFDFIDSIRNDCLEGLETCKSVEEYEEFLDTHGFRETVSLFKNMIESLESLDA

EYSPIFLNITDKYQKFSEDLGNFKSMLLMLKYSLVNDASGFKSYRATGAHAIDLTMKKHIEIGEFNLLGLIQLIKACESC

HNSDSILNLVSLRNVLSKTYTISSRKIQLYYDINLQNDLMERSYDFKTLVLPDINLSDYSKEILKENGFVVSGENIKIDR

EIGDEDFVGLASFDVMRLDEEQMYDEIVKDMKIKRKKKGYLFPSNTLILSEMIKFLINGIKGTSFDVESLLRNSFNVTIF

AGSRLGKVSTSVPSLKIYSTVFMEYEKSDCPLNEISECLEGFLKITKSEISEPILEGKLKKVLIQLRNEKNKSKKLEVFR

AIYGFLSNNPLCLTDKTLYGRMTFEDINRYIMETREIIINKIKELDDGDSSDSIEVLLKYLNEAN

>Pepper_chlorotic_spot_virus_(from_Capsicum_annuum)_(AQX77525)

MNIQTINCFLDSTGEINVYLQNIKDHLMIMSNIVEHKFKNGSSIIDSGLNSIMSMNCSLDKIESMKIHYYQNSSTIDKKE

MESEIFLFLDKYKELELMRHDLFGVIASTRLHFAPKYRTDVFLKECLVDYIEFCGASTAIMNKIDKPQDLMSSLVFQHLT

PDNYVIYKEGKGQKACLMIYDWKVSVDTVTESKTSENYYTSVWKTFKDVVIEGEPFLERHPIFITIVILNPVGKMGITAT

TCRVLQEFKNSPYRTFVDRQRAAHQAKLINVVNLRQILVHDADIFCRFYAETQTFKSSLLGKVGDYMNRANEVFFSHWSY

EYKKTCLADNAMAIDIIELINSMSTDKIGRELLVHFLFGSFLYFKDTMSDIHIKDRFLGYQRCCEMMGIEPAGNEIELKD

YLDSNEQLFEDLYKDHAEKIRRDVLMKKSKEIEIKSIEEAFNINADEYQKEYPNCFTNNLQETKTNFSVCWSPMVEDLDM

NGLNYNNAIIESFRNSFSEATNIIYNKPYGGPTVEKGFCNTLFDLVKTCIVDLSCNTTGYTKEKMVDVVDVKDGSIQIDR

TDQAKSWIEIGNIKTRNNNEFTLSQKTTSDSRRCFMKGLNLMHIDMGKKKKSEARDDLKSKLQDAKMKSVEGGEYDPSKI

EEDQNIDLKKVSHKKKLIRHDNADVDYFCQSMIQSMYVLHALDTRKDKEGKIDKVYNEYCNKPESVFTKHNLIGTEMETC

KNINAVAKELSIYSYSEDMMQISKGLMIADRFMKKTDFKILTCANTSMICLAFKGDGINTGKSGVPYITVHRVDNDAQPH

FVSLYTKELVISFRSGNYHINIMRPQRLNQVRLLSLFKSPSKVPILFSQYCLLSKELKNWLTKPKVTIFSCPNNRIFNLQ

QILFSSIIIGTVTKLSRMGIFDFMRYAGFLPLSDYSNIKEYIAEKFDPDITNVIDCYFVSGIKNLLLKMEGINLSSSVKP

LTIDQENDMSGGISDLDISCPITGSTLRTIECLYNNVYLAIYMMPKSLHTHIHNLTNLLSVPADWELKFREKMGFKLNEE

IYPKKEMFNDSGPFSIDGVLNVKTLFDYYQKTIDNVGACRSNIEDKEDFLSAPYKIQTLTSSKKCSKSDIIKNIEIKDAL

KNSLNKDPESIKGNDLYILKGVLKCFQEDRETLNSFLEAMKLDQAEYYSYFSKLLSGDSKMIMKTNKEKFYHNSHPLTVE

SYMKVRYGFFNSTTVLKSKKVSEELYDLIKEFNKITELDLESLENMGRGLTGNRVTFLQLLEFVMMKTKSNAGNTDFLVS

VFEKMQRTKMDREIYLMSMKTKMMLYFIEHTYKHIAQSDPSEAISVSGDYKIKNLASLSYDTITNYNTALQKHECKMAFL

SADQSKWSASDLTYKYILAVIMNPILTTGEINLMCECILMYVKLKRVCIPTDVFLNLKRGQTEYGSYGTSLSTLTENLET

NTFPVSMNWLQGNLNYLSSVYHSCAMMGYEKAMKTNKDLDFTIRWMVHSDDNATSMVVKGDIKKLFKEFNCQSLSELLFR

SIQSHFKSYCITLNPKKSYASESEVEFISERIINGAVIPLYCRHLANCSTESSHTSYFDDLMSLSIHMTMLLRKGCPNEL

IPVAIAAIQVQALSIYSMLPGEENDIMTIIKDSDMPLEKNEIPTCAGGWLTAPVEVMSILGPSANDQLIYYKILLDFFKV

KDFNMLKKNVSSLGYVELRRCELFKRVKNGTLTTEDKKLICMVNLFKISLMSEDSDSLSVGMKFQTMITQIIKLPSFVSE

SSLQKNSSFKDFCKIFPNLKKNTDILSALKNKNGANEYDIDESSDKSLMSKIQLEELHRHMASHPEAFLIAPMNDKDYIL

TNLYAYSSVSKRNQMSNQSTEKLALDRILRSKARTFIDSDSKEMMTYKENMMLKMELLKKVDGNDFNSIKTISDLMIRDM

NFEMVISLMENTVANASIPKSNYNFRWFITEKVPSIIEGSPGLIVMSAVYGMDYLTNLGLKKLPLTIDSIAILHDIFGYK

GIFDDVKNFIRNEGKDYKTSEFLESDVLKKYVLSINYMIQSQNKLLSINTCFSRKNFPFYSKYNLGRTFITNTLAIWSTI

YSRLTNINFYTNLNFVIDKSSRIIVSLQRDVNLEKMIDCCSYVSDRLQGLFPDMTKNSIREILNRLNFNGIDLMKKLISE

LKEVKRAINNVRTSNHVTLSFRPQMIAMSKHAAWLYNFELINEKEFKFVIDQIRQSEVHYIKTDEQDVKGYYVAGQSYKI

GIKTQHNYSQLSLSNQDVSVFLHSPYEFKREDDGKIWEVHVKSVYKLLQKLLYDKSSMIKTFLSIRAELLPGEFCIHESS

NKTMLILINDTTRSLNIERMKLKGTIKYTPSSEYAWETMTNHSNYKLRLATVGECYSDLYKSVDDQGNVIKNVLNNLKTS

LEYGEEMGNIVEQSLAEIDDEETVEMVRDSVQQIHDVAYRGLELCETSEEFEEYLITEDFQDMVDLQRDLLESIVDEKYK

IGAIVEGTARKLSRWTNSLSNFKNVCTMLKFSMVNDSKGIRTYKATGLDFNSLSSSEIMTSQGFDLFELLKLIKACEACH

SSNSVLNLFSFRNIKNTKYIPGYRRFLPNKVHFDYDIVLNNDVMKRFYEYKTISLMDLKLSRAAKEVLMENGFSITGETM

KLDEEIIEPSRAQLQDSMSAYDQVAMQMKLTKKKSAYLIPSNTLLLGELLKFLMLCVKGEEYDVMKLLRNHFRVEAPRDD

RLTSIQDAITSCKISGFIQKHFMNDKKEIVLLGIAESLENFMFLASPDQYGEEQINAEELVKKALAVNEQRFKHKILTKM

RKYITTDVDFLTERCLYGNMSSEEINLLIFNAVSKIDLELSVLNPKKKKKRAKMLEDLRGFLDSDTPQGSQEG

>Polygonum_ringspot_virus_(from_Polygonum_sp.)_(YP009293585)

MNLQNVHSFLEITGQITVFLNDLREKLMVLSNVIQKKYKDGSDPISSGLKAIMSMSCTIDEITDMKATYYQSTMSIDQKE

MEKKIFIFIDRYKELELMRHDLFGVLASSKLHFAPKHRHDVVMKDCILSYLEYCSEKEDISNRIEDLDGLTSQLVFQHQT

PDNYVIYKETTGEKACLMIYDWKVSVDTMTENKTSENYYTSIWKTFKDVKVGGEPFLSKFPIFVTIVVLQPMNFMPIVAT

TCRVLEEMRNSPYRTFVDRRNAASRAKLISSRNLKDLAGQDGSKFISFYSECQAFKNLLMTNIGDYMNRTDEVFFSHWSH

EYKETSLKDNLMSRDVVEIINSLPNDTIKKELIVNFIFGNYVFHSKTMSDLHRKDKFQGYRSNCKLLKIKPKKTEEDLKV

YLENNEALFESLYSKHMDKIKTDMLLKKSQEKEIKSIEDSFNINAENYQAEYPGCFTNDLQETKTNFSVCWSPSVEKLSV

KEMNFNNSIVENFRQCFDGEETLIHNKSYGGKFSQGEFSNTLFRLVKACLKDLSCDTTGQNKIVAEDVVDIKDGSIKISR

ESKEKSWKEIGDIKTRNGNEFTISERTSKETRSSFFKGLTLMNIDMGKKRKNDHKLELIEKLKQSRVVNEELSLKEGEYD

VSSSPEVVNIAVGSVSHNKKLIRHDNPDVQYWCDSMIQSMYALHGFDIREKDSGKINAVYSEYCNEPEKFFSKGKLIESE

INLSKNLHKVSQSLAVYSYSEDMMQLAKGLMVADRFMRKTDFKILTCANTSMVCLAFKGDGLNTGKSGVPYITVHKVSEL

LQPYFVSLYTKELVVSFKSGDFYINIMRPQRLNQVRLLSLFKAPSKVPVCFSQYCLLSTEVKRWISRKDIDLLDCPSNLL

PFLKNILFSSVVIGTVTKLSRMGIFDFMRYAGFLPLSDYSNIKEYIAEKFDPDITNVVDCYFVTGIKNLLLKMEGINLSN

SIKPLTIDQENDMSGGINDLNIKCPITGSTLKTIECLYNNVYLAIYMMPKSLHTHIHNLTSLLNVPAEWEVKFRTKMGFD

LNEEIVPKKEMFNDSGPFSIDGALNVKTLFDYYLKTVDNVGSTRSNIESKEEFLSTPYKIKTLTSSKKCSKAEIIKNSEI

KQCLSNCLGRDPETISGKDEYVLKGVLKCFVEDKDALRNFMTLEEMDESDYFHFFTRLTTGENKAVMKTSFDKFYYNSHP

TTVETFIKVRYGHVSTTTVLKSKKVSEELYDLIKEYNKIIELDLEALENLGRGLSGSKMTFMQLLEFVLMRTRTNAGNTD

FLVSVFEKMQRTKMDREIYLMSMRIKMMLYFIEHTFKHIAQSDPSEAISISGDYKIKTLASLSYDTITNYNTSLQKGLEC

KMAFLSADQSKWSASDLTYKYILAVLMNPVLTTGEINMMCECILMYVKLKRVCIPTDVFLNLKRGQTTYGSHGTAISALT

ENLETNTFPVSMNWLQGNLNYLSSVYHSCAMLGFEKALKTNSDFEFTVRWMVHSDDNATSVVVKGDMKKFLSKFNCGNLS

EFLFRSIQSHFKSYCITLNPKKSYASESEVEFISERIINGAVIPLYCRHLANCCTESSHNSYFDDLMSLSIHITMLLRKG

CPNELITFAYSAIQCQALSIYSMLPGEENDIIAIGKEVGFPLAKEEIPTCAGGWMRAPVEMLSILGPSSNDQYIYYKILI

EFFKQKDFTSLKTQVNSLGYVSQRINDLNKRIAKDTLTKEDIKMICMVNLFKTSLISEDSDSLSIGMKFQTMMTQIIKLP

SYVSEGSLMKNSSFQDFCKLFPNLKKNSDLLDSLKPIQIDEDGAGNFSDDNSMISRIQMEELYKHMSKHPEALLIAPMND

KDYILANLYTYSSISKRNQMSNQSTEKLALDRILRSKAKTFVDPNSKVMMTYRENMSSKLKEIMDKSSNDYKIITTISDM

MVKDMNFEMIISLMENSVANASIPKANYNFRWFVTEKVPSAIEGSPGLIVMSAIYGMEYLVELGLKRLPLTENSICILHD

IFGNRKTFDDVKKHLTSNETDMKTDEFMLADNLKRTVLSLNYMIQSQNKLLSLNTCFSRKNFPFYSKYNLGKTFITNTLA

LWSTIYSRTTNINFYTNLNFIIDRNSRMIVSLQRDMSLEKLLDCCSYVSDRLQSLFPDMKIEDIRSILSKLNYNSVNLLQ

KVTSELKSVKRAISQIKTASHVTLSYRPQLMAMSRYAAWLYNFGYINEREFKFVIEQVRQSEVNYIKTDEQDTRGYYVSG

ISYKIGIKTLHNYAQLEMTNRDIAIQLNSPYEFIRGEDNRMWDTHVKSVYKLLQKLLVDKQSVLKTFLNMKVDVMPNEFC

IHESSQKTLLIYINETNRVVTLDRVKFKGKVKYNYSNEFTWSLMENNYNYMLRKAETGECFSELYKTIDSNSSLMENILA

NLKSSLIYNSDMENMVQNAVEGIDDEENKDIFINSLNQIYDLAYKGLKECKSSEEFETYLRDNEFDDLVNTHKEMLELIC

QELSDTDTRIQSVVDKLRVWSNSLSNFGDLCVMLKFSMINDSKGIRTYKATGVDFHSLSASESMICSDYDIFEMLKLIKA

CEACHTSNSTLNLIAFRDIKNTRYIPPHKRLYGGVVHFSYPLKLNNDVMSRFYEHKTISLNDIEITESVREILKQNGFTI

TGSNVKLEPDMLELNPITVTDEHSTFDSISRQMRLTKKKSSYLIPANTLLLGELMKFLMLCIKGNEYDVQKMLSAHFDFS

IERDDRIDSLIKCIMTLKSSGYVQKYFSNDKEEEILLGVATSLENFMILSTPHGKFVEPFDAQKLIKSALAEKDKDMKLK

VLLRIRSYISSKLDYLNEQCIYKNTTNIEISDLVFSSINHIDKEISELRTFDKTVYHDEFNEYLKTAEGTSSD

>Peanut_bud_necrosis_virus_(from_Arachis_hypogaea)_(NP619688)

MNIQTINNFLDTSGEITVYLRSIKDEIMSMSNIVERGYPEGKGVIESGLNSIMSMNYVLDRIEDMKSRYHQSSSSIDQKE

MEKEIYLFLDKYKELELMRHDLFGVLAGRRLHFAPKHRSDVFLKDCLMSYIEFCNTSTNIINQIVDIDGLKEKLVFQHLT

PDNYTIYKETKGEKACLMIYDWKVSVDAVSEAKTSENYYTSVWKTFKDITIDGQPFLERHPIFITIVVLNPSGNMPIVAT

TSRVIQEFRNSPYKTFNDRKKAASAAKLISVNSLGQLIGLGADCFRRFYSETQTFKNSLLTKVGEYMNRSSEVFFSHWSY

EYRKTNLSNNQISQDIIDLIDAMPNNTVNKETLVHFLFGNYLYFKETMSDLHLKDRFEGYKAHCEMMKVKPIKTEEKLKE

YLEENELIFENLYEDHLEKIKSDVLYRKAKEVEIESIERAFNINAEEYQKEYPGCFTNDLQETKTNFSICWSPMTEELEM

NERNYNNAIIQSFRDAFDEQPKLIHHKSYGGSKMDIHFSMTLFNLVRTCLVDLSTDTTGHSKATLEDVVDVKDGSIQVKR

TETSQKWKDIGKVKTRNGNEFTLSPSADNEARKHFFKGLSLMYIDMGKKKKIDIKNELKSKIQESIAKQKDEDSNGPSGE

YDVSKANIPFQTSIKGVTHNKKLIRHDNPDVKYHCESMIEAMYVLYGMDERKNKPSKISSVYDEYCSNPASLFAKGNLIE

SEMTIAKNIHEVAKELSVYTYSEDMMQMAKGLMVADRFMKKTDFKILTCANTSMICLAFKGDGINTGKSGVPYITLHKVE

KGIQPFFASLYTKELITSFKAGNYFINIMRPQRLNQVRLLSLFKAPSKVPILFSQYSLLSTEIKKWLNQSSVDVFTCPEN

KIQYLQKILFSSVIIGTVTKLSRMGIFDFMRYAGFLPLSDYSNIKEYIAEKFDPDITNVIDCFFVSGIKNLLLKMEGINL

SNSIKPLTIDQENDMSGGISDLDIVCPITGSTLKTIECLYNNVYLAIYMMPKSLHTHVHNLTTLLSVPAEWEIKFREKMG

FMIGDEIKPKKEMFNDSGPFSINGVLNVKALFDYYRKNIINVGACRSNIEDKEDFLSAPYKIKTLTSSKKCSKADIIKSS

EIISALKSTFGKHPDDIKGSDLYILKGVLKCFVEDRESLLNFMEMENLEESKYLHFFSNMMSGSNKVLMKTNQDKFYYKS

HPLTVETFMKVRYGYFKTTPVLKSKKVSEELYDLIKEFNKITEIDLESLEKLGRGLKGNRVTFIQLLEFILMKSRTNAGN

TDFLVSVFEKMQRTKMDREIYLMSMKTKMMLYFIEHTYKHVAQSDPSEAISISGDYKIKNLASLSYDTITNYNTALQKNL

ECKMAFLSADQSKWSASDLTYKYILAVMMNPILTTGEINLMCECIMMYVKLKRVCIPTDIFLNLKRRQTEYGSYGTALSV

LADNLETNTFPVSMNWLQGNLNYLSSVYHSCAMMGYVKAMQKMKDYDFTIRWMVHSDDNATSIVVRGDIKKLLSSFNCSS

LSELLFRSIQSHFKSYCITLNPKKSYASESEVEFISERIINGAVIPLYCRHLANCSTESSHNSYFDDLMSLSIHITMLLR

KGCPNELIPFAYAAIQIQSLSIYSMLPGEENDITSIVKDINFPLKKREIPICAGGWMHAPIELLSILGPSSNDQLIYYKI

ILDFFNVKDFSSLKKNVNSLGYIDLRINELFKRIKYQNIIEADKKMICMVNLFKTSLMSEDCDSLNVGMKFQSMITQIIK

LPSFVSEGSLLKNSSFQDFCKLFPNLRKNTDIIDALKPVSLDERDLGEINESKFLSQIQLEELSRHMSSHPESFLIAPMN

DKDYILTNLYAYSSVSKRNQMSSQSTEKLALDRILRSKAKTFLDPNSKIMVSYKDNMSMKMKEIMSSEGNDFKIINTISG

LMVRDMNFEMVVSLMENTVANASIPKANYNFRWFITEKVPSVIEGSPGLIVMSAVYGMDYLIELGLKKLPLTETSISILH

DIFGNRKTFDDVKECIQNVSKQYKTEEFQNADILKRYVLSINYMIQSQNKLLSINTCFSRKNFPFYSKYNLGKTFITNTL

AIWSTIYSRLTNINFYTNLNFVIDRSSRLIISLQRDMNLEKSIDCCAYVSDRLQSLFPAMTIESIKQVLCRLNFNGVDLM

KKMKSEISEVKRAINNIKTSTHVTLSFRPQMIAMSKHAAWLYNFDYINEKEFKFVIDQIRQSEVHYIKTDEQDVRGYYVS

GHAYKIGIKTQYNYGQLAMSNQDISIHLHSPYEYQREDDGKIWEVHVRSAYKLLQKLLLDKQSAIKTFLNVRADLMPNQF

CIHESSNKTLLILINDTTRPITLEKIKFKGDIKYIPSSDFAWNLMNNQSKYRLRKAETGECYTELYKMIDNDDSLMDTIL

ANLKKSLEYGNEMENLIEQSIQGIDDSDAIECIKDSVDQIHKLAVEGLETCKTSEEFENYLKRVDFQSTVDFHKGLLDQI

VEEKYKSSDRVDEAVEKLTRWTNSLSTFKDVCTMLKFSMINDSKGIKTYKANGADFHSLSASEVMTSQGFDLFELLKLIK

ACEACHTSNSVLNLIAFQNIKNKTFIPGFKRSLPNTVHFDYEMKLNNEVMSNFMNINISLQDIKISERVIQVLEAHGFSV

TGQNLKLDEVNLELNPVEIIDDSSTYDQIARQMRLTKKKSSYLIPANTLLLGELMKFLMLCIKGEEHDIMKLLRSHFAMR

PVRENRLLCVQDAVMTCRISGFIQRHFLNDKKEIVLLGVADSLENFISLATPNYAVQEPFKAEALIKKALLEVYDSGKHK

ILKNIRSYIVADVEFLTEKCLYSNITNEEISSMIFNAVSKIDLELANLKPKKRKKRLDLHDVFNKFLGSGATSSEQE

>Nome_phantom_Virus_(from_Chaoborus_cf._flavicans)_(Phasmaviridae)_(AIA24562)

VLLIDEELPTDIANKTPDILMYMEAIETIFLGDLTVTNNVANARIEKSKKYSAIKEYLINKGYRVEHQDV

IIHSSLHNVYSEFRKLSTIGLIHENIDLLHTYIEYHGIAMESMTNAQNNCVDLQLFNVYLNKFDKVTYEA

YDLELDNDEFVKSLPYDDYVPHKTEDELLAEIKNDFDNMNFDAMFEDGIEKTKLAFEKLIKNNVENFEHI

EPKSVFQVVYNVSETEKLTNHNLIMDYVKDIINSDDSEIKSYLLDILPKYSQIGVMKTTYQNKVTDSALK

LEIDIYKSHGVYGPYQYDMATTLNSTITANTKEKLEYGKKLPNLKKSPAXXLLKDIWLTESKTEIHETKE

WRDCYDYVRKSNALQLCSSMSALYDRITHLTTFKALKSNVFVPANGAFIAIMPKNHGPVTNSDANIPLIT

LTRFTNGHFETLCSKYDISRSMDIEYIHKTDNYTYVTSKLCRINLNKMSIWDGVPFKVCATACHLMSTSK

NLRLGKNRTVXILKTRFDAWLIYQVRDFIIKLSDIXXXXXXXFLVPNTRHESAVLFIEEICAVNTIRGKK

LYGNQFMHASAHKCAEWNKEMEDETLVHGTWITEGYNNEKPFPYDSTFAFSSDFIHFAETHTSRKLGLSR

DKIMNDISKSVLSGYVHENTSLRGCTKEPSDMVSETDYHTTSIEAALNYYASVEFDEVKSKVIPCALYFL

KKARSDPASIKFLFYLSLKDQRGPPRPIATPTLAAKIALMLLEKPSQCKGKYVRNNIIVPGVNKLQKQTQ

IYKDTLAEGAARKYKFYSQNSEDQTKYSEGDNLNKYDCYIRSNMTITNTLKKLQLEVLRMLKDRVHIYPX

LPLSVMDDPDLRRYTLGDSKSMSVFAGWPQGMLNYISTDIHCAVSTYITDIYNDIYPEHSVIAEDLAHSD

DSYIVVCTKTKDDFKRFIAFRTMMKRRACLKLNIKKVYGSAITGEIVSNWNINGTVHQSIVKTVANATGN

LSFQNWVIDVQSQXSQLQQLARIGAPLGTLILLHTILYQQMIKVYNVRGEHLKIISMLPVDIGGYPTISA

FELGLCGLSAHYKNILEKCQQEGNEKALKMVLTCLAWSMRSINTGDNDKYISDTVLAAKVEEIMRGKPGI

AFSSEDYMNMQMPNKGTLFLGIKHLMPQTRKITRTMAAINSLNYVSDGMESLITRPTSLRQALGHLKAKS

QSLVYELAAERYSQSARRLAISQSLQASGKVVRLGDYQPMTFNDFHDLLLTIYSFKKCDMEMLRNYLDDE

SELVTLXNLVVNNSVIEEINKSDRKIINTMPEVYNKYETIGHLQDVLLFIVDEDNRTHNITTNFYKTFAK

KEMDRGSALDDAKKIKKRFKNEFRFYEVKSACKLVLQGYLQVPFKKLWIQASCREDNIENFVCDLYGNML

SDKKAYSVKIRYKSQPKNKKDKDIVNSLYSVALLNRMYDGRFQVDRINNIPLFDAIRDVDFSGLDTNEEA

KFAVLSYIYNDDTRYL

>Shuangao_Insect_Virus_2_(from_Abraxas_tenuisuffusa)_(Phasmaviridae)_(AJG39250)

MTKTDIKDTKVSKFIDMVNSHADFKKDKIDKAPASIKNKSPDILTYRSDLQCIIVGDVTVSESTYLAIQL

KRGKYQPIIEHLSKHVRVVEYYLAIDDDLNNIEVVLKGLEDIQLCKNDDMVKSRCMTLHKDINTSMKLGI

TLMDEKNRDMFKLKLSNANKQCEGIDYSEFTRGLNPMPLYIPKKPIEYYVEQIKKKTDETFNDMFDDDYK

PAEDAFIELEENLLKQDKSTFLQNAKSPLQICENSSTLEEKTSHDLVNEYTKDLLLSTALGLNKTQEEMD

VLYYILNLLPNGKQLDLMKNYNDENKDANTEHKVYGLWQYTMNQGYDSALFDWFKTKVKKGRKDPNIKKQ

PQRVHPDQYTRILDQMEKYIDYLGEPSEKPNFMKDAKWEAKTTAEINGSIEIKKVYDYVTGTNGVQLATA

MNQAYQRITHLSANKSRRANLFVPPNASFIMVIPKNHAPLTQSNVDIPFITITRRPKSMTSEPLSYHASY

ETYDYIYYVSKLSRINADKMDSWDQSQYKIIMTATYLGSKMNLDHERLRRLVGIITMYSLDTHQKTSELL

DLFKYVVYMPLSSLSMLSKLVKDKFDIMLKTHLDVWTLKETEAFMKKLSTYRNDPKKPKLKVHQGRVLNQ

SYGLEFKLPSFSVKGYNHSKVEDYIDETSAFFTLRGKKFGGNQFMQRSIASVAEYDMAWEDEQLVYGGWV

TNGYDDIDYPFNSNYAFSSDMIYYATRHKETSNTYNHRNARVNIQKQDLLSPIHYVCSLRGSLKQEKDMK

HDTDYHSTSLHEALKHYEDVNYDNSKCTLMAVGVDAVRHKTARQIESAKEQRGPGRPINTPDLPTKAMYA

LIELPERTITDANPMNVMAQNRNKLKYLETTYKNACEVLVAKNVPFIAQVTEDQTKYSEQDNTEKFRVYI

QSSTIHRHDVRQIQYNALSKFKGRVHIEKNMPEIFDEVKGDTRYINKLRKSNNQIKTREVVLNIGWPQGM

LNYISTNVHEIAFSYVVECYKIAYPFDEIYIFPLVHSDDSWYTIGYVNPVTYKRFCAFLMYYKRMFCLKV

NMKKWWSSPIGGEMVSNLNFNGETIKSLSKTIANCTQNLLFQTYPIDAMSRVSALQQLIREGADMPVLIM

CHTVLRHQLESNYNMINKRTLNIDYSMLPIEMGGYPDISTFELATTGLKGHYFKIWKFVMSNPNSVEAML

ILKLATLSQDKRMKEMMPSDTVTEFEEEAYYQIAAPEKPDVFIALKHKMPKIKKIAQTLKAIDKLPQVDD

GLGLIMGRALSLEQSLGHLKGQTRSQVYALASEHYSSKKRRMAISQTIQSTGKTVILNNMSPMTIDEALL

TLHAMDVHTQSVDHIKMAFHDESEITDIAYRTVHMSSITDSTIPKTKQINRMPQYKNIFQTTTKFQCVLL

SIYDKEKKTQFANSIFPSEDRTHLEIDMDFILKRFSIYFKSYPLTKALSLIHQQFHSQIKTRLFNQPRLR

TDDIVSFITDLYGCTLNRTQNYNVNIKLSEYSVTKESRVLDTLYTAEQLYKIYNYFKPISVNLNTLGCVR

KDIFVRDLDITMLELPQQYKHAILMKLYYNKDQHIEKIDKGGQYRQSWLVRQKYTRERK

>Measles_morbillivirus_(Order:_Mononegavirales)_(BBB34989)

MDSLSVNQILYPEVHLDSPIVTNKIVAILEYARVPHAYSLEDPTLCQNIKHRLKNGFSNQMIINNVEVGN

VIKSKLRSYPAHSHIPYPNCNQDLFNIEDKESTRKIRELLKKGNSLYSKVSDKVFQCLRDTNSRLGLGSE

LREDIKEKIINLGVYMHSSQWFEPFLFWFTVKTEMRSVIKSQTHTCHRRRHTPVFFTGSSVELLISRDLV

AIISKESQHVYYLTFELVLMYCDVIEGRLMTETAMTIDARYAELLGRVRYMWKLIDGFFPALGNPTYQIV

AMLEPLSLAYLQLRDITVELRGAFLNHCFTEIHDVLDQNGFSDEGTYHELIEALDYIFITDDIHLTGEIF

SFFRSFGHPRLEAVTAAENVRKYMNQPKVIVYETLMKGHAIFCGIIINGYRDRHGGSWPPLTLPLHAADT

IRNAQASGEGLTHEQCVDNWKSFAGVRFGCFMPLSLDSDLTMYLKDKALAALQREWDSVYPKEFLRYDPP

KGTGSRRLVDVFLNDSSFDPYDMIMYVVSGAYLHDPEFNLSYSLKEKEIKETGRLFAKMTYKMRACQVIA

ENLISNGIGKYFKDNGMAKDEHDLTKALHTLAVSGVPKDLKESHRGGPVLKTYSRSPVHTSTRNVKAKKG

FVGFPHVIRQNQDTDHPENIETYETVSAFITTDLKKYCLNWRYETISLFAQRLNEIYGLPSFFQWLHKRL

ETSVLYVSDPHCPPDLDAHVPLCKVPNDQIFIKYPMGGIEGYCQKLWTISTIPYLYLAAYESGVRIASLV

QGDNQTIAVTKRVPSTWPYNLKKREAARVTRDYFVILRQRLHDIGHHLKANETIVSSHFFVYSKGIYYDG

LLVSQSLKSIARCVFWSETIVDETRAACSNIATTMAKSIERGYDRYLAYSLNVLKVIQQILISLGFTINS

TMTRDVVIPLLTNNDLLIRMALLPAPIGGMNYLNMSRLFVRNIGDPVTSSIADLKRMILASLMPEETLHQ

VMTQQPGDSSFLDWASDPYSANLVCVQSITRLLKNITARFVLIHSPNPMLKGLFHDDSKEEDERLAAFLM

DRHIIVPRAAHEILDHSVTGARESIAGMLDTTKGLIRASMRKGGLTSRVITRLSNYDYEQFRAGMVLLTG

RKRNVLIDKESCSVQLARALRSHMWARLARGRPIYGLEVPDVLESMRGHLIRRHETCVICECGSVNYGWF

FVPSGCQLDDIDKETSSLRVPYIGSTTDERTDMKLAFVRAPSRSLRSAVRIATVYSWAYGDDDSSWNEAW

LLARQRANVSLEELRVITPISTSTNLAHRLRDRSTQVKYSGTSLVRVARYTTISNDNLSFVISDKKVDTN

FIYQQGMLLGLGVLETLFRLEKDTGSSNTVLHLHVETDCCVIPMIDHPRIPSSRKLELRAELCTNPLIYD

NAPLIDRDATRLYTQSHRRHLVEFVTWSTPQLYHILAKSTALSMIDLVTKFEKDHMNEISALIGDDDINS

FITEFLLIEPRLFTIYLGQCAAINWAFDVHYHRPSGKYQMGELLSSFLSRMSKGVFKVLVNALSHPKIYK

KFWHCGIIEPIHGPSLDAQNLHTTVCNMVYTCYMTYLDLLLNEELEEFTFLLCESDEDVVPDRFDNIQAK

HLCVLADLYCQPGTCPPIRGLRPVEKCAVLTDHIKTEARLSPAGSSWNINPIIVDHYSCSLTYLRRGSIK

QIRLRVDPGFIFDALAEVNVSQPKVGSNNISNMSIKDFRPPHDDVAKLLKDINTSKHNLPISGGSLANYE

IHAFRRIGLNSSACYKAVEISTLIRRCLEPGEDGLFLGEGSGSMLITYKEILKLNKCFYNSGVSANSRSG

QRELAPYPSEVGLVEHRMGVGNIVKVLFNGRPEVTWVGSIDCFNFIVSNIPTSSVGFIHSDIETLPNKDT

IEKLEELAAILSMALLLGKIGSILVIKLMPFSGDFVQGFISYVGSHYREVNLVYPRYSNFISTESYLVLT

DLKANRLMNPEKIKQQIIESSVRTSPGLIGHILSIKQLSCIQAIVGGAVSRGDINPILKKLTPIEQVLIS

CGLAINGPKLCKELIHHDVASGQDGLLNSILILYRELARFKDNQRSQQGMFHAYPVLVSSRQRELVSRIT

RKFWGHILLYSGNRKLINRFIQNLKSGYLVLDLHQNIFVKNLSKSEKQIIMTGGLKREWVFKVTVKETKE

WYKLVGYSALIKD

>Actinidia_chlorotic_ringspot-associated_virus_(from_kiwifruit)_(Fimoviridae)_(ALX00127)

MSESIERIKKAECDKVAQDVKDGKVFDNDVLSRFLSLVGKPRNRYTISSKPKEVEAIYKQCISSEGFMSE

LLSLAERILYSPPSSNQVDFALTIVQLLECSRHDELLFTVVNRLQDFGYNVTATDCQIKDVYPDINSIMT

PDIFYNDGNGKDYVLELKVRNKATDLSKYFDKYRSVLPAFVDISVMNYTTDGFLEFGEFKLSHQLNFIGD

EFRLVDECINLAKSIREKYSQFPQYALFTQFNSESSRDDFMSGFNDLVVNHPSYDEISKLFGSSWSDIID

GLDNYSLIENEELVTDDLISSESDLHGYCNREILNYENHLSYYTFQNSYGRTKLNNKNLDLLIESKNLTK

YRITKSYKPSIYIPITKTIKLDSYGGSRLKFYREAFQNINIIGDRYSKSAYGLINEIFNTISVELLVSKD

GCIDPLLYKDVLDPDFTKFLNDQTTKYRKVAHISNITSDTTILANNSFSINSHVDPFLRKNICDYDKKHY

NPSEKKKECLSYKSSSADLPMLLKLCSEMFHSDHHCGVYLNDLVTLEPMSMHVKGSDIPLESQTKYMEHL

YNIHVIYKNLISLNTINSHKFRLLQTPDPNVILILLPNADALRGNPLRYFCINIMNNDCETEVEANKLLG

IYHSHTTTKKYTIMLSKVISLDVTRLKLLSNSFCKYSLLVSYYSQFKSRLPVNIHTMCWLMTQFITISSL

TITDTYKNLIMAIYSDFSNIDKLIEDKLECRPRTIGQIYILKLMIDGLIKASDQLDQISLNKCKTEVDDS

GELFGTGFDKSLKLRLPLSNLSTHTPKEIIHESFILFYIGNKGLHGSPQELLNLYYTPYQFEDEYKQMIE

NYQTFLIEDGNNSQMSFSYEAMKLTTRYAYAKLMSKSAEIRSNIISNLSLDSPILSIKQFSSTKSMVSNS

TKTEVDRVVNLPSNADLLILERYIDETKIDDIYEFVKEMNAQIDSINSRRVVEVEAQTIDNLSRLNKGKV

MLPHLEIHVKNGVSFIGIARHKYAKAVDGNFIKQSNTKVFDEFYRICDEENIDTLRQFYTKYIDDNELII

RIFYKDQRTADDREIYTGNAQTRLCLFPIEMTFKSICKHIPEEAITISGDQKQKRLLDQRVSLLKTKKYL

DREKKQTEIYSVSSDASKWSARDLFPKYIITLAYNPYLTKNEKYFLLYLMLKYYKKKIVLTDSAFLNILR

FASPDIVGNYEKMTNGYTTNAFEVRSNWLQGNLNMTSSFVHHCSTLLTEMMLTVLSTHHHFNSIMTSMVH

SDDSTYDFLIATDHNTTGDWSNKAQIGRLIVSLITFSNKKHCITLNEKKTYISTFYKEFLSTTIVNNELF

FFYMADLLPIASDTSYTSPLGDFSSYNGYINNSFSHACPLRVIKTAICLINHLTLTTYNMQYTSEKNPKT

MLPDPIDLPIQIYPRYKLNPSLAGSIPYYSSDAFNIVNDIIETLDKSDDLYKSLVEDTINDQVVKSYLKL

IKKDHPRKYKYIQYCMLTMDLNQYERDDQDPYNIIDYDLSQKSLINVVSLNKGSRIRKSHTYQQYLENEK

LVKLTCAVNPMWCVSKPKDNDLIKLSILSNYSNPVFKDSLIFSKPALDYGRRIINSNKNLYTLSSHLFEK

EKPRNLKTIYSHLAEKAQSIEVDEDMLIKYLSIYLFSDKKISASLQIYYSKKSVTYMDKPEFTKVIMPRS

VYGEEYGTHSVNSMFENLLVEPYYNILTIDSKSERFIKTCEYSLQRIPSDIKLYRDPEDIDEDFINYMTF

KYHLAKPEDGLIKICFEIDDDFNMIIYESKLAYQGLLIRYYTDIKKTIEDPSYNIPSYVSPNSLIMTIDS

LMKRSEISTKIYMAHTRANRFDDYWLSRFGMYADDNFYIKYKLGYRIKVATDNLLMPTMKRVRSTSEPVS

FLTKLLCSDPELYDELTQNEEFIVGGYLYRDLLKEIESTTDMNNNLLLYVLGQISMQRMTRVMEENNRVW

NHWILPTGGNIEDPDASIALYNYKSTFMKVETVGINGSVSFTVSVAKSNFLHDDGINIMLKSMCKDYATQ

LRRAIILAPFPGSYRRRPLYINAYGRLATSGDKVKNCIANINIGKIIALKALYCEANQSVSQLMSIESDL

FHHEFLYKFRHYVDDDYYINNLIENIELDSISICQHLISKGFIQNHFEYYKEISPYMGSGHFLELFNTSK

NISCYTSRIDPLRLRKLVHIANFLKNDHKDDIIIKLCDCLKPLCSSSGISITEVLDPEPFIKGLMNYKFD

QAYYKDFYDLYNKIESPPYEAIIQFVSSSTSVNGTLSKIILAIITILKSYPSRYIRQTDDLEI

>Redbud_yellow_ringspot-associated_emaravirus_(from_Cercis_canadensis)_(Fimoviridae)_(AEO95760)

MDKLKKVEYESTVHDLKEGKIFDDDIFSKFMKLVGKTISSYTITSKKKEVEAVYHQCVSSGEFMPELIDI

ATRMIHSPPSKNQIDFALTIVQLLESARHDELGYVVALKLQNCGYNVIATDFKIKDFFPGINSILTPDIL

YQDEHGNKFILELKVRNKLTDLKYYYDRYASVIKSEASVAVFNFIDDKFYEYGDYKLSTQINIIGDEFKA

VTECIQLAKELRAKYSNYPQYILYSTEAQDIIQEDLISGFDEIYKELEGYEEIKGLFGEHWDDIIKNIDE

YNLVDNEEAVHEDLMQTEANLQQYCVKHMADYKQHRYRNYEKVGSYNQTKLLNKNVDELIDRRNEKKYHI

VENYKPSVYIPISKTIKLTSYDGSRLKFYRDAFIDIKISGDTYSRSAYNLIDSIFNTISVELLVTKDSKI

DHKLYKDVLDPEFVKTIFDETPKYRKVAHISNITSDVTILANNSFSINSHTDPMMKQNICGYKNKHYDAD

RKGKDCLLYSKASNDINKFLMCASKVFNSEHHAGVYLNDLVTLESMSIRHKDSDIPIQARTKYLEHLYNC

HVIFKNIISLNTVNSHKFRLLQTPDPNTILVLLPNADALKGNPLRYFCINILDKDCDDDIELNKLLGIYH

SHTTTKKYTIMLSKVISLNVTRMKLLSNSFCKYTLLISYYSQFKKKIPINIQTMCWLLTQFVTISSLTIT

DVYKNIIMAIYSDYSNIDNLIEDKLECRPRTISQIYLMKLMFTGIIKASDQLDKIILSKNEAQVDDSGEL

TMTGFNKSLKLRLPISDISTNNPKEVIHEAFILFYLGNKGLHGSPQELLNLYHTPFQFEKEYQAMIDNYG

CYTYEMGNYSNMSFSYEAMQLTAKYAYAKLLNKTREIRETLKSYLCFDDPILTIKQFSSTKSMVSNSIPK

EVESDIKINHKTDLLGLERFVDGQKIDSPEEFVKEMNVRINKINTEKKAQVDQNVKNNLSKLSKGVTLLP

LLELHVKGDVKFIGFTKTKYAKAVGGDFVKQTSTKVFDEFYRISDEEGISTLRSFYMNYLNKHDLLVRIF

YKDQRTADDREIYTGNAQVRLCLYPIEMVFKSVCKFIPEEAITISGDQKQKRLLDQRLALLKTKKHMDRS

GKKTEIYSVSSDASKWSARDLFPKFILALSVNPFLTKNEKYFVLYLMIKYYDKKIVLTDSAFLNILRFAK

PGNSGPYEAMTKDYTSNNFKVRSNWLQGNLNLTSSFVHHCSTLMTEMMLSVLSEQEGFQAVVTSMVHSDD

STYDFLIATDGNSSTEYISKQDIGKTIISLLSFSNMKHCITLNEKKTYISTFYKEFLSTTIVGNELFFFY

MADILPIASDTSYTSPVGDFSSYNGYINNSFSHACPIDVLKPAICLINHLTLATYNMQYTSEKNPKSLIG

DSIDLPMQIYPRYKLDLSLAGSIPYYSADAFNILHDMLEMLDKANEIKSSLIESIVDEELIQRYLDVIKA

DFPTKYRYLQYCMLTMDYSQYERDDQDPYNIIDYDLSQKSIINVVSINKGARIKKTHTYQKYLEDEKNIK

LTCATYPMWCISKPKDHSLIKLNILSNYSNPNFKDSLIYSKPAIDYGRRIINSNRSLYTLSSHLFEKEKP

KNLKTIYNQLSKKSAEIILTPESVMKYLSIYLFSEKKISAALQIYYSKRTVTYMDRPEFTKVIMPKSVYS

EEYGRNSINSMFEHLLVQKYCDIDDIDPKAERFIKICEYTLERLPSHIKLYYNPEDVDEQFIDYMNFKYS

NNNVEECLISTNDIDSYQMTVYHNKVKFQGLMIRYYTDIKQTLENPSYNIPNYVSPNSIIMTIDSLMKRD

SISTKIYLAHTKANRFDDYWLGRFGMYADNKFFVKYKLGYRIKIATDNMLAPTMTKVKSTYEAVGMLTKL

LCTDQELLSELMSDEEFTIGGFTINELLSEMKMTNDINNNLLLYAFDQIQYPTFLRVLELNNRIWNYWVL

PTDTNINDPDASIALYNYKSAFMRVETVGINNGVSFTITLAKSGYLPEDCVPIMLKQVCKDYASQLRRAI

IMKNVKDSYNQKGFYINSYGRLARPYDKDKHCIGTIKVCNIKSIRPQYTIADGIIRQMVVVDTPLFSNEF

VFKFKHHVDDDYYINNLVDNMELQPSLICTHLIEREYITNNFEYYKEISPYMGSGHYMQLFAINRGFRNY

ADKIDISKFARLLHISNHIKDNHPNDVIVKQSQSLKLTCLQMGLDLNKKTKPDAFINSLMKYKFDQSYYT

DFLDLYSKNESTPYENLIRFIHCTNNQGPLMNKIILAVLTILKCYPSRYIDQDDDVTFD

>Amur_virus_(from_Apodemus_peninsulae)_(AFX97680)

MDKYREIHNKLKGYSPGTLTAVECIDYLDRLYAVRHDIVDQMIKHDWSDNKDSEEPIGKVLLFAGVPSNI

ITALEKKIIPNHPTGKNLKAFFKMTPDNYKITGTTIEFVEVTVTADVDKGIREKRIKYEAGLAYIEQELH

KFYLKGEIPQPYKITLNVVAVRTDGSNITTQWPSRRNDGVVQYMRLVQAEISYVREHLIKPDERAALEAM

FNLKFNISTYKSQPYYIPDYKGIDPVGANIEDLVEYSKEWLSRARNFSFFEVKGTAVFECFNSNEASHCQ

KYPMSRKPRNFLLIQCSLITSYKPATTLSDQIDSRRACAYILNLIPDTPASYLIHDIAYRYINLTREDMI

NYYAPRIQFKQTQNVREPGTFKLTSSMLRAESKAMLDLLNNHKSGEKYGQQIESLNIASHIVQSESVSLI

TKILSDLELNITEPSTQEFLTTKHTYVDTVLDKFFQNETQKYLIDVLKKTTAWHIGHLVRDITESLIAHS

GLKRSKYWSLHSYNNGNVILFILPSKSLEVAGSFIRFITVFRIGPGLVDKENLDTILLDGEYQWGVSKVM

SIDLNRLLALNIAFEKALIATATWFQYYTEDQGQFPLQYAIRSVFANHFLLSVCQKMKLCAIFDNLRYLI

PAVTSLYSGFPSLIEKLFERPFKSALEVYVYYNIKSLLVALAQNNKARFYSKVKLLGLTVDQSTVGASGI

YPSFMSRIVYKHYRSLISEVTTCFFLFEKGLHGNMNEEAKIHLETVEWALKFREKEEKYGESLVENGYMM

WELTENPELAEQQLYCQDAVELAAIELNKVLSTKSSVVANSILNKNWEGPYFSQTRNISLKGMSGQVQED

GHLSSSVTIIEAIRYLSNSRHNPSLLKLYEETREQKAMARIVRKYQRTEADRGFFITTLPTRCRLEIIED

YYDAIAKNITEEYISYGGEKKILAIQGALEKALRWASGESFIELSNHKFIRMKRKLMYVSADATKWSPGD

NSAKFRRFTSMLHNGLPNDKLKNCVIDALKQVYKTDFFMSRKLRNYIDSMETHDPHIKQFLDFFPDGHHG

EVKGNWLQGNLNKCSSLFGVAMSLLFKQIWSHLFPELDCFFEFAHHSDDALFIYGYLEPVDDGTDWFLFV

SQQIQAGHLHWFSVNTEMWKSMFNLHEHILLLGSIKISPKKTTVSPTNAEFLSTFFEGCAVSIPFVKILL

GSLSDLPGLGYFDDLAAAQSRCVKALDLGASPQVAQLAVALCTSKVERLYGTAPGMVNHPAAYLQVKHSD

TPIPLGGNGAMSIMELATAGIGMSDKNLLKRALIGYLHKRQKSMLYILGLFKFLMKLSDETFQHERLGQF

SFIGKVQWKIFTPKSEFEFADMYTSKFLELWSNQHVTYDYIIPKGRDNLLIYLVRKLNDPSIVTAMTMQS

PLQLRFRMQAKQHMKVCRLEGDWVTFREVLAAANSFAESYVPSNQDIDLFQTLTSCTFSKEYAWKDFLNG

IHCDVIPTKQVQRAKVARTFTVREKDQIIQNSIPAVIGYKFAVTVDEMSDVLDTAKFPDSLSVDLKTMKD

GVYRELGLDISLPDVMKRIAPMLYKSSKSRVVIVQGNVEGTAEAICAYWLRSMSLVKTIRVKPHKEVLQA

VSIFNRKEDIGQQKDLAALKLCIEVWRWCKANNAPYREWFHALWFEDKTFSEWLDRFSRVGVPPVDPEIQ

CAALMIADIKGDYSILQLQANRRAYSGKQYDAYCVQTYNEETKLYEGDLRVTFNFGLDCARLEIFWDKKA

YILETSITQKHVLKIMMDEVSKELLRCGMRFNTEQVQGVKHMVLFKTESGFEWGKPNIPCIVYKNCVLRT

SLRTTQAINHKFMISIKDDGLRAIAQHDEDSPRFLLAHAFHTIRDIRYQAVDAVSNVWFIHRGIKLYLNP

IISSGLLENFMKNLPAAIPPAAYSLIMNRAKISVDLFMFNDLLRLINPSNTLDLSGLETTGDGFSTVSSM

SSRLWSEEMSLVDDDEELDDEFTIDLQDVDFENIDIEADIEHFLQDESSYTGDLLISTEETEVKKMRGIV

KILEPVRLIKSWVSRGLSIEKVYSPVNIILMSRYISKTFNLGNKQVSLLDPYDLTELESIVRGWGECVID

QFDTLDKEAQSMVVNKGICPEDVIPDSLFSFRHTMVLLRRLFPQDSISSFY

>Soochong_virus_1_(from_Apodemus_peninsulae)_(AAY56324)

MDKYREIHNKLKGYSPGTLTAVECIDYFDRLYAVRHDIVDQMIKHDWSDNKDSEEPIGKVLLFAGVPSNI

ITALEKKIIPNHPTGKNLKAFFKMTPDNYKITGTTIEFVEVTVTADVDKGIREKRIKYEAGLAYIEQELH

KFYLKGEIPQPYKITFNVVAVRTDGSNITTQWPSRRNDGVVQYMRLVQAEISYVREHLIKPDERAALEAM

FNLKFNISTYKSQPYYIPDYKGIDPIGANIEDLVEYSKEWLSRARNFSFFEVKGTAVFECFNSNEASHCQ

KYPMSRKPRNFLLIQCSLITSYKPATTLSDQINSRRACAYILNLIPDTPASYLIHDIAYRYINLTREDMI

NYYAPRIQFKQTQNIREPGTFKLTSSMLRAESKAMLDLLNNHKSGEKYGQQIESLNIASHIVQSESVSLI

TKILSDLELNITEPSTQEFLTTKHTYVDTVLDKFFQNETQKYLIDILKKTTAWHIGHLVRDITESLIAHS

GLKRSKYWSLHSYNNGNVILFILPSKSLEVAGSFIRFITVFRMGPGLVDKENLDTILLDGEYQWGVSKVM

SIDLNRLLALNIAFEKALIATATWFQYYTEDQGQFPLQYAIRSVFANHFLLSVCQKMKLCAIFDNLRYLI

PAVTSLYSGFPSLIEKLFERPFKSALEVYVYYNIKSLLVALAQNNKARFYSKVKLLGLTVDQSTVGASGI

YPSFMSRIVYKHYRSLISEVTTCFFLFEKGLHGNMNEEAKIHLETVEWALKFREKEEKYDESLVENGYMM

WELIENPDLAEQQLYCQDAIELAAIELNKVLSTKSSVVANSILNKNWEGPYFSQTRNISLKGMSGQVQED

GHLSSSVTIIEAIRYLSNSRHNPSLLKLYEETREQKAMARIVRKYQRTEADRGFFITTLATRCRLEIIED

YYDAIAKNITEEYISYGGEKKILAIQGALEKALRWASGESFIELSNQKFIRMKRKLMYVSADATKWSPGD

NSAKFRRFTAMLHNGLPNDKLKNCVIDALKQVYKTDFFMSRKLRNYIDSMENHDPHIKQFLDFFPDGHHG

EVKGNWLQGNLNKCSSLFGVAMSLLFKQIWSQLFPELDCFFEFAHHSDDALFIYGYLEPVDDGTDWFLFV

SQQIQAGHLHWFSVNTEMWKSMFNLHEHILLLGSIKISPKKTTVSPTNAEFLSTFFEGCAVSIPFVKILL

GSLSDLPGLGYFDDLAAAQSRCVKALDLGASPQVAQLAVALCTSKVERLYGTAPGMVNHPAAYLQVKHSD

TPIPLGGNGAMSIMELATAGIGMSDKNLLKRALIGYLHKRQKSMLYILGLFKFLMKLSDETFQHERLGQF

SFIGKVQWKIFTPKSEFEFADMYTPKFLELWSNQHVTYDYIIPKGRDNLLIYLVRKLNDPSIVTAMTMQS

PLQLRFRMQAKQHMKVCRLEGDWVTFREVLAAANSFAEGYVPDNQDIDLFQTLTSCTFSKEYAWKDFLNG

IHCDVIPTKQVQRAKVARTFTVREKDQIIQNSIPAVIGYKFAVTVDEMSDVLDTAKFPDSLSVDLKTMKD

GVYRELGLDISLPDVMKRIAPMLYKSSKSRVVIVQGNVEGTAEAICAYWLRSMSLVKTIRVKPHKEVLQA

VSIFNRKEDIGQQKDLAALKLCIEVWRWCKANNAPYRDWFHALWFEDKTFSEWLDRFSRVGVPPVDPEIQ

CAALMIADIKGDYSILQLQANRRAYSGKQYDAYCVQTYNEETKLYEGDLRVTFNFGLDCARLEIFWDKKT

YILETSITQKHVLKIMMDEVSKELLRCGMRFNTEQVQGVRHMVLFKTESGFEWGKPNIPCIVYKNCVLRT

SLRTTQAINRKFMITIKDDGLRAIAQHDDDSPRFLLAHAFHTIRDIRYQAVDAVSNVWFIHRGVKLYLNP

IISSGLLENFMKNLPAAIPPAAYSLIMNRAKISVDLFMFNDLLKLINPSNTLDLSGLETTGDGFSTVSSM

SSRLWSEEMSLVDDDEELDDEFTIDLQDVDFENIDIEADIEHFLQDESFYTGDLLISTEETEVKKMRGIV

KILEPVRLIKSWVSRGLSIEKVYSPVNIILMSRYISKTFNLGNKQVSLLDPYDLTELESIVRGWGECVID

QFDTLDKEAQSMVVNKGICPEDVIPDSLFSFRHTMVLLRRLFPQDSISSFY

>Dobrava-Belgrade_orthohantavirus_(from_Apodemus_agrarius)_(ADP21254)

MEKYREIHRDLQSFPVGSLTAVECIDYLDRLYAIRHDIVDQMIKHDWSDNKDSEESIGKVLLFAGVPNNV

ITAMEKKIIPDHPSGKTLRSFFKMTPDNYKITGSTIEFVEVTVTADVDKGIREKKLKYEAGLNYIEQELH

NHFLKGDIPQPYKITFQVVSVRTDGSNISTQWPSRRNDGVVQYMRLVQAEISYVREHLIRQEERAALEAM

FNLKFNISNVKNQPYYIPDYRGIALIHPNINDLVVYMKNWLSKSHKFSFHEVKGPAVFDCFNENEIEHAV

KYPISRHPRNFLLIQCSLLSSYSPATILSDQVDSRRACNFVLNLIPETPTSFLIHDMAYRYINLTREDMV

SFYAPKTQFVPTQNVKEPGTFKLTANSMRPESKAMLDMLGSHEPGEKKGALIESLNLSSHIVQSECVSLI

TKILSDLELNISEPTSHGSFTTKHTYVDNVLDKFFQNEIQRYLLDVLKKTTAWHIGHLIRDITESLIAHS

GLKRSKYWSIHAFNNGNVILFILPSKSLEVAGSYIRFVTVFRMGPGLVDKDNLDTILTDQDVTWGVSKVM

SIDLNRLLALNIAFEKALIATATWFQYYTEDQGQFPLQHAIRSIFAYHLLLAVCQKMKLCAIFDNLRYLI

PAVTSLYSGFPSLINKLFERPFKSALEVYVYYNIKSLLVALAQNNKARFYSKVKLLGLTVDQSTVGASGI

YPSFMSRVIYKHYRSLISEVTTCFFLFEKGLHGNMNEEAKIHLETVEWALKFRQKEEQYGESMVENGYTI

GELNDNQDLVEQQLYCQDAVELAAVELNKILSTKSQVVANSILNKYWEVPYFSQTRDISLKGMSGQVQED

GHLAASVTIIEAIRYLSSSQNNPNVLQLYEETRKIKAQARIVRKYQRTEADRGFFITTLPTRCRLEIIED

YYDAISKNVAEEYISYGGERKILCIQSALEKALRWASGESFIELSNGKFIRMKRKLMYVSADATKWSPGD

NSAKFRRFTAALHNGLPDDRLRNCVIDALRNVYKTDFYMSRKLRAYIDNMNGHEPAVKNFLEFFPDGHCG

EVRGNWLQGNLNKCSSLFGVGMSLLFKQLWNELFPELDCFFEFAHHSDDALFIYGYLEPIDDGTDWFLYV

SQQIQAGNLHWFSVNTEMWKSMFNLHEHVLLLGSIKISPKKTTLSPTNAEFLSTFFEGCAVSIPFIKILL

GSLSDLPGLGYFDDLAAAQSRCVKAMDLGASPQVAQLAVALCTNKVERLYGTAVGMINHPSTYLQVKHAD

TPIPLGGSGAMSIMELATAGIGMSDKNLLKRALLGYLHKRQKNMAYILGLFKFLMNLSKDTFQHERLGEF

SFVGKVQWKIFIPKSEFEFFDMYTPKFLKLWSEQHVTYDYIIPKGRDNLLIYLVRKLNDPSIVTAMTMQS

PLQLRFRMQAKQHMKVCKLDDNWVTFREVLAAANSYAQSYEVTQEDLDLFQTLTSCTFSKEYAWKDFLNG

VQCDVIPTKQIQRAKVARTFTVREKDQIIQNSIPAVIGYKFAVTVDEMSDVLDSAKFPDSLAVDLKTMKD

GVYRELGLDISQAEVMKKVAPMLYKSSKSRVVIVQGNVEGTAEAICGYWLRTMSLVKTIRVKPHKEVLKA

VSIFNRKEDIGQQKDLAALRLCIEVWRWCKANNAPYHEWFHALWFEDKTFSEWLDRFIRIGVPPVDPEIQ

CAALMIADIRGDLSVLQVQANRRAYSGKQYDAYCIQTYNEETKLYEGDLRVTFNFGLDCARLEIFWDKQT

YVLETSITQKHVLKIMMDEVTKELLRCGMRFKTEQVSNVKHLVLFKTEAGFEWGKPNIPCIVYKNCALRT

GLRTNQTINHKFMISIKDNGLRAIAQYDDESPRFLLAHAFHTIRDIRYQAVDAVSNVWFIHKGIKLFLNP

IISSGLLENFMKNLPAAIPPAAYSLIMNRAKISVDLFMFNDLLRLINPSNTLDLSGLQPTEEGFSTVSGL

SSRLWSEEVSFVDEDEEIDDEFTIDLQDVDFENIDVEADIEHFLQDESSYTGDLLIMSEETEVKKMRGII

KLLEPVRLIKSWVSKGLCIEKVYSPTNIILMTRYLSKNFNFSGKQVSLLDPYDLTEFESIVKGWGECVVD

QFSTFDQEAQLLVSQKGICPEDVVPDSLFSFRHTIVLLRRLFPQDSVSTFY

>Seoul_orthohantavirus_(from_Rattus_norvegicus)_(AQR58375)

MEKYREIHRDLKEFTINSLTAVECMDYLDRLYAVRHDIVDQMIKHEWSDNKDSEEPISKVLLFAGIPNNV

ITALEKKVIPDHPSGKTLRSFFKMTPDNYRITGSLIEFVEVTVTADVDKGIREKKMKYELGLRYLEQELM

TFFHRGELQNPYKITFKVVAVRTDGSNISTQWPSIRNDGVVQYMRLVQAEISYVREHLVKTEERAALEAM

FNLKFNISSLKTQPYFIPEYKGVDLIKPDIDGLVNYAQSWMSKTQEFSFFEVKGSAVFDCFNENEQGHIV

RYPMSRHPRNFLLIQCTVLTAYKPATILSDQLDSRRACIQFLNLIPETPTSILAHDMAHRYINLTRDDLL

AYYAPRIQFNPTQNIKEPGTFKLTSNMMRPESKIMLDMLSQHEPRENLGKSIESLNISSHIVQSDCVSLI

TKILSDLELNISEPSGHEQITAKHTHVDTVLDKFFQNETQKYLIDILKKTTAWHIGHLVRDITESLIAHS

GLRRSKYWSIHAYNNGSVLLFILPSKSLEVAGSFVRFMTAFKLGPGLVDKDNLDSILADGDILWGVSKVM

SLDLNRLLALNIAFEKALLATATWFQYYTEDQSQFPLQHSIRSVFAYHFLLAICQKMKLCAIFDNLRYLI

PAVTSLYSGFPSLVEKLFERPFKSALEVYVYYNIKSLLVALAQNNKARFYSKVKLLGLTVDQSTVGASGI

YPSFMSRVVYKHYKSLISEVTTCFFLFEKGLHGNVNEEAKIHLETVEWATKFKEKEDKYGEMLVENGYTI

GELVESSELAVQQLYCQDAVELAANELNRILITKSQVVANSILNKYWEEPYFSQTRNISLKGMSGQVQED

GHLSSSTTIIEAIRYLSNSRNNPNVLQLYEETRHQKAQARIVRKFQRTEADRGFFITTLPTRCRLEIIED

YYDAISKNVAEEYISYGGERKILCIQAALEKALRWASGESFIELSNGKFIRMKRKLMYVSADATKWSPGD

NSAKFRRFTAALHNGLPDDRLKNCVIDALRHVYKTDFYMSRKLRHYIDSMDTYEPHVRDFLNFFPDGHHG

EVRGNWLQGNLNKCSSLFGVAMSLLFKEIWTRLFPELDCFFEFAHHSDDALFIYGYLEPADDGTDWFLFV

SQQIQAGKLHWFNVNTEMWKSMFNLHEHILLLGSIKISPKKTTLSPTNAEFLSTFFEGCAVSIPFIKILL

GSLSDLPGLGYFDDLAAAQTRCVKAMDLGASPQISQLAVSLSTSKVERLYGTSIGMVNYPGTYLRTKHSE

TPIPLGGSGAMSIMELSTAGIGMSDKNLLKQALIGYMHKHQKQMSYILGLFKFLMDLSGETFQHERLGQF

SFIGKVQWKIFTPKSEFEFSDMYSQKFLKVWSEQHPTYDYIIPKGRDNLLIYLVRKLNDPSIITAMTMQS

PLQLRFRMQAKQHMRVCRLDGDWVTFREVLAAANSFAENYEPSQNDIDLFQTLTSCTFSKEYAWKDFLNN

VHCDVIPTKQVQRAKVARTFTVREKDQIIQNSIPAVIGYKFAVTVDEMSDVLDTAKFPDSLAVDLKTMKD

GVYRELGLDISSPDVMKKVAPMLYKSAKSRVVIVQGNVEGTAEAICAYWLRNMSLIKTIKVKPHKEVLQA

VSIFNRKEDIGQQKDLSALKLCIEVWRWAKANNAPYRDWFHALWFEDKTFSEWLDRFIRVGVPPIDPEIQ

CAALMIADVKGDRSVLQLQANRRAYSGKQYDAYCVQTYNEETKLYEGDLRVTFNFGLDCARLEIFWDKKT

YILETSITQKHVLKIMMEEVSKELVRCGMRFNTEQVNGVKHLVLFKTDSGFEWGKPNIPCIVYKNCALRT

GLRTNQAINHKFMITIKDDGLRAIAQYDEDSPRFLLAHAFHTIRDVRYQAVDAVSNVWFTHKGIKLYLNP

IISSGLLENFMKNIPAAIPPAAYSLIMNRAKISVDLFMFNDLLRLINPGNTLDLSGLEMTGEGYSTVSSL

SSRLWSEEMSLVDDEEEMDDEFTIDLQDVDFENIDIEADVEHFLQDESAYTGDLLIMSEETEVKKMRGII

KLLEPVKLIKSWVSRGLSIEKVYNPVNIILMTRYISKNFNFSGKQVSLLDPYDLTELESIVKGWGESVVD

QFDSLDLEAQNLVQKQGIVPEDVIPDSLFSFRHTMVLLRRLFPQDSVSTFY

>Anjozorobe_hantavirus_(from_Rattus_rattus)_(AHA83439)

MEKYREIHRNLKEFATNSLTAVECIDYLDRLYAVRHDLVDQMIKHDWSDNKDVEEPIAKVLLFAGIPNNV

ITALEKKIIPDHPSGKTLKAFFRMTPDNYRIQGNLIEFVEVTVTSDVDKGIREKRLKYELGFNYLELELH

KYYLKGELPQPYKIRFQVVAVRTDGSNISTQWPSPRNDGVVQYMRLVQAEISYVREHLIKNEERAALEAM

FNLKFNISNVKNQPYYIPEYRGIDLIFPSIDDLVAYSQDWLSKARNFSFFEVKGSAVFDCFNENEQSHMQ

TYPMSRHPRNFLLIQCSLLNAYKPSTVLSDQIDSRRACIQVLNLLPETPTSCLVHDMAHRYLNLTREDMI

SFYSPRIQFVPTQNVKEPGTFKLTANMMRPESKIALDMISSHEPGANKGQLIESLNISSHIVQSDAVGLI

SRILSDLELNISEPSGSEPIIAKHTYVDGVLDKFFQNETQKYLIDILKKTTGWHIGHLIRDITESLIAHS

GLRRSKFWSLHAYNNGSIILFILPSKSLEVAGSFIRFVTAFKVGPGLVDKDNLDAVIVDGDVTWGISKIM

SIDLNRLLALNIAFEKALIATATWFQYYTEDQGQFPLQHSIRSVFAYHLLLAVCQKMKLCAIFDNLRYLI

PAVTSLYSGFPSLIKKLFERPFKSALEVFIYYNIKSLLVALAQNNKARFYSKVKLLGLTVDQSTVGASGI

YPSFMSRVVYKHYRSLISEVTTCFFLFEKGLHGNVNEEAKIHLETVEWAIKFKEKELRYGEAMVEHGYTI

GELVENPDLVEQQLYCQDAVELAAMELNKLLVTKSQIVANSILSKYWEEPYFSQTRNISLKGMSGQVQED

GHLSSSVTIIEAIRYLSNARHNPNVLQLYEETRQIKAQARIVRKYQRTEADRGFFITTLPTRCRLEIIED

YYDAISKNVAEEYISYGGERKILSIQAALEKALRWASGESYIELSNSKFIRMKRKLMYVSADATKWSPGD

NSAKFRRFTAALHNGLPDNKLRNCVIDALKNVYKTDFFMSRKLRAYIDSMNGLESNVKEFLGFFPDGHCG

EVRGNWLQGNLNKCSSLFGVGMSLLFKEIWAQLFPELDCFFEFAHHSDDALFIYGYLEPSDDGTDWFLFV

SQQIQAGKLHWFSVNTEMWKSMFNLHEHILLLGSIKISPKKTTLSPTNAEFLSTFFEGCAVSIPFIKILL

GSLSDLPGLGYFDDLAAAQTRCVKAMDLGASPQIAQLAVALSTSKVERLYGTAVGMVNYPGSYLRTKHAE

TPIPLGGSGAMSIMELATAGIGMSDKNLLKQSLVGYVHKHQKHTTYILGLFKFLMNLSDETFQHERLGQF

SFIGKVQWKIFTPKSEFEFSDMYSSKFLKIWTEQHPTYDYIIPKGRDNLLIYLVRKINDPSIVTAMTMQS

PLQLRFRMQAKQHMKVCRLDNEWVTFREVLAAANSFAESYEPTQNDIDLFKTLTECTFSKEYAWKDFLNN

VNCDVIPVKQVQRAKVARTFTVREKDQVIQNNIPAVIGYKFAVTVDEMSDVLDSARFPDSLAVDLKTMKD

GVYRELGLDISLPDVMKKVAPMLYKSSKSRVVIVQGNVEGTAEAICAYWLRSMSLIKTIRVKPHKEVLKA

VSIFNRKEDIGQQKDLSALKLCIEVWRWAKANNAPYRDWFHALWFEDKTFSEWLDRFIRVGVPPVDPEIQ

CAALMIADVKGDRSILQLQANRRAYSGKQYDAYCVQTYNEETKLYEGDLRVTFNFGVDCARLEIFWDKRT

YILETSITQKHVLKIMMDEVSKELLRCGMRFNTEQVNSVKHLVLFKTESGFEWGKPNVPCIVYKNCALRT

GLRTNQAINHKFMITIKDDGLRAIAQYDEDSPRFLLAHAFHTIRDIRYQAVDAVSNVWYIHKGIKLFLNP

IISSGLFENFMKNIPAAIPPAAYSLIMNRAKISVDLFMFNDLLRLINPNNTLDLTGLEITGEGFSTVSSL

SSRLWSEEMSLVDDEEEIDDEFTIDLQDVDFENIDLEADIEHFLQDESAYTGDLLISTEETEVKKMRGII

KLLEPVKLIKSWVSKGLCIEKVYNPVNIVLMTRYISKNFNFANKQVSLLDPYDLTELESIVRGWGEIVVD

QFDSIDKEAQSLVSNKGIIPEDVIPDSLFSFRHTIVLLRRLFPQDSVSTFY

>Sanxia_Water_Strider_Virus_2_(from_unidentified_Gerridae)_(AJG39245)

MTYQANIEGLDYELPDFSDPILCAEIYWKYINLRSMVAERYVHDHFSIYDRDASVHDTQVNEYLYKKGLN

TVQTNQTPDVMVMIGHKLYVGDVTVTVNVRERQASKTEKYLELMEKIRQANNNLEVEFVVFAFEEDGSNV

DLILDKINLITSAVRHAPNVITGQAEKELVRELKRINDYCKNIKNNVQDKETFINEMETQQGIIDNYEIG

TGRHVERTKPLFTERAITLNLFKKVDKTNTTTKKTRDVDLNAIRQELAEKNDETFARRQPSQALPFLMNF

NNDETEMNLNLLKSYALDIKSDSEMFSLFPHGEQLKNMIDLFNDLNIGKISESNLGKTKDEILENAKEIY

GQTSPYYCKENALRQRNGFTSTYSYYKEKINKMNEPKPPESIHSCDYKMCISNTNKLIDLLGSQCNSTST

NLTYDKNMRADRYNREKMNIDDVDDIFQQINKTIGVNLASVMRKFVTDIAYIPKRFKKGIYYSIPVQKSG

IVLTFTDTNFRARNPTIYFASITRVSNKINGSKNPDIALFQENYFSQNIIDIYKSENYTYFASKLYRYDL

ETLTKLVNADKELRTAIMCIATQLPEGDEFTRKLEVEKKKKLVKKISGVFTLLHLDLHQKSSIILDLMKY

LTAMPMSEYSRLDILLKDKLNIMLKTNLDVWLVNRIFGYLKENIRLQQYRRKNNIIRVGGELSSTSFNIR

GEFALFCDLEYKTDSVMLYLTEAQLLFQSRPKKLYKSQFINKAAVKVAKNNKEMALQEGKNPGWVIKGED

VNLRKIWSFANRVVKMNKPFQDVSQLSKQERLEQSEAAADLQRGYKLIDDPDSFSFPYESGFAWSKDVVY

YMQQLNNSTHADRISPNKHTIYKKLGELFIYNLSLRGSCIIPGSELDKRFNKNFNGENIDERKSKDDGKA

RRVKNIKIKRSTTAMLNGLMSMKDHIESDNTESSRIHKILTDNLTRQMYFNMSEKEQRGDGRPIGSPDFF

TKQKLYAIEMAYQIIGKNDPANLMARGVHRGAKISNMTRGMIKDSLDSNRRYLFYIVMDQSQFSEGDNVA

KFEEYIRNTADIPYNIKRDMMDVMLGMQNRIQFFPKLPDQLKQNYSSEIVQNGVKGVAGWVQGMFNIIST

DFHASLARWLVHCFNKFYYEHYQEDYNRDMDLYGSNLKLGETLTPIMYDQAVNSDDSLIAISSYNIKIIE

RFNEFFVLGKRLGCLEQNIKKSYTSSMIAEVIQKYAVNGTTVNIWAKDATALYANIRGLDLSKDISAAIG

SLQTISRNGCPENICTYIRAELRNMIFRLYNLEKFNNLARIGIDINMLPVELGGWPGHLSTFELLMAGSQ

AQSNYCRKEFLKEDNNSIEKHICATSVYLNLLSNLNKNESEGVLVFKKDKPKEELITGKDIEMEFKQTID

EIDNSEFDYYSNDVVELAKNTQLEYEEKNIEIAYKSLGFSDNVDNYCRTLINCMSYIIKVPVKVSDTVVE

IKKYKGKESGLSGLLKIRSSIISAVSDVAENLNVMLTSLSDANFSKDIKAMSAAVSYVAFNRCIVISGLK

DRHTLIGAYKILKEIATVKRNKAVIIDSYKKAIQALTDQSDRSVYAELAITNTVLTGDFTTSGNIVVNKI

PRTKDALNLQNDLQLVLSDILKPSILEAEGYKLKYPLQMRLDRQAILHQYEDWFKLSADKKKMCQTIYYY

YLNIKRSRYRISFFVPDDNLSGYTNSCYLNIRDMKTKLIGNYESTVREKLLVKDEETKKIYSTIRTYYEI

FMRFCIRERLTDIETFVDSVDIEIDNRIGNYRIHKDKLNYLSDIDQDLAEIVGVLNLLIDNDQKLLDRNL

TTDMFRTEWVKEQNKTYNRRTGKVTWSGDFHVRLMKKNTIVVLIGAPGCISSLYSNTEDTKAIRELLYLF

RVNNRFEGYSVNIRYKSIFYDGFFDISPVALATNDYIIEERPGIGMVIVKNPYKKLNFNTTTLNKIPFEF

RSYRIEKLEKMRYIDYKVEFDNNKLQLKGGLNETKTIIDDKGRSVKVVGLVWKHIKNIVSFVHRFYPEQL

MFKENLKIMGFPLNELKSNGSLLEFVRNKLYNLNIGQLLNLLKYIKDDNTIVLETIMNLIELTYNENDMF

SRASNLTEAINTIRVDVGNQIKIMDQIDQEDQFADPDYMNEYIDVTDTQFSRTESIILALNSKQFDKMNY

YKDLISGYVLSGFDDYISNLDFVKKDGTNFKGEKTEFYRDDTVDWADEVESDHFIKEHNNNVLLSTVGNA

LSLYFDNIKTKGTNRAYKKLSFLIEASLKYTSFMPESFNEEVYKEHYETMIEYNDKPVGDDNDLTDGFNI

MNLILSILVEGAPSLDFDYKEKENQQDESESSFF

>Notori_virus_(from_Drosophila_suzukii)_(AWA82264)

MNVTNLFERIKVLIKERSIESENLVFNIIERSKTTDTIFKIGDIKLKDEEEMTPFGLKPINTNKTPDILIKKGDNYWIGD

ITVTRAQWTALSIKTNKYFSDIQLLKQSNPTKRFNDIYIIIHESCNNIINDLKPLLDLFDVPYSVKILINQTKQSIININ

KKIKELEISAGADFDKLREMLFKDTNKKPSEIGSNKSIPKYTPKLSEEQLADMIFRKMKTDKVDESDMIDEHFNLSSVLN

QLKINNDEDFKPRKINSSLPYLINMEESLQTGPIDMLEEYRDDLALNSSNQSFILQMLPVNEHLNIMKKITTDYNETSKN

ANTKNTDKYKFREELKKKYGQSIQYWSHTKNLTRSGNEHHDLVQEYMNLNEKINAKKEPESIPSSEWTKCFELQEDLNNY

LSTKTELNMKSSNINFDIPDYGIDREERNIADIKPVFDKINNSKGFVLASIIRKFVNDLAYMPAISKAGIYYSIPVYKNC

IFGVLTNNNIGSKNPHYAFFTITRFKKDKEHSLFRTDSINNLSADINKHYPNLLRTIEETENYFYTITTLQKYTLEKFSK

LNNIDSEFRCHAMSMGLMISTLDKEKTESYFSKYIGGMTHLLLDLHQKPSEILDLMKYLVSMPFSARCSVKALLLDKLNI

MKKTPLDIWLILKIIGFFHRNVVFNKINKPNKMIITPSGISSMSFNVTGNYAMFFDESFVTSNLVLYLSESQLLFHARPK

KLYNSQFLDKACIKVAENNKQMDMEESSDISTWVIDGLPKDNEDFPFDSDYGFSRDALYYAQLDEDNILSKYADSIKTRI

SGIMKELSLHNLSMRGGCIEQETMAEREKEFNEKINNNKIRNSDDYKQKKKEKLSFGKSITSLKGGLDYMADIIKTDNTD

KARVINIALDTLDRKMLFNMSEKEQRGSGRPIGSPSFTTKQKLYLIEHTYKIIDKDQNENLLVKGVNRSAKISSIVTNSI

KTAVDKNMKEIFHIVMDQSQFSEGDNLRKFKSNIQDNLLIPQGLKGDLMDIIDSMMERVQFFTRLPDRIKEEDLIKYNFK

SNGIIGKAGWVQGMLNLSSTHIHIISVKWLAKLFNLYYSKFKGYDAPEIICEHIVNSDDSYCIVIGHDREQIMDFYDFLI

YGKKMFCLRQNKKKSYLSKFLGEIIQKYVAHGSVVNIWAKQAISVFNNNQGIDLTKDISSSIGVLQNLIRDGAPETMCTY

LRSELKNQLFRLYNLGKNKSNSLSKLNIDYSLLPCELGGWPSRLPTFDICQAGSPAQSTFCMNYYKLNPDCIEYNVTTAA

VMLNNYRISTNDDKTIIYEDGKDNNFKNETEKMKNKLADVLKLSSLDDDIIFIDEENIDELTQYELAVRKLPESNTDSNF

SKSMLNPFCFIIPCSKNVSDTIKTLKGFEYTPSGMAGLLKIRSSIKTAVSDLNDILNTMLINLSESGFSKDLRARSKANA

AIAGDKSVMLSGYRSRFTLIRAYNALIKVYFSVKKEKIDEYKNIVLVLLNDRTTRSQIAKSISSKAQLTSKHQMGNTIVT

KLPNPYDDLETSNDLQLVLSEMAKPGILAQEGYEMKIPSNFESDKITIINLFKDWIDSTEDKVNCYKAIYFHYLNSRRVR

WFTGYYIDNTTILTALKTIYSVSRDGAFVEGYQMGSLTNIPLKSRTINDNYNVIIMTIWELLLYCYKNCKINDETANKWE

TIAKSIEIDHEMYKGNVIDLLNDRTRVMEINNYLHDNDKRYLSALLFLVKGDHKLLIENNKQMDGMTIDWIKEQEITKNL

SGKIVYRGEFEVRLCKGNYSIIIDGKPGDINMIKANTNDIHIISKMIFQFLTRVQFEGYNKPKSYKKTFLTGFFKNNKCN

MSQFLISTDRFGLNTIVSNSERFKKFLSIVDDFNDEFIPFNYIPSLATNRSIPEAYSEYEYNHSTRCIRAKVEKMHRKYD

YINGGSIEELVTESVNIISLKDTLYMVDYKIIKDTGFLNVEGIPVQKLIEKGIMRHITRNEYHKISSSSAIGIIKDIITE

EKIFYRSMINLFNTCRDDNIFKTSVTNDIELEEVIIEVSTASTMGILKSMAQNLDDPEIMDFDDNYITDKDEIKIDDVSL

IKGMLSIIGRQITSQSIISTIPGMLFNETTSKILKCLKEADDDIKNGDWEYRDDNKDDHIINMMSIIENIITEERFKNVE

IPSDSMKEKRRKTMRNLEIDQIIEEMIQTGIMVKAVTVNKTSKYFDHLKKLFDDDNFEDMDIEYIRQNDPIDFTNSTFLD

KNLRIATIMDGYSGHVYSIVE

>Wuhan_Insect_virus_2_(from_Hyalopterus_pruni)_(YP009270651)

MDNYLNVVDYSDAQACSDEVSSLLFKRHEVAGEYVNIALARVVISLVTDDVNINEWILDNDLNPLSKNYKKTPDLIFIYG

RSIFIGDIAVTRTVKSVEAIKSKKYDELRTLIQSANPHMTVEELNLIFDENGTNISDISRKLRSWFTGGWSERAAMEFKS

TLCMCTDKISEIEANVRDYPLFLQYRNEQNSNNPNLIDSMLDKSLADEDIYDKKYQPVYSELDLFNMAFEHSKGVTSSMD

KMSEIKDLSDTRKELDTIIMESYDKRDIRSPLQYVFNLENEETQSGIDLIESYYHDITMGCDPNDQNRLRFLSVLPHGTQ

IDKMKNIDNEYKTRRLVFKEETMEGNFANKMKEKYGQKHAYYAPETGLAFNNRGEKNMLRMFIEKSKTVNFNTRPKSIYP

EDYQEAIKNTKELISYLSRENGKGHTSTNLTFDVPSGRRSTRELENIQKILPIYETINKRNGVILLTSIVKWIQDIVRIK

IGNNTGIYYSVPIQKNMIMCTITGNSMYGNNPHYNYFSMTRFEYNETIDNSKLEILNFINSVFNNMICCHVVSKQYIYIM

SKLQKTTLDKLEKIVTVDYEMRSHAMAMGILFNETTVLDYDKMFNNKYYDINQFNNTDEFVKKAKSVYDSTNNELSKKNI

INLSDYFNEYIGANALIMLDLHQKPSEILDLMKYLSGINFSELSNIRTLLKDKLTMMRKTSLDVFLIEKMLAFCERNIRI

QSYCRPCKILMSDTGIIPSSFNVTGIFSSFWHEELYTSSLNFYYSEAQLIFQARPKKLYNEQYMAKACLKVLENNKKFEL

EEKEAPGSCSKGMIYRKFNFNSSFNYSRDAIYYAQELMNKTYRKFAKSQKMKIAGKLQEIGISNVSMRGSCKLEENMRAG

QGKSQTSLYSCLEYLSDMILRNDTKSTKLIELVKGSKHRNQCYNMSSKEQRGGGRPIGSPDFPSKQELYLVESVYKILSI

VQNENLLVKGVNRSAKIASINRTVISKAYSERYKEIRHIVMDQSQFSEGDNVNKFCDFIWFNENIPSRLKRIMIESEKKH

MNRRQYWPLLPLDAEKNYPGMVLPLNGTVGKAGWVQGMKNIISTYAHIAAVTWIIDIFNKYYFTLPGNDMYGEHKGLLCD

QIVNSDDSYIIVAFKHIKPIHDFYSFMISAKRLFRLEQNTKKSYITGTIGEIIQKYVANGTIVNIWSKSAVSSFRNNMGV

DMARDVSNSVSALGSLMREGAPETMCTYIRAELKNQIFRLYNIGKGRFNDLSEVGIENYRLPCELGGWPTQVTTYELCVA

GLQAQLDYCKEYYKQNQDCNEYKIVSCSIQLNMMRFTERDIWQRIKASIGVSNTRDLISQLPSLKVSNIDMSKSDDEGKA

LKLLQHMMFLDYKYLEKENKVFDLIKPQIKGQDNGDATVSNEFNISNEGLTSYDSSFARSTINCINWIINVPERVTKTLE

IMKRFEHLKPSGLAGLYKERMSIQTGVADLIDQSNSMIIKLSEMNYTKSIRQKAAANAFAATQRCCSISGIPYKMSIVGV

YFVLLGLSDRLGSIFNKNISAEFVIRVMTDPTSRADIARDIVQNYEKETEAESDSYVANKLPRSEDDIILHNDIQSVLVE

MVKPGHMKDQGYIIRYEARLNADVETIKLIYKDWIEISKNMVNTVRTIYFHYISVRRSRYIIAPQLETKNMNDALLSIFE

KCQNIATKNIGVYRKPKSNNYSVKRTEETRTIINNLMGGLEVVMFLEKNYNVPATESMKEIEMHIGLESGCLSDLVLNQD

LFGMYYNLSTPYEKKYLALLSHTAGDSTYLSDATKHNEFNIEWEKEQNFIVSAEGRKIYVGHFRVLISKGNDTVAVDGEP

GNIKCVYTTTYNIKFITDALFYFLKKTPFDGYPRPVEKKQWGNSTFWNSKNSTTSLKLMYSRFGETRIERNEENTIDRKM

GKRLVYKPEQNSLQIAVDTPTNYVTYIPLEYNHNLNGSVGFGVTYNEFLVSDNKVFGLKYKTDLRSRTDPITGKRYTDNN

FKREKCFLFGYKDAHKSSSYSMLDFTNIMIDTIKLSSLAKYGMLKNLILGELIKSSRNQIVKFMTNEVNPSLVVVETIKR

AISAVSGVDIPSTLDTSTNLSVETIGISLDNDIMDNIKDLDDDEEIIEDIILEEDEEEYGGVNFGLNLAKSIPSILAELS

MITVDVSKARMVVDTFFGSEISNLLTDNIIEIENKVNNISGIEVDDWAESVEAYNDKDITLSKIDSRLYNILAPIIDLEH

TSSDSIKRENMNTLIVAILSSGLLSKETWHVKDIKKKVDTYHYAIKSNTRYVKQEYTRDAGIELYFEYQIIKTLLDRIYK

NRMSDFDPDAPPDFGSSSGSGSTHSTRGNIADQFSASRRSSSGSIEIWS

>Hubei_bunya-like_virus_10_(from_Odonata)_(APG79271)

MAFTNGGKDNMFNDFLSKSNRVNNTKKPASIHPCDYDEAIENTIELCKYLSKENENNHTSTNLTFDIPGGRRSTREMENV

SMIEPLFETINKRNGVILLSSIVKWINDIVRIKIGHKRGIYYSIPIQRNMIMCTLTGNSMHGSNPHYNYFSIARFRYPID

DSGKAIEILDKITKLFSNMIVSTLISENYVYIMSKLQKTTLDKLEKIITVDHEMRTHAVSMGVLFNETIMMNYKDMFKNN

YYDINKFNDVTEYIKSVKHVYNSLEKGENVKNLITVEDYFNSYIGANTLIMLDLHQKPSEILDLMKYLSGINFSELSNIR

KLLIDKLAMMRKTNLDVFLIDKLLKFCERNIGIQSHCKPSKILMSTTGILPSSFNVTGIFSSFWHDDLYTSSINFYYSEA

QLIFQARPKKLYNEQYMAKASLKVLENNRKYDQEELQKNGTCSEGRIYGTFNFDSSFNYSVDAVFYAQTLMNLNYRKFAT

SHKLKIAGKLQEIGISNVSMRGSCKLEENMKPGIGKSQTSLYSCLEYLEPIITSGNTNASRLIELVKRSRHRTQAYNMSS

KEQRGGGRPIGSPDFPTKQELYLVESVYKILSLVQNENLLVKGVNRSAKIAAINRTLISRAYSVKYKEVRHIVMDQSQFS

EGDNVNKFCDFIRFNDNIPSRLKRIMIECERKHMSRHQYWPLLPLDAEVNYPNMILPLNGTVGRAGWVQGMKNIISTYAH

IAAVTWIVEVFNKYYFSLEENKGLYGEHVGLIYEQIVNSDDSYIIVAFKNVKPIHDFYAFMISAKRLFRLEQNFKKSYIT

GTIGEIIQKYVANGTIVNIWAKQAVSAFRNNMGVDMSRDISNSISSLGSLLREGAPETMCTYLRAELKNQIYRLYNIGSG

KFNDMTTIGIKNYNLPCEMGGWPTQVTTYELCVAGLQAQLDYCKHYYKHNPESTEFKVVSTSIQLNMLRFTDSNLWHEIK

RRYKIKNINDITSELDEVDISGVDLTHTDPIRGACKLLYYMISLDNEYVRKKKEDQHVRKIMINGKDEDTFSSANEFNIS

NEGLSSYDSSFARSTINCINWIINVPKRITRTLEMINKFKHLKPSGLAGLYQERMCIQTAVADLIDQSNSMIIKLSEMNY

TKSTRQKASSNAFAATQRCCMISGVPYRMSILGTYYVLLGLNSLLLSVFNQKISPSFVHRVLSDPTSRANIAREIVQNYE

KELTVESDGYIANKLPRSEDDITLNNDIQSVLVEMVRPGYMKSQGYIIRYEARLNADIETIRKIYKDWLDISKDMVNTVR

TIYFHYISVRRSRYMIAPALDTKNMNDALLSIYEKCQCISTKNIGVYRRTKTEMPSVKRTEETRLMIDNLMGSLEIVLYL

SRNYNMTFEEIMSSISILADDAHIKLDNFILENSGFGVFYNLATPYEKKYLALLAHYAGDSSFLTDATKHNEFNIEWERE

QDSIINPEGYKIYTGPFRVLISKGNDTVCIDGEPGDIKCIYTTTYNIRFIEDALFYFIRKVPFDGYNRPTEKIKWSNTPF

WNSRNTDSSLKLMYTIYGDTKIEKNDKQVITYKKRAAISTDSLKKTIQVSLDADTKYVTYLPLEYNHNLNGSTKKGISYT

RFKISNNKVYGLVYKTDNRERTDPITGKKYIKGEFAVESDFLFSYKDAHKSSSYSLLHFSKIFVDGIKLESLSKYGMLKN

LLLSELIKTPRAQIMNFMLNEVSPSFLFVEFVKRLIGCITGTTLPSNLDTTSSLATEYIGIDIGGSSADQMKAFNDLSEE

NDMFDAEEDDDDLYGGVNFGNDLSKSVTSVLVELSMITIDVSKAKLLINCFVNSKIFTEITELCVMLFNATRVPTLDEDW

SEMVENINLTEVIGSIESTIYNRFGQLLEVHKTDDNSNIKDEMNTLLSCVLQSGLLSKTTWTIKSILTRANELSADINRK

AMPKKILYKRSDSINQYYESTLITKYLDYLYKNRLSKYNIDDAPGDDDFISGSGSNGRRLSTRSSSSLVSGKTDNDSLDI

WIK

>Seattle_Prectang_virus_(from_unknown)_(from_Pasiphila_rectangulata)_(AOF41423)

MLENNDAVNIYLQLRNLRHDLWIDYIKKARSKPYYKFNDIRVGEFHDAFQRKYPSPDIPERLRRLTPDILIHDTEMDIIF

IGDVSCSNSQMDADRRKYLKYKAIAEFYSSNGYRVRHANFIIENNLQNVTSMIRDFETTGIIMPVVGLRNRYIHYHRMCN

TAMEDSLTYSVDKVATVNMISVIDKEEDPSYDQLILPEDIKWPEVIEADPKVSEEELAEMIKQEVAKMDENYFDKDIMPA

LSAINELETRQIDNPIDEPKSTLKVCSNQSDVEIHTGHRLIESYLADIMLSEDEVIMEYVKWLLPSSRQIDIMRKYQTED

IDKVKAKENGVFGNYQYKIPRHTSDNMLINKTIEHLSRGKKLRNEKTKPETISPERFAEIFSSMEDTVYHYGSLSNKPPF

LDDSWDCATQLEADNSEEIRQIYKYVRKTNGAQICHSLSNFYQRVCHLKTSLSTKDNIYVPPNGSFIAIIPSDHAPVTSS

NCDLPMIFITRTKKTEKSHNEIIAINEFNNKFETDDYVYMVSKLCRLNVSKMANWDQAGYRLTANATYICSLNQNVSIER

VVGILTLMMLDVHQKTSELLDLLKYVAFMPFSDLTRLSSLIRDKFDILMKTKLDVWTLVTLKYLIYELSLVEKLNASKPK

LQLHNGIAIHESFGMHLELPSIFDLSKRHNTPDKYIEEISMIYTVRGKHLYGSQFMDKSIQQTAQWEDDYQEEVKKYSGW

VKDGEGDGDFPFDSKFAYSSSAIIHAMRTFDKIVPVSKGKAMRELTKGTYNDFMHYNCSLRGCVKEPNERSKPSDIHTTS

MEACLKRYQDNNYSTEKATSNEAAYDMFRSSNKMEFSMSEKEQRGGGRPIATPTLLTKAALMMLEKPEVAIGKQSPNNII

VPGKHKLQALCECYKRFITQASVEGYQLAFQVTEDQTKFSEMDNPRKFNTYINNNRHLDDNVKRIQLKTVEMLCNRTHLV

KRMPENVRDTKLARYLNPQKNGVNTTIGWPQGMCNFMSTSIHMIADYWITEMYNKAYPENKIKTAGLVHSDDSWVAIACN

DISDFERFCKFRIIAKKMFCLKMNEKKLWASRYLGELVSNYNINGNVHLCISKTIANSLNGLTYQNWVMDVHTQVSSLQQ

AYRQGANIPTLILLGTILRQQIMSSYQVKGNQKEYMDILPIELGGYPTNPVFDLAVNGVNCHYHKLLDYVKTNISSKIST

IILRCLRLSIMRLKSLEENDHTLDVRRAAKTQFLRNYLTTDEQNEGSLRLDYTNIRLPYRGDVFSCINHLMPQSVKIKKT

VKRIKALPFISDGLEMVVTRPRELDVSLGHLKEQTSTMLYTLAAEKYTQSARRLAVNQTLQSSGKTVRLSGMVPMTYNEL

LQAFLEMDNVPRATVDQLAAAFTDSNPIPDITEAIIYNSDHEVTNKDKRKIINRIPEIDDNFKTLSPLRDILLYIIQAST

NEDVFTKYSSSKSPIQLIQDESKIIKRRFSSYFTCYTVKVACNLIMRLNMENKKTKLWMQPYLNQETLTTFLEDLYGKTL

SQDVNYRLRAQIDSRREKNGDSELIKSVYSVCVLNKLYPGKFIIETVDEYPIGDMIGDIDVGKLSMDDLLKLAIIRKEIW

DCDILISEYDRSRMFSKKYIKAQRFVDGKYLGPIICDVRYGSTVMRIEGEPGSMSLTVNKYNINEILMAMMLFVNDNFPQ

DRYSHPTMWHMSRVWQTQFKFGKMFLTAYTQSATNITTTSQINSIPIMLAETMKFDDAFLTAGDTIYTIEENLRTVFKEI

DGKKIKIGNVKQNLSCPLAKRINCSYDKIDGITNNELLHSKIILNLTVKRPFANSKTDMEKMINDASQITDGIVVSMYKN

ILSKFTLSVTPHQFEHLKPLEAIEDYVIHGYDTSKYITAHDETEAADLSHVETNFMYEEIEDSGAITKYNSIIKYMAQCV

LLTQTEVDRDHVVSGILNDPSIIRKISADAMDEDTEELLNDMIETEDKTANITTICFIIANDLDNQHGWDRLDVKKILED

GITMGTKFSSNILQIIK

>Hubei_odonate_virus_8_(from_Odonata)_(YP009329887)

MDKYKYHKGKVDRRKVVEAIRPKALVEHAIEDVRSRLQSTENLTNPELVELYLSSRNNRHEVWINYLNNHNDFGIKYGDV

NFGLFCESLNIPEPDRKLRRLTPDILFQNPDTKYVFLGDVSVSVSTALANNRKYIRYKPLKDHLEKHGLTVKHYNFIVNE

DLSNVHNLLNEAYNMGLVDNTTEDLRRLLFFANASQWCMNECFNCSPNRQELKTLIDTQDKIEKVDPLEINVPKELHVDM

TSDRKKLSELEIIQMIKDEVESIGIDNYFDNGVDSTIEEFNKLEDSYANRDTCAPKSILKVADNQMEIEESSGYDLLESF

IYDLTFNDDNDVTNYIRHLLPTGPQIDRMREWYNKRRELTKAEKDSMREYNVFGPHQYRMRPMKDNRLVQNFKYQLSKGK

KTRNEKNAPKTIHRDNFDICRSDCERLINYYGTISKKPPFLDDSWDSATEFENDNSKVERENYHFAKSTCGAQLAHSLSG

LYQRISHLKIGQGKYDNIYIPPNGSFICVIPSAHAPVSNKNCDLPFIFITRVTHGSKAVFCEYEQIVDTDNYRYYVTSLS

RLNIEKIQVWDQAGYRLTACISHILSVCPELIPSKNRVAGLLTLLMLDCHQKPSEYLDLLKYVSYMPYSDISRLSSLVKD

KFQILLKTSLDVWLLLSLKDFIIKLADTCSIDAAKPKLQIYNNVMTKESQGICLKLPSFIDQSIRHKSAGSYIEEMGMLF

IIRGKHLYGSQFLDQSITKVCEWNEEYSKEVEDYGDWAVNGQGEGKFPFQSKYCYSSDAIQYAMEYAMTKFGASENDVLK

ELSNTTYGDYMHNNCSLRGCTKEADKRLNAHDLHTTSMDECLKAYVEEKYEDEKCTTIAIGLKHIISGRRQQYSMSEKDQ

RGSGRPIATPTLGTKASLCLIEKPEQAIGSRTNNNILVAGKNKLREMSECYKHLVSSAALKGYKQIYQLTEDQSKFSEND

NTRKYRNYIKTNTLLGNNVRAIQLAALDRVIDREHLTHRLPKNVISDKNLSKYINKDGNGVWTNIGWPQGMLNNISTSIH

SHADYWITRAYNIAYPKNKVETSGLVHSDDSWVAVACNSIHDFKRFTLFRIIAKKLFCLKVNEKKLWGSKYLGELVSNYN

LNGTVHLSISKILANSFCNLLYINWPIDVHSQISAIQQAMRNGASQPTLILMATILRQQITSSYMVKGTQLDLLHLLPIE

LGGYPKCSVFELGVNGLDCHYQYILNMLKREPTCKASIIILKALTLSVNKRYKEASSNIIVDKEDLMDAYRNSIGEAPLN

WGFNPVVLPSRGEIFCCISHLLPMTNKLNKTIVMLNQIPYETDGLENIITKPKDLASALGHLKATTKGRIYHLAAEHYSN

NVRRLAMCQSLQSGGKTVRLFDTPPLTMNEMLNSIYERVVPIAGYELAESALTDESKMSQICEAIVYMGVFTKSGVDKRK

IINKLPEHESRYRVISRLRNVLLFMIDQVRNSNLLSKYGDTIEPNDILVSDSVLIRKRFSSYFSYYSIEKACSLIMMQSM

DTLKTKLWMQPYLRNDNMKVFLEDLYGKTVSMHENFGVTSELAESFRNKDSDLVDSIYSTMLLNKMYPGSFEIESISGSN

PDEVIAAIDYKNLSGDHYLKYGIIKFVTKNDDSVIRSYDQSKQYRQNYRVRQQRNKHGAYEGRFIVQVQYGPVTIEIHHD

NYGNTDIVSNSNHIYYITNAMMQFVNRNFKEDSYTHPHRWSECPIYRNRNKFGRGFLTSYRQLCTTITTTPQMDSIPFRY

DPNLGLQLTASVDVAAEYSIDEDLRVVTKKVDDKTYRIGNAIQNLRCPMSKVIEIKQSFLDGIDNQMLYKNGLIFNISMR

RFGLCSVSDIRTCLEARTPSINSSCLVKFYLNLLSQFKKIDINLDDYNDEEFIIDDVDIHGITPVSCYDDINMTTAEDLS

CVVAEYEFTEDEKVQQGSISKFTSISRILATIMSRQYSHDDISNMIHILLNDSKFTNVLFDDIQKGQVHIDDVLVSVIEV

AQDEEIDTDIYSLIMGHKLDQTIGWANLKLRNIMSSNVDGVNNLGKLLRIANKISVFVTNFIHGMDEENKVETLKSLLK

>Hubei_odonate_virus_9_(from_Odonata)_(YP009329871)

MSHTRAFEYRGRADRIRRRGANDGRINPSSELEHSIDKTKHDLINAVDLSNSELLELYTQARVDRHEVWIEFLTRSRSDI

GYQFGDIKFGDFLTNVLGFRGAVPDEIKNKTPDILFRHKYTGIVYLGDVAVSASVNLVYQRKYLKYKELVDFLKKNKYAV

RDCNFIVNENLDNTTELINLMLNIGVINYNPDLVSRVRYFGAMAHSNMLACFNRSPDQMQLKQLIDHRDKTEQADAIDIP

VPAELAIDMTPLPTKHTEMELINMIKAKVDQIDPETYFDNGIEVTIKEFDKLEKTYDRDDITDPKSVLKVCDNQLSVESL

TNHDLILDYISDIIFSENKEVSDYVRFLLPTRSQLECMKALYHTRDKTVEGHLLESSKERSVYGPFQYKMMKADKNKLID

DTVVNLAKGKKTRPNSKEAPKTVHLDCFDQCVVNIEKMINYYGSPSLKPTFLNDDWDSSTNFELENSLVERENYDYCRHT

CGAQLAHSLSGLYQRLTHLKISQGKYDNVYIPPNGSFICVIPKEHAPVNSKRCDVPLVFISRSKNGADHSLFNEYEHKVV

TDTYTYYVSKLCRMGLDKIANWDQAGYRLVASSSHILSSCPELIGSKNRVVGVLTLLMLDCHQKPSEYLDLLKYVSYMPF

SDISRLSMLIKDKFNILIKTSLDVWLLRTMKKFMVCLADTLSLDAKKPKLMLFNHQMVKESQGISMKLPSFINMSVRHKS

VGPYIEEMGMLFIVRGKHLYGSQFLDQSTTSTAQWNVDYINECDRYGNWCTNGQGEGDYPFESNFSYSSDAIYYATLYGM

KNYKGTANDVLRNLSKTQYSSYMHYNCSLRGCTKEPEDRANSNDYHSTSMDECLTYYDRKNYDETQCTTIAVGLNHLLSH

RIQQYSMSEKDQRGSGRPIATPTLGTKAALCLVEKPEHAIGVKASNNILVAGKQKMKEMSEAYKSLVSSAAVENYKQVYQ

LTEDQSKWSENDNTRKYENYIKVNPLLDTNIRMIQLNVVRNIVNREHLVHRIPKQIANNPELLKYVNKDGNGVKAIIGWP

QGMLNNLSTSIHSNADYWITYAYNIAYPNNKVKTQGLVHSDDSWVTVACNSIHDFKKFTLFRIFAKKMFCMKVNEKKLWG

SRYLGELVSNYNINGTVHLSISKLLANAFNNLLYINWPIDVNTQISSIQQALRNGANQPTLILMATILKQQLLGSYQVRG

KHKELLHMLPIELGGYPSCSAFELAVNGTATHYQNLLGMLKTNPQCEASIIIGKALTLSIIRRVDDNEIINPELISSLQD

HLKMAEDEQTDWYYEKIHMPSRGEVFGCISHLLPMTSKLTRTITKLRNLPFETDGLEDVIYRPKELSTALGHLKSTTSSR

IYSLAAEHYSNNVRRLAMCQSLQSSGKVVKIYGCQPMTYEGAIRYILTSDYTICNYEVAECAMIDESMMSQLSSLIVNFG

TYTPSGHDKRKIINKLPETENRYKTISRLRNVLLFMLDSVRGTNFLTKYGVSVEPSDVLLNDSSILKSRFSTYFKYYRIE

KAISLIMTQSTELIKSRLWMQPYLKSDNIKTFLEDLYGKTVNSTTNFMVGSTTSEIYKNKEADMVDSLYSMILLNKLYPN

KFVVRSIGNVPIADAINNIDYAKLSGDHMFKYSILKKHHFDDDTFLREYDRSKDYCQNYMVRQTFEKGVYSGVFKVRIKY

GGTTMDIHHDGEYTEIKADNTNIYNITNAMMQFVSRNFKEMSYSHPHQWSSCPVFKPNPIKWGKSFLTSYRGLSTVISTT

AQHDSIPFTLNPNLSFRDTMVVDIADSYTLSEDYRCAFKHYGNRKARIGAAIQNMRCPFNKQIEVVPDVIEGLDNQLLVK

TGLIFNITMKRFGLCSPSDVDKLINSRLPAPTAKCLINLHQCILNSFKKIDVEIPEDDTTEVVIETFDMHGITPTDFINT

DSYTTAEDLTSHDVAFVFEDGEVEQAGALVQFHNLTLALCMIKTRHYSTEDVLNIISLIYNDPVLLRYLLKDVKSNISEI

EPRYKDLIEASNYIEIDSDLYCLIIGMRLSSRSFWNDLKVKDIMNKDLSNVTNIAQLQYIANKIISFIKQEIYEDAGDRN

RDEVLELLKD

>Hubei_diptera_virus_6_(from_Diptera)_(APG79294)

MNTITKYVGRVDRERNYIRQVEETIDNMTVVTLTHACIKMLVKDIAIDMPPDEAVQIYLSARSNRHNNWCDYLTYNTSME

GYKVHDTAIKEFVKDILKRDDIVPADIQDKTPDILTLDVTNSFIYLGDVAVTVATGSTRARKYEKYKPIANYLRKLGYLV

KHEDFIVQQDLSNLSGVIHKFVENGFIKKNHAHTHIFKEYSLLCNKIMTDASEFCSNKTQFNHLINEQDKTIKDSTFITE

PPESLLDIDTSDYIPRIDEDTLMDMIKTKVDSMDFNEYFTGDIQKSIKAINDLISVNLNRPHCKPKSILKVVDNSHYVEN

KSGHDLIIDYVEDILEADESPVVQFALHLLPNLKQLSLMKKISDKKITDKKGLKSAEFINSYVYGEYQYKISSSYQSFHI

ANFGTKLSYGKKLRNEKSEPECINLKFINDYYSNVSSTINYYGSISLKPPILPNTWEAKTKFEEDNTMNEKELYFYTKST

NGAQLCQAMALLYDRITHLSTTLSTKDNIFIPPNGSFVAIIPKEHAPISAKVVDLPFIFITRCPIKESLNHIEYEYTFNS

DNYTYYISKLCRLNIEKISNWANAGYKLLSTSTYLLARCPKLQVRKHKVVGVLTMMILDVHQKVSEFLDLLKYISFMPFS

DISRLPDLIKDKFDLLVKTKLDAWMIYTLKSFITELSSIPSLNAKKPKIRTFNTNVIPDSLGINLTLPSFIDNDIRHDNP

DEFIEEINMIYTVRPKHLYGSQFMDKSITSVCEWNLEHESEIRKFGGWAVDGFDNSDFPFEAKFCYSADVIHYAEKYLQS

QYNINRNSVENILSKTVYSSYMHKNCSLRGCTKDKKDRLNHTDIHTTSLDACLKYYKDNSYIDEKCTTTSVAYNFLMSGE

VQQYSMSEKDQRGGGRPIATPTLGTKAALMMIEKPEASKGRFMPNNIIVPKKNKLKEQCETYKSAISAGIRLKYKYVYQL

TEDQSKYSENDNCNKYEYYIRSNDAVGPKIKAIQLQSLKGLNNREHLFKRMPKGVSGDLDQYKINDSKSLGVKATIGWPQ

GMLNFMSTNVHCGADLWITMAYNRCYPDNTVYTRGLVHSDDSWVVVCCNHIDDFKRFTLFRLLAKRMFCLKLNEKKLWGS

RYLGELVSNYNLNGNVHLSVSKTLANSFANLTYQNWSIDVHNQISSIQQCYRNGAGLGSIILLSTILKQQLIKTYHVKGK

QLELLNKLPIDLGGYPDCSAFELAVTGVTCHYKSILEESKMNPKSSVAKMIAKCIDWSVNYMVCKEQTLLDQHSEATKRL

YMEALKNEDPRWRLEDYENLCIPSRGDIFKAIKHIMPKSKKLSKTVEHIRSLPFESNGLEMIVSKPLDLQDALGHLKAQT

STMLFELAAERYTQSARRLAMSQAIQSSGKVIRLNNYPAMTFNELYDFIESIKVDDEINLLMISNAFSDDNDLVGCAYDV

VHSSEAVLSDKDKRKVINYMPDVQDKFTTVGHLPDVLLYLIDESNRTSYLTKYSSPNVSMSTLKQDSDLITSRFIHYFRY

HNVIYACSLIMQQYLSSIKSKLWTQPHLRSDNLQNFLEDLYGKTINSTNNYIIYSTSGNNYVSTHDRDVVQTIYCTRILN

KVYDNLFTIVDIQGSTEVEALNKVDHSKLNDNDFLKFAVCEHIICGNRDYLDKYLQASRFNCRFIKAQKYINSRYVGDFH

ARCTVNDIVLDIIGDRGKFRLIANKPDVSGIMKIMRLFVASNFPYDRYELNGAWGNKDFWRSDIEFSDLYLSFYTTYSST

ITDKKGLYSLPLSIQPTLSYFYDKQKDLPDGYILDKSLRVVSSLNGNRTYKVGSITQSFSLPLRNYVVLEGNYLQGLDVQ

QMFSSGIMEAVILSKIHTIPVSSIEELLIDSGMITSKCVLETYLHLSSRCINHDLGWTNDRDDTTVEIETYTLEETCIST

VYNISELAEPEDLSALEQECINPPGRISGKLYHTNNILGEICKAYIGGLTDHRIKSFLHNLMYNRFINDWTISLYRVKQS

DPGFIMSIDDLMENADEMVGDQPTIDLFCFIMASDIDLTRYWDHITISSIKEISYTCENSPYIKTLTDDFVRCLNSVLFA

QELTPARCLTDI

>Apis_bunyavirus_2_(from_Apis_sp.)_(ARO50046)

MLSQNIFIVMDNNKTADDPDWFKNYMGKSDRKNLNVPSHDPIVVSLEPKTPTQQLLKNINKEINECMDKTGSLSIPWSDA

ISWYLNARMLRHDNMCDFITESLTIQDAKVGDTKLSDFCERYNICKIAEIPAAIASKTPDILLMDKASSCIFLGDVTITN

SVKMSIQRKHEKYILLVDFLREKGFLVNHIDCIFKSDRSNINQVTLELSNSGLIKINQGTLDVYNCYFELANSIMFLCRN

YNDSPIEFNAKLDSTDMVKPSNLILPNVPEHIIKALPPPYKPKMSEPEIINMIKNEVDKYDLSAYFNSDINQSLKTIDSI

INDNITKEHSEPKSTLQVVYNTSDIEVHTDLDLTLDYLKDIRMNDQPVADYMRYILPSDSQIKQMKRFKNKNNKIDVKTD

QDLKECNVYGPYQYKIGGIPANFQISEFNYRMSKGKKNKNIKSEPKSINLAFLDKYSNDIDITMSYLGQESAKPSMFNDD

WDCKTNFEFENTKYEYEVMKYVKSTNGAQLAHSMAMLYDRFTHLSIKQSQVDNIFIPPNGSFICIIPHQHAPMTAKNVDL

PMVFIARCNKSKTLQHIEYEHSFEGEVYRYYISKPCRLNIERLSNWTNASCKVVASSSYILSRCRSLNIQKDMLIGMLTV

LTIDSHQKVSEFTDLLKYISYMPFSQVHKLPDLIREKMDLMIKTRLDAWMLKKLKNFIIELSKIDLLNASKPKLKTFNTN

VMSESLGLEAALPSFVCPDIRHTNPSDFIEEMNIIHCCRAKHLYGSQFMDKSITNTVDWNIEYIDEVNKYGGWATDGIGD

GNFPFESKFCFSADAIYYANNELNARMGITPGKVETRLHSSGYARFMHENCSLRGCTKEMEDRDNQIDIHTTSMDACLQY

YKKIGYNDEKGTSNYIAYDALINGRDQHFSMSEKDQRGGGRPIATPTLSAKAVLMMIEKPEQARGRFMTNNIIVPNINKA

QAQCEAYKRAISDGIRLKYTKIYQLTEDQTKFSENDNVNKYLVYLRTNLDVDPSIRSIQYAALLQLHNRVHCVKRLPIDV

TNSQERLPYVVNTPNTLGVKAIIGWPQGMLNCISTNVHAGADIWITKAYNIAYPDNKVHTVGLVHSDDSWVTVCCNSEED

FKRYALFRIVAKKLFCLKLNIKKLWGSGMMGELVSNYNLNGNVHLSVAKIIPNSFGNLNYQNWVMDVHNQVSVLQQCYRN

GANLGTITMLATILKQQILHSYQVKGLQLKLLHELPVDIGGYPDVSPFELAVAGLPASYRDILDKMKREPGSEAAKIIKV

CLHWSIKRIVDEEQIIVSAFGNNTRRDFYTKLRDSYNDKWSDDDYENLVIPSKGDIFRCIRHIMPKSRKLAKTVSTIRNL

PFESNGLEMLVTRPKTLAEGLGHLKARASNMLYELAADKYTQNARRLAISQAIQSSGKVIKIGNLCPMSFNEFYTIALNS

ITPELSTNLISDAFDETNELVSCTKNIVYQSNHMRIGNDKRQVLSRMPIIDTKFRTISPLRDVLLRIIDMHMGTNYVEQY

SKLDVALRTLDDDARLIVRRFESYFNCHNIVTTCNLIMQQFLSVTRSRLWTQPHLRNETLLVFLEDLYGKTINSSFNFSI

VSSVQSSRALTTDVHTVQTLYNTCVLNNLYPGKFTMLTYDDRPISNVISQIDYKNFDDNDMCKYAVIQSIINKNDDFFKQ

MYNNNVYRQFYIVKQNYSPVYRRYSGYFKCVCTMGSTVIEISGEPEVSIELKSNTTAVNSILNIMKIFIERNFRYERYMT

SGLWGTKQFWKYVGISKGPYLCYHNQYSTVISPNPSWKSIPIEIDANLMFVGQVDNFIPTKYTISESLREIFYLTPNDEK

RRLASIHQNFSLPHRNHITLEPASIQQLDTSELYASGVMESLIMNDTKYINSAAIERLLLKSPYGQLSSMNVIVSFLKLL

SKAMGVNLTMPDIKKEVDNVVYSEELIGYTPEHVWTTYESQSPEDLSAVYVELAESQFERIGRLHVIKDLKRALAKAYIG

AVTDDERDSFIKCLLIDDRIKQWYSSIKKQDGKYLSFEEIIHDLEEMSVTPVNIKLYSFITELDLDTQDGWDKINLGSIN

NNVATDNIKPHVRELAKSYVKFIAETVFEEEYQIQPEISILDL

>Wuchang_Cockroach_Virus_1_(from_Blattella_germanica)_(YP009304995)

MDPFSLVHKKGKIDRQKENWREKRKNERALLPRDDVERQAFDINEQYNEMIKEWRNPNINPEDALELYLQARAIRHDNWI

RVLKLMRVSRGMRFGDIEVGKFCEELGIQNVPIEIARKTPDILSMSPDGTYIVLGDVTVSKSPKIAHSKKVVKYNSIHQF

FSNLGYNVKWNNFILEEQLTNVWSELYKMENDGIILVDKEKVNRTIHYHELLQKIMINLPTKTNNAQMFQMLLSRHDGLD

VQDVDIKLPASMIGCELENYEIMQTESQLMEELTSKTNELLKDGYFDCNINKVNSKINDIISSKENEEGDYADTKSTLKV

VFNTKTIEPNTGYDLIQDYIQDIQLGLDSINKDYLLDLLPSRTQVEKMKDLYKLRTEQLSKDESKKILQSYKDFRVYGPN

QYDMTRNSTCILTTHLCTKLKQGKKNRNVKTKPLNLNPAMFNEYKQNIDCMINYLGSESNKPVFLDDSWDSATNTENDHS

KDIREIYNIVKKTNGAQLAQGLSNMYQKIMHAKTSISVKDTIYVPPSGAFIMIKPRDHAPITAKNCDIPMLFIAREKHSQ

DTQTIQSFLEHEVVYKGDEYIYFVSKLSRLNLNKFSNWDQAGYKLVTACTHLINICPNLKGDLSTVVGLLTILLLDSHQK

VSEYLDLLKYISFMPFADISRLDKLVEDKLDLLMKTKMDVWLILRIKEFMNELSDINRLNAKKPKLHMFNCQATRESLGL

SINLPSFTNPRIRHGTVQEFIEEITMLYIIRPKQLYGSQFMDKSITQTAEWNNEYNEEVAKHGGWATVGNEDSPYPFDAK

FTYNRSAIMHAHEYFKKTIPLDDNKIAKELHRTVYDTYMHYNCSLRGCTKYISDRKNNNDLHTTSMEACLAHYRNSCYKE

EQCTTIGVVDDFINRGEVMQFSMSEKEQRGGGRPIATPTLGAKAGLMLIEKPEQVIGKFVPNNIIVPGKHKLKEQAETYK

DLLSEGAKHGMSKVYQLTEDQTKYSENDNVKKYYCYIANNSVLPINVRIMQTEMLKRLEKREHLVKRMPTRINSEYSLSQ

HRNKELNGIKAIVGWPQGMLNDLSTSLHSIADYWITYMYNKCYPNNPVIAKGLVHSDDSWVAVACDSENDFKRFAIFRML

AKKYFCLKLNDKKLWGSKHLGELVSNYNINGNVHLSISKMLANSMNNLLYQNWVMDVNNQISSIQQLYRAGARLPTLIML

STVLRQQLMACYNVSGLQKEYLNILPIELGGYPSSSAFELGVTGLTSHYNRLIEFTKNNPNHPITDIMRKSLLASNVLNA

AKKLGKEDPSTYAISKCMEYPTDVSQLVPNAQLQVEIENDYAETPIPSRGEVFSCVKHLMPKSNKLTKTLEQIKDLPFDT

DNLEMIVTRPDELTVSLGHLKHQLTTRLYELASEHYTQSARRLAVSQALQASGKVVRFLNLTPMTINELLQAILDLPIRL

AKPDLLQIAMTDDTDVSNLCISIVHNSELVACDADKRKIINRMPEIIDKFTTVASLKNVLLYTIDTVRRTDYLNKYGYKI

EPLDILRNDSRLIHLRFNIYFRYFQIEQACNMIMRLFMFRERARLWMHPYLKTDTLSNFLEDLYGKSVSDVDNFKVKITN

PYIMGNRDDANMVNTMYSVNVLNNIYPGKFDLQEIKTMSPVEALDKIDYENLDNEEFLKYAVLRKLYCNTDWYLNEYDKS

KVYDQEFIVKQEYDEKHKKYFGYFELLARYGKTILKVSGEPEDVEIESSTNNINEILHCMFILVNRNFTFYKYQHYTYWS

RSMFWKSKPKFSRTFLSNYHTTCTLITQTKSESSVPFLINKNLARPVSYGCLEAIGYELTENLRVVVKITKDSSQRLDNI

HQNLQCPYASDCVLIGNLLDGLDNEELLHSGIIISVSNKRYYNCSRTRILGLLESRSPPLSNSILVDMYTNFVNKLADDN

QVPTRHIQHINEEVEYFTISGETVAERLIKYEETTPEDLTYVELEYEMDSDERVGCLVKYDNLSRTLCEILYPRLTDYQQ

DLLIHMLLADRKIVTKVISDLDRDPCDEEESLAQRIETLHDAASCMRHPTFVNYFIYSNQLDVEATWANIDVRKVMYLVP

ENIDSQLKKVFTLLRNSLIESIFEETEEDVIQMLRDRLFKH

>Wuhan_mosquito_virus_1_(from_Culex_tritaeniorhynchus)_(YP009305130)

MDQYKSYVGKVDREMVMDRHSDRVYSRMTSCTRVHARINDIKKQLDKSLTPEEGANLYLEVRSLRHDNWCEFINETCKIR

GAVIEDTKVGEFVREYISNEVNVPAEISNKTPDILVLGNNQNVVYLGDVSVSINPSVARARKHEKYLPIKNYLNSLGITV

QTLSFNVNENCTNLASEINHLVRHDFIESHPDTVALYETYSIACNSLMLSIREVVDDKKAMQDMIDRADNIKFDNPIMPD

IPFEIRSSVDLEYVPTLSEDELIEMIKAETDIKHNGKYFSKNISDSIDAINKIVDNNANISHMAPKSTLKVVDNSYDLNV

ETDLSLIRDYVDDILNADDSNAKELVLHLLPTLTQLDMMKKAGDKKMTHTECKQDQEMKANGVFGKYQYKIQSTYCSSLV

NKYKAEIVKGKKDPNVKKEPSTINLSSIESYHNFISNSIDYYGSLSKKPQVLSDDWEAATKFENDSTILEKEMYDYTIRT

NGAQLCQSMAHLYDRVSHLSTSLSIKDNVFIPPNGSFIAVIPGNHAPVTSKNVDLPFIFITRCNKEKVLHHIEHEHYFES

DKFGYYVSKLCRLNVDKMSNWANASFKLISTASYLLSKSRTLQTSRTKVIGTLTYFILDVHQKTSEYLDLFKYISFMPFA

DISRLPNLISDKMDLLMKTRMDGWMLHQLKWFIRELGDKSKLNAVKPVIKKFNTDIVSSSLGITMCLPSFCNPDLRYRKP

EDFIEEINVMNTVRPKHLYGSQFMDKSITQTCEWNDEYSAEVIKFGDWAVNGCGDGSFPFDSKFCYSSDAIYYAEQSIVK

EYSLSKSRVEQKLGSSMYSGYMHKNCNLRGCTKNKSDRSNAADIHTTSLEACLREYERQNFRDEKCTAIAFGIRHIMSGD

VQQYSMSEKDQRGGGRPIATPTIGTKAALMLIEKPEAAKGHHMLNNIIVGGKNKLREQCETYKSAISEGTRRGYKMVFQL

TEDQTKYSENDNTRKFLTYVKCNKTLSPDVRALQLKVLENMIGREHLIKRMPICVKSSQALSKYIIDSSDSLGVSAYIGW

PQGMLNFISTNVHCAADIWITKAYNKAYPNSNVYTKGLVHSDDSWVVVCCNSVDDFERFTIFRMLAKKMFCLKLNEKKLW

GSRYMGELVSNYNLNGNVHLSVGKMLANSFGNLSFQNWPVDVHNQISSLQQCYRNGAGLGIIIMLATLLKQQMVRTYNVK

GSQLENLGVLPIELGGYPSCSAFELAVTGVTCYYKKLLNICRMKPDSITSDCITKCLLWSIKRVSISEEHVKVDNKSKGR

LEYYSKLKDKTLEWCEEDYVDLSIPSRGNVFSALTHILPKSRKLSKTLKYIDTIRDKFPTNGLELIVTKPLSLSESLGHL

CAQTTGMVYELAADKYSSSTRRMAISQMIQSTGKVIKIKGMIPMTINELLNFIELSEMENKLDIDILASAFSDDNDVVGI

TNDIVYHSTHEIADDDKRKIITKMPFIDDKFNTIGRFSDVLLKIIDLESKTDMLYKYSKPNVSDETLVVDAMNLMRVFKS

YFKFYEVKAACSLLAQQYFQTLKARLWTQPHMRSDCLTNFLCDLYGKTISNDKNYRVYIHVGRSYERSHDQDITRTIYFT

DVLNHIYHGKFIIESVGERSIGDVINDIDYTKLNDDDMLKYSIIQQLHNYNDDYLRKMHSAGGYRDKWEIAQRRIGRVYK

GKFSALCSLGQVVVRISGEEGDLSIESNKTDVIAIMHIMQKFVQKNFSYSKYNIHGSWGESPFWRSKNQFSTHYLTYYNR

ISTVITSERKLCSIPIKIDSSLSYGEEPIGSIVDGFSFDNMLRVVYVIVNGSKYKFLTARQNFTLPCRDEINLEMNFIQG

FDCRKLYSTGAIEHLVLDSSHNISSNVIASLLETGFNTINSEPIGQMLTNLISTINRRQYLKYIQSSIAQEEIHEVVLEG

VTVKEVVDIDETTSPEDLGCIDIEYVEGMRERSSGICRVLDINRVLCMAYIGKISTKQVESVIYHLLHNESMIDWAKHVM

SAYEPDPAIYFDYLDTLSSYRGSSISLRTYAFIISSGLNVTDTWLDLDIINIAKLTDEVDHSQYLKKISQEIVEVNAEFH

GFTVETKQSLADLVR

>Ganda_bee_virus_(from_Osmia_cornuta)_(APT68154)

MERFFDREGRADRTRRFRGPRRGSEVDLQATSQIEVSIKLIREQLDENWRSLPHTKALNCYLNARSLRHDLWLDFLKDNR

SRNEFTFTDTRVLDFVNKLKTDRIVISNIEVPSDIAYKTPDMLIYNSYNKCVILGDISVSSSVVLAEQRKYEKYLKVKNF

LIEIGMRVQHVNFIVDEDLHNIDRLVNMFYNYGIISINQQSLLKTRTYHLTCNKLMSDAKNHCDDKIKFNEILALTDRSY

EYEGLNIDYEPSLNLKYEKAIRTEDEIVNMIKNECDNLCDKYFDNGFEDAKIKFNELIESFKNREHMKPKATLKVIHNSR

DLEEFSGHKLIRSYLMDIAYADEEMISNYVLNLLPNSEQLADMETKFNDNVIIDKEELKRMKVYGKWQYNIIVKYHGNLL

TLDTKEKLTRGKRNPNEKKEPKTIDPDNYDNIFAELNMYIEQLGSISNKMPFLSDEWDAMTNNEMDQTQDEKEIYNYVRR

TQGPQLGQSLSMLYQRLTHLKTNLSMKDNIFVPPNGSFICIIPKEHQPFTGSKADVPLIFITRVLKQNNNKIFEHEFTFS

TNNYIYYVSKLCRLSLDKIAHWDQSGYKIVACSTYLISTCPVLHNIMNKVIGVISILSLDLHQKTSELLDLLKYVSFMPF

ADISRVSKLITDKFDLMLKTPLDVWVLLTIQSFMIRLSEPGNVSGLKPKLKVFNGIVLHDSLGMEIKIPSLLNIEGKHSK

IQHFIEEISMINIIRGKQFYGSQFMDKSITLTAKWNDEFVEEQEKYQGWTEGHSNTVFPFDAKFCFSKDAIIYAMEYFNT

NYPINKNVILNKLSKIEYNVFPHYLCSLRGCTKEKEFRKNNSDLHTTSLDACLDYYMATGYDNKIANTVVMSKTFHNEEY

VAQYSMSEKEQRGGGRPIATPTLLTKMGNVMIEKPEQAIGTYTRNNILVAGKHKLKTQSETYKELIEEGYARGLKQVYQC

TEDQSKFSENDNTRKYYTYIRNNPLLPEAVRELQIKSMYKMIGREHLVKRLPTQVKNDHDLIKYVNSDSNGVIAIIGWPQ

GMLNNISTSIHSIADYWITYVFNKAYGTNIITKGLVHSDDSWYAIACNDKETFIRFAVFRALAKKLFCLKLNDKKLWGSK

MLGELVSNFNINGEVLVPVAKVIANGFGNLLYQNWVIDVHTQISTIQQVYRNGCNIGCLVMLATVLRQQIISAYNLHLKP

NPMLYTLPIEMGGYPKCSAFELGVAGVNSHYKQIFDYVNKNPRSKLAIIVLRTMNLSMQYNIAREESDVLYHIDIKSKTN

LSHLYESTTVLEQGAYESIVVPKRGEVFSCIKHIMPKSKKISLTVKKIQNLPFETDNLELLVTRPKDLGVALGHLKSQMS

TILFSLASEKYTGSKKRLAINQSIQATGKTVQIAGLRPMTLKEMTETILMMDGVPLASIDNLKISFEDDTNIVGICSDIV

YHSDISLKNFDKRKCINRMPDFEDKYRTICPLRNVLLHIIDRVRKTNFRDEFTGSSDPIELIDQDADLVLKRFSTYFAYY

SVEYACNLIMQQYFSRIQPRLWTQCKIRNDDLTNFLSDLYGCSLNNTANFNINIDVTITPIGHDDNNLISSMYTVEVLNT

LYQGQFKMDFIGNKTVNKTLELIDYANLSQSNYLKFGILKYIYDYNMRYLVDYDNKKVYSQYYLKAQKVKDGTYKGDFKV

YIKYGNLVMIVEGEPEDLLITVNNNNIQDITMAMFIYVNRDHVDYAYNYYGSWNLNKFWASKIYQTELKLMCYGQNMTVI

KKGAPGEGVSIIINKNIKYPIREPVDIPDRFEFDNGLRVVYKIYKNTRIRVDNVKQNMNCPYKDKITLQTEYIDGFSNNE

LLKTGVILNITMRRSFSVAQTSISRLLDNRVPATNMKPVLASYLRFAKVINKEILDDIPETLSGVETVIEAIGGMDPESY

MEEHEKTSPEDLTGDEVGIIFSESERAGALTIHGSFTRLFCKYALSPFLSQEIEDFMLILFKNKKFIEMIKDVGNQINNE

ELDIDMFIEMGEDMEFNKRLYLFIVSNELDNDKYINRTFIRKSLSDTNRIISSPAIINWVDLYVKILNQALEDEGEDDFQ

DIINLLD

>Culex_phasma-like_virus_(from_Culex_sp.)_(ASA47399)

MDPGEGTSLSVSSLAKELSERENKAERKDRELQKSLMDKIENLVVVQRSPFVVIKDNSSILSKEELSVAQAITVYENVME

ARHNVWYDLIYHVKFPNWRDNGAESTFRAFCKDLLQVDIGDADDKNYRPDLLLFNKRVNEVIIGDITVTSNISAATTRKH

EKYACLVAILEQFDIFKVTHLDFVLREDMSNLRQMINKFINAGILPNDIDYTVHRNYCASATDLMVAVKSLCPDKQAFVL

GLESKRRKQEMEGDKRNYSLFEGLLGDTIKDIALVPYVPRRTEDEIINMIKVEVERLGGASYFKTTQKEVEAAFDSVLEK

NKGRKVMPPKSTLKVIDNSHSIVDKTGIELIIDMVLDIASGPPEDDVKGYLLDLLPSYVQLEKMKSVRDNKMTTEEVKGN

DDLKEARVYGQYQYKRVQGGNNLLTQNLTVECKKGSKNNRNAKKEPAVIDLENMNHYESFITNSIKYYGEVSNKPHFLDD

SWDAYNKLEEDNTLVEREQYRYVKSTNGAQLCNSMSGLYNRIMHMSTRQGKYDNIYIPPNGSFICIIPDDHAPVDRATCD

LPMIFITRTSINDSLNHIEAEHQYSTTEFTYYISKLCRLNVGKIANWNSAGERLCSSATFLLSKCKATQQCKAEVVGLLT

YLILDVHQKVSEYLDLLKYISFMPFAELNRLPSLIVDKCDLLMKTKMDAWMLNRIRSYIRELHDVSKLQAEKPTPVVCNG

MVHTDSLGITMKLPSFFNVMIRHNDPTEFIEEVSMLFTSRPKQLYGSQFLDMSITMTAQWQLDYDKEKEEYGGWADKGYG

DGDFPFTARFCYSSDAIYYATQQFEKHVEATGTKIENHLFSGTYSGFMHENCSLRGCTIEKEKRQNQDNMHTTSIGACLE

LYKEKGFDERSCRAISIAQEFIDGKKTMEFSMSQKEQRGSGRPIATPTLGTKAVLMMVEKPEASIGTFVKNNILVAGRNK

LKEQHLAYKETISVGLNQGLTKVYQLTEDQTKYSENDNPHKYEVYLKSSTLLKPDIRRLQLAGLRMLYNRRHLVHRLPEK

VMTDNDLHKQAYTDANTRGVKTEIGWPQGMLNNLSTTIHSMCDYWVARAFKIAYPNIKIYARGLVHSDDSWVTVCVNNKD

DFLLFSLFRMVAKKLFCLKINEKKLWGGKHLGELVSNYNLNGRVHLTVSKSISNGMSNLTYHNWPIDVNNQISVIQQVYR

SGGKIGVMIMLMTILRQQIMHAYNVDGLQKKLLHQLPIELGGFPRCSVFKLGVGGSNAAYDELAEQYRQGNFEEAYKLVC

AAAAVTRITIDNGADTVKDENWADDDYAEVSLPSKGELLKGVKFLLPKSSKLKHSLQMIYEVTKNYPDDGLGMIITKPIT

LAESLGHLRQNTSGTLYQLATEGFGQSKRRLAISQAQQASGKVVKVYNAGTMTMEELYNYLKTMIINEEDKLMVNQALQP

DNEVIWACNQIVETAIIHDAHKETRGNVINRMPEFDTKYDTISPIKDVLLHIIDSHYAKINKPTNYYDKYGTNYTCTETL

KNDAANIISRFPSYFCYYNVVRACSIIMQNKYNTIKERTWVQPRIESDTMTGFLESLYGVSLREGMVYKIYSQPIRSKQS

ESDSAVVKSLYTAEVMDSIYPGNFRIEKIGEKDAIDALKAVDPIKLNKNDSLKYGLLMMTMCNNTDYLRKIYADDKFVYD

WIKRQRYYNGRYIGDWCVRYQIGTHSGEISHLNGMTYIKSSTISLPPILSAMRLIASRCFNMNYEFDNGWSSCQLWSFRS

MPRSGYTYYLKSRSFLSTTIINSPEEGCIPLTLDPTVRIRNIVVVKEPESFETDSALRVIYVNLLGDDGYPHKYRMESIR

QDLSSPLKNQMTFEPTFLDGFIAEELFKEGIMEDLMLNRPIGAPISTIRRMIGNAISTNKSRPVWNVTRSIYNKLHGLGL

PTLANAEDPIVDHIVYDWVIEDRGFVEHVEFHKTTDVESLDAAYVDYETDSIKGQNKIKKTRSIMRVLCKQLWASLTVNN

LFDLLGKIINHKVTDIMHEKGNEEITEIDEDDEWILENPPSTDLFSLVYGKDMDTDAFWRTVTRLRLEHASLHPSPFENE

DRLLADYAKFVSNVLDVDEPKPVRSKFEMFR

>Whenzhou_Shrimp_Virus_2_(from_Penaeus_monodon)_(AJG39257)

MEDIIEVLHYLDERLYNEPLSSGGGWRMDLAEEVLLEVRDLEVGRRVTEEATHMKATEAEEVCERWEELR

HDLLAARLLTGIFSTLQVDFSFSRILDDLYEKNIIEDREYDFDPRKVEELNKEVPVNQFMVSGFLTGNSI

DRERLRRIVGLLFRKTPDNFIIERGSLILMDYASSFNPRLAINNKLAKYGNECRRFKMLLDAVGIEIDCS

TSAIVADLINNQLLRGTEHGVEVIDLDWPEYFEVFGMNIRQLFRVSLSREEAEAFEPEEEEELETFYELG

MREATERIIAGTGFNEWLEDLKQRSREPHLFERLYEMNSADSNISDRTSLPYILEPMVKELMSDLMAEAE

SMDNPFEVPSPEEIKRAFKNFHGRIRERNSKFLRTDDPKPNFHFAWPISSEVMESELGDTKRQVMINLLR

GEQSDLLGDYPKVTLSMALIVLANLSQEEFRGYQEKGLVCDVKDEKDMEFVKEVRGHKFGVPKKSGKKKR

NTVYLKKPGEDLEEIYSEAKIDMPDEDKAAEKAENAKQVKTGQPYIESLPFWDVKQRRLHEQNWKEEKSH

LTIPLMKDNTDEDEDINWLCKTAAYRCLSDVSVLFKNIMFTAQTHRRNRFSIATGPSPYQAALVLPGEDL

QSSNGVSFCHIYKRRRGQEVISGLREKTLCTLEDGHLYRTQTVRLDKVRLKSLSTSFPKFVILFKVLVNL

IEKRRVKEKKFGRDKEEIYIMACEMAFYSVTNVNINTSSLMDNVRYLVLGAAADYSGASAFIEDKMEVAI

KTTFQMHLYDRTLKLLSKMHGSKLEEGIEGIEGAYFGNFWGLTGDDIYSDLVDVIQESYAIFFSVEKGMH

NKFHNMVSIHKTAFDYQQKVEGVKDFINLDISRGASFTFNPKVLMLSSFMTNSFSSKVINRVRRRFNVSE

RPFKHPLTINTMSSLKKCAAENRGRKELDLKDMDAMFRRDVDMEEEDENVRLNNAIPYELRGTFEYVDKE

LQYKKQKKLRGDALKRALKSMNWVDYIDAGSRPVLDEAVRLMRSPDHSANGDAKLNYHIANKLMMDKTME

MSLDKSLQQLALVPNTRIARVVPKMQRTQKDREIYLVSIQYKTSLYFIEHFMKQVNKEVEEEWITIPGDQ

KFVELFKDREGGEGHSKITGDCSKFSAEDNQDKFIFNIVANSCLTRSEKTLFFVILYLYTKKRLLLSDDM

CSYLRNMSASEAGCIHENPFFGLTDGLKRNYAILNKNWLQGQLNYLSSNVHACCARLIKKTSETHQQSRV

SYAVHSDDFKVNIKHGPNFDISTFLCLLDLVMNLHTLKLNYKKSSVHKDYLEMVSQIIYHGEARPSWVKS

LISVVSALPYTGFEQDRDATVSKVATALSNGCPPSVASVALRYIMEETSRVYSMHKTGANSVLRIFNKSN

QEVPTFLGGWGNEDTFALSALGSKAHDVTLLVDFLKGVYSGMELNMVFKRSFPLFEDFIGDIKRVLEMYP

ELAEILRGSWLLKVFSKRLSLIPNEAVEDWDDMATVGGLFRFKRFVNAKMKYLSWLYQMYPDDEGWTELK

KDWMLKNPSYSIVKPQTDKDLLIYYRILLDNSAFLRSYTNQSQEQLLINRVRSSKDKIMSLPVKDLTIHE

SAASKSGIMSGDMVTIKEGLEWFRDLDENITNDDIVRVVTVWLISNTRVPTWLNVITQTTTTSALYYKQS

VPRRMPKPEGKRVYSAPVTTMLQYLYDIENFRKDGKELIVNDAVKADLSFISKTVKEIFGDIAQVDLWKN

LRLLVKFLTADDSHKIYMMPTTNIGALSNMMSQIYRSSKNDFFMVGGETMSMIVSGDSIGKKDNQTIACN

LCKRLSEIYPHSENDRFFPFLKDLLNRVYIGDEKAISILTESPRHSSKNFKIWHYILTGEDLGISEEDYD

RSHIAVKKEQIYDESLKGYRGPFVVVGTIRRGQLVTQAQMEGVDQYVTDLSYTGSRWHAALILTDINHFV

QKKWKLSLNFNNLIVEAEPEGFIIIKKVITLNNKKNFSDYLGLQVRQKEGKEILRQIRKSVAGRSSVKIS

YSNMTHQIIETHFVAGKNLRGHCTVPEGNLEFPVTVVWKGVVTEANCNARICIEEGLLDYKNLSESLKSR

RWAKAIFSGSVIISEETYKESNLKPTLSLVETSEHIDLDSILNELGLKNITLPNGVLEQFTHRVSKLGLD

LIQISTVVTSGDAKVDWVLEDDMDSEEVIGDFKKLLRKKKITEDDMHLMKRLANHLNLDYEDSEVSSSIK

S

>Wuhan_Louse_Fly_Virus_1_(from_unclassified_Hippoboscoidea)_(AJG39233)

MEYNTYNQFLARIRGCSDPALAKDIDTDILVARHDYFGRELCKTLDIEYRNDIPLVDILLEVKNDFDPLS

INIPLMTPDNYLVANGKLFIIDYKVSVSRESSIQTLNKYTGILEEVCPKIGIDFEIAIVRANPITSEITF

SSDEFRNLFGALNFDLDFSQFLELKRILYEKFADNEEFLLKVSHGDFTITAPWCKSGVKDLERHPIMKDF

KKSLPFSYRRLFEESIRFNPYQAEKWNMQLIRLKEYTAEKYHNFIKDHTKKIFECTGNFERPSSSEIQKG

WIEMTERMNETRILSTELCDQKPSAHFIWSKPDENYSNNNIQKLLRLSKSLQQINGVDTIDSAFKSIGLC

MDFSDNIEEYNSICIMRKNEARKYVGQVQNKKIEPVKIGKSLIYWEQQFGLSNENFEGNTRVHFMKDYCG

IGKHKNFKNKMLEDLELDKPKILDFNDDKIYLSSKQMIEQTSKILNQESNLDINKDFIYKNFKNNILESS

EKMLETLETILSSKYWSCINDISILIKNIISISQYNRHNTFRVATSPNNNLFGILMPSTDIKSKRSTVVY

FIITLHKEKDSVLNPGCLYKTYKTKDGYISISKAIRLDKERCQRLVSSPGLFLLTTTLFKYYSPDSSLSD

IMPFSFYTSVSITKSLLTLTEPARYMIMNSLATSSHVREYISEKFSPNTKTLFSVYVTRKIKNACFTANK

QREKIQLRNISLTDYDITQKGVEDNTDFDSIWFPGKVSLKGYINQIYLPFYFNAKGLHEKHHVMIDLAKT

VLEIEKDQRINCPIPWSEDYHKQSVNLPIFLHSLAKNLILDTSRHNHLRSKIENKNNFNRSITTISTFTS

SKSCIKIGDYQDLKEKIDQTTKKAIKANIKRTRVANNNFITDEEIDNEIKHSNYTDLRKSIPDYRDYIST

KVFDRLYELLKLNLIDGSKTAIQNIMSMMLEHKEFYFTFFNKGQKTAKDREIFVGEFEAKMCMYAVERIS

KERCKLNPDEMISEPGDSKMKVLEQKAEQEIRYIIEQTRQKNKNLEMNQNLYKATKIEINADMSKWSAQD

VFYKYFWLIALDPILYPAEKERIIYFLCNYMEKRLILPDELLCNILDQRKTYSDDIISEMTNMMSSNYVN

IKRNWLQGNFNYTSSYVHSCAMSVYKDIIKEISQLIEGDVLINSLVHSDDNHTSITYIHDRLDSDILIEH

AIETFEKICLAFGCQANMKKTYINNVIKEFVSLFCISGEPFSIFGRFLLTSVGDCAYIGPYEDMASRLTS

TQTAIKHGCPPSLAWLSIAVNHWMTFTTYNMLPGQVNDPLLRLPCKERTELPIELFGILNADLSTIALIG

LESSNITFLTQLIQRMSPIDIQKESIIDQCVKIPKWDLRQLSESEIFRLKIMRYLILDSEIDVDSIMGET

SEMRGRSLITPRKFTTIGSLKKLISFNDYQQRLSQKDGMEEIFEFLLNKPELLVTKGEEKDDYMNSILFR

YNSKKFKESLSIQNSVQLFIEQILFSHKPVIDYSGIRDKYSLIGDNLELEENPTIIGRMTFNQTYEQLSK

DLAGFKLDNEDINLVYQFMILNDPLLITVANSILFKINGIFQKRTGLTCNNMPELRNLKLIHHSPALVLR

AYSRNTPDIPGCEIEEMRRDLIHLEEFIEKTKLKEKMDQRIINNQMAIGMRDLKFELSEYTRFYQICYEY

VKSTEHKVKVFILPCKAYTTIDFCSILQGNLLRDDEWVSIHYLKNITAGGYKAIVQKTNVSEINTALEAI

RLLAYFTDTFVNNYSKKQFIQEVIDKFTYKNVPISKFYEILLNSHLRHEIIPFLFITGHLNQKDLDKYDA

MKTAETITWNNWQINRSLQTGPIDLKISAHNKSIRIIGADDLLQIAELQVTRMNYDNIILAGRRLLMSRH

GLKFETFKKIQFQDEIDYYITYQKKGKNQFAYQIHNKDSIIRRNTDNLNQRTRYYNEIVPVCPLLVAEMQ

VRSRINIRQLNYLNHDENYAGKIKIDIDEYIVMRRVQLYKMQNFEGPDIKSGLINFRVLMKTPELLNTNY

NKISQSSILSFSRILTCTGNKYEDTLEFLSDEPLDIEEESLIESTPIFSVTYMKKGKKNMSYKSAIEQLI

LNGVEEIENVLDFSKNGFVSNENLGILEVIVSSINLLNTNEWSTMLLNCLHIALIKNGYDAEFHMFNMPD

YFLENKIEFKVDWIKLREFIITLPDLDKEPWNSMFQRFKEKAINSIDKEVKKTKRFTSYLEVLKKRGGRS

MFDFE

>Athtab_Virus_(from_Cherax_quadricarinatus)_(AXY54926)

MDLDSLSDRLKQLRLLLYEKNVNLMSYYDFQGTLIDHILRSVEDLNPRLVIQMNDKRFSASQANTFCEEW

EMLRHDVYTACMIQSVGQTQFVVDQPFSVMVDEMLPFEEKYYGNLDFMQSMTNNFAIGVKIGLREWTKRD

LLDIISDTMRRTPDNYKIEKQELTLIDFAVSINPNRAIEEKFRKYTEDCETLIKFFSAIGYTINSSVLVY

CGDIANNRDLIASSEVDTKIEHRVQGEFWDAFSWNNRIYLSQFMNDEEKELFQEVDPEEVQVFYNLSINE

KAARAIMTTDFHTWLDNLVEASGQRDLFRKLTKNYNADTDPNLRAAVRDSCAEQIYSLERDAIEAAEEME

SPFEKPTPRSVKKAFANFHKRILEGNSKFSRVDRAKPNFHFVWPVTADKITSDNGDTKQEVIRNVTMNCP

GSYLDVDKCTDLMHIMIFLSNLSRSDFALFQKKGLVCDNFFEESSVENTIRGRNMYIPKQAGKKRQGTIS

LKRVGPHLRHKLDEAKVGMSKEEKIKEKEENTRNVRSNTPNVESLPFWNQKERKLHVQNWKDESDSLKIK

IEEVKDDDAEMSYLRGTSSYNCLYDISILFKNILFLSQSHRRNRFAIATCPNPYMFAVILPGEDLQSSNG

CSFYYVFKQRRGTPLISGLEVELMWETEEGFIYASQTVRLDKVRLRTLSVCWAKFCILYKCLCSLTAKRN

FKMALADVVSKDYMELCMIMSFYSVTNLSINMSSLMDNVRYLVLGAAGEYSGVSEFIDEKMDVAIKTSFQ

MFLYDRTLNLLANVGGVELSEISDIEGAYVGHFSGLTGDDMISDLVDVIQESYSIFFAVEKGLHNYFHAM

VDIHKVAFDYQCKFLMCKNPVNKSMTYGENFTYNPELIMISSMMCNNFTQKEINRIRRGFKVLERPYKHP

LTIKTMSSLKKCVSPRPKQNTISMAELDKKIRQNTRDELDELNDAIHFSLKSIYVYEKIDGKNVLTMKKQ

TKLRGVALGKALKLEGFTHFIEASSQTVVDAVTNLIQSPYPKVPDADISEIPLHVEEQVDMNLTEMLSKS

ICWLVIDPDRRLARVVRKFQRTQKDREIYLVSIEYKVSLYFVEHFYKQVNRAIPEEWISISGDAKYLELF

KEKAPEDWYNLTGDCSKWSAGDNQDKFIFNIAGNCALTESEKYLFMFLIYLYRKKKLLLDNEMCNYLKRM

SESQRGAIHENPFFLLTDGVMKNYADITHNWLQGQLNYLSSNVHACCARFIKNCAEKHLQMKVNYAVHSD

DFKIEYHLRKLENHEPFLCLVDLIMNLHCLKINKKKSFIHQKYLEMVSMIIYLGEARPSWVKLAMSVISG

LPYLGFEQDRDSALSKIQAAILMGCPKSVACIAQSMAMKEVFRTYSMHETGVNFPGRIFSKHIKEIPSFL

GGWGSDDCYDIATLGTKGQDVKLLYNEMNRLHKIHTLKRPRLYRLEDFIYNFEDLDAESKLELQDEIENS

WIIQAFIKRLKLVENNLQDEWETSDVLGSLFRFKRFINVALKYDTLLYKKFPDGEDWQEKKDLWMQKHPS

YSLMKPQTNDDLNIYFRLLLDNPSFLRSYTNQSQEQLLINRVRSSRDRIMSLPIDRDSLDPLFTGNKGVA

EGDNVTIVEGLSWFKYESEVKINMEDLTLALNVWIRSDPKVRTWLNVVQNTTMGKGMSLKLFYPRRMPEP

ESHRVFSAPITNILQWIYDKETFEKDGKELTMNKSLTRDVEFIRDFVRKTFGDSFEKDKWKNLRLVFKLL

TGGEMGRIYMAPTSEIGSLSQMWCQMYRSCKQDFTIVEGDVLENIDGPVIEHIEYFDQATAVTNLCYKIS

SHHPPVDNPVAYKKYFINILKNVWVGNGDAYEFLNDVNKNSSKNLKIWKFFVHGKDEGLNEEIWRNTQII

VRRAQTFSDKQKRYVGVFKLLGVIQHKTVTVVEFYGDQNGLLEINYSGDSYLATNIMNSLHNYLEKKWGL

KVSYNNLFIEASRGGFVLQDGRVKLFRKKLETHIGIKIERNSELSKITLRDSKVIVASDDGLYVDLMYGD

DLVRLETHFVERTKSMRRCLSCKPDLKFLVSTLFGGSREVREASLLRSINEGLLDVRSIKSVSLNVNWLR

VLADKHISSEFLVLRPSQFVMGKVEKLSLINLDELLASRGLKKVTLPREVLEMSISEKREIQDLIGVGMA

ITNNSAKMDWYKEYDTDDEDHEYPLPEVEIMSRVSKAYRFGKITESDKLLMHCIIHDKLPDDFKK
