# Supplementary material for: Genomic and developmental characterisation of a novel bunyavirus infecting the crustacean Carcinus maenas
Source: Sci Rep. 2019 Sep 10;9:12957. doi: 10.1038/s41598-019-49260-4 (PMC6736955; doi:10.1038/s41598-019-49260-4)
Supplement: Supplementary file 2 — Supplementary Figure 1 [file 41598_2019_49260_MOESM2_ESM.docx]

**Genomic and developmental characterisation of a novel bunyavirus infecting the crustacean *Carcinus maenas***

Jamie Bojko^1,2*^, Kuttichantran Subramaniam^3^, Thomas B. Waltzek^3^, Grant D. Stentiford^4,5^, Donald C. Behringer^1,2*^

Suppl. Figure 1: A concatenated phylogenetic tree developed from the L and M segment (LM) proteins of 31 bunyaviruses. The protein sequence data were aligned separately in Geneious using MAFFT default protocol and then merged. The tree was developed from the merged data using IQ-tree. The corresponding viral family within the *Bunyavirales* is highlighted on the tree, including the crustacean-infecting viruses, *Cruliviridae* and ‘Carcinus maenas Portunibunyavirus 1’ in bold. The accession numbers for the L and M proteins, according to the NCBI repository, are presented after each viral isolate used in the tree.
